# HFIP-Induced Formation of *O*‑Aryl Oxyallyl Cation and Nucleophilic Addition with Sodium Sulfinate Salt

**DOI:** 10.1021/acs.joc.6c00351

**Published:** 2026-05-26

**Authors:** Jhonny Aldás-Bedón, Adenike O. Adebanji, Raju S. Thombal, Estefania Armendariz-Gonzalez, Edward W. Mureka, Frank R. Fronczek, Rendy Kartika

**Affiliations:** 232 Choppin Hall, Department of Chemistry, 5779 Louisiana State University, Baton Rouge, Louisiana 70803, United States

## Abstract

We report a new reactivity of α-hydroxy *O*-aryl enol ethers, which can be ionized in 1,1,1,3,3,3-hexafluoroisopropanol (HFIP) to generate *O*-aryl oxyallyl cation intermediates. In the presence of sodium sulfinate salts, nucleophilic addition occurs to furnish α-sulfonyl enol ethers in high yield as a single regioisomer. The resulting α-sulfonyl enol ether adducts are highly versatile for applications in postfunctionalization reactions to create structurally diverse value-added compounds.

## Graphical abstract

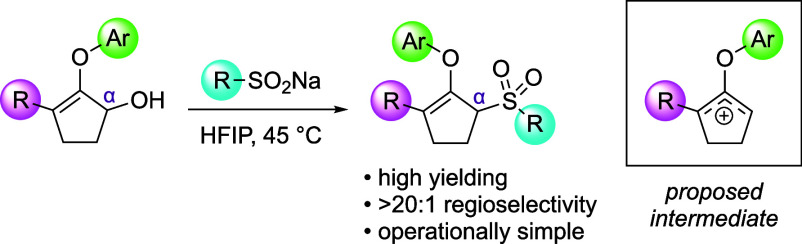

## Introduction

Sulfones are a class of organosulfur compounds with broad significance in organic chemistry. As synthetic building blocks, sulfones exhibit unique reactivity that can be tuned by reaction conditions to enable numerous synthetic reactions.^1^ As a functional group, sulfones are commonly found in pharmaceuticals,^2^ agrichemicals,^3^ and materials.^4^ Because of their vast importance, the development of methods for the synthesis of sulfones has attracted significant interest.^5^ A particularly interesting approach to creating sulfones features the versatile reactivity of sodium sulfinate salts.^6^ Specifically, owing to the nucleophilicity of the sulfur atom, sodium sulfinates are known to attack carbon electrophiles to form the sulfone functionality.^6a^ A notable reaction in this context is allylic sulfonation, which requires electrophilic allylic species to form the carbon–sulfur bond.

Examples of allylic sulfonation reactions often rely on the Tsuji–Trost approach, which employs allylic esters or carbonates as starting materials, activated by transition-metal catalysts.^7^ Examples of the catalyst-free conditions have also been reported.^8^ There are also precedents for allylic sulfonation using allylic alcohols and amines.^9,10^ As shown in Scheme 1, Chandrasekhar demonstrated that activation of cinnamyl alcohol **1a** with catalytic Pd­(OAc)_2_ in the presence of BEt_3_ enabled allylic substitution with sodium benzenesulfinate to produce sulfone **1c**.^9d^ Tian discovered that cinnamyl amine **1b** could be activated by a [Pd­(allyl)­Cl]_2_ catalytic system at a very low loading to produce allylic sulfone **1c**.^10^ In these examples, allyl palladium complex **1d** was proposed as the participating intermediate, which was subsequently captured by the sodium sulfinate. Another method to create allylic sulfones involves the intermediacy of allyl cations.^9b,9c^ For example, Sreedhar reported that ionization of 2-cyclohexen-1-ol **2a** with catalytic FeCl_3_ in the presence of TMSCl produced cyclohexenyl sulfone **2b** in 65% yield. The reaction mechanism was proposed to proceed via allylic cation **2c**.^9c^

**Scheme 1:**
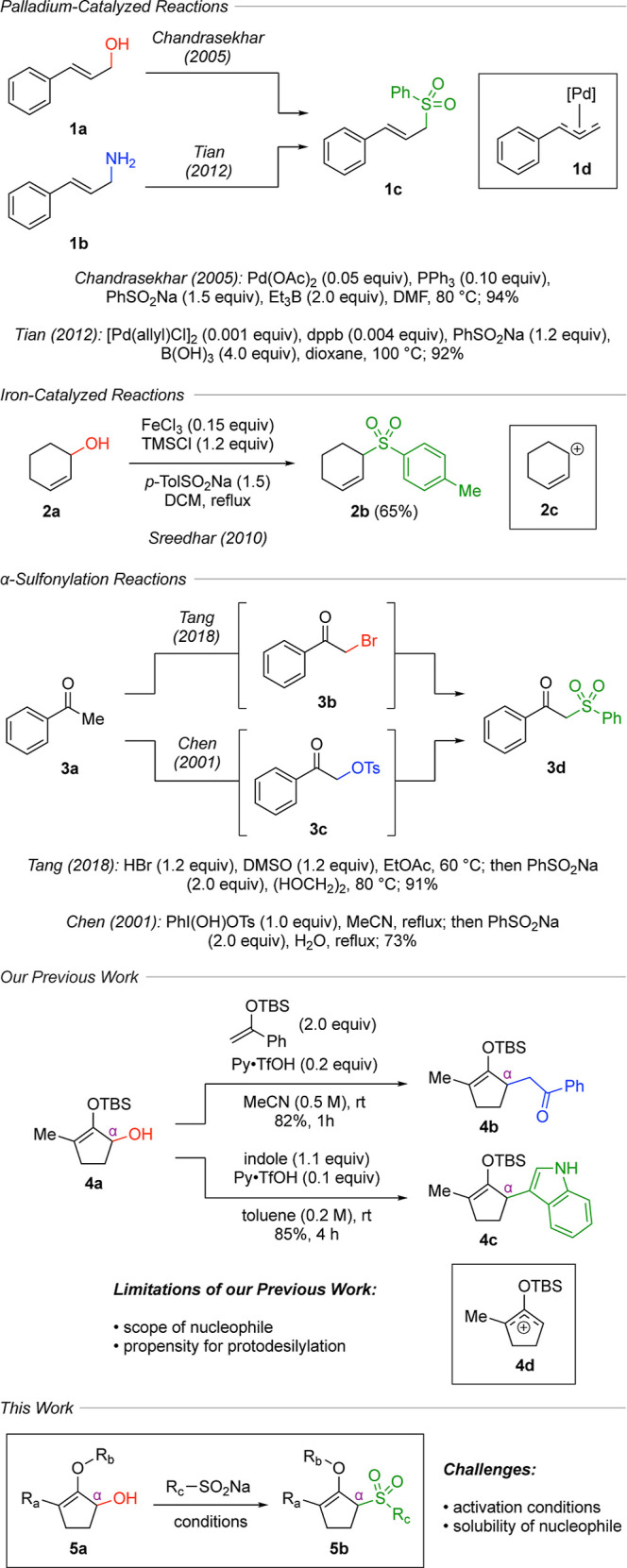
Sodium Sulfinate Salts in Nucleophilic Substitution Reactions

A related structural motif to an allylic system is the carbonyl compound, as sodium sulfinate salts have proven effective in displacing a leaving group at the α-position.^11^ For example, Tang installed an α-bromo group to acetophenone in **3b**, which could then be substituted by sodium sulfinate salt to form α-sulfonyl ketone **3d**.^11a^ Similar chemistry was reported by Chen, who employed a tosyl group in **3c**.^11b^

Recently, our group reported synthetic reactions that feature regioselective nucleophilic substitution of protected oxyallyl cations **4d**. By incorporating a silyloxy group at the central carbon, our chemistries effectively leveraged the reactivity of the allylic cation moiety toward carbon–carbon bond formation to create uniquely functionalized carbonyl-derived compounds at the α-position.^12^ For example, ionization of α-hydroxy silyloxy enol ethers **4a** under catalytic pyridinium triflate in the presence of silylenolate produced 1,4-dicarbonyl motif **4b**, which was readily differentiated as a monosilylenolate in 82% yield.^12e^ The use of indole as a nucleophile was also successful, resulting in the forging of sp^2^–sp^3^ carbon–carbon connectivity at the α-position of cyclopentanone-derived product **4c**.^12f^ Remarkably, these reactions exhibited excellent control of regioselectivity.

Despite this versatility, our chemistries had limitations. So far, only certain π-carbon nucleophiles (silylenolates, indoles, and related compounds) were applicable. The use of ionic nucleophiles,^13^ such as sodium sulfinate salts, has not been feasible because they are insoluble under the reaction conditions. Since sodium sulfinates tend to be more soluble in protic solvents, we recognize that such conditions could cause protodesilylation, leading to the decomposition of the α-hydroxy silyloxy enol ether moiety, especially in the presence of Brønsted acid catalysts. In this communication, our studies aim to overcome these challenges by reimagining this chemistry to enable regioselective nucleophilic addition of sodium sulfinates to protected oxyallyl cations.

## Results and Discussion

Our pilot studies are depicted in Scheme 2. To this end, we judiciously designed α-hydroxy enol ether **7** as a model substrate, featuring a *para*-methoxyphenyl (PMP) group not only to enhance the stability of the enol ether functionality but also to allow easy monitoring of reaction progress by TLC under UV light. The PMP group also presented a remotely positioned methoxy signal in the ^1^H NMR, which aided regioselectivity analysis of the crude reaction mixtures. Compound **7** was conveniently prepared from substituted cyclopentenone **6a** in simple three steps, involving epoxidation, nucleophilic epoxide-ring opening, and elimination in the presence of *p*-methoxyphenol and K_2_CO_3_, followed by reduction of the resulting ketone with DIBAL to install the hydroxy group.

**Scheme 2:**
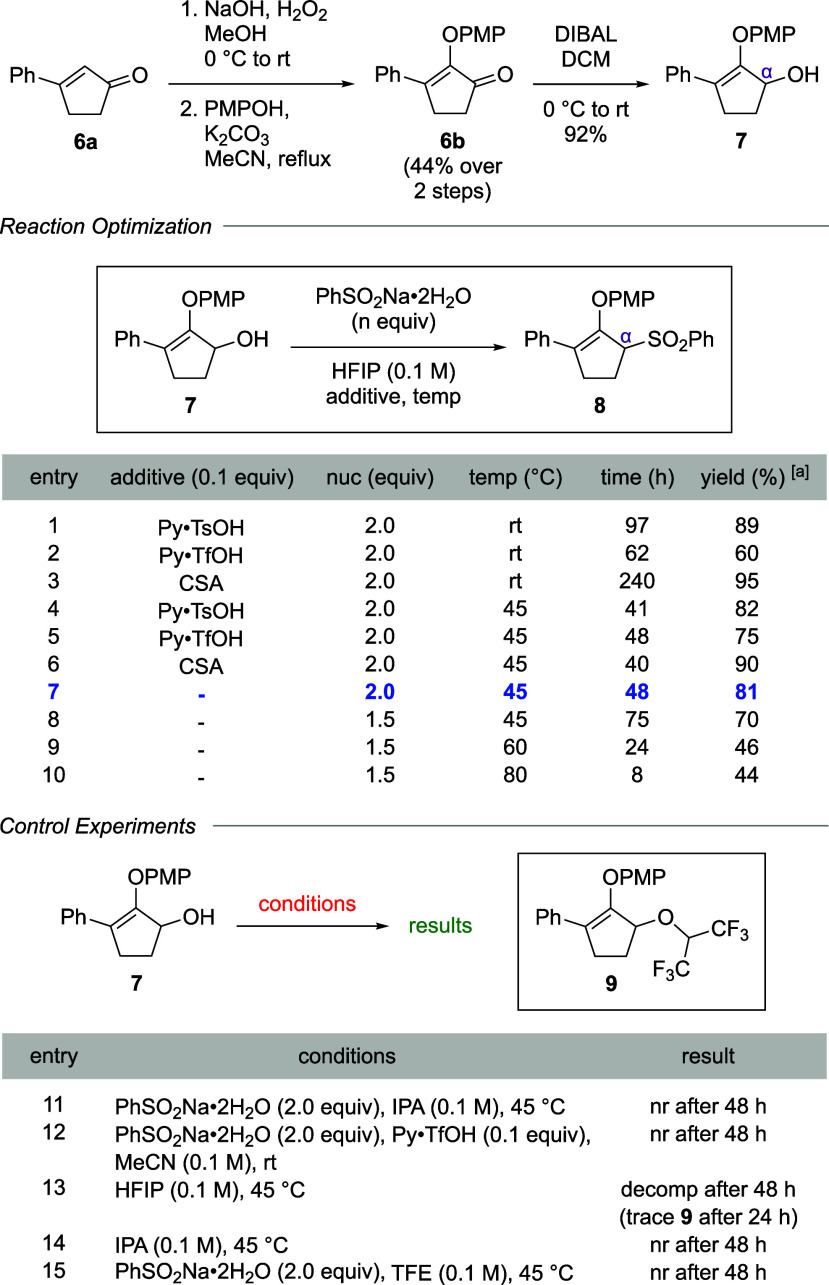
Preliminary Studies

As shown in entries 1–3, we initially exposed α-hydroxy enol ether **7** to 2 equiv of sodium benzenesulfinate dihydrate and catalytic Brønsted acid in 1,1,1,3,3,3-hexafluoroisopropanol (HFIP) at room temperature. HFIP is a highly versatile solvent with numerous applications in organic synthesis.^14^ In our case, HFIP was selected due to its ability to solubilize the sodium benzenesulfinate salt. Several common Brønsted acids were evaluated, including pyridinium tosylate (Py•TsOH), pyridinium triflate (Py•TfOH), and *rac*-camphorsulfonic acid (CSA). Under these conditions, α-sulfonyl enol ether **8** was obtained in excellent yields, reaching up to 95% as a single regioisomer. A drawback observed in these preliminary experiments was the notably prolonged reaction times. To expedite product formation, we raised the reaction temperature to 45 °C (entries 4–6), which cut the reaction time without substantially affecting the product yields (75–90%). Entry 7 indicated the background reaction in the absence of Brønsted acid. We were cognizant that, because HFIP is mildly acidic (p*K*_a_ 9.3), α-hydroxy enol ether **7** could potentially be ionized by the solvent. Indeed, these catalyst-free conditions yielded α-sulfonyl enol ether **8** in 81% yield. An attempt to further optimize this reaction by reducing the molar quantity of sodium benzenesulfinate salt to 1.5 equiv resulted in a reduction in yield to 70% (entry 8). Additionally, elevating the reaction temperature to 60 or 80 °C significantly eroded the yields due to decomposition (entries 9–10).

Control experiments were conducted to confirm the significance of HFIP as an activating solvent. As illustrated in entry 11, no product formation was observed when the reaction was performed in isopropanol (IPA), which has a p*K*_a_ of 17.1. The efficacy of acetonitrile (MeCN) was also assessed (entry 12). Despite the presence of pyridinium triflate catalyst, the reaction did not proceed to yield α-sulfonyl enol ether **8**. Under these two conditions, substrate **7** remained unreacted, and sodium benzenesulfinate salt appeared to be insoluble. Liu reported that ionization of allylic alcohols in HFIP formed allylic cation intermediates, which HFIP could capture to produce isolable HFIP ether adducts.^9a^ To determine whether this mechanism was operative in our reaction, we exposed α-hydroxy enol ether **7** to HFIP at 45 °C. Notably, isolation of HFIP ether adduct **9** was unsuccessful (entry 13). After 24 h, only a trace amount of this compound was detected via GC–MS. After 48 h, substrate **7** was entirely decomposed. Based on this result, intermediate **9**, if formed, is likely unstable under the reaction conditions. The propensity of α-hydroxy enol ether **7** to ionize in HFIP was further supported by its lack of reactivity in IPA or TFE in the presence of sodium benzenesulfinate at 45 °C (entries 14 and 15), highlighting the importance of the acidity of HFIP.

Having established that the addition of sodium benzenesulfinate salt to α-hydroxy enol ether **7** could be achieved in HFIP without catalysts, we were positioned to evaluate the scope of reaction (Scheme 3). First, this chemistry was amenable to scale-up. In a one-gram synthesis, α-sulfonyl enol ether **8** was isolated in 77% yield. Although the reaction optimization studies were conducted using the dihydrate salt, an attempt to employ anhydrous sodium benzenesulfinate as a nucleophile was also successful, yielding product **8** in 67% yield, albeit with a longer reaction time (see Supporting Information).^12c,12f^ Next, we varied three critical positions: the R_a_ substituent of the α-hydroxy enol ether substrate motif, the R_b_ substituent at the *O*-enol ether position, and the R_c_ substituent of the sodium sulfinate salt. Commencing with the R_a_ substituent, replacing the phenyl group with *p*-fluorophenyl afforded **12a** in 80% yield. Unsurprisingly, strongly electron-withdrawing 3,5-(CF_3_)_2_ substituent substantially eroded reactivity to form **12b** in 20% yield. α-Sulfonyl enol ethers **12c** to **12f** indicated compatibility with aliphatic substituents, such as methyl, octyl, phenethyl, and cyclohexyl, which were isolated in 65–76% yields.

**Scheme 3:**
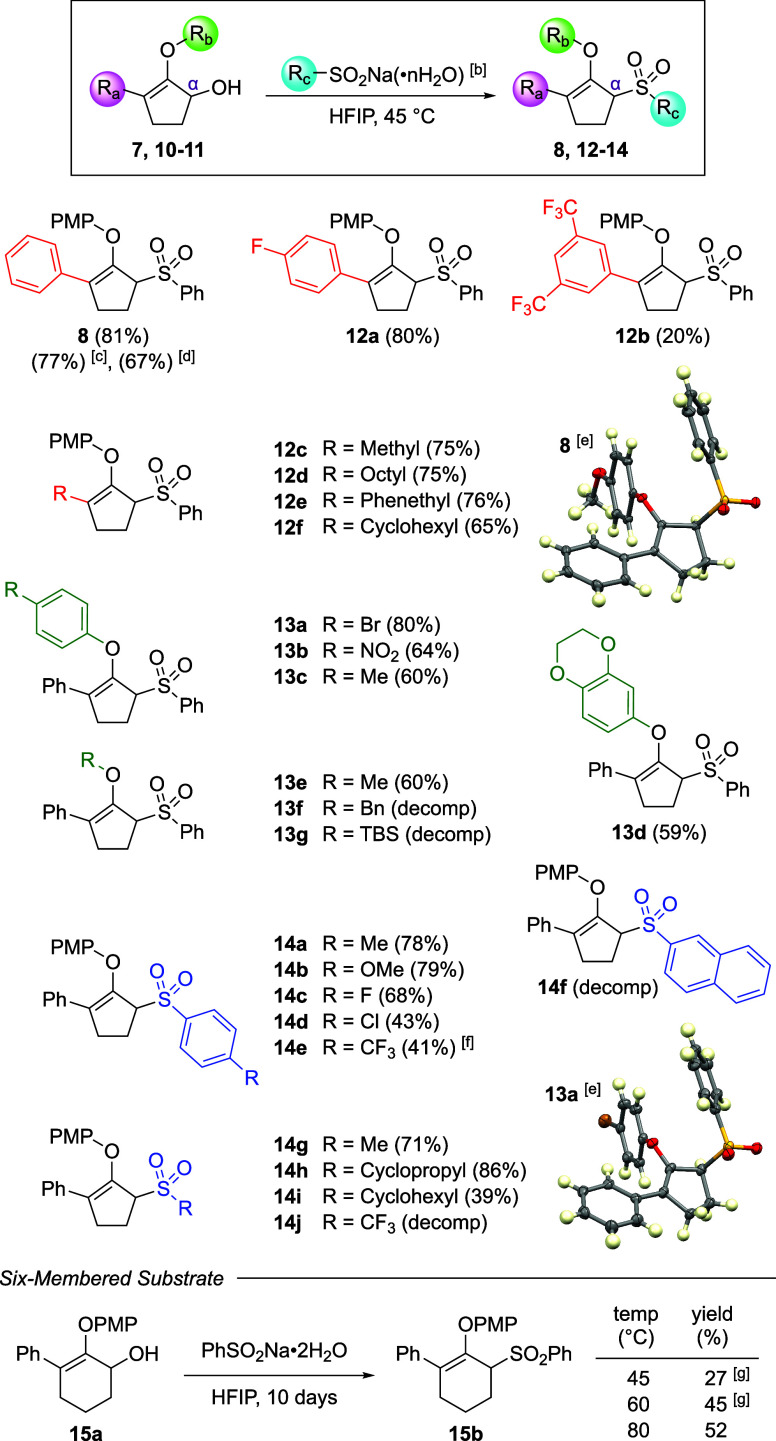
Scope of Reactions^a^

a Isolated yield after column chromatography.
b The hydration level of each sodium sulfinate salt is provided in the Supporting Information.
c Isolated yield for a 1 g synthesis.
d Isolated yield using anhydrous sodium benzenesulfinate.
e The ellipsoid contour was set at a 50% probability level.
f The reaction was performed at room temperature.
g Starting material was not fully consumed after 10 days.

We then examined the R_b_ substituent by introducing various *para*-substituted phenyl groups at the *O*-enol ether moiety, including bromo, nitro, and methyl, which furnished α-sulfonyl adducts **13a** to **13c** in 80%, 64%, and 60% yields, respectively. The 1,4-benzodioxane was also tolerated, yielding **13d** in 59%. Substrates containing nonarene groups at the *O*-enol ether position proved to be less reliable. For example, while α-sulfonyl *O*-methyl enol ether **13e** was successfully isolated in 60% yield, the *O*-benzyl and *O*-TBS versions did not survive the reaction conditions and resulted in decomposition. The commercial availability of sodium sulfinate salts allowed us to subject a variety of nucleophiles, starting with substituted benzenesulfinate at the *para*-position. For instance, electron-donating methyl and methoxy groups produced **14a** and **14b** in high yields. Halogen-containing nucleophiles were found to be compatible, as the reactions generated the fluoro- and chloro-containing α-sulfonyl adducts **14c** and **14d** in 68% and 43%, respectively. The CF_3_ group, interestingly, yielded product **14e** in 41% despite its electron-deactivating nature. Unexpectedly, naphthylsulfinate salt did not lead to nucleophilic addition, causing the substrate to decompose. Finally, alkyl nucleophiles, such as methyl, cyclopropyl, and cyclohexyl, produced α-sulfonyl enol ethers **14g** to **14i** in 71%, 86%, and 39% yields, respectively. The significantly lower yield observed for cyclohexyl-containing adduct **14i** suggested that steric effects influence reaction efficiency. Lastly, poorly nucleophilic trifluoromethylsulfinate salt did not react, resulting in substrate decomposition.

The applicability of 6-membered α-hydroxy enol ether **15a** to the reaction conditions was also examined. This substrate was found to be more challenging to activate than the 5-membered version.^12c^ In fact, the resulting α-sulfonyl enol ether **15b** was isolated in only 27% yield under the optimized conditions. Increasing the reaction temperature to 60 °C improved the yield to 45%. In both cases, we observed that substrate **15a** was not fully consumed even after 10 days of reaction time. An attempt to raise the temperature further to 80 °C slightly increased the yield to 52% after 10 days, at which the starting material was no longer detected in the reaction mixture. All products shown in Scheme 3 were produced with >20:1 regioselectivity, where nucleophilic addition of the sulfonyl group occurred at the α-position opposite the R_a_ substituent. This regioselectivity was unambiguously confirmed through X-ray crystallography analysis of α-sulfonyl enol ethers **8** and **13a**.^15^

Scheme 4 underscores the versatile synthetic utility of α-sulfonyl enol ether adduct **8**, which can function as a convenient precursor for a wide range of intriguing synthetic transformations. In this context, several postfunctionalization reactions were executed to generate a library of structurally unique compounds. For instance, catalytic hydrogenation of the enol ether moiety with Pd/C and a hydrogen balloon yielded contiguous stereotriad **16a** in 58% yield with >20:1 diastereoselectivity. The enol ether functionality could also be subjected to oxidative cleavage with a mixture of RuCl_3_ and NaIO_4_, resulting in the formation of 1,5-ketoester **16b**, which featured a sulfone at the α-position of the ester, in 91% yield. Subsequently, this compound was subjected to an α-alkylation reaction in the presence of allyl bromide, Cs_2_CO_3_, and catalytic KI in acetone at room temperature. These mild conditions facilitated the formation of highly functionalized 1,5-ketoester **16c**, which was isolated in 57% yield.

**Scheme 4:**
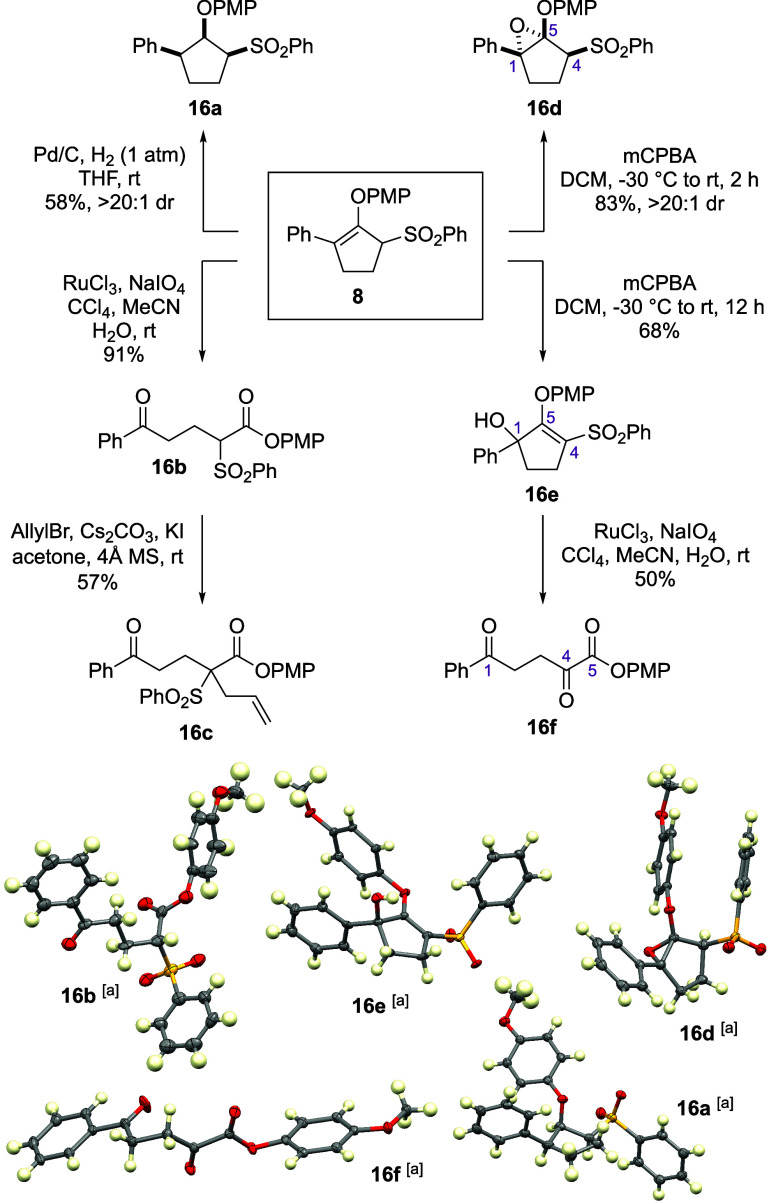
Post-Functionalization^a^

a The ellipsoid contour was set at a 50% probability level.

Oxidation of the enol ether double bond could also be carried out using mCPBA, and we observed remarkable time-dependent selectivity. Specifically, stopping the reaction after 2 h yielded isolable stereochemically complex epoxide **16d** in 83% yield as a single diastereomer. However, extending the reaction time to 12 h resulted in a different product, namely α-hydroxy enol ether **16e**, in 68% yield. This finding suggested that under prolonged exposure to the reaction conditions, epoxide **16d** underwent deprotonation at the α-sulfonyl C4 position, leading to ring-opening of the epoxide. Compound **16e** itself is a unique synthetic intermediate. For instance, an attempt to oxidatively cleave the enol ether using a mixture of RuCl_3_ and NaIO_4_ surprisingly yielded 1,4,5-diketoester **16f**, in which oxidation of the C4 position led to elimination of the sulfonyl group. We were able to unambiguously determine the structures of compounds **16a**, **16b**, **16d**, **16e**, and **16f**, including their relative stereochemistry where present, by X-ray crystallography.^15^

## Conclusions

In conclusion, this work demonstrates that HFIP is an effective solvent for the ionization of cyclic α-hydroxy enol ethers to generate *O*-aryl-protected oxyallyl cations, leveraging its mild acidity and eliminating the need for an external Brønsted acid catalyst. The solubility of sodium sulfinates in HFIP enables their use as nucleophiles to capture the cationic intermediate in a regioselective manner. The resulting α-sulfonyl enol ether adducts are synthetically versatile and readily transformed into diverse molecular architectures. This approach overcomes key limitations of our previous studies on silyloxyallyl cations, which were ineffective for sulfonylation with sodium sulfinates under previously reported Brønsted acid-catalyzed conditions due to poor salt solubility. Although HFIP improves sulfinate solubility, silyloxyallyl cations appear unstable in this acidic medium. In contrast, incorporation of an *O*-aryl group enhances stability of the oxyallyl cation intermediate, enabling productive reactivity under HFIP conditions with soluble sodium sulfinate salts.

## Experimental Sections

Experimental procedures and characterization data are detailed in the Supporting Information.

WARNING: Appropriate safety precautions should be exercised when handling hazardous chemicals and conducting the reactions described in this work.

## Supplementary Material



## Data Availability

The data underlying this study are available in the published article and its Supporting Information.

## Notes

Experimental procedures, characterization data, X-ray crystallographic data, and ^1^H and ^13^NMR spectra for all new compounds (PDF)
