# Supplementary material for: HFIP-Induced Formation of O‑Aryl Oxyallyl Cation and Nucleophilic Addition with Sodium Sulfinate Salt
Source: J Org Chem. 2026 May 26;91(22):7436–42. doi: 10.1021/acs.joc.6c00351 (PMC13247966; doi:10.1021/acs.joc.6c00351)
Supplement: Supplementary file 1 [file jo6c00351_si_001.pdf]

HFIP-INDUCED FORMATION OF *O*-ARYL OXYALLYL CATION AND NUCLEOPHILIC ADDITION WITH SODIUM SULFINATE SALT

Jhonny Aldás-Bedón,[‡] Adenike O. Adebanji,[‡] Raju Thombal, Estefania Armendariz-Gonzalez, Edward W. Mureka, Frank R. Fronczek, and Rendy Kartika*

‡ Equal Contribution

Department of Chemistry

232 Choppin Hall

Louisiana State University

Baton Rouge, LA 70803, USA

email: rkartika@lsu.edu

SUPPORTING INFORMATION

Table of Contents

1. General Information……………………………………………………………... SI-3

2. Experimental Data for Schemes 3 ……………………….……………………………… SI-4

3. Experimental Data for Schemes 4 ……………………….……………………………… SI-24

4. Synthesis of Substrates …….…………………………………………………………… SI-32

5. X-Ray Crystallography Data ………….……………………………………………… SI-55

6. ^{1}H And ^{13}C NMR Spectra ………………….…….……….…….…….………………… SI-120

General Information

Unless otherwise noted, all materials were used as received from commercial suppliers without further purification. All anhydrous reactions were performed using oven-dried glassware, which was then cooled under vacuum and purged with nitrogen gas. Tetrahydrofuran (THF), dichloromethane (CH_2Cl_2), acetonitrile, toluene, diethyl ether (Et_2O), dimethylformamide (DMF), and *n*-hexanes were filtered through activated silica or 3Å molecular sieves under argon contained in a solvent purification system. All reactions were monitored by analytical thin layer chromatography (TLC silica gel 60 F254, Glass Plates) and analyzed with 254 nm UV light and / or anisaldehyde – sulfuric acid or potassium permanganate treatment. Column chromatography was completed using silica gel (32-63 μ).

Unless otherwise noted, all ^{1}H and ^{13}C NMR spectra were recorded in $CDCl_3$ using a Bruker Ascend 400 spectrometer operating at 400 MHz for ^{1}H and 100 MHz for ^{13}C or Bruker Ascend 500 spectrometer operating at 500 MHz for ^{1}H and 125 MHz for ^{13}C. Chemical shifts (δ) are reported in ppm relative to residual $CHCl_3$ as an internal reference (^{1}H: 7.26 ppm, ^{13}C: 77.00 ppm). Coupling constants (*J*) are reported in Hertz (Hz). Peak multiplicity is indicated as follows: s (singlet), d (doublet), t (triplet), q (quartet), p (pentet), x (sextet), h (heptet), b (broad), and m (multiplet). FT-IR spectra were recorded on Bruker Tensor 27 spectrometer and OPUS 6.5 Data Collection Program, and absorption frequencies were reported in reciprocal centimeters (cm^{-1}). High Resolution Mass Spectrometry (HRMS) measurements using a time-of-flight (TOF) mass analyzer were performed at the Louisiana State University Mass Spectrometry Facility. X-ray structure analyses were performed by the Louisiana State University X-ray Structure Facility.

Experimental Data for Scheme 3

General Procedure:

α-Hydroxy enol ether starting material and sodium sulfinate salt were introduced into a heavy-wall cylindrical pressure flask. 1,1,1,3,3,3-Hexafluoroisopropanol (HFIP) was then added. The reaction mixture was stirred at 45 °C in an oil bath until the starting material was fully consumed, as monitored by TLC. After cooling to room temperature, the reaction mixture was diluted with DI water (15 mL). The aqueous layer was then extracted with CH_2Cl_2 (3 x 15 mL). The combined organic layers were dried over Na_2SO_4 and then concentrated under vacuum. The crude material was purified with column chromatography to yield α-sulfonyl enol ether product.

Compound 8

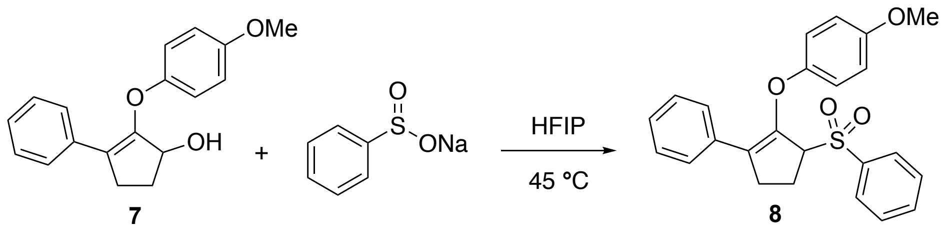

Quantity: α-hydroxy enol ether starting material **7** (213 mg, 0.756 mmol); sodium benzenesulfinate dihydrate (303 mg, 1.51 mmol); HFIP (7.6 mL); α-sulfonyl enol ether product **8** (249 mg, 0.613 mmol, 81% yield, yellow solid).

Reaction Time: 48 h

Rf: 0.4 in CH_2Cl_2

Column Chromatography: 90:10 hexanes : ethyl acetate

^{1}**H NMR:** (400 MHz, $CDCl_3$) δ = 7.84 – 7.81 (m, 2H), 7.54 (m, 1H), 7.44 – 7.37 (m, 4H) 7.24 – 7.14 (m, 3H), 6.68 (ddd, J = 10.4, 3.2, 3.0 Hz, 2H), 6.59 (ddd, J = 10.3, 3.1, 2.9 Hz, 2H), 4.23 (m, 1H), 3.68 (s, 3H), 2.59 – 2.52 (m, 2H), 2.42 (m, 1H), 2.29 (m, 1H).

^{13}**C{1H} NMR:** (100 MHz, $CDCl_3$) δ = 155.6, 148.7, 142.3, 138.0, 133.6, 133.5, 130.8, 129.3, 128.8, 128.3, 128.0, 127.1, 117.9, 114.7, 69.0, 55.6, 29.5, 22.7.

IR: f (cm^{-1}) = 3068, 3000, 2933, 2831, 1505, 1449, 1316, 1244, 1189, 810.

HRMS (ESI-TOF): *m/z* $[M+Na]^+$ = 429.1136 calculated for $C_{24}H_{22}NaO_4S$; found 429.1142.

X-Ray Crystallography:

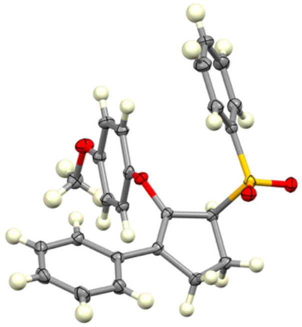

One-Gram Synthesis

Quantity: α-hydroxy enol ether starting material **7** (1.007 mg, 3.54 mmol); sodium benzenesulfinate dihydrate (1.421 g, 7.08 mmol); HFIP (35 mL); α-sulfonyl enol ether product **8** (1.106 g, 2.73 mmol, 77%, yellow solid).

Reaction Time: 47 h

Column Chromatography: 80:20 hexanes : ethyl acetate

Using Anhydrous Benzenesulfinate Salt

Quantity: α-hydroxy enol ether starting material **7** (50 mg, 0.177 mmol); anhydrous sodium benzenesulfinate (58 mg, 0.354 mmol); HFIP (1.8 mL); α-sulfonyl enol ether product **8** (48 mg, 0.117 mmol, 67%, yellow solid).

Reaction Time: 70 h

Column Chromatography: 80:20 hexanes : ethyl acetate

Compound 12a

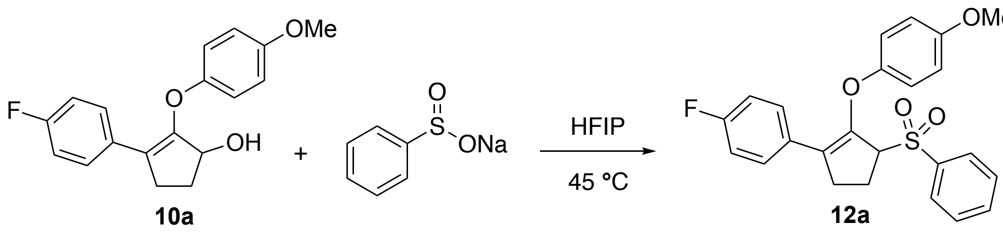

Quantity: α-hydroxy enol ether starting material **10a** (102 mg, 0.339 mmol); sodium benzenesulfinate dihydrate (136 mg, 0.679 mmol); HFIP (3.4 mL); α-sulfonyl enol ether product **12a** (116 mg, 0.273 mmol, 80% yield, yellow oil).

Reaction Time: 23 h

Rf: 0.6 in CH_2Cl_2

Column Chromatography: 80:20 hexanes : ethyl acetate

^{1}H NMR: (400 MHz, $CDCl_3$) δ = 7.91 – 7.88 (m, 2H), 7.63 (m, 1H), 7.53 – 7.44 (m, 4H), 7.00 – 6.94 (m, 2H), 6.77 – 6.73 (m, 2H), 6.67 – 6.63 (m, 2H), 4.30 (dt, *J* = 9.5, 1.8 Hz, 1H), 3.76 (s, 3H), 2.66 – 2.58 (m, 2H), 2.48 (dtd, *J* = 15.4, 8.0, 2.5 Hz, 1H), 2.36 (m, 1H).

^{13}C{1H} NMR: (100 MHz, $CDCl_3$) δ = 162.2 (d, $J_{C\text{-}F}$ = 248.4 Hz), 155.6, 148.5, 142.1, 138.0, 133.6, 129.6, 129.2, 128.94, 128.96, 128.8, 117.8, 115.1 (d, $J_{C\text{-}F}$ = 21.3 Hz), 114.7, 68.9, 55.6, 29.5, 22.6.

^{19}F NMR: (470 MHz, $CDCl_3$) δ = -112.7.

IR: *f* (cm^{-1}) = 3064, 3019, 2931, 1649, 1602, 1501, 1212, 1033, 831, 747.

HRMS (ESI-TOF): *m/z* $[M+Na]^+$ = 447.1042 calculated for $C_{24}H_{21}FNaO_4S$; found 447.1043.

Compound 12b

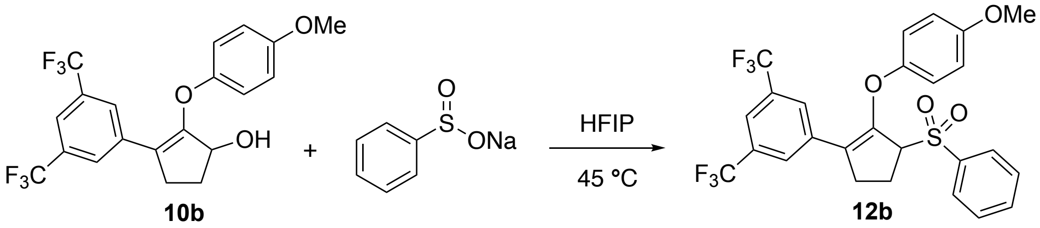

Quantity: α-hydroxy enol ether starting material **10b** (100 mg, 0.239 mmol); sodium benzenesulfinate dihydrate (96 mg, 0.48 mmol); HFIP (2.4 mL); α-sulfonyl enol ether product **12b** (26 mg, 0.05 mmol, 20% yield, yellow oil).

Reaction Time: 98 h

Rf: 0.6 in CH_2Cl_2

Column Chromatography: CH_2Cl_2

^{1}H NMR: (400 MHz, $CDCl_3$) δ = 7.93 (d, *J* = 1.72 Hz, 2H), 7.90 – 7.87 (m, 2H), 7.73 (s, 1H), 7.64 (m, 1H), 7.52 – 7.47 (m, 2H), 6.76 (ddd, *J* = 10.3, 3.0, 2.9 Hz, 2H), 6.67 (ddd, *J* = 10.4, 3.0, 2.9 Hz, 2H), 4.36 (dt, *J* = 9.3, 1.7 Hz, 1H), 3.76 (s, 3H), 2.72 – 2.55 (m, 3H), 2.40 (m, 1H).

^{13}C{1H} NMR: (125 MHz, $CDCl_3$) δ = 156.0, 148.1, 146.0, 137.8, 135.5, 133.9, 131.6 (q, J_{C-F} = 33.0 Hz), 129.1 (d, J_{C-F} = 13.8 Hz), 126.4, 126.9 (q, J_{C-F} = 3.9 Hz), 123.2 (q, J_{C-F} = 271.2 Hz), 121.2 (m), 118.2, 117.8, 114.8, 68.8, 55.6, 29.3, 22.7.

^{19}F NMR: (470 MHz, $CDCl_3$) δ = -63.0.

IR: *f* (cm^{-1}) = 3070, 2931, 2837, 1745, 1643, 1502, 1250, 1110, 1005, 790.

HRMS (ESI-TOF): *m/z* $[M+Na]^+$ = 565.0884 calculated for $C_{26}H_{20}F_6NaO_4S$; found 565.0889.

Compound 12c

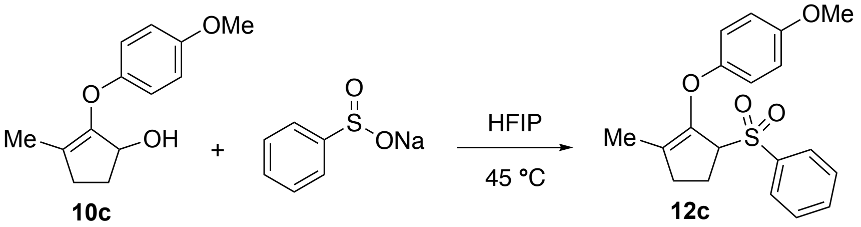

Quantity: α-hydroxy enol ether starting material **10c** (100 mg, 0.454 mmol); sodium benzenesulfinate dihydrate (182 mg, 0.908 mmol); HFIP (4.5 mL); α-sulfonyl enol ether product **12c** (117 mg, 0.339 mmol, 75% yield, colorless oil).

Reaction Time: 29 h

Rf: 0.4 in CH_2Cl_2

Column Chromatography: 80 : 20 hexanes : ethyl acetate

^{1}H NMR: (400 MHz, $CDCl_3$) δ = 7.89 – 7.86 (m, 2H), 7.62 (m, 1H), 7.51 – 7.47 (m, 2H), 6.73 (ddd, *J* = 10.4, 2.9, 2.8 Hz, 2H), 6.59 (ddd, *J* = 10.4, 2.9, 2.8 Hz, 2H), 4.14 (dp, *J* = 9.4, 1.7 Hz, 1H), 3.75 (s, 3H), 2.53 (m, 1H), 2.27 (m, 1H), 2.17 – 2.12 (m, 2H), 1.60 (q, *J* = 1.3 Hz, 3H).

^{13}C{1H} NMR: (100 MHz, $CDCl_3$) δ = 155.1, 149.7, 141.0, 138.3, 133.4, 131.2, 129.1, 128.7, 117.2, 114.5, 68.0, 55.6, 31.6, 23.0, 12.4.

IR: *f* (cm^{-1}) = 3060, 2914, 2904, 2845, 1692, 1498, 1210, 1055, 812.

HRMS (ESI-TOF): *m/z* $[M+Na]^+$ = 367.0980 calculated for $C_{19}H_{20}NaO_4S$; found 367.0963.

Compound 12d

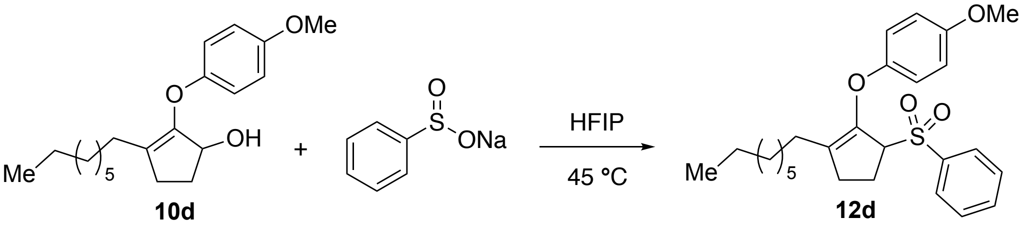

Quantity: α-hydroxy enol ether starting material **10d** (110 mg, 0.345 mmol); sodium benzenesulfinate dihydrate (138 mg, 0.690 mmol); HFIP (3.5 mL); α-sulfonyl enol ether product **12d** (115 mg, 0.260 mmol, 75% yield, pale yellow oil).

Reaction Time: 24 h

Rf: 0.4 in CH_2Cl_2

Column Chromatography: 90:10 hexanes : ethyl acetate

^{1}H NMR: (400 MHz, $CDCl_3$) δ = 7.89 – 7.87 (m, 2H), 7.62 (t, *J* = 7.4 Hz, 1H), 7.49 (t, *J* = 7.7 Hz, 2H), 6.75 – 6.70 (m, 2H), 6.60 – 6.56 (m, 2H), 4.14 (d, *J* = 9.3 Hz, 1H), 3.75 (s, 3H), 2.55 (m, 1H), 2.27 (m, 1H), 2.17 – 2.14 (m, 2H), 2.04 (t, *J* = 7.3 Hz, 2H), 1.30 – 1.21 (m, 12H), 0.87 (t, *J* = 6.9 Hz, 3H).

^{13}C{1H} NMR: (100 MHz, $CDCl_3$) δ = 155.1, 149.7, 140.6, 138.4, 135.9, 133.4, 129.2, 128.7, 117.2, 114.5, 68.0, 55.6, 31.8, 29.4 (2C), 29.3, 29.2, 27.1, 26.6, 23.2, 22.6, 14.1.

IR: *f* (cm^{-1}) = 3063, 3018, 2925, 2854, 2046, 1995, 1502, 1306, 1205, 828.

HRMS (ESI-TOF): *m/z* $[M+Na]^+$ = 465.2075 calculated for $C_{26}H_{34}NaO_4S$; found 465.2086.

Compound 12e

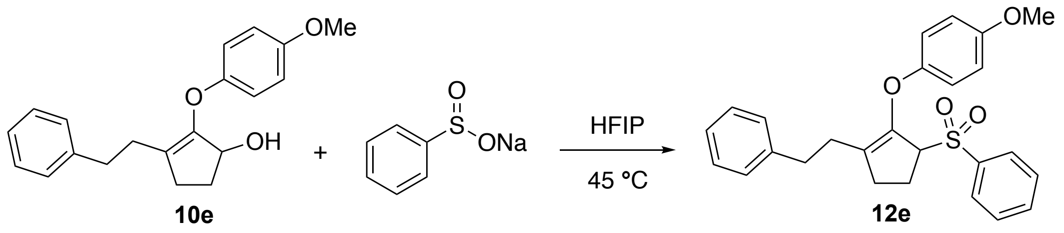

Quantity: α-hydroxy enol ether starting material **10e** (100 mg, 0.322 mmol); sodium benzenesulfinate dihydrate (129 mg, 0.644 mmol); HFIP (3.2 mL); α-sulfonyl enol ether product **12e** (106 mg, 0.244 mmol, 76% yield, yellow oil).

Reaction Time: 60 h

Rf: 0.5 in 80 : 20 hexanes : ethyl acetate

Column Chromatography: 80 : 20 hexanes : ethyl acetate

^{1}H NMR: (400 MHz, $CDCl_3$) δ = 7.88 – 7.85 (m, 2H), 7.63 (m, 1H), 7.52 – 7.48 (m, 2H), 7.24 – 7.22 (m, 2H), 7.17 (m, 1H), 7.12 – 7.09 (m, 2H), 6.68 (ddd, *J* = 10.6, 3.2, 3.1 Hz, 2H), 6.44 (ddd, *J* = 10.5, 3.1, 3.0 Hz, 2H), 4.09 (m, 1H), 3.75 (s, 3H), 2.67 – 2.51 (m, 3H), 2.45 (m, 1H), 2.33 (m, 1H), 2.27 – 2.10 (m, 3H).

^{13}C{1H} NMR: (100 MHz, $CDCl_3$) δ = 155.1, 149.5, 141.5, 141.3, 138.3, 134.3, 133.5, 129.2, 128.7, 128.4, 128.3, 126.0, 117.4, 114.5, 67.9, 55.6, 33.2, 29.4, 28.4, 23.2.

IR: *f* (cm^{-1}) = 3063, 2929, 2852, 1738, 1500, 1306, 1134, 815.

HRMS (ESI-TOF): *m/z* $[M+Na]^+$ = 457.1449 calculated for $C_{26}H_{26}NaO_4S$; found 457.1451.

Compound 12f

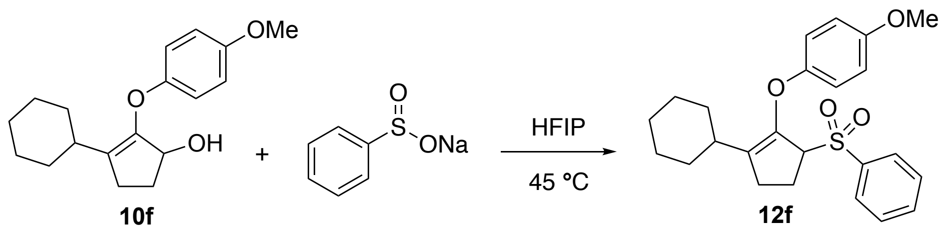

Quantity: α-hydroxy enol ether starting material **10f** (70 mg, 0.24 mmol); sodium benzenesulfinate dihydrate (97 mg, 0.49 mmol); HFIP (2.4 mL); α-sulfonyl enol ether product **12f** (65 mg, 0.16 mmol, 65% yield, yellow oil).

Reaction Time: 42 h

Rf: 0.4 in CH_2Cl_2

Column Chromatography: 90:10 hexanes : ethyl acetate

^{1}H NMR: (400 MHz, $CDCl_3$) δ = 7.91 (d, *J* = 7.6 Hz, 2H), 7.63 (t, *J* = 7.5 Hz, 1H), 7.50 (t, *J* = 7.7 Hz, 2H), 6.77 – 6.73 (m, 2H), 6.66 – 6.62 (m, 2H), 4.11 (dt, *J* = 9.4, 1.9 Hz, 1H), 3.75 (s, 3H),

2.50 (dd, J = 14.0, 8.6 Hz, 1H), 2.40 (ddd, J = 11.8, 8.4, 3.6 Hz, 1H), 2.28 – 2.12 (m, 2H), 2.00 (dtd, J = 15.8, 7.9, 2.8 Hz, 1H), 1.69 – 1.60 (m, 3H), 1.49 (d, J = 8.6 Hz, 1H), 1.40 (d, J = 12.3 Hz, 1H), 1.26 – 1.16 (m, 3H), 1.12 – 0.94 (m, 2H).

^{13}C{1H} NMR: (125 MHz, $CDCl_3$) δ = 155.1, 149.6, 140.4, 139.2, 138.1, 133.4, 129.3, 128.7, 117.3, 114.5, 68.0, 55.6, 35.8, 30.9, 30.2, 26.5, 26.04, 26.02, 25.9, 23.2.

IR: f (cm^{-1}) = 3019, 2927, 1675, 1587, 1502, 1207, 1036, 829, 745.

HRMS (ESI-TOF): m/z $[M+Na]^+$ = 435.1606 calculated for $C_{24}H_{28}NaO_4S$; found 435.1616.

Compound 13a

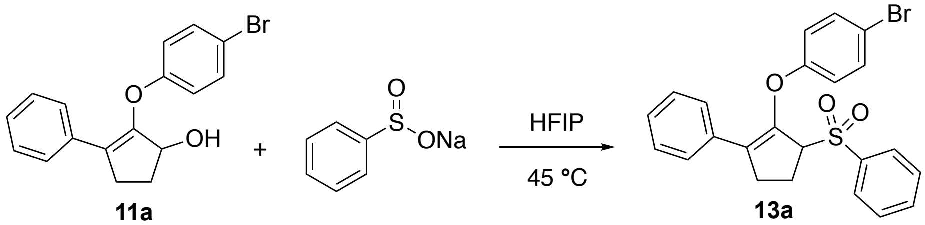

Quantity: α-hydroxy enol ether starting material **11a** (100 mg, 0.302 mmol); sodium benzenesulfinate dihydrate (121 mg, 0.604 mmol); HFIP (3.0 mL); α-sulfonyl enol ether product **13a** (110 mg, 0.241 mmol, 80% yield, white solid).

Reaction Time: 48 h

Rf: 0.6 in CH_2Cl_2

Column Chromatography: 70 : 30 hexanes : ethyl acetate

^{1}H NMR: (400 MHz, $CDCl_3$) δ = 7.91 – 7.88 (m, 2H), 7.62 (m, 1H), 7.50 – 7.42 (m, 4H), 7.34 – 7.25 (m, 5H), 6.62 (ddd, J = 10.1, 2.8, 2.7 Hz, 2H), 4.31 (dt, J = 9.4, 2.0 Hz, 1H), 2.71 – 2.63 (m, 2H), 2.57 (m, 1H), 2.40 (m, 1H).

^{13}C{1H} NMR: (100 MHz, $CDCl_3$) δ = 154.1, 141.0, 138.0, 133.7, 133.0, 132.5, 132.2, 129.2, 128.9, 128.4, 128.3, 127.1, 118.4, 115.7, 68.9, 29.5, 22.5.

IR: f (cm^{-1}) = 3066, 2927, 1694, 1588, 1486, 1210, 1022, 950.

HRMS (ESI-TOF): m/z $[M\text{-}SO_2Ph+H]^+$ = 313.0228 calculated for $C_{17}H_{14}BrO$; found 313.0233.

X-Ray Crystallography:

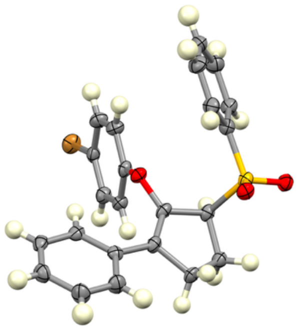

Compound 13b

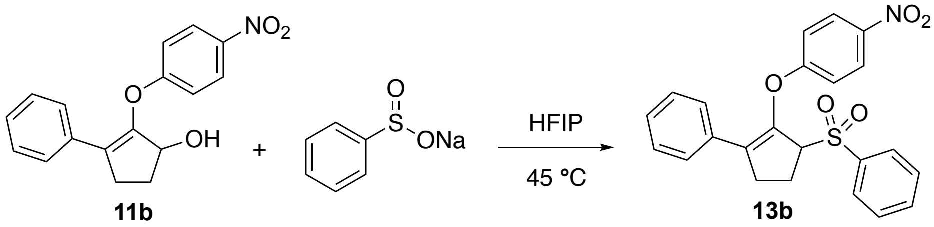

Quantity: α-hydroxy enol ether starting material **11b** (100 mg, 0.336 mmol); sodium benzenesulfinate dihydrate (135 mg, 0.672 mmol); HFIP (3.4 mL); α-sulfonyl enol ether product **13b** (90 mg, 0.34 mmol, 64% yield, colorless oil).

Reaction Time: 64 h

Rf: 0.4 in CH_2Cl_2

Column Chromatography: 80 : 20 hexanes : ethyl acetate

^{1}H NMR: (400 MHz, $CDCl_3$) δ = 8.13 (ddd, J = 10.4, 2.8, 2.7 Hz, 2H), 7.91 – 7.89 (m, 2H), 7.64 (m, 1H), 7.49 (t, J = 7.7 Hz, 2H), 7.40 – 7.37 (m, 2H), 7.31 – 7.26 (m, 3H), 6.86 (ddd, J = 10.4, 3.0, 2.8 Hz, 2H), 4.35 (m, 1H), 2.78 – 2.60 (m, 3H), 2.48 (m, 1H).

^{13}C{1H} NMR: (125 MHz, $CDCl_3$) δ = 159.9, 143.2, 139.8, 137.8, 133.9, 133.7, 132.5, 129.1, 129.0, 128.8, 128.5, 127.2, 125.9, 116.5, 69.1, 29.7, 22.4.

IR: *f* (cm^{-1}) = 3075, 2917, 2850, 1589, 1516, 1487, 1344, 1210, 1082.

HRMS (ESI-TOF): *m/z* $[M\text{-}SO_2Ph+H]^+$ = 280.0974 calculated for $C_{17}H_{14}NO_3$; found 280.0978.

Compound 13c

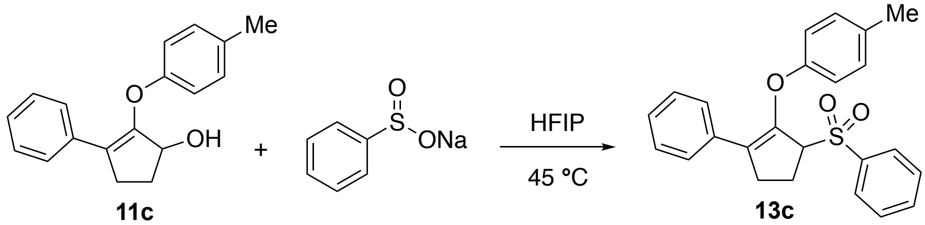

11c **13c**

Quantity: α-hydroxy enol ether starting material **11c** (102 mg, 0.384 mmol); sodium benzenesulfinate dihydrate (154 mg, 0.769 mmol); HFIP (3.8 mL); α-sulfonyl enol ether product **13c** (90 mg, 0.23 mmol, 60% yield, pale yellow solid).

Reaction Time: 66 h

Rf: 0.5 in CH_2Cl_2

Column Chromatography: 90:10 hexanes : ethyl acetate

^{1}H NMR: (400 MHz, $CDCl_3$) δ = 7.82 – 7.80 (m, 2H), 7.52 (tt, *J* = 7.1, 1.3 Hz, 1H), 7.44 – 7.34 (m, 4H), 7.21 – 7.12 (m, 3H), 6.94 – 6.91 (m, 2H), 6.54 – 6.50 (m, 2H), 4.25 (dt, *J* = 9.5, 1.9 Hz, 1H), 2.60 – 2.52 (m, 2H), 2.45 (dtd, *J* = 15.5, 8.1, 2.5 Hz, 1H), 2.30 (m, 1H), 2.19 (s, 3H).

^{13}C{1H} NMR: (100 MHz, $CDCl_3$) δ = 152.7, 141.9, 138.0, 133.6, 133.4, 132.6, 131.2, 130.0, 129.2, 128.8, 128.2, 128.0, 127.1, 116.5, 69.0, 29.5, 22.6, 20.5.

IR: *f* (cm^{-1}) = 3059, 3028, 2923, 1648, 1607, 1504, 1220, 1136, 819, 759.

HRMS (ESI-TOF): *m/z* $[M+Na]^+$ = 413.1187 calculated for $C_{24}H_{22}NaO_3S$; found 413.1189.

Compound 13d

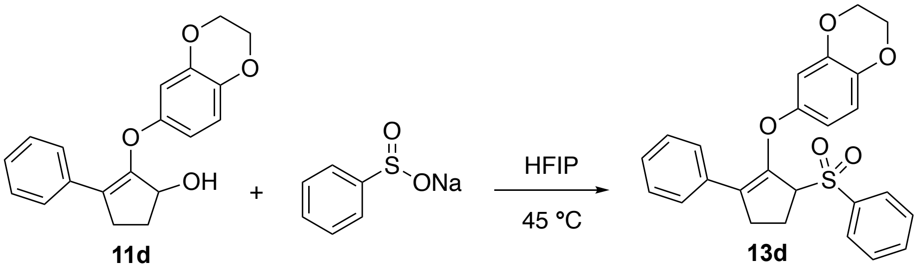

Quantity: α-hydroxy enol ether starting material **11d** (109 mg, 0.351 mmol); sodium benzenesulfinate dihydrate (140 mg, 0.702 mmol); HFIP (3.5 mL); α-sulfonyl enol ether product **13d** (90 mg, 0.21 mmol, 59% yield, pale yellow solid).

Reaction Time: 85 h

Rf: 0.5 in 99:1 CH_2Cl_2 : MeOH

Column Chromatography: CH_2Cl_2

^{1}H NMR: (400 MHz, $CDCl_3$) δ = 7.83 – 7.81 (m, 2H), 7.54 (m, 1H), 7.41 – 7.37 (m, 4H), 7.22 – 7.13 (m, 3H), 6.61 (d, J = 8.7 Hz, 1H), 6.17 – 6.11 (m, 2H), 4.26 (dt, J = 9.5, 1.8 Hz, 1H), 4.13 – 4.10 (m, 4H), 2.56 (dt, J = 15.8, 7.7 Hz, 2H), 2.44 (dtd, J = 15.5, 8.0, 2.5 Hz, 1H), 2.30 (m, 1H).

^{13}C{1H} NMR: (100 MHz, $CDCl_3$) δ = 149.0, 143.8, 142.0, 139.5, 138.0, 133.6, 133.4, 131.2, 129.2, 128.8, 128.2, 128.0, 127.1, 117.4, 109.8, 105.8, 69.0, 64.4, 64.0, 29.4, 22.6.

IR: f (cm^{-1}) = 3024, 2933, 2876, 1649, 1599, 1497, 1214, 1141, 1065, 745.

HRMS (ESI-TOF): m/z $[M+Na]^+$ = 457.1086 calculated for $C_{25}H_{22}NaO_5S$; found 457.1088.

Compound 13e

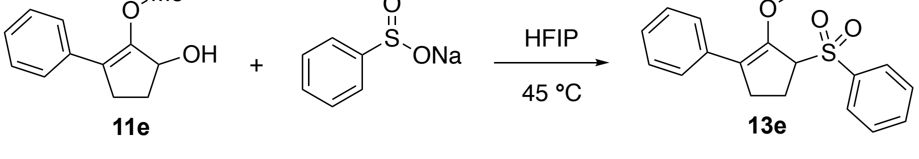

Quantity: α-hydroxy enol ether starting material **11e** (100 mg, 0.526 mmol); sodium benzenesulfinate dihydrate (212 mg, 1.05 mmol); HFIP (5.4 mL); α-sulfonyl enol ether product **13e** (100 mg, 0.317 mmol, 60% yield, yellow oil).

Reaction Time: 7 h

Rf: 0.5 in CH_2Cl_2

Column Chromatography: 80 : 20 hexanes : ethyl acetate

^{1}H NMR: (400 MHz, $CDCl_3$) δ = 7.95 – 7.92 (m, 2H), 7.58 (m, 1H), 7.47 – 7.41 (m, 4H), 7.31 – 7.27 (m, 2H), 7.20 (m, 1H), 4.52 (dt, J = 9.3, 2.3 Hz, 1H), 3.74 (s, 3H), 2.46 –2.37 (m, 2H), 2.31 (m, 1H), 1.91 (m, 1H).

^{13}C{1H} NMR: (100 MHz, $CDCl_3$) δ = 146.5, 136.9, 134.4, 133.8, 129.3, 128.7, 128.0, 127.2, 127.1, 124.1, 69.2, 57.7, 29.0, 22.9.

IR: *f* (cm^{-1}) = 3057, 2937, 2855, 1735, 1635, 1447, 1311, 1227, 1133, 962.

HRMS (ESI-TOF): *m/z* $[M+Na]^+$ = 337.0874 calculated for $C_{18}H_{18}NaO_3S$; found 337.0883.

Compound 14a

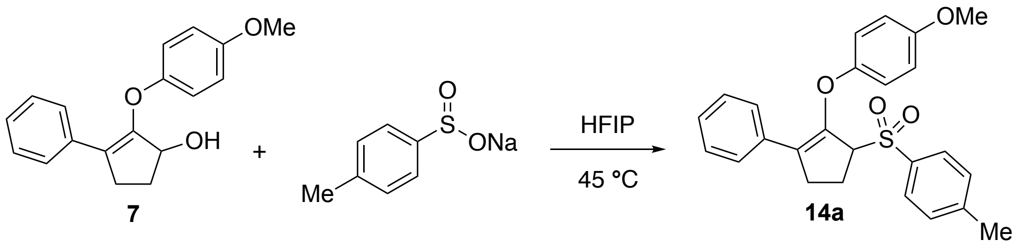

7

14a

Quantity: α-hydroxy enol ether starting material **7** (101 mg, 0.357 mmol); sodium 4-methylbenzenesulfinate (127 mg, 0.715 mmol); HFIP (3.6 mL); α-sulfonyl enol ether product **14a** (117 mg, 0.278 mmol, 78% yield, yellow solid).

Reaction Time: 46 h

Rf: 0.6 in CH_2Cl_2

Column Chromatography: 80 : 20 hexanes : ethyl acetate

^{1}H NMR: (400 MHz, $CDCl_3$) δ = 7.76 (ddd, *J* = 8.7, 1.9, 1.8 Hz, 2H), 7.52 – 7.48 (m, 2H), 7.30 – 7.21 (m, 5H), 6.80 (ddd, *J* = 10.2, 2.9, 2.8 Hz, 2H), 6.70 (ddd, *J* = 10.1, 2.8, 2.7 Hz, 2H), 4.28 (dt, *J* = 9.4, 2.0 Hz, 1H), 3.75 (s, 3H), 2.65 – 2.56 (m, 2H), 2.48 (dtd, *J* = 15.0, 7.3, 2.3 Hz, 1H), 2.42 (s, 3H), 2.34 (m, 1H).

^{13}C{1H} NMR: (100 MHz, $CDCl_3$) δ = 155.6, 148.8, 144.6, 142.5, 135.1, 133.6, 130.7, 129.4, 129.3, 128.3, 128.0, 127.1, 118.0, 114.7, 69.0, 55.7, 29.5, 22.8, 21.6.

IR: *f* (cm^{-1}) = 3062, 2998, 2933, 2843, 1651, 1506, 1297, 1246, 820.

HRMS (ESI-TOF): *m/z* [M+Na]$^+$ = 443.1293 calculated for $C_{25}H_{24}NaO_4S$; found 443.1293.

Compound 14b

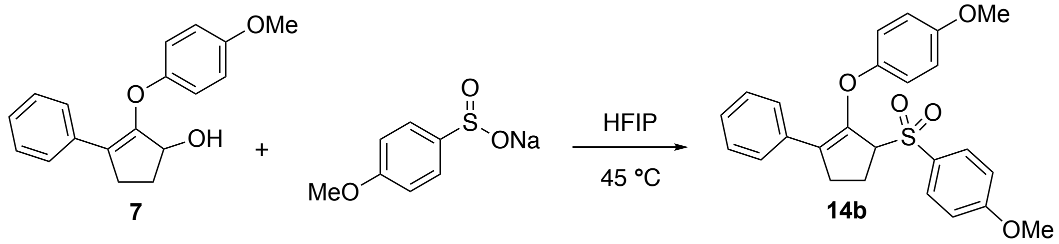

Quantity: α-hydroxy enol ether starting material **7** (100 mg, 0.354 mmol); sodium-4-methoxybenzene-1-sulfinate (138 mg, 0.708 mmol); HFIP (3.5 mL); α-sulfonyl enol ether product **14b** (122 mg, 0.280 mmol, 79% yield, white solid).

Reaction Time: 53 h

Rf: 0.4 in CH_2Cl_2

Column Chromatography: 90 : 10 hexanes : ethyl acetate

^{1}H NMR: (400 MHz, $CDCl_3$) δ = 7.74 (ddd, *J* = 9.8, 2.5, 2.4 Hz, 2H), 7.45 – 7.42 (m, 2H), 7.23 – 7.14 (m, 3H), 6.84 (ddd, *J* = 9.8, 2.5, 2.4 Hz, 2H), 6.68 (dddd, *J* = 15.9, 9.1, 2.8, 2.7 Hz, 4H), 4.20

(ddd, J = 9.4, 2.6, 1.6 Hz, 1H), 3.77 (s, 3H), 3.69 (s, 3H), 2.57 – 2.48 (m, 2H), 2.37 (m, 1H), 2.27 (m, 1H).

^{13}C{1H} NMR: (125 MHz, $CDCl_3$) δ = 163.8, 155.5, 148.8, 142.6, 133.5, 131.4, 130.5, 129.3, 128.3, 127.9, 127.1, 118.0, 114.6, 114.0, 68.9, 55.6 (2C), 29.3, 22.8.

IR: f (cm^{-1}) = 3029, 2959, 2928, 2866, 1595, 1499, 1262, 1210, 950.

HRMS (ESI-TOF): m/z $[M+Na]^+$ = 459.1242 calculated for $C_{25}H_{24}NaO_5S$; found 459.1246.

Compound 14c

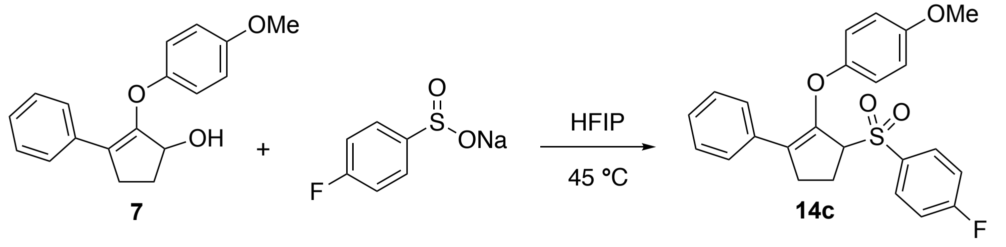

Quantity: α-hydroxy enol ether starting material **7** (100 mg, 0.354 mmol); sodium 4-fluorobenzenesulfinate (129 mg, 0.708 mmol); HFIP (3.5 mL); α-sulfonyl enol ether product **14c** (102 mg, 0.239 mmol, 68% yield, yellow solid).

Reaction Time: 49 h

Rf: 0.4 in CH_2Cl_2

Column Chromatography: 95 : 5 hexanes : ethyl acetate

^{1}H NMR: (500 MHz, $CDCl_3$) δ = 7.86 – 7.82 (m, 2H), 7.45 – 7.42 (m, 2H), 7.24 – 7.20 (m, 2H), 7.18 (m, 1H), 7.09 – 7.05 (m, 2H), 6.70 (ddd, J = 10.4, 3.2, 3.1 Hz, 2H), 6.62 (ddd, J = 10.4, 3.1, 2.9 Hz, 2H), 4.20 (ddd, J = 9.4, 2.6, 1.6 Hz, 1H), 3.69 (s, 3H) 2.61 – 2.53 (m, 2H), 2.44 (dtd, J = 15.7, 7.9, 2.5 Hz, 1H), 2.31 (dtd, J = 14.7, 9.5, 8.4 Hz, 1H).

^{13}C{1H} NMR: (125 MHz, $CDCl_3$) δ = 166.0 (d, $J_{C\text{-}F}$ = 254.6 Hz), 155.6, 148.6, 142.1, 134.0 (d, $J_{C\text{-}F}$ = 3.1 Hz), 133.3, 132.1 (d, $J_{C\text{-}F}$ = 9.5 Hz), 131.0, 128.4, 128.1, 127.1, 117.7, 116.1 (d, $J_{C\text{-}F}$ =

22.4 Hz), 114.7, 69.1, 55.7, 29.4, 22.6.

^{19}F NMR: (470 MHz, $CDCl_3$) δ = -103.6.

IR: *f* (cm^{-1}) = 3102, 3066, 2934, 2834, 1590, 1501, 1316, 1200, 1133, 810.

HRMS (ESI-TOF): *m/z* $[M+Na]^+$ = 447.1042 calculated for $C_{24}H_{21}FNaO_4S$; found 447.1050.

Compound 14d

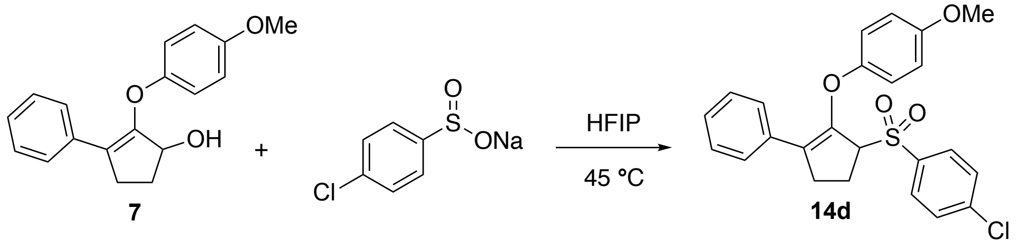

Quantity: α-hydroxy enol ether starting material **7** (101 mg, 0.357 mmol); sodium 4-chlorobenzenesulfinate (142 mg, 0.715 mmol); HFIP (3.6 mL); α-sulfonyl enol ether product **14d** (67 mg, 0.15 mmol, 43% yield, pale yellow solid).

Reaction Time: 65 h

Rf: 0.7 in CH_2Cl_2

Column Chromatography: CH_2Cl_2

^{1}H NMR: (400 MHz, $CDCl_3$) δ = 7.74 (d, *J* = 8.6 Hz, 2H), 7.44 – 7.42 (m, 2H), 7.37 – 7.34 (m, 2H), 7.24 – 7.15 (m, 3H), 6.71 – 6.65 (m, 2H), 6.59 – 6.55 (m, 2H), 4.25 (dt, *J* = 9.4, 1.7 Hz, 1H), 3.68 (s, 3H), 2.62 – 2.47 (m, 3H), 2.30 (m, 1H).

^{13}C{1H} NMR: (100 MHz, $CDCl_3$) δ = 155.6, 148.5, 141.9, 140.4, 136.7, 133.3, 131.1, 130.6, 129.1, 128.3, 128.1, 127.1, 117.6, 114.7, 69.1, 55.7, 29.5, 22.5.

IR: *f* (cm^{-1}) = 3058, 3022, 2930, 1646, 1581, 1500, 1198, 1032, 826, 751.

HRMS (ESI-TOF): *m/z* $[M+Na]^+$ = 463.0747 calculated for $C_{24}H_{21}ClNaO_4S$; found 463.0751.

Compound 14e

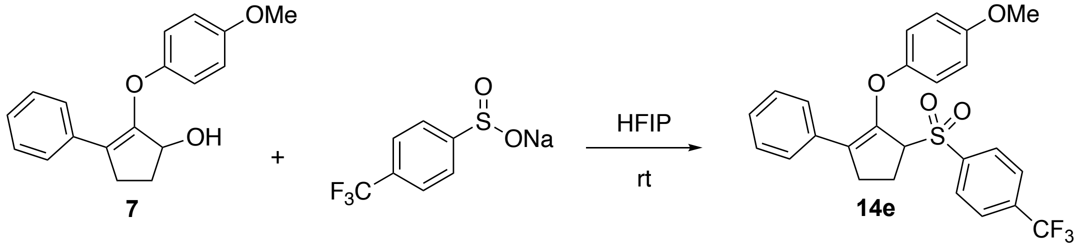

Quantity: α-hydroxy enol ether starting material **7** (100 mg, 0.355 mmol); 4-(trifluoromethyl) benzenesulfinic acid sodium salt (165 mg, 0.710 mmol); HFIP (3.5 mL); α-sulfonyl enol ether product **14e** (69 mg, 0.15 mmol, 41% yield, yellow oil).

Reaction Time: 144 h

Rf: 0.7 in CH_2Cl_2

Column Chromatography: 90 : 10 CH_2Cl_2 : hexanes

^{1}H NMR: (400 MHz, $CDCl_3$) δ = 8.01 (d, J = 8.0 Hz, 2H), 7.73 (d, J = 8.2 Hz, 2H), 7.50 – 7.47 (m, 2H), 7.32 – 7.23 (m, 3H), 6.72 (ddd, J = 10.5, 3.1, 3.0 Hz, 2H), 6.53 (ddd, J = 10.6, 3.1, 3.0 Hz, 2H), 4.39 (dq, J = 9.4, 1.4 Hz, 1H), 3.75 (s, 3H), 2.76 – 2.69 (m, 3H), 2.43 (m, 1H).

^{13}C{1H} NMR: (125 MHz, $CDCl_3$) δ = 155.5, 148.3, 142.0 (2C), 141.4, 135.2 (q, $J_{C\text{-}F}$ = 33.0 Hz), 133.1, 131.6, 129.7, 128.3 (d, $J_{C\text{-}F}$ = 10.9 Hz), 127.1, 125.9 (q, $J_{C\text{-}F}$ = 3.5 Hz), 123.2 (q, $J_{C\text{-}F}$ = 271.6 Hz), 117.1, 114.7, 69.1, 55.6, 29.6, 22.3.

^{19}F NMR: (470 MHz, $CDCl_3$) δ = -63.2.

IR: f (cm^{-1}) = 3004, 2923, 2833, 1639, 1500, 1434, 1320, 1215, 990, 800.

HRMS (ESI-TOF): m/z $[M+Na]^+$ = 497.1010 calculated for $C_{25}H_{21}F_3NaO_4S$; found 497.1017.

Compound 14g

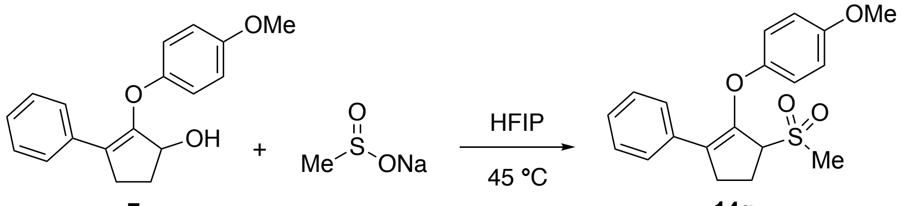

Quantity: α-hydroxy enol ether starting material **7** (100 mg, 0.355 mmol); methanesulfinic acid sodium salt (72 mg, 0.71 mmol); HFIP (3.6 mL); α-sulfonyl enol ether product **14g** (86 mg, 0.25 mmol, 71% yield, yellow solid).

Reaction Time: 48 h

Rf: 0.2 in CH_2Cl_2

Column Chromatography: 80 : 20 hexanes : ethyl acetate

^{1}H NMR: (400 MHz, $CDCl_3$) δ = 7.56 – 7.52 (m, 2H), 7.26 – 7.17 (m, 3H), 6.88 (ddd, *J* = 10.4, 3.0, 2.9 Hz, 2H), 6.79 (ddd, *J* = 10.4, 3.1, 3.0 Hz, 2H), 4.11 (dt, *J* = 9.6, 2.1 Hz, 1H), 3.71 (s, 3H), 3.07 (dtd, *J* = 15.8, 8.2, 2.6 Hz, 1H), 2.84 (s, 3H), 2.78 (ddd, *J* = 15.5, 9.2, 1.6 Hz, 1H), 2.65 (ddt, *J* = 14.7, 8.4, 1.7 Hz, 1H), 2.34 (dtd, *J* = 14.8, 9.4, 8.2 Hz, 1H).

^{13}C{1H} NMR: (100 MHz, $CDCl_3$) δ = 155.7, 148.5, 141.5, 133.2, 131.6, 128.4, 128.3, 127.3, 117.2, 115.2, 67.8, 55.7, 40.1, 30.1, 21.3.

IR: *f* (cm^{-1}) = 3053, 3031, 2898, 2750, 1680, 1494, 1450, 1275, 1211, 1034.

HRMS (ESI-TOF): *m/z* $[M+Na]^+$ = 367.0980 calculated for $C_{19}H_{20}NaO_4S$; found 367.0986.

Compound 14h

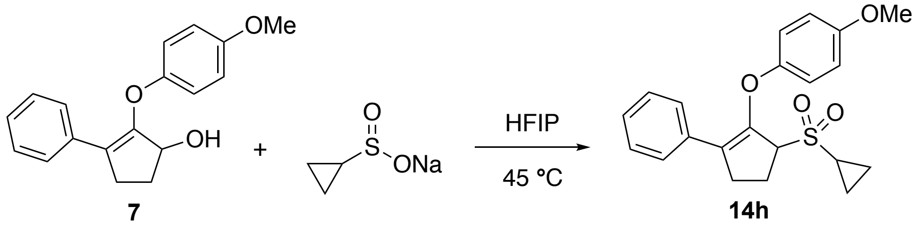

Quantity: α-hydroxy enol ether starting material **7** (100 mg, 0.354 mmol); cyclopropanesulfinic acid sodium salt (91 mg, 0.71 mmol); HFIP (3.5 mL); α-sulfonyl enol ether product **14h** (112 mg, 0.303 mmol, 86% yield, yellow oil).

Reaction Time: 72 h

Rf: 0.3 in CH_2Cl_2

Column Chromatography: 70 : 30 hexanes : ethyl acetate

^{1}H NMR: (400 MHz, $CDCl_3$) δ = 7.58 – 7.55 (m, 2H), 7.27 – 7.23 (m, 2H), 7.18 (m, 1H), 6.88 (ddd, *J* = 10.5, 3.2, 3.1 Hz, 2H), 6.78 (ddd, *J* = 10.5, 3.1, 3.0 Hz, 2H), 4.16 (dt, *J* = 9.6, 2.0 Hz, 1H), 3.70 (s, 3H), 3.06 (dtd, *J* = 15.5, 8.2, 2.6 Hz, 1H), 2.78 (ddd, *J* = 15.4, 9.3, 1.6 Hz, 1H) 2.64 (ddt, *J* = 14.6, 8.2, 1.6 Hz, 1H), 2.44 – 2.29 (m, 2H), 1.26 (m, 1H), 1.03 – 0.90 (m, 2H), 0.75 (m, 1H).

^{13}C{1H} NMR: (100 MHz, $CDCl_3$) δ = 155.6, 148.9, 142.1, 133.5, 131.1, 128.3, 128.1, 127.3, 117.4, 115.0, 67.1, 55.7, 30.1, 28.8, 21.9, 5.0, 4.5.

IR: *f* (cm^{-1}) = 3054, 2935, 2834, 1647, 1498, 1315, 1285, 850.

HRMS (ESI-TOF): *m/z* $[M+Na]^+$ = 393.1136 calculated for $C_{21}H_{22}NaO_4S$; found 393.1143.

Compound 14i

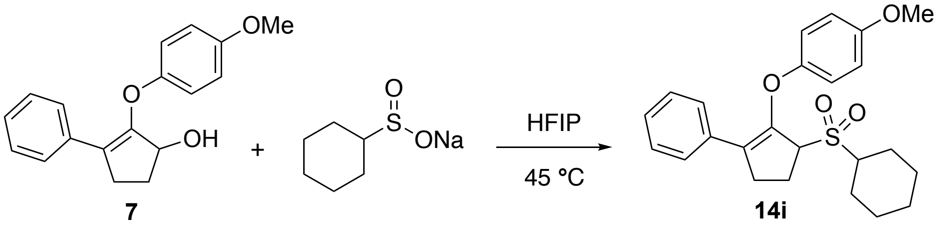

Quantity: α-hydroxy enol ether starting material **7** (101 mg, 0.354 mmol); cyclohexanesulfinic acid sodium salt (121 mg, 0.708 mmol); HFIP (3.6 mL); α-sulfonyl enol ether product **14i** (56 mg, 0.14 mmol, 39% yield, yellow oil).

Reaction Time: 60 h

Rf: 0.3 in CH_2Cl_2

Column Chromatography: 90 : 10 CH_2Cl_2 : hexanes

^{1}H NMR: (400 MHz, $CDCl_3$) δ = 7.58 – 7.55 (m, 2H), 7.31 – 7.22 (m, 3H), 6.95 (ddd, *J* = 10.3, 3.0, 2.9 Hz, 2H), 6.86 (ddd, *J* = 10.4, 3.0, 2.8 Hz, 2H), 4.31 (dt, *J* = 9.5, 1.9 Hz, 1H), 3.78 (s, 3H), 3.27 – 3.14 (m, 2H), 2.85 – 2.76 (m, 2H), 2.41 (dtd, *J* = 14.6, 9.0, 8.7 Hz, 1H), 2.22 (m, 1H) 1.93 (m, 1H), 1.69 (t, *J* = 10.6 Hz, 2H), 1.59 (m, 1H), 1.52 – 1.38 (m, 2H). 1.32 – 1.09 (m, 3H).

^{13}C{1H} NMR: (100 MHz, $CDCl_3$) δ = 155.5, 148.9, 141.3, 133.3, 132.7, 128.3, 128.2, 127.3, 116.2, 115.1, 63.1, 60.5, 55,7, 30.4, 26.4, 25.3, 25.1, 25.0, 22.9, 21.2.

IR: *f* (cm^{-1}) = 3060, 2929, 2857, 1643, 1504, 1439, 1191, 990, 820.

HRMS (ESI-TOF): *m/z* $[M+Na]^+$ = 435.1606 calculated for $C_{24}H_{28}NaO_4S$; found 435.1614.

Compound 15b

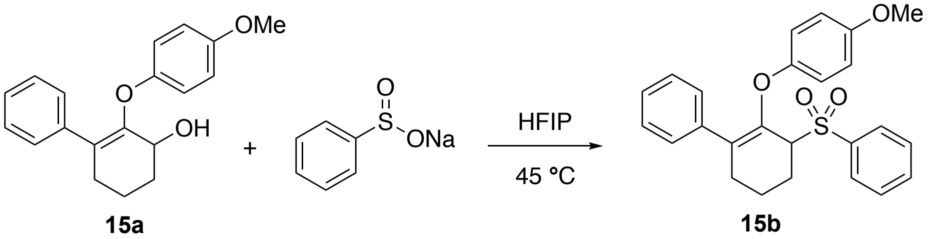

Quantity: α-hydroxy enol ether starting material **15a** (120 mg, 0.404 mmol); sodium 4-chlorobenzenesulfinate (162 mg, 0.807 mmol); HFIP (4.0 mL); α-sulfonyl enol ether product **15b** (45 mg, 0.11 mmol, 27% yield, white solid).

Reaction Time: 10 d

Rf: 0.6 in 99.5:0.5 CH_2Cl_2 : MeOH

Column Chromatography: CH_2Cl_2

^{1}H NMR: (400 MHz, $CDCl_3$) δ = 7.84 – 7.81 (m, 2H), 7.54 (tt, *J* = 6.8, 1.3 Hz, 1H), 7.44 – 7.40 (m, 2H), 7.22 – 7.19 (m, 2H), 7.17 – 7.12 (m, 2H), 7.07 (m, 1H), 6.53 – 6.49 (m, 2H), 6.19 – 6.14 (m, 2H), 3.89 (dd, *J* = 6.6, 2.7 Hz, 1H), 3.59 (s, 3H), 2.69 (dq, *J* = 14.4, 3.7 Hz,1H), 2.55 (dddd, *J* = 17.8, 10.0, 6.4, 1.6 Hz, 1H), 2.43 (ddd, *J* = 17.8, 6.1, 3.1 Hz, 1H), 2.18 (dddd, *J* = 19.4, 13.4, 9.7, 3.7 Hz, 1H), 1.94 (dddd, *J* = 14.3, 12.8, 6.1, 3.8 Hz, 1H), 1.75 (ddq, *J* = 13.9, 7.0, 3.6 Hz, 1H).

^{13}C{1H} NMR: (100 MHz, $CDCl_3$) δ = 154.6, 149.0, 140.4, 138.4, 138.1, 133.3, 132.6, 129.0, 128.8, 127.9, 127.5, 127.3, 116.6, 114.4, 62.04, 55.5, 29.6, 24.1, 18.8.

IR: *f* (cm^{-1}) = 3020, 2932, 2836, 1661, 1586, 1501, 1202, 1034, 830, 747.

HRMS (ESI-TOF): *m/z* $[M+Na]^+$ = 443.1293 calculated for $C_{25}H_{24}NaO_4S$; found 443.1300.

Experimental Data for Scheme 4

Compound 16a

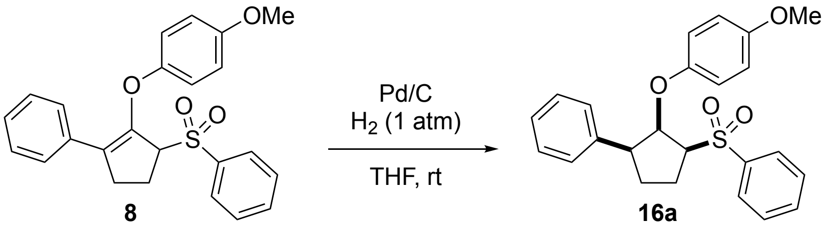

Procedure: Pd/C (524 mg, 0.492 mmol, 10% Pd/C) was introduced in a round-bottomed flask. After vacuum purging with N_2 gas, THF (4 mL) was then added, followed by α-sulfonyl enol ether **8** (200 mg, 0.492 mmol) as a solution in THF (2 x 1 mL). The H_2 gas balloon was then attached. The reaction mixture was stirred at room temperature for 24 h when the starting material was fully consumed, as monitored by TLC. After filtering the solid particles through a pad of celite, the mixture was concentrated under vacuum. The crude material was then purified with column chromatography using 90:10 hexanes : ethyl acetate to yield product **16a** (117 mg, 0.286 mmol, 58% yield, white solid). The ^{1}H NMR of the crude mixture indicated as a single diastereomer.

Rf: 0.5 in 60:40 hexanes : ethyl acetate.

^{1}H NMR: (400 MHz, $CDCl_3$) δ = 7.82 – 7.79 (m, 2H), 7.58 (m, 1H), 7.46 – 7.42 (m, 2H), 7.23 – 7.20 (m, 2H), 7.17 – 7.08 (m, 3H), 6.53 (ddd, *J* = 10.6, 3.2, 3.1 Hz, 2H), 6.41 (ddd, *J* = 10.6, 3.2, 3.0 Hz, 2H), 4.97 (t, J = 3.6 Hz, 1H), 3.81 (m, 1H), 3.67 (s, 3H), 3.22 (m, 1H), 2.66 (m, 1H), 2.55 (m, 1H), 2.23 – 2.16 (m, 2H).

^{13}C{1H} NMR: (100 MHz, $CDCl_3$) δ = 153.8, 152.8, 139.8, 137.8, 133.3, 128.92, 128.85, 128.6, 128.1, 127.0, 117.6, 113.8, 83.5, 69.4, 55.5, 50.8, 27.9, 23.8.

IR: *f* (cm^{-1}) = 3062, 2955, 2820, 1506, 1439, 1208, 1150, 1044, 830.

HRMS (ESI-TOF): *m/z* $[M+H]^+$ = 409.1474 calculated for $C_{24}H_{25}O_4S$; found 409.1477.

X-Ray Crystallography:

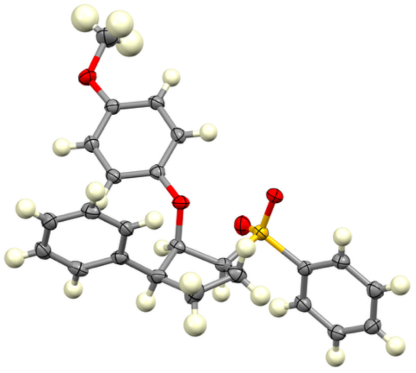

Compound 16b

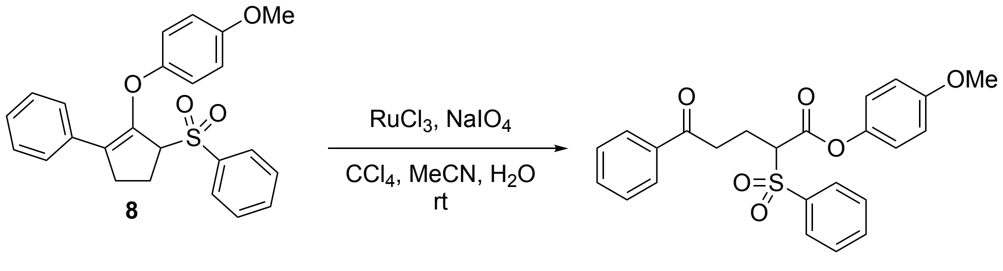

Procedure: α-Sulfonyl enol ether **8** (249 mg, 0.613 mmol) was introduced in a round-bottomed flask and dissolved in CCl_4:MeCN:H_2O (1:1:1.3 ratio, 12.3 mL). Then, NaIO4 (538 mg, 2.51 mmol) and $RuCl_3$ (5.2 mg, 0.021 mmol) was then added sequentially. The reaction mixture was stirred at room temperature for 15 min when the starting material was fully consumed, as monitored by TLC. After the addition of a saturated aqueous solution of $Na_2S_2O_3$ (5 mL), the reaction mixture was stirred for 15 min and diluted with H_2O (10 mL). The aqueous layer was then extracted with CH_2Cl_2 (3 x 15 mL). The combined organic layers were dried over Na_2SO_4 and then concentrated under vacuum. The crude material was then purified with column chromatography using 70:30 hexanes : ethyl acetate to yield product **16b** (244 mg, 0.557 mmol, 91% yield, white solid).

Rf: 0.3 in 70:30 hexanes : ethyl acetate

^{1}H NMR: (400 MHz, $CDCl_3$) δ = 7.99 – 7.92 (m, 4H), 7.69 (tt, *J* = 6.8, 1.2 Hz, 1H), 7.59 – 7.55 (m, 3H), 7.48-7.44 (m, 2H), 6.89 – 6.82 (m, 4H), 4.44 (dd, *J* = 9.3, 5.2 Hz, 1H), 3.77 (s, 3H), 3.31 (dt, *J* = 18.0, 6.8 Hz, 1H), 3.20 (dt, *J* = 18.0, 6.90 Hz, 1H), 2.64 (dtd, *J* = 14.3, 7.0, 5.2 Hz, 1H), 2.51 (ddt, *J* = 14.3, 9.3, 6.7 Hz, 1H).

^{13}C{1H} NMR: (125 MHz, $CDCl_3$) δ = 197.8, 164.8, 157.6, 143.5, 137.2, 136.2, 134.4, 133.5, 129.3, 129.2, 128.7, 127.9, 121.7, 114.4, 69.5, 55.5, 35.0, 21.5.

IR: *f* (cm^{-1}) = 3063, 3012, 2933, 1752, 1682, 1504, 1226, 1029, 752, 723.

HRMS (ESI-TOF): *m/z* $[M+Na]^+$ = 461.1035 calculated for $C_{24}H_{22}NaO_6S$; found 461.1046.

X-Ray Crystallography:

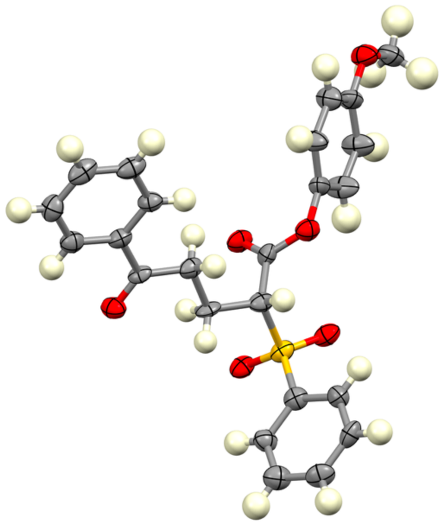

Compound 16c

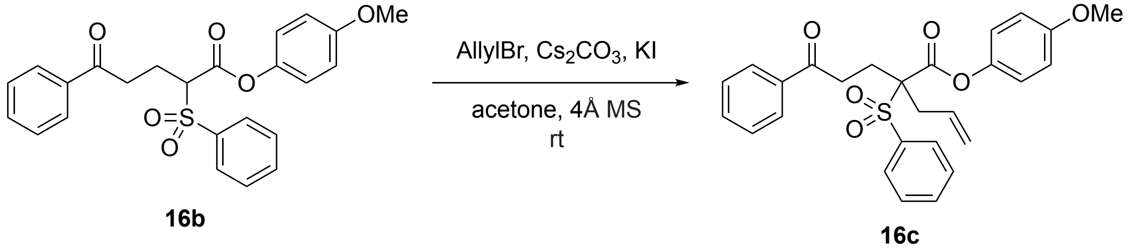

Procedure: α-Sulfonyl 1,5-ketoester **16b** (124 mg, 0.283 mmol) was introduced in a round-bottomed flask and dissolved in anhydrous acetone (2.8 mL). Then, allyl bromide (37 μL, 0.42 mmol), Cs_2CO_3 (185 mg, 0.566 mmol), KI (4.7 mg, 0.028 mmol), and 4Å molecular sieves (200 mg) were then added sequentially. The reaction mixture was stirred at room temperature for 1 h when the starting material was fully consumed, as monitored by TLC. After removal of acetone under vacuum and addition of H_2O (20 mL), the aqueous layer was then extracted with CH_2Cl_2 (3 x 15 mL). The combined organic layers were dried over Na_2SO_4 and then concentrated under vacuum. The crude material was then purified with column chromatography using 90:10 hexanes : ethyl acetate to yield product **16c** (77 mg, 0.16 mmol, 57% yield, yellow oil).

Rf: 0.5 in 70:30 hexanes : ethyl acetate

^{1}H NMR: (400 MHz, $CDCl_3$) δ = 8.01 – 7.99 (m, 2H), 7.95 – 7.92 (m, 2H), 7.68 (tt, *J* = 6.8, 1.2 Hz, 1H), 7.59 – 7.53 (m, 3H), 7.49 – 7.45 (m, 2H), 6.98 – 6.94 (m, 2H), 6.90 – 6.86 (m, 2H), 5.84 (ddt, *J* = 17.1, 10.0, 7.2 Hz, 1H), 5.30 – 5.22 (m, 2H), 3.80 (s, 3H), 3.62 (ddd, *J* = 17.7, 11.3, 4.4 Hz, 1H), 3.37 (ddd, *J* = 17.7, 11.4, 4.7 Hz, 1H), 3.00 – 2.88 (m, 2H), 2.77 (ddd, *J* = 15.4, 11.3, 4.4 Hz, 1H), 2.53 (ddd, *J* = 15.6, 11.3, 4.7 Hz, 1H).

^{13}C{1H} NMR: (125 MHz, $CDCl_3$) δ = 198.4, 166.6, 157.7, 143.6, 136.6, 136.2, 134.4, 133.2, 130.64, 130.59, 128.9, 128.6, 128.1, 121.8, 121.0, 114.6, 75.4, 55.6, 37.1, 33.5, 25.5.

IR: *f* (cm^{-1}) = 3065, 3015, 2926, 2838, 1746, 1684, 1503, 1213, 749.

HRMS (ESI-TOF): *m/z* $[M+H]^+$ = 479.1528 calculated for $C_{27}H_{27}O_6S$; found 479.1537.

Compound 16d

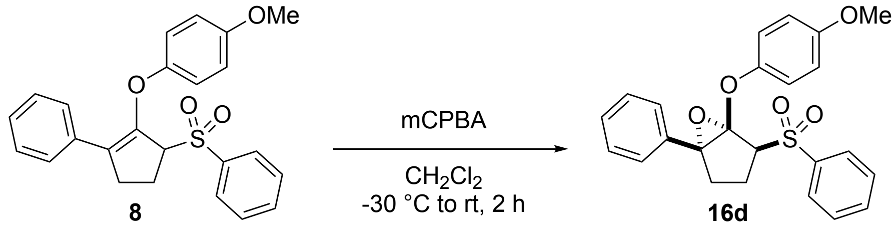

Procedure: α-Sulfonyl enol ether **8** (200 mg, 0.492 mmol) was introduced in a round-bottomed flask and dissolved in CH_2Cl_2 (3.0 mL). After cooling to -30 °C, mCPBA (170 mg, 0.738 mmol, 75% purity) was then added as a solution in CH_2Cl_2 (3.0 mL). The reaction mixture was allowed to warm up to room temperature and stirred for 2 h until the starting material was fully consumed, as monitored by TLC. After the addition of a saturated solution of $NaHCO_3$ (4 mL), the aqueous layer was then extracted with CH_2Cl_2 (3 x 15 mL). The combined organic layers were dried over Na_2SO_4 and then concentrated under vacuum. The crude material was then purified with column chromatography using 80:20 hexanes : ethyl acetate to yield product **16d** (172 mg, 0.406 mmol, 83% yield, yellow oil).

Rf: 0.5 in 60:40 hexanes : ethyl acetate

^{1}H NMR: (400 MHz, $CDCl_3$) δ = 7.87 – 7.85 (m, 2H), 7.53 (m, 1H), 7.42 – 7.37 (m, 2H), 7.32 (s, 5H), 6.73 (ddd, J = 10.4, 3.1, 2.9 Hz, 2H), 6.64 (ddd, J = 10.4, 3.0, 2.8 Hz, 2H), 4.34 (d, J = 7.9 Hz, 1H), 3.73 (s, 3H), 2.58 – 2.48 (m, 2H), 2.14 – 2.00 (m, 2H).

^{13}C{1H} NMR: (125 MHz, $CDCl_3$) δ = 155.1, 148.4, 138.7, 133.6, 133.1, 129.0, 128.8, 128.3, 128.1, 126.9, 118.1, 114.2, 91.4, 71.9, 64.4, 55.6, 29.0, 22.3.

IR: f (cm^{-1}) = 3062, 2925, 2830, 1714, 1573, 1494, 1440, 1208, 1132, 890.

HRMS (ESI-TOF): m/z $[M+Na]^+$ = 445.1086 calculated for $C_{24}H_{22}NaO_5S$; found 445.1092.

X-Ray Crystallography:

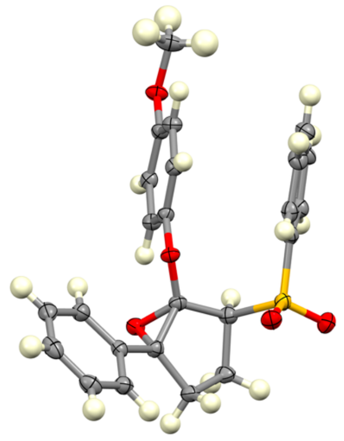

Compound 16e

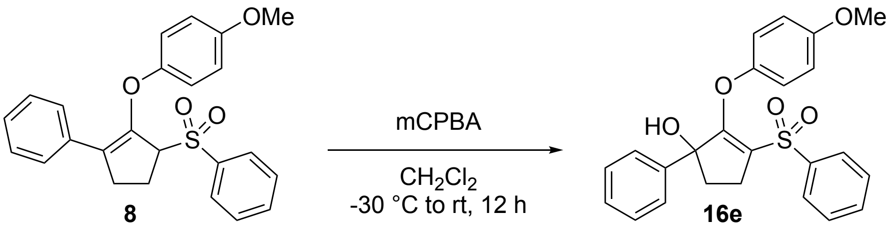

Procedure: α-Sulfonyl enol ether **8** (200 mg, 0.492 mmol) was introduced in a round-bottomed flask and dissolved in CH_2Cl_2 (3.0 mL). After cooling to -30 °C, mCPBA (170 mg, 0.738 mmol, 75% purity) was then added as a solution in CH_2Cl_2 (3.0 mL). The reaction mixture was allowed to warm up to room temperature and stirred for 12 h until epoxide **16d** was fully consumed, as monitored by TLC. After the addition of a saturated solution of $NaHCO_3$ (4.0 mL), the aqueous layer was then extracted with CH_2Cl_2 (3 x 15 mL). The combined organic layers were dried over Na_2SO_4 and then concentrated under vacuum. The crude material was then purified with column chromatography using 85:15 hexanes : ethyl acetate to yield product **16e** (142 mg, 0.337 mmol, 68% yield, yellow solid).

Rf: 0.4 in 60:40 hexanes : ethyl acetate

^{1}H NMR: (500 MHz, $CDCl_3$) δ = 7.97 – 7.94 (m, 2H), 7.63 (m, 1H), 7.54 – 7.51 (m, 2H), 7.27 – 7.23 (m, 3H), 7.15 – 7.12 (m, 2H), 6.56 (ddd, *J* = 10.3, 3.1, 3.0 Hz, 2H), 6.49 (ddd, *J* = 10.4, 2.9, 2.8 Hz, 2H), 3.71 (s, 3H), 2.89 (ddd, *J* = 15.6, 8.2, 4.1 Hz, 1H), 2.73 (ddd, *J* = 15.6, 8.1, 6.1 Hz, 1H), 2.31 – 2.22 (m, 2H), 1.87 (s, 1H).

^{13}C{1H} NMR: (125 MHz, $CDCl_3$) δ = 161.4, 156.4, 148.1, 143.2, 141.0, 133.3, 128.9, 128.5, 127.8, 127.7, 124.4, 123.9, 120.9, 114.1, 85.6, 55.5, 39.7, 25.6.

IR: *f* (cm^{-1}) = 3415, 3070, 2919, 2851, 1692, 1502, 1195, 1132, 990.

HRMS (ESI-TOF): *m/z* $[M+Na]^+$ = 445.1086 calculated for $C_{24}H_{22}NaO_5S$; found 445.1090.

X-Ray Crystallography:

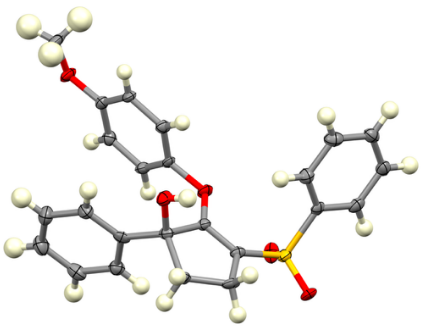

Compound 16f

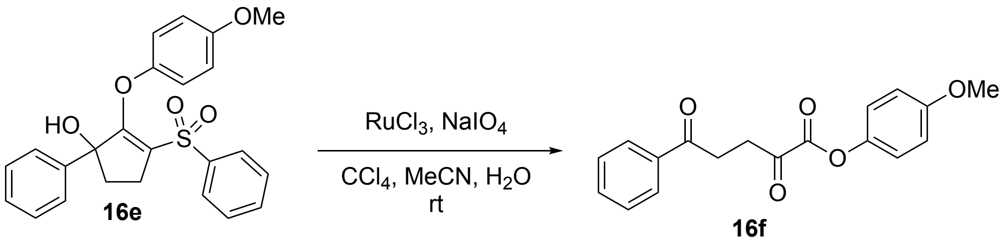

Procedure: α-Hydroxy enol ether **16e** (41 mg, 0.096 mmol) was introduced in a round-bottomed flask and dissolved in CCl_4:MeCN:H_2O (1:1:1.3 ratio, 6.3 mL). Then, $NaIO_4$ (85 mg, 0.40 mmol) and $RuCl_3$ (1.0 mg, 0.0034 mmol) was then added sequentially. The reaction mixture was stirred at room temperature for 15 min when the starting material was fully consumed, as monitored by

TLC. After the addition of a saturated aqueous solution of $Na_2S_2O_3$ (2.5 mL), the reaction mixture was stirred for 15 min and diluted with H_2O (5 mL). The aqueous layer was then extracted with CH_2Cl_2 (3 x 10 mL). The combined organic layers were dried over Na_2SO_4 and then concentrated under vacuum. The crude material was then purified with column chromatography using 80:20 hexanes : ethyl acetate to yield product **16f** (15 mg, 0.048 mmol, 50% yield, white solid).

Rf: 0.4 in 70:30 hexanes : ethyl acetate

^{1}H NMR: (400 MHz, CDCl3) δ = 8.03 – 7.99 (m, 2H), 7.59 (m, 1H), 7.52 – 7.47 (m, 2H), 7.17 – 7.12 (m, 2H), 6.95 – 6.91 (m, 2H), 3.82 (s, 3H), 3.50 – 3.45 (m, 2H), 3.39 – 3.35 (m, 2H).

^{13}C{1H} NMR: (125 MHz, $CDCl_3$) δ = 197.5, 192.5, 159.4, 157.8, 143.6, 136.1, 133.5, 128.7, 128.2, 121.9, 114.6, 55.6, 33.2, 33.0.

IR: *f* (cm^{-1}) = 3065, 3021, 2965, 2927, 1734, 1687, 1594, 1505, 871, 763.

HRMS (ESI-TOF): *m/z* $[M+H]^+$ = 313.1076 calculated for $C_{18}H_{17}O_5$; found 313.1083.

X-Ray Crystallography:

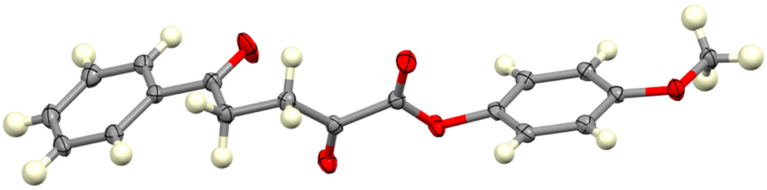

Synthesis of Substrates

General Sequence and Procedure:

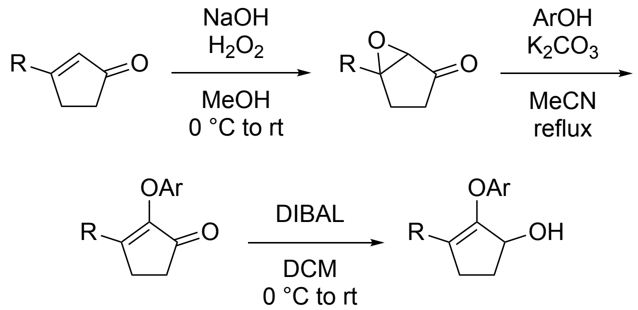

Epoxidation: Cyclic enone was introduced into a round-bottomed flask and dissolved in methanol. After cooling to 0 °C, aqueous solutions of H_2O_2 (30%) and NaOH (4 M) were added sequentially. The reaction mixture was allowed to warm up to room temperature and stirred until the starting material was fully consumed, as monitored by TLC. After cooling to 0 °C, DI water (15 mL) was added. The aqueous layer was then extracted with CH_2Cl_2 (3 x 15 mL). The combined organic layers were dried over Na_2SO_4 and then concentrated under vacuum. The crude material was taken on the next step without purification.

Ring Opening: The crude epoxide was combined with substituted phenol and potassium (or cesium) carbonate in a round-bottomed flask, followed the addition of acetonitrile. The reaction mixture was then warmed to reflux until the epoxide was fully consumed, as monitored by TLC. After cooling to room temperature, the reaction mixture was diluted with an aqueous NaOH solution (1 M, 20 mL). The aqueous layer was then extracted with CH_2Cl_2 (3 x 15 mL). The combined organic layers were dried over Na_2SO_4 and then concentrated under vacuum. The crude material was purified with column chromatography to yield the *O*-aryl enol ether adduct.

Reduction: The *O*-aryl enol ether adduct was introduced to a round-bottomed flask and dissolved in CH_2Cl_2. After cooling to 0 °C, DIBAL (1.1 or 1.0 M in cyclohexane) was added dropwise. The

reaction mixture was allowed to warm up to room temperature and stirred until the starting material was fully consumed, as monitored by TLC. After cooling to 0 °C, an aqueous sodium potassium tartrate (20 mL) was added, and the mixture was stirred vigorously until a separation of layers was achieved. The aqueous layer was then extracted with CH_2Cl_2 (3 x 15 mL). The combined organic layers were dried over Na_2SO_4 and then concentrated under vacuum. The crude material was purified with column chromatography to yield the α-hydroxy *O*-aryl enol ether.

Compound 7

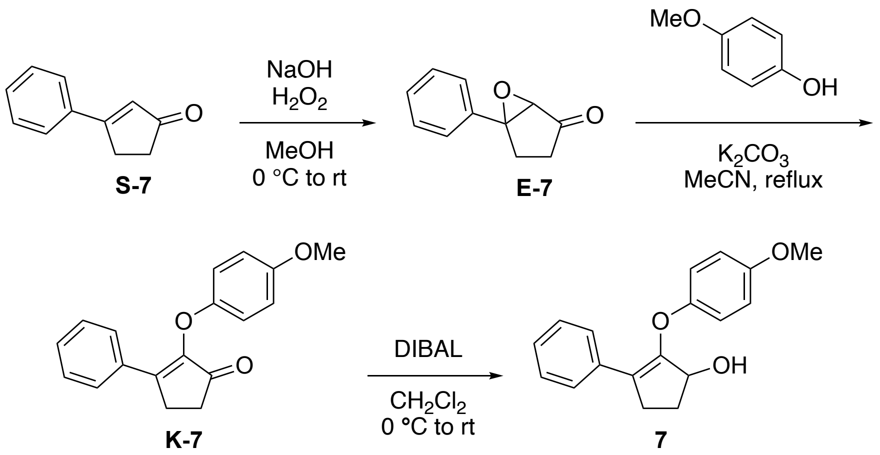

Epoxidation

Quantity: Starting material **S-7** (1.80 g, 11.4 mmol); H_2O_2 (5.8 mL, 56.9 mmol, 30% in H_2O); NaOH (2.8 mL, 11.4 mmol, 4 M in H_2O); MeOH (20 mL); epoxide **E-7** (1.18 g, crude, yellow oil).

Reaction Time: 30 min

Rf: 0.6 in 70:30 hexanes : ethyl acetate

Ring Opening

Quantity: Epoxide **E-7** (1.18 g, crude); 4-methoxyphenol (1.26 g, 10.2 mmol); K_2CO_3 (1.12 g, 8.13 mmol); MeCN (27 mL); *O*-aryl enol ether **K-7** (1.41 g, 5.03 mmol, 44% yield over two steps).

Reaction Time: 14 h

Rf: 0.5 in 60:40 hexanes : ethyl acetate

Column Chromatography: 70:30 hexanes : ethyl acetate

Reduction

Quantity: Starting material **K-7** (1.41 g, 5.03 mmol); DIBAL (5.0 mL, 5.53 mmol, 1.1 M in cyclohexane); CH_2Cl_2 (25 mL); α-hydroxy enol ether product **7** (1.31 g, 4.64 mmol, 92% yield, white solid).

Reaction Time: 1 h

Rf: 0.4 in 70:30 hexanes : ethyl acetate

Column Chromatography: 75:25 hexanes : ethyl acetate

^{1}H NMR: (400 MHz, $CDCl_3$) δ = 7.69 – 7.66 (m, 2H), 7.34 – 7.30 (m, 2H), 7.23 (m, 1H), 7.00 (ddd, *J* = 10.5, 3.3, 3.1 Hz, 2H), 6.84 (ddd, *J* = 10.5, 3.2, 3.0 Hz, 2H), 4.88 (m, 1H), 3.78 (s, 3H), 2.94 (dddd, *J* = 15.6, 8.8, 4.8, 2.1 Hz, 1H), 2.71 (dddd, *J* = 15.3, 8.7, 4.3, 0.8 Hz, 1H), 2.37 (dddd, *J* = 13.8, 8.7, 7.7, 4.8 Hz, 1H), 1.91 (dddd, *J* = 13.8, 8.9, 4.3, 3.6 Hz, 1H), 1.78 (d, *J* = 5.2 Hz, 1H).

^{13}C{1H} NMR: (100 MHz, $CDCl_3$) δ = 155.5, 150.8, 149.2, 134.3, 128.2, 127.3, 127.1, 123.5, 118.6, 114.8, 73.4, 55.7, 29.6, 28.3.

IR: *f* (cm^{-1}) = 3444, 3086, 2917, 1651, 1502, 1437, 1216, 1177, 970.

HRMS (ESI-TOF): *m/z* $[M{-}OH]^+$ = 265.1229 calculated for $C_{18}H_{17}O_2$; found 265.1238.

Compound 10a

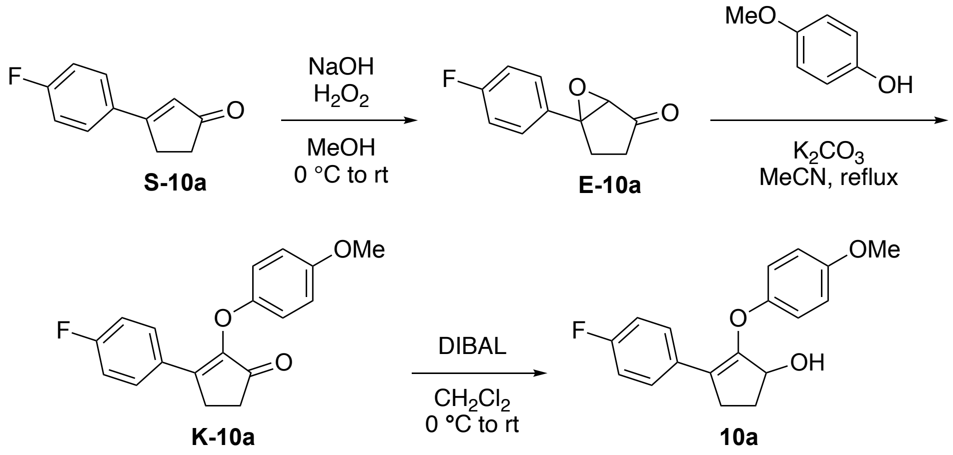

Epoxidation

Quantity: Starting material **S-10a** (2.04 g, 11.6 mmol); H_2O_2 (5.9 mL, 57.8 mmol, 30% in H_2O); NaOH (2.9 mL, 11.6 mmol, 4 M in H_2O); MeOH (23 mL); epoxide **E-10a** (1.03 g, crude, yellow oil).

Reaction Time: 1 h

Rf: 0.6 in 80:20 hexanes : ethyl acetate

Ring Opening

Quantity: Epoxide **E-10a** (1.03 g, crude); 4-methoxyphenol (0.998 g, 8.04 mmol); K_2CO_3 (0.889 g, 6.43 mmol); MeCN (54 mL); *O*-aryl enol ether **K-10a** (1.00 g, 3.36 mmol, 29% yield over two steps).

Reaction Time: 12 h

Rf: 0.2 in 80:20 hexanes : ethyl acetate

Column Chromatography: 70:30 hexanes : ethyl acetate

Reduction

Quantity: Starting material **K-10a** (136 mg, 0.457 mmol); DIBAL (0.62 mL, 0.69 mmol, 1.1 M in cyclohexane); CH_2Cl_2 (2.3 mL); α-hydroxy enol ether product **10a** (126 mg, 0.421 mmol, 92% yield, pale yellow solid).

Reaction Time: 50 min

Rf: 0.5 in 70:30 hexanes : ethyl acetate

Column Chromatography: 75:25 hexanes : ethyl acetate

^{1}H NMR: (400 MHz, $CDCl_3$) δ = 7.67 – 7.63 (m, 2H), 7.03 – 6.97 (m, 4H), 6.86 – 6.82 (m, 2H), 4.86 (m, 1H), 3.77 (s, 3H), 2.89 (dddd, *J* = 15.5, 8.8, 4.8, 2.1 Hz, 1H), 2.67 (ddd, *J* = 15.2, 8.6, 4.3 Hz, 1H), 2.36 (dddd, *J* = 13.6, 8.6, 7.7, 4.8 Hz, 1H), 1.93 – 1.86 (m, 2H).

^{13}C{1H} NMR: (100 MHz, $CDCl_3$) δ = 161.8 (d, $J_{C\text{-}F}$ = 247.2 Hz), 155.5, 150.4 (d, $J_{C\text{-}F}$ = 2.0 Hz), 149.0, 130.5 (d, $J_{C\text{-}F}$ = 3.3 Hz), 128.8 (d, $J_{C\text{-}F}$ = 7.9 Hz), 122.4, 118.6, 115.1 (d, $J_{C\text{-}F}$ = 21.2 Hz), 114.7, 73.2, 55.6, 29.5, 28.4.

^{19}F NMR: (470 MHz, $CDCl_3$) δ = -114.1.

IR: *f* (cm^{-1}) = 3358, 3048, 3002, 2934, 2836, 1602, 1500, 1208, 1034, 830.

HRMS (ESI-TOF): *m/z* $[M{-}OH]^+$ = 283.1134 calculated for $C_{18}H_{16}FO_2$; found 283.1151.

Compound 10b

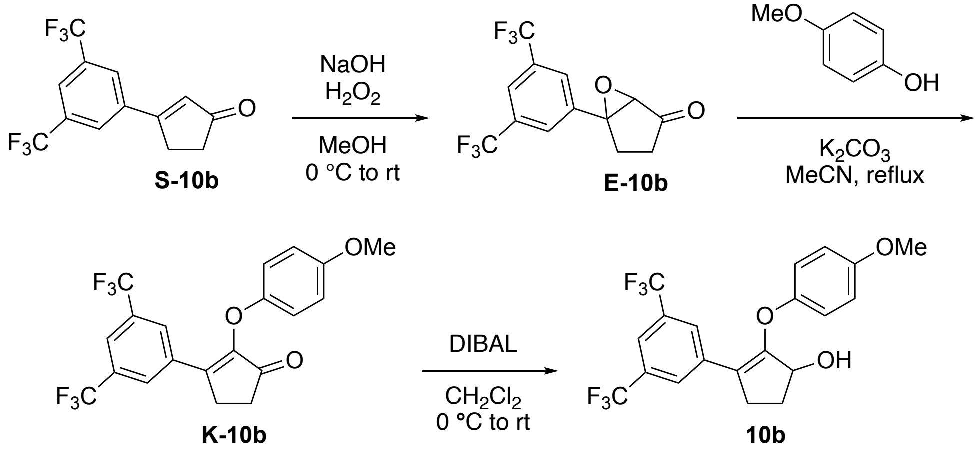

Epoxidation

Quantity: Starting material **S-10b** (1.86 g, 6.32 mmol); H_2O_2 (3.2 mL, 31.6 mmol, 30% in H_2O); NaOH (1.60 mL, 6.32 mmol, 4 M in H_2O); MeOH (30 mL); epoxide **E-10b** (1.44 g, crude, yellow oil).

Reaction Time: 25 min

Rf: 0.6 in 70:30 hexanes : ethyl acetate

Ring Opening

Quantity: Epoxide **E-10b** (1.40 g, crude); 4-methoxyphenol (0.840 g, 6.77 mmol); K_2CO_3 (0.749 g, 5.42 mmol); MeCN (18 mL); *O*-aryl enol ether **K-10b** (0.4 g, 0.961 mmol, 15% yield over two steps).

Reaction Time: 12 h

Rf: 0.6 in 70:30 hexanes : ethyl acetate

Column Chromatography: 80:20 hexanes : ethyl acetate

Reduction

Quantity: Starting material **K-10b** (400 mg, 0.961 mmol); DIBAL (0.96 mL, 1.06 mmol, 1.1 M in cyclohexane); CH_2Cl_2 (4.8 mL); α-hydroxy enol ether product **10b** (296 mg, 0.708 mmol, 74% yield, white solid).

Reaction Time: 45 min

Rf: 0.5 in 70:30 hexanes : ethyl acetate

Column Chromatography: 85:15 hexanes : ethyl acetate

^{1}H NMR: (500 MHz, $CDCl_3$) δ = 8.10 (d, *J* = 1.8 Hz, 2H), 7.71 (s, 1H), 7.02 (ddd, *J* = 10.4, 3.1, 3.0 Hz, 2H), 6.86 (ddd, *J* = 10.6, 3.0, 2.9 Hz, 2H), 4.92 (ddd, *J* = 7.8, 3.6, 1.9 Hz, 1H), 3.79 (s, 3H), 2.93 (dddd, *J* = 15.2, 8.9, 4.8, 2.1 Hz, 1H), 2.74 (ddd, J = 15.1, 8.6, 4.4 Hz, 1H), 2.42 (m, 1H), 1.96 (m, 1H), 1.75 (s, 1H).

^{13}C{1H} NMR: (125 MHz, $CDCl_3$) δ = 156.2, 154.7, 148.4, 136.6, 131.4 (q, $J_{C\text{-}F}$ = 32.7 Hz), 126.8 (q, $J_{C\text{-}F}$ = 3.9 Hz), 123.4 (q, $J_{C\text{-}F}$ = 271.1 Hz), 120.3 (m), 119.5, 118.8, 114.8, 73.1, 55.6, 29.4, 28.

^{19}F NMR: (470 MHz, $CDCl_3$) δ = -62.9.

IR: *f* (cm^{-1}) = 3394, 3105, 2949, 2844, 1641, 1505, 1292, 1122, 880.

HRMS (ESI-TOF): *m/z* $[M–OH]^+$ = 401.0976 calculated for $C_{20}H_{15}F_6O_2$; found 401.0978.

Compound 10c

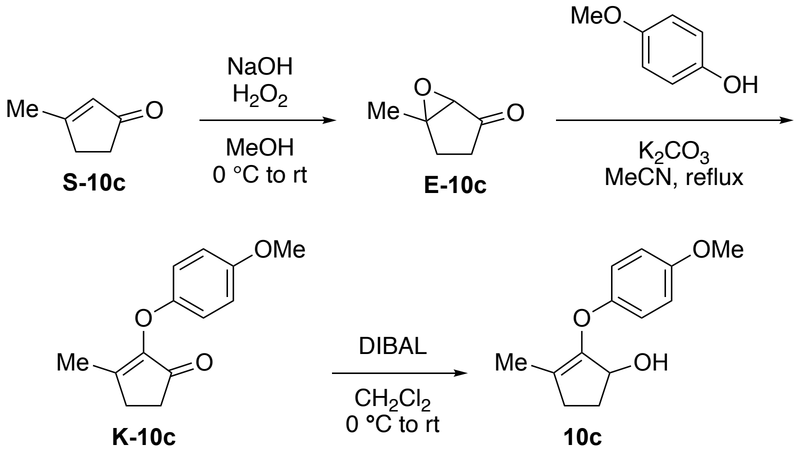

Epoxidation

Quantity: Starting material **S-10c** (2.1 mL, 20.8 mmol); H_2O_2 (10.6 mL, 104 mmol, 30% in H_2O); NaOH (5.2 mL, 20.8 mmol, 4 M in H_2O); MeOH (42 mL); epoxide **E-10c** (0.800 g, crude, yellow oil).

Reaction Time: 10 min

Rf: 0.5 in 80:20 hexanes : ethyl acetate

Ring Opening

Quantity: Epoxide **E-10c** (0.800 g, crude); 4-methoxyphenol (1.33 g, 10.7 mmol); Cs_2CO_3 (2.79 g, 8.56 mmol); MeCN (29 mL); *O*-aryl enol ether **K-10c** (0.74 g, 3.37 mmol, 16% yield over two steps).

Reaction Time: 50 min

Rf: 0.3 in 70:30 hexanes : ethyl acetate

Column Chromatography: 70:30 hexanes : ethyl acetate

Reduction

Quantity: Starting material **K-10c** (736 mg, 3.37 mmol); DIBAL (3.4 mL, 3.71 mmol, 1.1 M in cyclohexane); CH_2Cl_2 (16.9 mL); α-hydroxy enol ether product **10c** (731 mg, 3.32 mmol, 98% yield, colorless oil).

Reaction Time: 4 h

Rf: 0.3 in 80:20 hexanes : ethyl acetate

Column Chromatography: 75:25 hexanes : ethyl acetate

Rf: 0.3 in 80 : 20 hexanes : ethyl acetate

^{1}H NMR: (500 MHz, $CDCl_3$) δ = 6.91 (ddd, J = 10.5, 3.0, 2.9 Hz, 2H), 6.82 (ddd, J = 10.4, 3.0, 2.8 Hz, 2H), 4.73 (m, 1H), 3.77 (s, 3H), 2.46 (m, 1H), 2.32 – 2.19 (m, 2H), 1.82 – 1.76 (m, 2H), 1.62 (q, J = 1.3 Hz, 3H).

^{13}C{1H} NMR: (125 MHz, $CDCl_3$) δ = 154.8, 150.7, 148.8, 124.5, 117.4, 114.6, 73.3, 55.7, 30.9, 30.1, 12.3.

IR: f (cm^{-1}) = 3454, 3090, 2910, 2870, 1684, 1500, 1439, 1210, 810.

HRMS (ESI-TOF): m/z $[M{-}OH]^+$ = 203.1072 calculated for $C_{13}H_{15}O_2$; found 203.1069.

Compound 10d

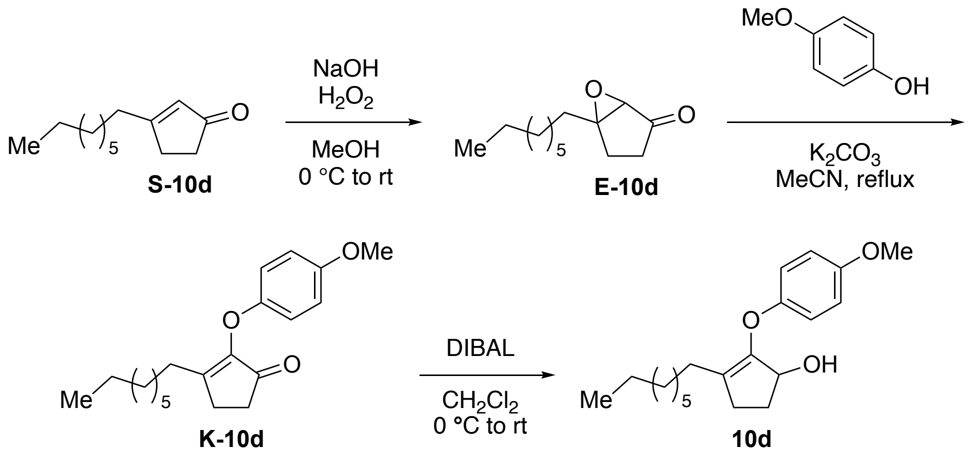

Epoxidation

Quantity: Starting material **S-10d** (1.25 g, 6.43 mmol); H_2O_2 (3.3 mL, 32.2 mmol, 30% in H_2O); NaOH (1.6 mL, 6.43 mmol, 4 M in H_2O); MeOH (12.9 mL); epoxide **E-10d** (0.732 g, crude, pale yellow oil).

Reaction Time: 25 min

Rf: 0.8 in 80:20 hexanes : ethyl acetate

Ring Opening

Quantity: Epoxide **E-10d** (0.732 g, crude); 4-methoxyphenol (0.643 g, 5.22 mmol); K_2CO_3 (0.577 g, 4.17 mmol); MeCN (13.8 mL); *O*-aryl enol ether **K-10d** (0.943 g, 2.95 mmol, 46% yield over two steps).

Reaction Time: 10 h

Rf: 0.5 in 80:20 hexanes : ethyl acetate

Column Chromatography: 80:20 hexanes : ethyl acetate

Reduction

Quantity: Starting material **K-10d** (943 mg, 2.98 mmol); DIBAL (3.0 mL, 3.28 mmol, 1.1 M in cyclohexane); CH_2Cl_2 (14.9 mL); α-hydroxy enol ether product **10d** (938 mg, 2.95 mmol, 99% yield, pale yellow liquid).

Reaction Time: 2 h

Rf: 0.6 in 80:20 hexanes : ethyl acetate

Column Chromatography: 80:20 hexanes : ethyl acetate

^{1}H NMR: (400 MHz, $CDCl_3$) δ = 6.93 – 6.88 (m, 2H), 6.83 – 6.79 (m, 2H), 4.73 (m, 1H), 3.77 (s, 3H), 2.45 (m, 1H), 2.31 – 2.20 (m, 2H), 2.11 (dd, J = 12.0, 4.0 Hz, 2H), 2.03 (dd, J = 12.0, 4.0 Hz, 2H), 1.82 – 1.73 (m, 2H), 1.42 – 1.35 (m, 2H), 1.30 – 1.23 (m, 10 H), 0.87 (t, J = 8.0 Hz, 3H).

^{13}C{1H} NMR: (100 MHz, $CDCl_3$) δ = 154.8, 150.8, 148.6, 129.0, 117.4, 114.6, 73.2, 55.6, 31.8, 30.1, 29.5, 29.3, 29.2, 28.5, 27.2, 26.5, 22.6, 14.1.

IR: f (cm^{-1}) = 3381, 3043, 2923, 2852, 1687, 1501, 1210, 1036, 827, 748.

HRMS (ESI-TOF): m/z $[M{-}OH]^+$ = 301.2168 calculated for $C_{20}H_{29}O_2$; found 301.2171.

Compound 10e

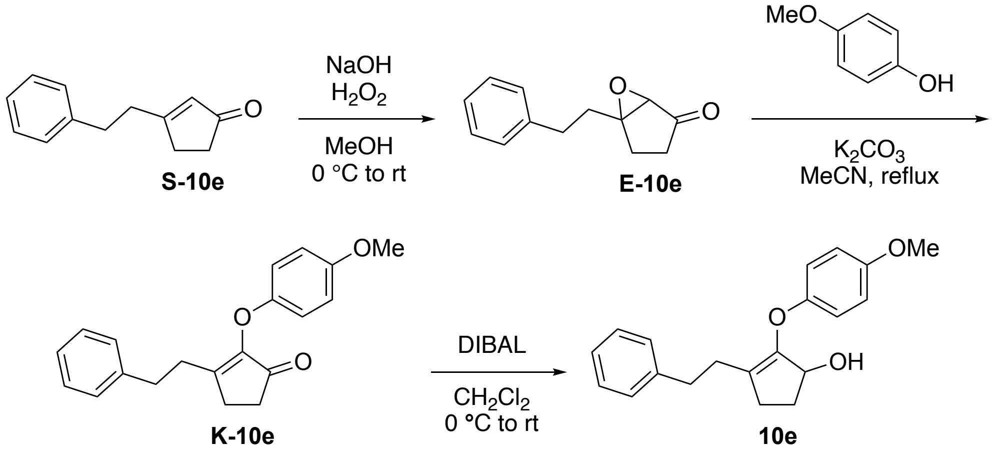

Epoxidation

Quantity: Starting material **S-10e** (0.495 g, 2.66 mmol); H_2O_2 (1.40 mL, 13.3 mmol, 30% in H_2O); NaOH (0.80 mL, 2.66 mmol, 4 M in H_2O); MeOH (15 mL); epoxide **E-10e** (0.538 g, crude, yellow oil).

Reaction Time: 32 min

Rf: 0.5 in 80:20 hexanes : ethyl acetate

Ring Opening

Quantity: Epoxide **E-10e** (0.293 g, crude); 4-methoxyphenol (0.270 g, 2.17 mmol); K_2CO_3 (0.240 g, 1.74 mmol); MeCN (5.8 mL); *O*-aryl enol ether **K-10e** (0.270 g, 0.876 mmol, 33% yield over two steps).

Reaction Time: 12 h

Rf: 0.2 in 80:20 hexanes : ethyl acetate

Column Chromatography: 70:30 hexanes : ethyl acetate

Reduction

Quantity: Starting material **K-10e** (268 mg, 0.869 mmol); DIBAL (0.90 mL, 0.96 mmol, 1.1 M in cyclohexane); CH_2Cl_2 (4.4 mL); α-hydroxy enol ether product **10e** (187 mg, 0.603 mmol, 69% yield, white solid).

Reaction Time: 57 min

Rf: 0.5 in 70:30 hexanes : ethyl acetate

Column Chromatography: 80:20 hexanes : ethyl acetate

^{1}H NMR: (400 MHz, $CDCl_3$) δ = 7.25 – 7.22 (m, 2H), 7.19 – 7.12 (m, 3H), 6.79 (s, 4H), 4.69 (s, 1H), 3.77 (s, 3H), 2.78 – 2.66 (m, 2H), 2.50 – 2.34 (m, 3H), 2.31 – 2.21 (m, 2H), 1.79 (m, 1H), 1.63 (d, J = 4.7 Hz, 1H).

^{13}C{1H} NMR: (100 MHz, $CDCl_3$) δ = 154.9, 150.6, 149.5, 141.7, 128.30, 128.29, 127.34, 125.9, 117.7, 114.6, 73.0, 55.7, 33.5, 30.1, 28.6, 28.4.

IR: *f* (cm^{-1}) = 3519, 3080, 3024, 2944, 2859, 1693, 1505, 1207, 1029, 825.

HRMS (ESI-TOF): *m/z* $[M{-}OH]^+$ = 293.1542 calculated for $C_{20}H_{21}O_2$; found 293.1548.

Compound 10f

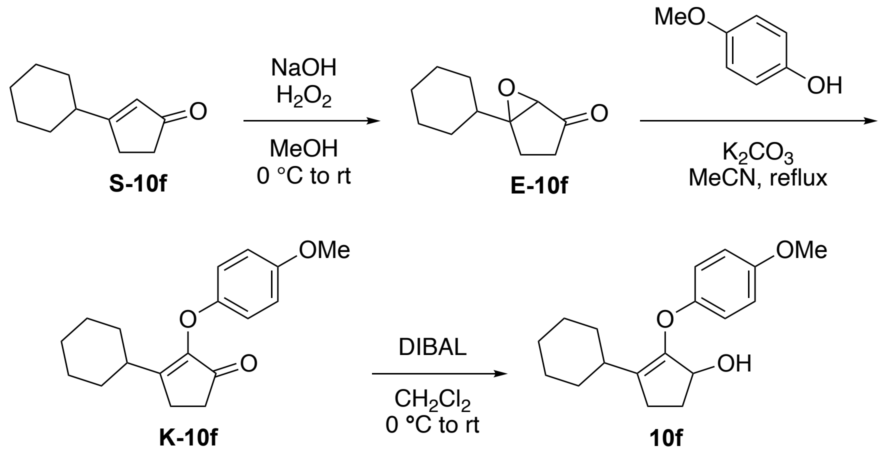

Epoxidation

Quantity: Starting material **S-10f** (0.750 g, 4.57 mmol); H_2O_2 (2.3 mL, 22.8 mmol, 30% in H_2O); NaOH (1.1 mL, 4.57 mmol, 4 M in H_2O); MeOH (9.1 mL); epoxide **E-10f** (0.510 g, crude, colorless oil).

Reaction Time: 35 min

Rf: 0.7 in 80:20 hexanes : ethyl acetate

Ring Opening

Quantity: Epoxide **E-10f** (0.119 g, crude); 4-methoxyphenol (0.123 g, 0.99 mmol); K_2CO_3 (0.109 g, 0.79 mmol); MeCN (2.6 mL); *O*-aryl enol ether **K-10f** (0.138 g, 0.48 mmol, 46% yield over two steps).

Reaction Time: 10 h

Rf: 0.5 in 80:20 hexanes : ethyl acetate

Column Chromatography: 80:20 hexanes : ethyl acetate

Reduction

Quantity: Starting material **K-10f** (138 mg, 0.482 mmol); DIBAL (0.48 mL, 0.53 mmol, 1.1 M in cyclohexane); CH_2Cl_2 (2.4 mL); α-hydroxy enol ether product **10f** (97 mg, 0.34 mmol, 70% yield, white solid).

Reaction Time: 90 min

Rf: 0.5 in 80:20 hexanes : ethyl acetate

Column Chromatography: 80:20 hexanes : ethyl acetate

^{1}H NMR: (400 MHz, $CDCl_3$) δ = 6.93 – 6.89 (m, 2H), 6.83 – 6.79 (m, 2H), 4.70 (m, 1H), 3.77 (s, 3H), 2.49 – 2.36 (m, 2H), 2.28 – 2.18 (m, 2H), 1.78 – 1.62 (m, 6H), 1.57 (m, 1H), 1.33 – 1.08 (m, 5H).

^{13}C{1H} NMR: (100 MHz, $CDCl_3$) δ = 154.8, 150.7, 147.1, 133.8, 117.4, 114.6, 72.9, 55.6, 35.8, 31.2, 30.4, 30.1, 26.2, 26.2, 26.0, 25.6.

IR: *f* (cm^{-1}) = 3404, 3004, 2924, 2851, 1682, 1501, 1210, 1036, 828, 749.

HRMS (ESI-TOF): *m/z* $[M{-}OH]^+$ = 271.1698 calculated for $C_{18}H_{23}O_2$; found 271.1696.

Compound 11a

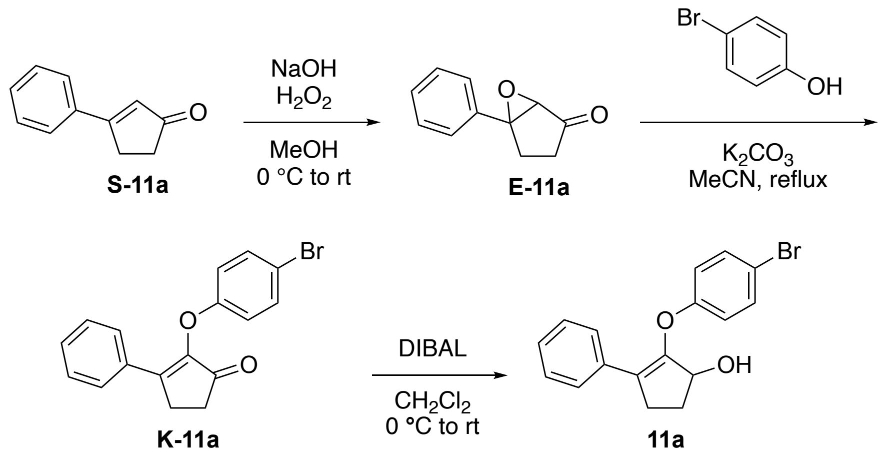

S-11a **E-11a** **K-11a** **11a**

Epoxidation

Quantity: Starting material **S-11a** (2.71 g, 17.1 mmol); H_2O_2 (8.8 mL, 85.7 mmol, 30% in H_2O); NaOH (4.3 mL, 17.1 mmol, 4 M in H_2O); MeOH (34 mL); epoxide **E-11a** (1.44 g, crude, yellow oil).

Reaction Time: 30 min

Rf: 0.6 in 70:30 hexanes : ethyl acetate

Ring Opening

Quantity: Epoxide **E-11a** (0.611 g, crude); 4-bromophenol (0.909 g, 5.26 mmol); K_2CO_3 (0.581g, 4.21 mmol); MeCN (14 mL); *O*-aryl enol ether **K-11a** (0.596 g, 1.81 mmol, 25% yield over two steps).

Reaction Time: 15 h

Rf: 0.6 in 70:30 hexanes : ethyl acetate

Column Chromatography: 60:40 hexanes : ethyl acetate

Reduction

Quantity: Starting material **K-11a** (0.590 g, 1.79 mmol); DIBAL (1.8 mL, 1.97 mmol, 1.1 M in cyclohexane); CH_2Cl_2 (9.0 mL); α-hydroxy enol ether product **11a** (489 mg, 1.48 mmol, 82% yield, white solid).

Reaction Time: 100 min

Rf: 0.4 in 90:10 CH_2Cl_2 : hexanes

Column Chromatography: CH_2Cl_2

^{1}H NMR: (400 MHz, $CDCl_3$) δ = 7.60 – 7.57 (m, 2H), 7.39 (ddd, J = 10.1, 3.0, 2.9 Hz, 2H), 7.33

– 7.29 (m, 2H), 7.24 (m, 1H), 6.93 (ddd, *J* = 10.1, 3.0, 2.8 Hz, 2H), 4.91 (m, 1H), 2.96 (dddd, *J* = 15.8, 8.9, 4.6, 2.2 Hz, 1H), 2.74 (ddd, *J* = 15.4, 8.6, 4.5 Hz, 1H), 2.42 (m, 1H) 1.93 (ddd, *J* = 17.6, 8.6, 4.1 Hz, 1H), 1.80 (d, *J* = 5.5 Hz 1H).

^{13}C{1H} NMR: (100 MHz, $CDCl_3$) δ = 154.9, 149.3, 133.8, 132.6, 128.3, 127.7, 127.1, 125.9, 118.8, 115.2, 73.6, 29.7, 28.4.

IR: *f* (cm^{-1}) = 3340, 3090, 2902, 1663, 1506, 1240, 1064, 820, 760.

HRMS (ESI-TOF): *m/z* [M−OH]$^+$ = 313.0228 calculated for $C_{17}H_{14}BrO$; found 313.0235.

Compound 11b

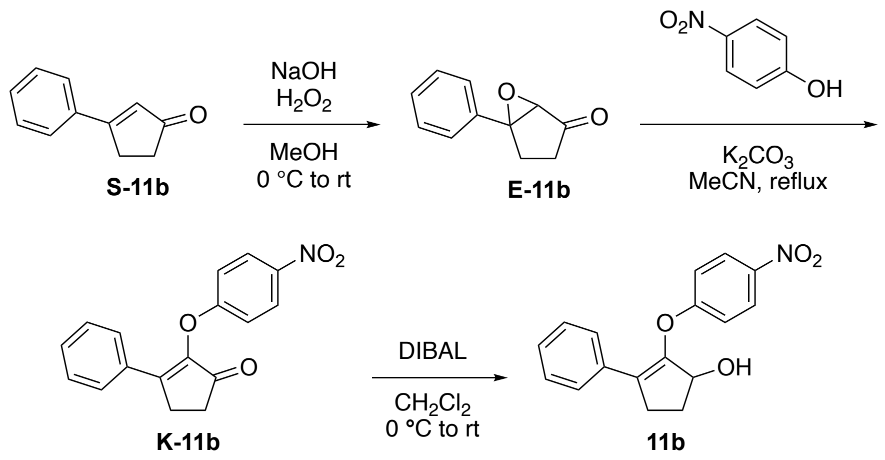

Epoxidation

Quantity: Starting material **S-11b** (1.50 g, 9.48 mmol); H_2O_2 (4.8 mL, 47.4 mmol, 30% in H_2O); NaOH (2.4 mL, 9.48 mmol, 4 M in H_2O); MeOH (19 mL); epoxide **E-11b** (0.601 g, crude, yellow oil).

Reaction Time: 30 min

Rf: 0.7 in 70:30 hexanes : ethyl acetate

Ring Opening

Quantity: Epoxide **E-11b** (0.601 g, crude); 4-nitrophenol (0.720 g, 5.17 mmol); K_2CO_3 (0.573g, 4.14 mmol); MeCN (13.8 mL); *O*-aryl enol ether **K-11b** (0.531 g, 1.80 mmol, 19% yield over two steps).

Reaction Time: 17 h

Rf: 0.4 in 70:30 hexanes : ethyl acetate

Column Chromatography: 70:30 hexanes : ethyl acetate

Reduction

Quantity: Starting material **K-11b** (524 mg, 1.77 mmol); DIBAL (1.8 mL, 1.95 mmol, 1.1 M in cyclohexane); CH_2Cl_2 (8.9 mL); α-hydroxy enol ether product **11b** (274 mg, 0.921 mmol, 52% yield, yellow oil).

Reaction Time: 1 h

Rf: 0.3 in CH_2Cl_2

Column Chromatography: 90 : 10 CH_2Cl_2 : ethyl acetate

^{1}H NMR: (400 MHz, $CDCl_3$) δ = 8.18 (ddd, *J* = 10.4, 2.8, 2.6 Hz, 2H), 7.52 – 7.49 (m, 2H), 7.32 – 7.22 (m, 3H), 7.12 (ddd, *J* = 10.4, 2.8, 2.6 Hz, 2H), 4.98 (m, 1H), 3.01 (dddd, *J* = 15.6, 8.9, 4.4, 2.3 Hz, 1H), 2.79 (dddd, *J* = 15.7, 8.7, 4.8, 1.0 Hz, 1H), 2.51 (dddd, *J* = 14.0, 8.6, 7.7, 4.4 Hz, 1H), 1.98 (dddd, *J* = 13.8, 8.8, 4.8, 4.0 Hz, 1H), 1.83 (d, *J* = 5.9 Hz, 1H).

^{13}C{1H} NMR: (100 MHz, $CDCl_3$) δ = 161.2, 148.0, 142.8, 133.1, 128.5, 128.2, 128.0, 127.1, 125.9, 116.5, 74.3, 29.9, 28.5.

IR: *f* (cm^{-1}) = 3554, 3096, 2847, 1592, 1488, 1246, 1042, 830.

HRMS (ESI-TOF): *m/z* $[M+H]^+$ = 298.1079 calculated for $C_{17}H_{16}NO_4$; found 298.1079.

Compound 11c

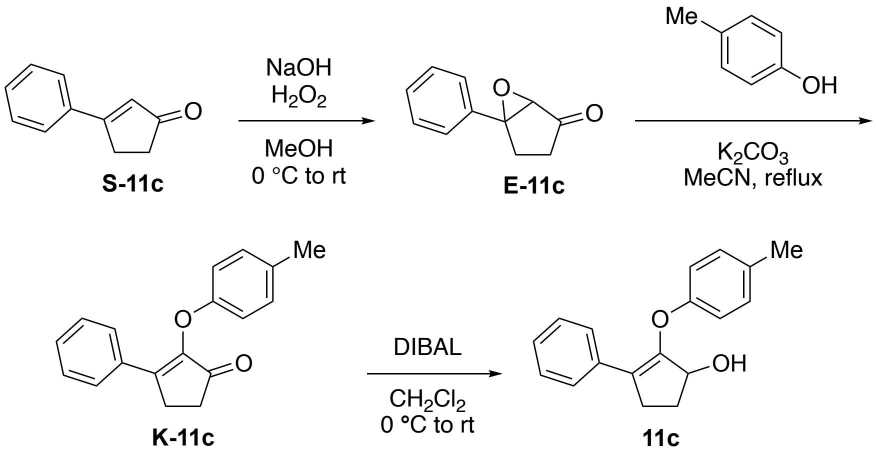

Epoxidation

Quantity: Starting material **S-11c** (0.620 g, 3.92 mmol); H_2O_2 (2.0 mL, 19.6 mmol, 30% in H_2O); NaOH (0.98 mL, 3.92 mmol, 4 M in H_2O); MeOH (7.8 mL); epoxide **E-11c** (0.375 g, crude, yellow oil).

Reaction Time: 60 min

Rf: 0.6 in 80:20 hexanes : ethyl acetate

Ring Opening

Quantity: Epoxide **E-11c** (0.375 g, crude); 4-methylphenol (0.340 g, 3.23 mmol); K_2CO_3 (0.357 g, 2.58 mmol); MeCN (8.6 mL); *O*-aryl enol ether **K-11c** (0.387 g, 1.46 mmol, 37% yield over two steps).

Reaction Time: 12 h

Rf: 0.4 in 80:20 hexanes : ethyl acetate

Column Chromatography: 90:10 hexanes : ethyl acetate

Reduction

Quantity: Starting material **K-11c** (0.387 mg, 1.46 mmol); DIBAL (2.2 mL, 2.20 mmol, 1.0 M in cyclohexane); CH_2Cl_2 (7.3 mL); α-hydroxy enol ether product **11c** (373 mg, 1.40 mmol, 96% yield, pale yellow solid).

Reaction Time: 3 h

Rf: 0.6 in 80:20 hexanes : ethyl acetate

Column Chromatography: 85:15 hexanes : ethyl acetate

^{1}H NMR: (400 MHz, $CDCl_3$) δ = 7.58 – 7.55 (m, 2H), 7.24 – 7.20 (m, 2H), 7.13 (m, 1H), 7.01 (d, *J* = 8.1 Hz, 2H), 6.88 – 6.84 (m, 2H), 4.81 (m, 1H), 2.85 (dddd, *J* = 15.6, 8.9, 4.7, 2.1 Hz, 1H), 2.62 (ddd, *J* = 15.3, 8.6, 4.4 Hz, 1H), 2.28 (dtd, *J* = 13.7, 8.1, 4.7 Hz, 1H), 2.2 (s, 3H), 1.82 (ddt, *J* = 13.3, 8.5, 4.1 Hz, 1H), 1.76 (d, *J* = 5.2 Hz, 1H).

^{13}C{1H} NMR: (100 MHz, $CDCl_3$) δ = 153.3, 150.3, 134.2, 132.4, 130.1, 128.2, 127.3, 127.1, 124.3, 117.2, 73.4, 29.5, 28.3, 20.6.

IR: *f* (cm^{-1}) = 3335, 3055, 3027, 2924, 1652, 1607, 1504, 1221, 1045, 819.

HRMS (ESI-TOF): *m/z* $[M{-}OH]^+$ = 249.1279 calculated for $C_{18}H_{17}O$; found 249.1286.

Compound 11d

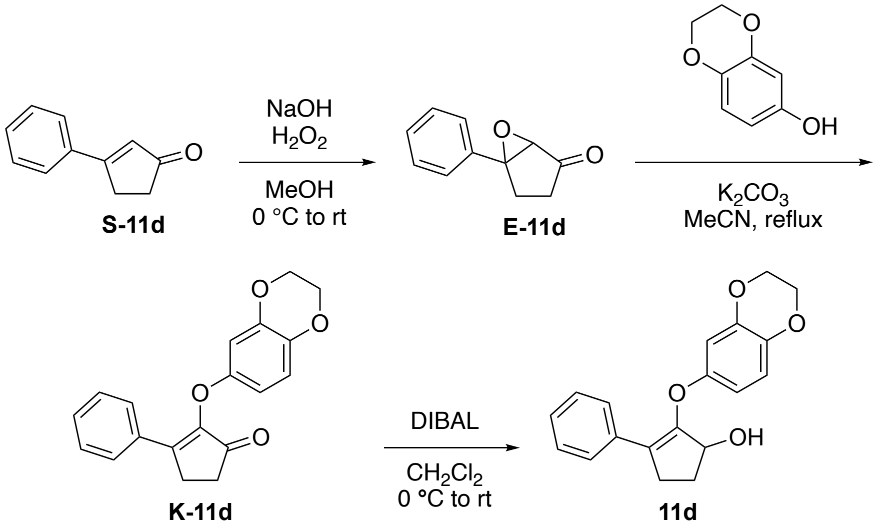

Epoxidation

Quantity: Starting material **S-11d** (2.07 g, 13.1 mmol); H_2O_2 (6.7 mL, 65.4 mmol, 30% in H_2O); NaOH (3.3 mL, 13.1 mmol, 4 M in H_2O); MeOH (26 mL); epoxide **E-11d** (1.18 g, crude, yellow oil).

Reaction Time: 60 min

Rf: 0.6 in 80:20 hexanes : ethyl acetate

Ring Opening

Quantity: Epoxide **E-11d** (0.586 g, crude); 6-hydroxy-1,4-benzodioxane (0.768 g, 5.04 mmol); K_2CO_3 (0.558 g, 4.03 mmol); MeCN (33 mL); *O*-aryl enol ether **K-11d** (0.644 g, 2.08 mmol, 32% yield over two steps).

Reaction Time: 16 h

Rf: 0.4 in 70:30 hexanes : ethyl acetate

Column Chromatography: 80:20 hexanes : ethyl acetate

Reduction

Quantity: Starting material **K-11d** (145 mg, 0.470 mmol); DIBAL (0.64 mL, 0.70 mmol, 1.1 M in cyclohexane); CH_2Cl_2 (2.4 mL); α-hydroxy enol ether product **11d** (135 mg, 0.435 mmol, 93%, yellow liquid).

Reaction Time: 7 h

Rf: 0.2 in 70:30 hexanes : ethyl acetate

Column Chromatography: 70:30 hexanes : ethyl acetate

^{1}H NMR: (400 MHz, $CDCl_3$) δ = 7.58 – 7.55 (m, 2H), 7.25 – 7.21 (m, 2H), 7.15 (m, 1H), 6.7 (d, *J* = 8.6 Hz, 1H), 6.53 – 6.47 (m, 2H), 4.82 (dd, *J* = 6.9, 3.3 Hz, 1H), 4.16 – 4.11 (m, 4H), 2.84 (dddd, *J* = 15.5, 8.9, 4.7, 2.1 Hz, 1H), 2.62 (ddd, *J* = 15.2, 8.6, 4.4 Hz, 1H), 2.29 (dddd, *J* = 13.6, 8.7, 7.7, 4.7 Hz, 1H), 1.82 (ddt, *J* = 12.6, 8.4, 4.0 Hz, 1H), 1.74 (m, 1H).

^{13}C{1H} NMR: (100 MHz, $CDCl_3$) δ = 150.4, 149.6, 143.9, 139.5, 134.2, 128.2, 127.4, 127.1, 124.2, 117.6, 110.5, 106.6, 73.4, 64.5, 64.1, 29.5, 28.3.

IR: *f* (cm^{-1}) = 3366, 3055, 3010, 2931, 1651, 1597, 1495, 1208, 1064, 749.

HRMS (ESI-TOF): *m/z* $[M{-}OH]^+$ = 293.1178 calculated for $C_{19}H_{17}O_3$; found 293.1192.

Compound 11e

The preparation of this compound is described in the following article:

Dange, N. S.; Stepherson, J. R.; Ayala, C. E.; Fronczek, F. R.; Kartika, R. "Cooperative Benzylic-Oxyallylic Stabilized Cations: Regioselective Construction of α-Quaternary Centers in Ketone-Derived Compounds." *Chem. Sci.* **2015,** *6*, 6312-6319.

Compound 15a

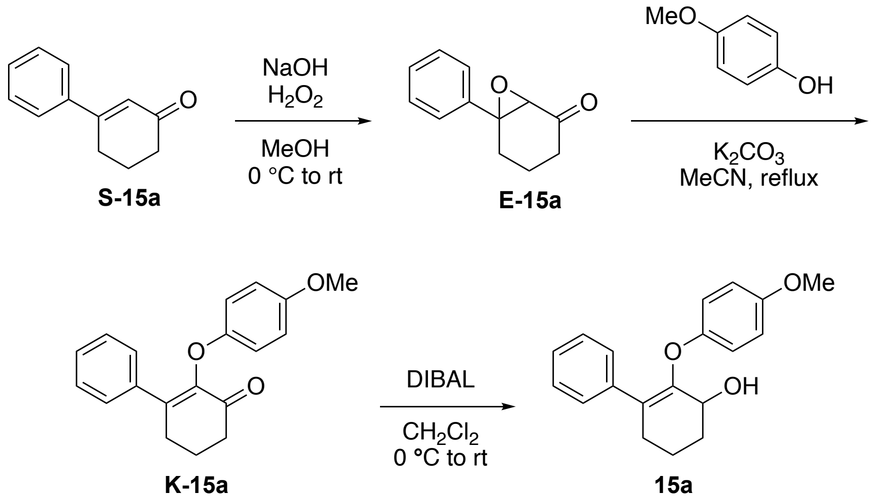

Epoxidation

Quantity: Starting material **S-15a** (2.00 g, 11.6 mmol); H_2O_2 (5.9 mL, 58.1 mmol, 30% in H_2O); NaOH (2.9 mL, 11.6 mmol, 4 M in H_2O); MeOH (23 mL); epoxide **E-15a** (1.54 g, crude, white solid).

Reaction Time: 2.7 h

Rf: 0.6 in 80:20 hexanes : ethyl acetate

Ring Opening

Quantity: Epoxide **E-15a** (1.54 g, crude); 4-methoxyphenol (1.52 g, 12.3 mmol); K_2CO_3 (1.36 g, 9.8 mmol); MeCN (33 mL); *O*-aryl enol ether **K-15a** (1.80 g, 6.1 mmol, 53% yield over two steps).

Reaction Time: 15 h

Rf: 0.4 in 80:20 hexanes : ethyl acetate

Column Chromatography: 80:20 hexanes : ethyl acetate

Reduction

Quantity: Starting material **K-15a** (1.80 g, 6.12 mmol); DIBAL (6.1 mL, 6.73 mmol, 1.1 M in cyclohexane); CH_2Cl_2 (30.6 mL); α-hydroxy enol ether product **15a** (1.54 g, 5.21 mmol, 85% yield, yellow liquid).

Reaction Time: 3 h

Rf: 0.3 in 80:20 hexanes : ethyl acetate.

Column Chromatography: 85:15 hexanes : ethyl acetate

^{1}H NMR: (400 MHz, $CDCl_3$) δ = 7.30 – 7.27 (m, 2H), 7.18 – 7.14 (m, 2H), 7.08 (m, 1H), 6.77 – 6.72 (m, 2H), 6.68 – 6.64 (m, 2H), 4.31 (q, J = 4.2, 3.8 Hz, 1H), 3.64 (s, 3H), 2.51 – 2.37 (m, 2H), 2.02 (d, J = 3.5 Hz, 1H), 1.92 – 1.82 (m, 3H), 1.70 (m, 1H).

^{13}C{1H} NMR: (100 MHz, $CDCl_3$) δ = 154.6, 150.3, 146.9, 138.5, 127.9, 127.7, 127.0, 126.8, 117.5, 114.6, 64.6, 55.6, 31.5, 30.4, 18.5.

IR: f (cm^{-1}) = 3412, 3055, 3002, 2935, 1660, 1599, 1500, 1203, 830, 753.

HRMS (ESI-TOF): *m/z* $[M-OH]^+$ = 279.1385 calculated for $C_{19}H_{19}O_2$; found 279.1388

X-Ray Crystallography Data

Compound 8

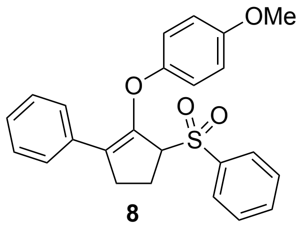

8

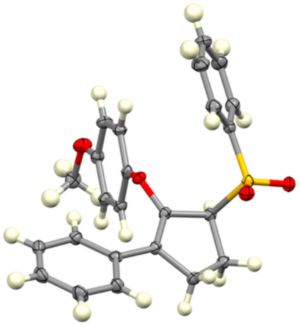

Sample Name: CCDC 2517777 / JAB1025C
Crystal Growth: Slow evaporation using a mixture of hexanes and dichloromethane

Crystal data

$C_{24}H_{22}O_4S$	*F*(000) = 856
M_r = 406.47	D_x = 1.360 Mg m^{-3}
Monoclinic, $P2_1/n$	Cu *K*a radiation, l = 1.54184 Å
a = 11.2484 (4) Å	Cell parameters from 9859 reflections
b = 10.6027 (4) Å	q = 5.0–79.4°
c = 16.9357 (6) Å	m = 1.69 mm^{-1}
b = 100.718 (2)°	*T* = 100 K
V = 1984.57 (13) $Å^3$	Plate, colourless
Z = 4	0.13 × 0.09 × 0.02 mm

Data collection

Bruker D8 Venture DUO with Photon III C14 diffractometer	3805 reflections with *I* > 2s(*I*)
Radiation source: ImS 3.0 microfocus	R_{int} = 0.053
f and w scans	q_{max} = 79.8°, q_{min} = 4.4°
Absorption correction: multi-scan *SADABS* (Krause *et al.*, 2015)	*h* = -14 - 14
T_{min} = 0.850, T_{max} = 0.967	*k* = -13 - 12
44663 measured reflections	*l* = -21 - 21
4276 independent reflections	

Refinement

Refinement on F^2	Hydrogen site location: inferred from neighbouring sites
Least-squares matrix: full	H-atom parameters constrained
$R[F^2 > 2s(F^2)] = 0.033$	$w = 1/[s^2(F_o^2) + (0.0338P)^2 + 1.1047P]$ where $P = (F_o^2 + 2F_c^2)/3$
$wR(F^2) = 0.083$	$(D/s)_{max} = 0.001$
$S = 1.04$	$Dr_{max} = 0.35$ e Å^{-3}
4276 reflections	$Dr_{min} = -0.38$ e Å^{-3}
264 parameters	Extinction correction: *SHELXL2019*/1 (Sheldrick 2019), $Fc^* = kFc[1+0.001xFc^2l^3/\sin(2q)]^{-1/4}$
0 restraints	Extinction coefficient: 0.00078 (14)

Fractional atomic coordinates and isotropic or equivalent isotropic displacement parameters (Å^2) for (JAB1025C)

	x	*y*	*z*	$U_{iso}*/U_{eq}$
S1	0.25683 (3)	0.40780 (3)	0.46139 (2)	0.01608 (9)
O1	0.21115 (8)	0.33001 (9)	0.26295 (5)	0.01672 (19)
O2	0.36555 (8)	0.33577 (9)	0.48824 (6)	0.0209 (2)
O3	0.22252 (9)	0.50247 (9)	0.51450 (6)	0.0222 (2)
O4	-0.16612 (9)	0.55884 (10)	0.05580 (6)	0.0244 (2)
C1	0.30109 (11)	0.40454 (12)	0.30595 (7)	0.0159 (3)
C2	0.27184 (11)	0.49020 (12)	0.37023 (7)	0.0163 (3)
H2	0.198334	0.542075	0.349725	0.020*
C3	0.38566 (12)	0.57337 (13)	0.38934 (8)	0.0190 (3)
H3A	0.372844	0.654369	0.359904	0.023*
H3B	0.406646	0.591161	0.447660	0.023*
C4	0.48615 (12)	0.49625 (13)	0.36158 (8)	0.0186 (3)
H4A	0.539955	0.551422	0.336749	0.022*
H4B	0.535288	0.450406	0.407217	0.022*
C5	0.41825 (12)	0.40530 (12)	0.30061 (7)	0.0163 (3)
C6	0.48051 (12)	0.33033 (12)	0.24766 (8)	0.0174 (3)
C7	0.41998 (12)	0.27713 (13)	0.17571 (8)	0.0202 (3)
H7	0.335146	0.288665	0.159821	0.024*
C8	0.48267 (13)	0.20771 (14)	0.12738 (8)	0.0227 (3)
H8	0.441007	0.175275	0.077572	0.027*
C9	0.60583 (14)	0.18516 (15)	0.15112 (9)	0.0267 (3)
H9	0.647737	0.134499	0.118980	0.032*
C10	0.66670 (14)	0.23732 (16)	0.22208 (9)	0.0284 (3)

H10	0.750906	0.222383	0.238744	0.034*
C11	0.60545 (12)	0.31152 (15)	0.26925 (8)	0.0231 (3)
H11	0.648953	0.349838	0.316638	0.028*
C12	0.13504 (12)	0.30089 (13)	0.43640 (8)	0.0180 (3)
C13	0.15874 (13)	0.17241 (14)	0.43886 (9)	0.0238 (3)
H13	0.239487	0.142139	0.452061	0.029*
C14	0.06264 (15)	0.08907 (15)	0.42175 (10)	0.0316 (3)
H14	0.077420	0.000812	0.422948	0.038*
C15	-0.05554 (14)	0.13397 (16)	0.40279 (10)	0.0308 (3)
H15	-0.120997	0.076271	0.390552	0.037*
C16	-0.07807 (13)	0.26271 (16)	0.40171 (9)	0.0272 (3)
H16	-0.158938	0.292870	0.389441	0.033*
C17	0.01724 (12)	0.34744 (14)	0.41850 (8)	0.0222 (3)
H17	0.002462	0.435685	0.417816	0.027*
C18	0.11857 (11)	0.39685 (12)	0.21300 (7)	0.0160 (3)
C19	0.13958 (12)	0.50844 (13)	0.17611 (8)	0.0182 (3)
H19	0.217790	0.545583	0.186811	0.022*
C20	0.04579 (12)	0.56695 (13)	0.12294 (8)	0.0190 (3)
H20	0.059579	0.644274	0.097771	0.023*
C21	-0.06765 (12)	0.51087 (13)	0.10733 (8)	0.0184 (3)
C22	-0.08827 (12)	0.39840 (13)	0.14529 (8)	0.0210 (3)
H22	-0.166136	0.360518	0.134399	0.025*
C23	0.00446 (12)	0.34174 (13)	0.19883 (8)	0.0188 (3)
H23	-0.009801	0.266026	0.225580	0.023*
C24	-0.14695 (13)	0.67085 (14)	0.01353 (9)	0.0232 (3)
H24A	-0.223016	0.696473	-0.020906	0.035*
H24B	-0.085904	0.654968	-0.019706	0.035*
H24C	-0.118728	0.738211	0.052098	0.035*

Atomic displacement parameters (Å^2) for (JAB1025C)

	U^{11}	U^{22}	U^{33}	U^{12}	U^{13}	U^{23}
S1	0.01649 (16)	0.01691 (16)	0.01441 (15)	-0.00042 (11)	0.00176 (11)	-0.00022 (11)
O1	0.0156 (4)	0.0160 (4)	0.0169 (4)	-0.0008 (3)	-0.0012 (3)	0.0003 (3)
O2	0.0177 (5)	0.0227 (5)	0.0205 (5)	0.0008 (4)	-0.0005 (4)	0.0034 (4)
O3	0.0269 (5)	0.0217 (5)	0.0189 (5)	-0.0017 (4)	0.0064 (4)	-0.0038 (4)

O4	0.0167 (5)	0.0253 (5)	0.0285 (5)	0.0006 (4)	-0.0028 (4)	0.0068 (4)
C1	0.0171 (6)	0.0154 (6)	0.0142 (6)	-0.0005 (5)	0.0000 (5)	0.0010 (5)
C2	0.0169 (6)	0.0158 (6)	0.0155 (6)	0.0007 (5)	0.0016 (5)	0.0013 (5)
C3	0.0214 (7)	0.0169 (6)	0.0186 (6)	-0.0021 (5)	0.0031 (5)	-0.0014 (5)
C4	0.0178 (6)	0.0192 (6)	0.0180 (6)	-0.0014 (5)	0.0009 (5)	-0.0013 (5)
C5	0.0177 (6)	0.0164 (6)	0.0137 (6)	-0.0004 (5)	-0.0001 (5)	0.0019 (5)
C6	0.0182 (6)	0.0174 (6)	0.0171 (6)	-0.0013 (5)	0.0044 (5)	0.0025 (5)
C7	0.0201 (6)	0.0212 (7)	0.0190 (6)	-0.0019 (5)	0.0029 (5)	0.0002 (5)
C8	0.0280 (7)	0.0231 (7)	0.0180 (6)	-0.0034 (6)	0.0066 (5)	-0.0033 (5)
C9	0.0301 (8)	0.0276 (7)	0.0259 (7)	0.0012 (6)	0.0146 (6)	-0.0016 (6)
C10	0.0207 (7)	0.0389 (9)	0.0269 (7)	0.0035 (6)	0.0074 (6)	0.0007 (6)
C11	0.0184 (7)	0.0318 (8)	0.0186 (6)	0.0003 (6)	0.0027 (5)	-0.0008 (6)
C12	0.0187 (6)	0.0207 (6)	0.0151 (6)	-0.0016 (5)	0.0045 (5)	-0.0003 (5)
C13	0.0224 (7)	0.0215 (7)	0.0291 (7)	0.0006 (5)	0.0086 (6)	-0.0012 (6)
C14	0.0356 (9)	0.0206 (7)	0.0420 (9)	-0.0054 (6)	0.0161 (7)	-0.0051 (6)
C15	0.0259 (8)	0.0349 (9)	0.0324 (8)	-0.0129 (6)	0.0071 (6)	-0.0064 (6)
C16	0.0174 (7)	0.0399 (9)	0.0234 (7)	-0.0033 (6)	0.0012 (5)	-0.0002 (6)
C17	0.0208 (7)	0.0244 (7)	0.0211 (7)	0.0012 (5)	0.0027 (5)	0.0012 (5)
C18	0.0156 (6)	0.0178 (6)	0.0138 (6)	0.0012 (5)	0.0006 (5)	-0.0015 (5)
C19	0.0150 (6)	0.0204 (6)	0.0183 (6)	-0.0030 (5)	0.0010 (5)	-0.0009 (5)
C20	0.0194 (6)	0.0183 (6)	0.0189 (6)	-0.0018 (5)	0.0027 (5)	0.0013 (5)
C21	0.0162 (6)	0.0209 (7)	0.0172 (6)	0.0020 (5)	0.0007 (5)	-0.0006 (5)
C22	0.0158 (6)	0.0218 (7)	0.0246 (7)	-0.0033	0.0017 (5)	0.0002 (5)

				(5)		
C23	0.0190 (6)	0.0174 (6)	0.0199 (6)	-0.0022 (5)	0.0038 (5)	0.0006 (5)
C24	0.0236 (7)	0.0222 (7)	0.0221 (7)	0.0037 (5)	-0.0006 (5)	0.0036 (5)

Geometric parameters (Å, º) for (JAB1025C)

S1—O2	1.4417 (10)	C10—C11	1.392 (2)
S1—O3	1.4473 (10)	C10—H10	0.9500
S1—C12	1.7679 (14)	C11—H11	0.9500
S1—C2	1.8088 (13)	C12—C13	1.387 (2)
O1—C1	1.3801 (15)	C12—C17	1.3935 (19)
O1—C18	1.4052 (15)	C13—C14	1.384 (2)
O4—C21	1.3738 (16)	C13—H13	0.9500
O4—C24	1.4237 (17)	C14—C15	1.392 (2)
C1—C5	1.3376 (18)	C14—H14	0.9500
C1—C2	1.5004 (17)	C15—C16	1.388 (2)
C2—C3	1.5386 (18)	C15—H15	0.9500
C2—H2	1.0000	C16—C17	1.387 (2)
C3—C4	1.5379 (18)	C16—H16	0.9500
C3—H3A	0.9900	C17—H17	0.9500
C3—H3B	0.9900	C18—C19	1.3788 (19)
C4—C5	1.5119 (17)	C18—C23	1.3900 (18)
C4—H4A	0.9900	C19—C20	1.3983 (18)
C4—H4B	0.9900	C19—H19	0.9500
C5—C6	1.4698 (18)	C20—C21	1.3879 (18)
C6—C11	1.3991 (19)	C20—H20	0.9500
C6—C7	1.3992 (18)	C21—C22	1.3945 (19)
C7—C8	1.3869 (19)	C22—C23	1.3853 (19)
C7—H7	0.9500	C22—H22	0.9500
C8—C9	1.390 (2)	C23—H23	0.9500
C8—H8	0.9500	C24—H24A	0.9800
C9—C10	1.383 (2)	C24—H24B	0.9800
C9—H9	0.9500	C24—H24C	0.9800
O2—S1—O3	118.68 (6)	C11—C10—H10	119.8
O2—S1—C12	108.06 (6)	C10—C11—C6	120.73 (13)
O3—S1—C12	108.00 (6)	C10—C11—H11	119.6
O2—S1—C2	108.47 (6)	C6—C11—H11	119.6

O3—S1—C2	105.87 (6)	C13—C12—C17	121.60 (13)
C12—S1—C2	107.25 (6)	C13—C12—S1	119.11 (11)
C1—O1—C18	114.67 (10)	C17—C12—S1	119.22 (11)
C21—O4—C24	116.65 (11)	C14—C13—C12	118.83 (14)
C5—C1—O1	127.05 (12)	C14—C13—H13	120.6
C5—C1—C2	113.40 (11)	C12—C13—H13	120.6
O1—C1—C2	119.43 (11)	C13—C14—C15	120.33 (15)
C1—C2—C3	102.32 (10)	C13—C14—H14	119.8
C1—C2—S1	113.25 (9)	C15—C14—H14	119.8
C3—C2—S1	107.66 (9)	C16—C15—C14	120.23 (14)
C1—C2—H2	111.1	C16—C15—H15	119.9
C3—C2—H2	111.1	C14—C15—H15	119.9
S1—C2—H2	111.1	C17—C16—C15	120.13 (14)
C4—C3—C2	105.14 (10)	C17—C16—H16	119.9
C4—C3—H3A	110.7	C15—C16—H16	119.9
C2—C3—H3A	110.7	C16—C17—C12	118.86 (14)
C4—C3—H3B	110.7	C16—C17—H17	120.6
C2—C3—H3B	110.7	C12—C17—H17	120.6
H3A—C3—H3B	108.8	C19—C18—C23	120.87 (12)
C5—C4—C3	103.96 (10)	C19—C18—O1	122.36 (11)
C5—C4—H4A	111.0	C23—C18—O1	116.69 (11)
C3—C4—H4A	111.0	C18—C19—C20	120.01 (12)
C5—C4—H4B	111.0	C18—C19—H19	120.0
C3—C4—H4B	111.0	C20—C19—H19	120.0
H4A—C4—H4B	109.0	C21—C20—C19	119.31 (12)
C1—C5—C6	128.70 (12)	C21—C20—H20	120.3
C1—C5—C4	109.67 (11)	C19—C20—H20	120.3
C6—C5—C4	121.61 (11)	O4—C21—C20	124.51 (12)
C11—C6—C7	118.20 (12)	O4—C21—C22	115.20 (12)
C11—C6—C5	119.15 (12)	C20—C21—C22	120.29 (12)
C7—C6—C5	122.65 (12)	C23—C22—C21	120.21 (12)
C8—C7—C6	120.63 (13)	C23—C22—H22	119.9
C8—C7—H7	119.7	C21—C22—H22	119.9
C6—C7—H7	119.7	C22—C23—C18	119.30 (12)
C7—C8—C9	120.63 (13)	C22—C23—H23	120.3
C7—C8—H8	119.7	C18—C23—H23	120.3
C9—C8—H8	119.7	O4—C24—H24A	109.5
C10—C9—C8	119.23 (13)	O4—C24—H24B	109.5
C10—C9—H9	120.4	H24A—C24—H24B	109.5

C8—C9—H9	120.4	O4—C24—H24C	109.5
C9—C10—C11	120.46 (14)	H24A—C24—H24C	109.5
C9—C10—H10	119.8	H24B—C24—H24C	109.5
C18—O1—C1—C5	-117.41 (14)	C7—C6—C11—C10	-2.7 (2)
C18—O1—C1—C2	66.82 (14)	C5—C6—C11—C10	177.67 (13)
C5—C1—C2—C3	15.10 (14)	O2—S1—C12—C13	-5.10 (13)
O1—C1—C2—C3	-168.57 (11)	O3—S1—C12—C13	-134.65 (11)
C5—C1—C2—S1	-100.48 (12)	C2—S1—C12—C13	111.65 (11)
O1—C1—C2—S1	75.84 (13)	O2—S1—C12—C17	171.83 (10)
O2—S1—C2—C1	54.64 (10)	O3—S1—C12—C17	42.28 (12)
O3—S1—C2—C1	-176.98 (9)	C2—S1—C12—C17	-71.42 (12)
C12—S1—C2—C1	-61.84 (10)	C17—C12—C13—C14	1.1 (2)
O2—S1—C2—C3	-57.73 (10)	S1—C12—C13—C14	177.94 (11)
O3—S1—C2—C3	70.66 (10)	C12—C13—C14—C15	-0.3 (2)
C12—S1—C2—C3	-174.21 (9)	C13—C14—C15—C16	-0.7 (2)
C1—C2—C3—C4	-22.37 (13)	C14—C15—C16—C17	0.8 (2)
S1—C2—C3—C4	97.21 (10)	C15—C16—C17—C12	-0.1 (2)
C2—C3—C4—C5	22.25 (13)	C13—C12—C17—C16	-0.9 (2)
O1—C1—C5—C6	1.2 (2)	S1—C12—C17—C16	-177.77 (11)
C2—C1—C5—C6	177.21 (12)	C1—O1—C18—C19	32.65 (16)
O1—C1—C5—C4	-176.99 (11)	C1—O1—C18—C23	-150.58 (11)
C2—C1—C5—C4	-1.00 (15)	C23—C18—C19—C20	-0.7 (2)
C3—C4—C5—C1	-13.65 (14)	O1—C18—C19—C20	175.91 (12)
C3—C4—C5—C6	167.99 (11)	C18—C19—C20—C21	-0.6 (2)
C1—C5—C6—C11	-157.85 (14)	C24—O4—C21—C20	2.88 (19)
C4—C5—C6—C11	20.17 (19)	C24—O4—C21—C22	-177.50 (12)
C1—C5—C6—C7	22.6 (2)	C19—C20—C21—	-179.38 (12)

		O4	
C4—C5—C6—C7	-159.41 (13)	C19—C20—C21—C22	1.0 (2)
C11—C6—C7—C8	0.0 (2)	O4—C21—C22—C23	-179.70 (12)
C5—C6—C7—C8	179.59 (12)	C20—C21—C22—C23	-0.1 (2)
C6—C7—C8—C9	2.7 (2)	C21—C22—C23—C18	-1.3 (2)
C7—C8—C9—C10	-2.8 (2)	C19—C18—C23—C22	1.7 (2)
C8—C9—C10—C11	0.0 (2)	O1—C18—C23—C22	-175.15 (12)
C9—C10—C11—C6	2.8 (2)		

Compound 13a

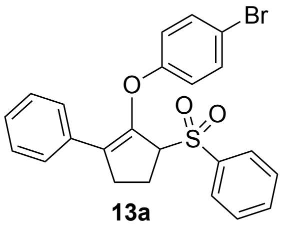

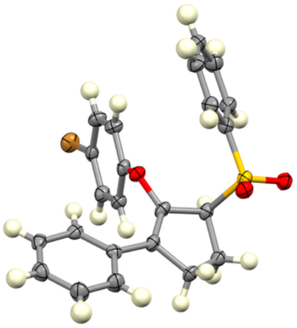

Sample Name: CCDC 2517778 / Adebanji3
Crystal Growth: Slow evaporation using a mixture of hexanes and dichloromethane

Crystal data

$C_{23}H_{19}BrO_3S$	$F(000) = 928$
$M_r = 455.35$	$D_x = 1.514$ Mg m^{-3}
Monoclinic, $P2_1/n$	Cu *K*a radiation, l = 1.54184 Å
$a = 11.6299$ (6) Å	Cell parameters from 3108 reflections
$b = 10.6231$ (5) Å	q = 4.3–73.6°
$c = 16.5611$ (8) Å	m = 3.96 mm^{-1}
b = 102.427 (4)°	$T = 100$ K
$V = 1998.11$ (17) Å^3	Fragment, colourless
$Z = 4$	0.03 × 0.03 × 0.01 mm

Data collection

Bruker D8 Venture DUO with Photon III C14 diffractometer	2937 reflections with $I > 2s(I)$
Radiation source: ImS 3.0 microfocus	$R_{int} = 0.140$
f and w scans	$q_{max} = 74.7°$, $q_{min} = 4.3°$
Absorption correction: multi-scan *SADABS* (Krause *et al.*, 2015)	$h = -14 - 14$
$T_{min} = 0.770$, $T_{max} = 0.962$	$k = -13 - 13$
34263 measured reflections	$l = -20 - 20$
4072 independent reflections	

Refinement

Refinement on F^2	0 restraints
Least-squares matrix: full	Hydrogen site location: inferred from neighbouring sites
$R[F^2 > 2s(F^2)] = 0.049$	H-atom parameters constrained
$wR(F^2) = 0.123$	$w = 1/[s^2(F_o^2) + (0.0508P)^2 + 2.6243P]$ where $P = (F_o^2 + 2F_c^2)/3$
$S = 1.01$	$(D/s)_{max} = 0.001$
4072 reflections	$Dr_{max} = 0.51$ e Å^{-3}
253 parameters	$Dr_{min} = -0.80$ e Å^{-3}

Fractional atomic coordinates and isotropic or equivalent isotropic displacement parameters (Å^2) for (Adebanji3)

	x	*y*	*z*	U_{iso}*/U_{eq}
Br1	0.31701 (4)	0.62465 (4)	0.02226 (3)	0.03380 (15)
S1	0.75136 (9)	0.41487 (9)	0.46215 (6)	0.0250 (2)
O1	0.7037 (2)	0.3478 (2)	0.25707 (16)	0.0246 (6)
O2	0.7205 (3)	0.5053 (3)	0.51911 (17)	0.0300 (6)
O3	0.8540 (2)	0.3373 (3)	0.48956 (17)	0.0291 (6)
C1	0.7687 (4)	0.5013 (4)	0.3715 (2)	0.0255 (8)
H1	0.698566	0.555849	0.350270	0.031*
C2	0.7944 (3)	0.4181 (4)	0.3044 (2)	0.0244 (8)
C3	0.9074 (4)	0.4161 (4)	0.2997 (2)	0.0248 (8)
C4	0.9768 (4)	0.5014 (4)	0.3658 (2)	0.0266 (9)
H4A	1.031203	0.555750	0.342874	0.032*
H4B	1.022896	0.451728	0.412235	0.032*
C5	0.8817 (4)	0.5805 (4)	0.3941 (3)	0.0278 (9)
H5A	0.903366	0.595902	0.454443	0.033*
H5B	0.871254	0.662598	0.365104	0.033*
C6	0.6293 (4)	0.3146 (4)	0.4295 (2)	0.0256 (8)
C7	0.5165 (4)	0.3661 (4)	0.4097 (2)	0.0288 (9)
H7	0.505258	0.454374	0.412944	0.035*
C8	0.4214 (4)	0.2868 (4)	0.3855 (3)	0.0354 (10)
H8	0.344010	0.320514	0.372033	0.042*
C9	0.4385 (4)	0.1581 (5)	0.3807 (3)	0.0392 (11)
H9	0.372858	0.104156	0.362548	0.047*
C10	0.5499 (4)	0.1083 (4)	0.4022 (3)	0.0384 (11)
H10	0.560553	0.019754	0.400589	0.046*
C11	0.6470 (4)	0.1861 (4)	0.4260 (3)	0.0315 (9)

H11	0.724150	0.151772	0.439809	0.038*
C12	0.9652 (3)	0.3440 (4)	0.2440 (2)	0.0240 (8)
C13	0.9042 (4)	0.2931 (4)	0.1697 (2)	0.0263 (8)
H13	0.821144	0.302385	0.154392	0.032*
C14	0.9629 (4)	0.2289 (4)	0.1177 (2)	0.0299 (9)
H14	0.919886	0.195983	0.066701	0.036*
C15	1.0834 (4)	0.2125 (4)	0.1395 (3)	0.0351 (10)
H15	1.123268	0.167383	0.104302	0.042*
C16	1.1461 (4)	0.2630 (5)	0.2140 (3)	0.0407 (11)
H16	1.229020	0.251918	0.229616	0.049*
C17	1.0879 (4)	0.3288 (4)	0.2649 (3)	0.0348 (10)
H17	1.131538	0.364479	0.314729	0.042*
C18	0.6148 (3)	0.4180 (4)	0.2054 (2)	0.0234 (8)
C19	0.6373 (4)	0.5350 (4)	0.1755 (2)	0.0265 (9)
H19	0.712723	0.572759	0.192434	0.032*
C20	0.5474 (4)	0.5967 (4)	0.1200 (2)	0.0270 (9)
H20	0.561108	0.676671	0.098215	0.032*
C21	0.4388 (4)	0.5400 (4)	0.0972 (2)	0.0260 (8)
C22	0.4166 (4)	0.4227 (4)	0.1279 (3)	0.0302 (9)
H22	0.341283	0.384649	0.111063	0.036*
C23	0.5054 (4)	0.3622 (4)	0.1832 (3)	0.0283 (9)
H23	0.491207	0.282848	0.205620	0.034*

Atomic displacement parameters (Å^2) for (Adebanji3)

	U^{11}	U^{22}	U^{33}	U^{12}	U^{13}	U^{23}
Br1	0.0292 (2)	0.0365 (3)	0.0318 (2)	0.0065 (2)	-0.00214 (17)	0.0042 (2)
S1	0.0257 (5)	0.0243 (5)	0.0238 (5)	0.0004 (4)	0.0027 (4)	-0.0005 (4)
O1	0.0220 (14)	0.0235 (14)	0.0254 (14)	0.0010 (11)	-0.0011 (11)	0.0009 (11)
O2	0.0321 (16)	0.0284 (15)	0.0298 (15)	0.0000 (12)	0.0070 (13)	-0.0053 (12)
O3	0.0232 (14)	0.0322 (15)	0.0288 (15)	0.0034 (12)	-0.0012 (12)	0.0027 (12)
C1	0.027 (2)	0.024 (2)	0.023 (2)	0.0030 (16)	0.0002 (16)	-0.0009 (15)
C2	0.025 (2)	0.0214 (19)	0.0233 (19)	0.0000 (15)	-0.0040 (16)	0.0001 (15)
C3	0.028 (2)	0.0235	0.0200	0.0010	-0.0015 (16)	0.0019

		(19)	(19)	(16)		(15)
C4	0.025 (2)	0.029 (2)	0.023 (2)	-0.0017 (16)	-0.0014 (16)	-0.0028 (16)
C5	0.029 (2)	0.028 (2)	0.025 (2)	-0.0023 (17)	0.0026 (17)	-0.0025 (16)
C6	0.026 (2)	0.025 (2)	0.0251 (19)	0.0014 (16)	0.0045 (16)	0.0003 (16)
C7	0.029 (2)	0.027 (2)	0.029 (2)	-0.0020 (18)	0.0045 (17)	-0.0028 (17)
C8	0.027 (2)	0.043 (3)	0.035 (2)	-0.0012 (19)	0.0035 (18)	-0.003 (2)
C9	0.037 (3)	0.044 (3)	0.039 (2)	-0.012 (2)	0.014 (2)	-0.013 (2)
C10	0.042 (3)	0.027 (2)	0.048 (3)	-0.005 (2)	0.014 (2)	-0.012 (2)
C11	0.033 (2)	0.027 (2)	0.036 (2)	0.0018 (18)	0.0106 (19)	-0.0030 (18)
C12	0.026 (2)	0.0222 (19)	0.0231 (19)	0.0005 (16)	0.0044 (16)	0.0048 (15)
C13	0.029 (2)	0.0225 (19)	0.0249 (19)	-0.0026 (16)	0.0004 (16)	0.0040 (15)
C14	0.042 (3)	0.023 (2)	0.024 (2)	-0.0066 (18)	0.0052 (18)	-0.0015 (16)
C15	0.038 (3)	0.035 (2)	0.035 (2)	0.003 (2)	0.015 (2)	-0.0038 (19)
C16	0.031 (2)	0.055 (3)	0.036 (2)	0.004 (2)	0.007 (2)	-0.003 (2)
C17	0.027 (2)	0.044 (3)	0.032 (2)	0.0032 (19)	0.0012 (18)	-0.0009 (19)
C18	0.025 (2)	0.0237 (19)	0.0193 (19)	0.0022 (15)	-0.0004 (16)	0.0003 (14)
C19	0.025 (2)	0.029 (2)	0.025 (2)	-0.0038 (17)	0.0033 (16)	-0.0020 (16)
C20	0.027 (2)	0.027 (2)	0.025 (2)	0.0015 (16)	0.0022 (16)	0.0017 (16)
C21	0.026 (2)	0.033 (2)	0.0177 (18)	-0.0001 (17)	0.0019 (16)	-0.0041 (16)
C22	0.023 (2)	0.031 (2)	0.034 (2)	-0.0011 (17)	0.0019 (18)	-0.0008 (18)
C23	0.026 (2)	0.025 (2)	0.033 (2)	-0.0024 (17)	0.0044 (17)	-0.0017 (17)

Geometric parameters (Å, º) for (Adebanji3)

Br1—C21	1.897 (4)	C9—H9	0.9500
S1—O3	1.441 (3)	C10—C11	1.386 (6)
S1—O2	1.445 (3)	C10—H10	0.9500
S1—C6	1.764 (4)	C11—H11	0.9500
S1—C1	1.808 (4)	C12—C13	1.390 (5)
O1—C2	1.389 (5)	C12—C17	1.404 (6)
O1—C18	1.406 (5)	C13—C14	1.389 (6)
C1—C2	1.499 (5)	C13—H13	0.9500
C1—C5	1.538 (6)	C14—C15	1.381 (6)
C1—H1	1.0000	C14—H14	0.9500
C2—C3	1.333 (6)	C15—C16	1.398 (6)
C3—C12	1.469 (5)	C15—H15	0.9500
C3—C4	1.513 (5)	C16—C17	1.379 (6)
C4—C5	1.541 (6)	C16—H16	0.9500
C4—H4A	0.9900	C17—H17	0.9500
C4—H4B	0.9900	C18—C23	1.380 (6)
C5—H5A	0.9900	C18—C19	1.384 (5)
C5—H5B	0.9900	C19—C20	1.398 (6)
C6—C11	1.384 (6)	C19—H19	0.9500
C6—C7	1.394 (6)	C20—C21	1.376 (6)
C7—C8	1.379 (6)	C20—H20	0.9500
C7—H7	0.9500	C21—C22	1.391 (6)
C8—C9	1.386 (7)	C22—C23	1.382 (6)
C8—H8	0.9500	C22—H22	0.9500
C9—C10	1.374 (7)	C23—H23	0.9500
O3—S1—O2	118.66 (17)	C9—C10—C11	120.6 (4)
O3—S1—C6	107.99 (18)	C9—C10—H10	119.7
O2—S1—C6	107.77 (18)	C11—C10—H10	119.7
O3—S1—C1	108.63 (18)	C6—C11—C10	118.8 (4)
O2—S1—C1	106.99 (17)	C6—C11—H11	120.6
C6—S1—C1	106.14 (19)	C10—C11—H11	120.6
C2—O1—C18	115.3 (3)	C13—C12—C17	118.1 (4)
C2—C1—C5	101.9 (3)	C13—C12—C3	122.9 (4)
C2—C1—S1	113.0 (3)	C17—C12—C3	118.9 (4)
C5—C1—S1	108.5 (3)	C14—C13—C12	121.0 (4)
C2—C1—H1	111.0	C14—C13—H13	119.5
C5—C1—H1	111.0	C12—C13—H13	119.5

S1—C1—H1	111.0	C15—C14—C13	120.3 (4)
C3—C2—O1	126.6 (4)	C15—C14—H14	119.8
C3—C2—C1	114.1 (3)	C13—C14—H14	119.8
O1—C2—C1	119.2 (3)	C14—C15—C16	119.4 (4)
C2—C3—C12	129.4 (4)	C14—C15—H15	120.3
C2—C3—C4	109.1 (3)	C16—C15—H15	120.3
C12—C3—C4	121.4 (4)	C17—C16—C15	120.1 (4)
C3—C4—C5	103.9 (3)	C17—C16—H16	120.0
C3—C4—H4A	111.0	C15—C16—H16	120.0
C5—C4—H4A	111.0	C16—C17—C12	121.0 (4)
C3—C4—H4B	111.0	C16—C17—H17	119.5
C5—C4—H4B	111.0	C12—C17—H17	119.5
H4A—C4—H4B	109.0	C23—C18—C19	121.6 (4)
C1—C5—C4	105.0 (3)	C23—C18—O1	116.8 (3)
C1—C5—H5A	110.7	C19—C18—O1	121.5 (3)
C4—C5—H5A	110.7	C18—C19—C20	118.9 (4)
C1—C5—H5B	110.7	C18—C19—H19	120.5
C4—C5—H5B	110.7	C20—C19—H19	120.5
H5A—C5—H5B	108.8	C21—C20—C19	119.2 (4)
C11—C6—C7	121.2 (4)	C21—C20—H20	120.4
C11—C6—S1	119.5 (3)	C19—C20—H20	120.4
C7—C6—S1	119.3 (3)	C20—C21—C22	121.6 (4)
C8—C7—C6	118.9 (4)	C20—C21—Br1	118.9 (3)
C8—C7—H7	120.5	C22—C21—Br1	119.5 (3)
C6—C7—H7	120.5	C23—C22—C21	119.1 (4)
C7—C8—C9	120.2 (4)	C23—C22—H22	120.4
C7—C8—H8	119.9	C21—C22—H22	120.4
C9—C8—H8	119.9	C18—C23—C22	119.5 (4)
C10—C9—C8	120.2 (4)	C18—C23—H23	120.3
C10—C9—H9	119.9	C22—C23—H23	120.3
C8—C9—H9	119.9		
O3—S1—C1—C2	54.0 (3)	C7—C8—C9—C10	1.7 (7)
O2—S1—C1—C2	-176.8 (3)	C8—C9—C10—C11	-2.2 (7)
C6—S1—C1—C2	-61.9 (3)	C7—C6—C11—C10	0.3 (6)
O3—S1—C1—C5	-58.3 (3)	S1—C6—C11—C10	178.4 (3)
O2—S1—C1—C5	71.0 (3)	C9—C10—C11—C6	1.3 (7)
C6—S1—C1—C5	-174.2 (3)	C2—C3—C12—C13	20.1 (6)
C18—O1—C2—	-116.4 (4)	C4—C3—C12—C13	-161.8 (4)

C3			
C18—O1—C2—C1	67.0 (4)	C2—C3—C12—C17	-162.1 (4)
C5—C1—C2—C3	14.8 (4)	C4—C3—C12—C17	16.1 (6)
S1—C1—C2—C3	-101.5 (4)	C17—C12—C13—C14	0.1 (6)
C5—C1—C2—O1	-168.3 (3)	C3—C12—C13—C14	178.0 (4)
S1—C1—C2—O1	75.5 (4)	C12—C13—C14—C15	1.1 (6)
O1—C2—C3—C12	1.6 (7)	C13—C14—C15—C16	-1.0 (6)
C1—C2—C3—C12	178.3 (4)	C14—C15—C16—C17	-0.2 (7)
O1—C2—C3—C4	-176.7 (3)	C15—C16—C17—C12	1.4 (7)
C1—C2—C3—C4	0.0 (5)	C13—C12—C17—C16	-1.4 (6)
C2—C3—C4—C5	-14.8 (4)	C3—C12—C17—C16	-179.3 (4)
C12—C3—C4—C5	166.7 (3)	C2—O1—C18—C23	-155.4 (4)
C2—C1—C5—C4	-22.7 (4)	C2—O1—C18—C19	27.4 (5)
S1—C1—C5—C4	96.8 (3)	C23—C18—C19—C20	-1.4 (6)
C3—C4—C5—C1	23.2 (4)	O1—C18—C19—C20	175.8 (3)
O3—S1—C6—C11	-6.3 (4)	C18—C19—C20—C21	0.6 (6)
O2—S1—C6—C11	-135.6 (3)	C19—C20—C21—C22	-0.3 (6)
C1—S1—C6—C11	110.1 (4)	C19—C20—C21—Br1	179.7 (3)
O3—S1—C6—C7	171.9 (3)	C20—C21—C22—C23	0.7 (6)
O2—S1—C6—C7	42.6 (4)	Br1—C21—C22—C23	-179.3 (3)
C1—S1—C6—C7	-71.7 (4)	C19—C18—C23—C22	1.8 (6)
C11—C6—C7—C8	-0.8 (6)	O1—C18—C23—C22	-175.5 (4)
S1—C6—C7—C8	-179.0 (3)	C21—C22—C23—	-1.4 (6)

		C18	
C6—C7—C8—C9	-0.2 (6)		

Compound 16a

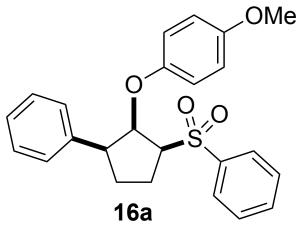

16a

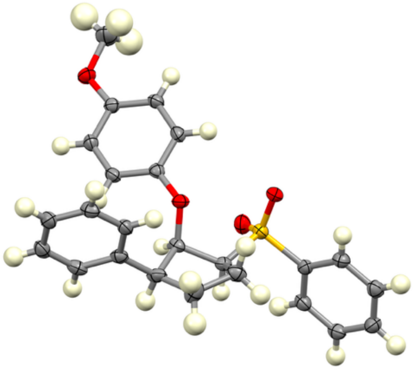

Sample Name: CCDC 2517779 / AA1201Pneedle
Crystal Growth: Slow evaporation using a mixture of hexanes and dichloromethane

Crystal data

$C_{24}H_{24}O_4S{\cdot}CHCl_3$	$F(000) = 1096$
$M_r = 527.86$	$D_x = 1.443$ Mg m^{-3}
Monoclinic, $P2_1/n$	Cu *K*a radiation, l = 1.54184 Å
a = 5.7652 (3) Å	Cell parameters from 1363 reflections
b = 15.0592 (7) Å	q = 4.3–73.5°
c = 28.0147 (14) Å	m = 4.47 mm^{-1}
b = 92.337 (4)°	*T* = 100 K
V = 2430.2 (2) Å^3	Needle, colourless
Z = 4	0.30 × 0.03 × 0.02 mm

Data collection

Bruker D8 Venture DUO with Photon III C14 diffractometer	3053 reflections with *I* > 2s(*I*)
Radiation source: ImS 3.0 microfocus	$R_{int} = 0.135$
f and w scans	$q_{max} = 74.6°$, $q_{min} = 3.2°$
Absorption correction: multi-scan *SADABS* (Krause *et al.*, 2015)	*h* = -4 - 7
$T_{min} = 0.768$, $T_{max} = 0.916$	*k* = -18 - 18
27316 measured reflections	*l* = -34 - 35
4810 independent reflections	

Refinement

Refinement on F^2	Hydrogen site location: inferred from neighbouring sites
Least-squares matrix: full	H-atom parameters constrained
$R[F^2 > 2s(F^2)] = 0.066$	$w = 1/[s^2(F_o^2) + (0.0729P)^2 + 2.3219P]$ where $P = (F_o^2 + 2F_c^2)/3$
$wR(F^2) = 0.172$	$(D/s)_{max} < 0.001$
$S = 1.01$	$Dr_{max} = 0.83$ e Å^{-3}
4810 reflections	$Dr_{min} = -0.94$ e Å^{-3}
300 parameters	Extinction correction: *SHELXL2019*/1 (Sheldrick 2019), $Fc^* = kFc[1+0.001xFc^2l^3/\sin(2q)]^{-1/4}$
0 restraints	Extinction coefficient: 0.0025 (3)

Fractional atomic coordinates and isotropic or equivalent isotropic displacement parameters (Å^2) for (AA1201Pneedle)

	x	*y*	*z*	$U_{iso}*/U_{eq}$
S1	0.52224 (18)	0.16555 (7)	0.30637 (3)	0.0269 (3)
O1	0.4688 (5)	0.28997 (18)	0.39050 (9)	0.0267 (6)
O2	0.7567 (5)	0.18102 (19)	0.32497 (11)	0.0335 (7)
O3	0.4220 (6)	0.22652 (19)	0.27163 (10)	0.0342 (7)
O4	0.5311 (6)	0.64630 (19)	0.34469 (10)	0.0335 (7)
C1	0.3243 (7)	0.1554 (3)	0.35343 (14)	0.0266 (9)
H1	0.177557	0.129195	0.339329	0.032*
C2	0.2598 (7)	0.2435 (3)	0.37771 (13)	0.0242 (8)
H2	0.155654	0.280244	0.356206	0.029*
C3	0.1301 (9)	0.2100 (3)	0.42141 (14)	0.0336 (10)
H3	-0.035562	0.201036	0.410474	0.040*
C4	0.2294 (11)	0.1178 (3)	0.43342 (17)	0.0450 (13)
H4A	0.103466	0.073051	0.433008	0.054*
H4B	0.306927	0.118083	0.465614	0.054*
C5	0.4071 (8)	0.0951 (3)	0.39501 (15)	0.0347 (10)
H5A	0.567607	0.109742	0.406210	0.042*
H5B	0.399059	0.031588	0.385913	0.042*
C6	0.5141 (7)	0.0560 (3)	0.28253 (13)	0.0260 (8)
C7	0.3182 (8)	0.0278 (3)	0.25585 (14)	0.0314 (9)
H7	0.193861	0.067662	0.248663	0.038*
C8	0.3082 (8)	-0.0593 (3)	0.24000 (14)	0.0317 (9)
H8	0.175147	-0.079804	0.222141	0.038*
C9	0.4925 (8)	-0.1169 (3)	0.25016 (15)	0.0336 (10)

H9	0.483536	-0.176797	0.239678	0.040*
C10	0.6885 (8)	-0.0873 (3)	0.27544 (15)	0.0330 (10)
H10	0.815853	-0.126372	0.281358	0.040*
C11	0.6995 (8)	-0.0004 (3)	0.29222 (14)	0.0304 (9)
H11	0.832598	0.019992	0.310097	0.036*
C12	0.1291 (8)	0.2738 (3)	0.46283 (14)	0.0279 (9)
C13	-0.0687 (8)	0.3246 (3)	0.47037 (14)	0.0310 (9)
H13	-0.203233	0.316921	0.450064	0.037*
C14	-0.0709 (8)	0.3861 (3)	0.50722 (16)	0.0352 (10)
H14	-0.205590	0.421059	0.511592	0.042*
C15	0.1220 (8)	0.3968 (3)	0.53757 (15)	0.0346 (10)
H15	0.121249	0.439181	0.562697	0.042*
C16	0.3154 (8)	0.3453 (3)	0.53091 (14)	0.0331 (10)
H16	0.446925	0.351357	0.552205	0.040*
C17	0.3215 (8)	0.2851 (3)	0.49393 (14)	0.0295 (9)
H17	0.457868	0.251068	0.489621	0.035*
C18	0.4784 (7)	0.3790 (3)	0.37781 (13)	0.0243 (8)
C19	0.3087 (7)	0.4393 (3)	0.38969 (14)	0.0272 (9)
H19	0.176662	0.419906	0.406046	0.033*
C20	0.3324 (8)	0.5280 (3)	0.37757 (14)	0.0290 (9)
H20	0.214792	0.569121	0.385297	0.035*
C21	0.5266 (7)	0.5574 (3)	0.35422 (13)	0.0256 (8)
C22	0.6953 (7)	0.4966 (3)	0.34244 (14)	0.0273 (9)
H22	0.827924	0.515621	0.326173	0.033*
C23	0.6701 (7)	0.4075 (3)	0.35451 (14)	0.0277 (9)
H23	0.786510	0.365950	0.346577	0.033*
C24	0.7319 (10)	0.6804 (3)	0.3236 (2)	0.0527 (14)
H24A	0.719308	0.745096	0.320862	0.079*
H24B	0.745754	0.654463	0.291757	0.079*
H24C	0.869633	0.665158	0.343603	0.079*
Cl1	0.2267 (2)	0.89834 (8)	0.47795 (5)	0.0460 (3)
Cl2	-0.1775 (3)	0.90278 (11)	0.41419 (6)	0.0671 (5)
Cl3	0.2311 (3)	0.80717 (17)	0.38807 (7)	0.1007 (8)
C25	0.0611 (9)	0.8396 (3)	0.43411 (18)	0.0417 (11)
H25	0.000870	0.784710	0.449389	0.050*

Atomic displacement parameters (Å^2) for (AA1201Pneedle)

	U^{11}	U^{22}	U^{33}	U^{12}	U^{13}	U^{23}
S1	0.0226 (5)	0.0264 (5)	0.0320 (5)	-0.0004	0.0044 (4)	-0.0001

				(4)		(4)
O1	0.0227 (15)	0.0250 (15)	0.0321 (14)	-0.0047 (12)	-0.0008 (12)	0.0016 (11)
O2	0.0209 (16)	0.0325 (17)	0.0475 (17)	-0.0042 (13)	0.0056 (13)	-0.0081 (13)
O3	0.0378 (18)	0.0309 (16)	0.0346 (15)	0.0063 (14)	0.0087 (13)	0.0039 (12)
O4	0.0383 (19)	0.0244 (15)	0.0385 (15)	-0.0032 (13)	0.0085 (14)	0.0018 (12)
C1	0.023 (2)	0.026 (2)	0.0314 (19)	-0.0055 (17)	0.0026 (16)	-0.0015 (15)
C2	0.018 (2)	0.030 (2)	0.0241 (17)	-0.0029 (17)	0.0004 (15)	-0.0002 (15)
C3	0.038 (3)	0.034 (2)	0.030 (2)	-0.017 (2)	0.0097 (18)	-0.0041 (17)
C4	0.067 (4)	0.032 (3)	0.036 (2)	0.000 (2)	0.012 (2)	-0.0003 (19)
C5	0.031 (2)	0.036 (3)	0.037 (2)	-0.002 (2)	0.0027 (18)	0.0086 (18)
C6	0.027 (2)	0.023 (2)	0.0280 (18)	-0.0013 (17)	0.0048 (16)	-0.0010 (15)
C7	0.024 (2)	0.038 (2)	0.032 (2)	-0.0005 (19)	0.0047 (17)	-0.0011 (17)
C8	0.027 (2)	0.038 (2)	0.030 (2)	-0.0064 (19)	0.0030 (17)	-0.0019 (17)
C9	0.037 (3)	0.027 (2)	0.037 (2)	-0.004 (2)	0.0063 (19)	-0.0037 (17)
C10	0.030 (2)	0.031 (2)	0.038 (2)	0.0019 (19)	0.0062 (18)	0.0052 (17)
C11	0.026 (2)	0.031 (2)	0.034 (2)	-0.0028 (18)	0.0004 (17)	-0.0008 (17)
C12	0.028 (2)	0.029 (2)	0.0271 (19)	-0.0072 (18)	0.0055 (16)	0.0023 (16)
C13	0.021 (2)	0.039 (3)	0.033 (2)	-0.0051 (19)	-0.0004 (16)	0.0098 (17)
C14	0.029 (2)	0.033 (2)	0.044 (2)	0.004 (2)	0.012 (2)	0.0081 (19)
C15	0.036 (3)	0.034 (2)	0.034 (2)	-0.003 (2)	0.0126 (19)	-0.0038 (18)
C16	0.029 (2)	0.041 (3)	0.029 (2)	-0.008 (2)	0.0016 (17)	-0.0029 (17)
C17	0.024 (2)	0.029 (2)	0.036 (2)	0.0007	0.0069	0.0022

				(18)	(17)	(17)
C18	0.021 (2)	0.027 (2)	0.0245 (17)	-0.0008 (17)	-0.0007 (15)	-0.0019 (14)
C19	0.021 (2)	0.031 (2)	0.0293 (19)	-0.0031 (17)	0.0052 (16)	-0.0009 (16)
C20	0.026 (2)	0.028 (2)	0.033 (2)	0.0033 (18)	0.0065 (17)	-0.0007 (16)
C21	0.025 (2)	0.024 (2)	0.0275 (18)	-0.0019 (17)	0.0011 (16)	0.0009 (15)
C22	0.022 (2)	0.029 (2)	0.0309 (19)	-0.0034 (17)	0.0051 (16)	0.0021 (16)
C23	0.020 (2)	0.030 (2)	0.033 (2)	0.0031 (18)	0.0021 (16)	-0.0032 (16)
C24	0.050 (3)	0.037 (3)	0.074 (4)	-0.003 (2)	0.026 (3)	0.013 (2)
Cl1	0.0409 (7)	0.0396 (7)	0.0573 (7)	-0.0016 (5)	0.0003 (5)	0.0061 (5)
Cl2	0.0477 (9)	0.0637 (10)	0.0881 (11)	0.0120 (7)	-0.0180 (7)	0.0064 (8)
Cl3	0.0460 (10)	0.158 (2)	0.1000 (13)	-0.0202 (11)	0.0286 (9)	-0.0718 (14)
C25	0.033 (3)	0.039 (3)	0.052 (3)	-0.001 (2)	0.001 (2)	0.003 (2)

Geometric parameters (Å, º) for (AA1201Pneedle)

S1—O3	1.442 (3)	C10—H10	0.9500
S1—O2	1.448 (3)	C11—H11	0.9500
S1—C6	1.780 (4)	C12—C17	1.393 (6)
S1—C1	1.785 (4)	C12—C13	1.396 (6)
O1—C18	1.389 (5)	C13—C14	1.388 (6)
O1—C2	1.427 (5)	C13—H13	0.9500
O4—C21	1.365 (5)	C14—C15	1.381 (7)
O4—C24	1.417 (6)	C14—H14	0.9500
C1—C5	1.537 (6)	C15—C16	1.377 (7)
C1—C2	1.543 (6)	C15—H15	0.9500
C1—H1	1.0000	C16—C17	1.378 (6)
C2—C3	1.545 (5)	C16—H16	0.9500
C2—H2	1.0000	C17—H17	0.9500
C3—C12	1.507 (6)	C18—C23	1.375 (6)
C3—C4	1.534 (7)	C18—C19	1.385 (6)
C3—H3	1.0000	C19—C20	1.386 (6)
C4—C5	1.554 (7)	C19—H19	0.9500

C4—H4A	0.9900	C20—C21	1.392 (6)
C4—H4B	0.9900	C20—H20	0.9500
C5—H5A	0.9900	C21—C22	1.386 (6)
C5—H5B	0.9900	C22—C23	1.393 (6)
C6—C11	1.383 (6)	C22—H22	0.9500
C6—C7	1.395 (6)	C23—H23	0.9500
C7—C8	1.386 (6)	C24—H24A	0.9800
C7—H7	0.9500	C24—H24B	0.9800
C8—C9	1.392 (7)	C24—H24C	0.9800
C8—H8	0.9500	Cl1—C25	1.762 (5)
C9—C10	1.383 (6)	Cl2—C25	1.745 (5)
C9—H9	0.9500	Cl3—C25	1.722 (5)
C10—C11	1.391 (6)	C25—H25	1.0000
O3—S1—O2	118.90 (19)	C11—C10—H10	120.0
O3—S1—C6	109.48 (19)	C6—C11—C10	119.1 (4)
O2—S1—C6	107.12 (19)	C6—C11—H11	120.4
O3—S1—C1	107.62 (19)	C10—C11—H11	120.4
O2—S1—C1	111.32 (19)	C17—C12—C13	118.3 (4)
C6—S1—C1	100.93 (19)	C17—C12—C3	122.1 (4)
C18—O1—C2	116.8 (3)	C13—C12—C3	119.6 (4)
C21—O4—C24	117.3 (4)	C14—C13—C12	120.7 (4)
C5—C1—C2	104.3 (3)	C14—C13—H13	119.7
C5—C1—S1	115.0 (3)	C12—C13—H13	119.7
C2—C1—S1	115.1 (3)	C15—C14—C13	120.3 (4)
C5—C1—H1	107.3	C15—C14—H14	119.9
C2—C1—H1	107.3	C13—C14—H14	119.9
S1—C1—H1	107.3	C16—C15—C14	119.1 (4)
O1—C2—C1	108.4 (3)	C16—C15—H15	120.4
O1—C2—C3	113.1 (3)	C14—C15—H15	120.4
C1—C2—C3	101.6 (3)	C15—C16—C17	121.2 (4)
O1—C2—H2	111.1	C15—C16—H16	119.4
C1—C2—H2	111.1	C17—C16—H16	119.4
C3—C2—H2	111.1	C16—C17—C12	120.4 (4)
C12—C3—C4	114.9 (4)	C16—C17—H17	119.8
C12—C3—C2	114.8 (3)	C12—C17—H17	119.8
C4—C3—C2	106.3 (4)	C23—C18—C19	120.0 (4)
C12—C3—H3	106.8	C23—C18—O1	117.6 (4)
C4—C3—H3	106.8	C19—C18—O1	122.4 (4)

C2—C3—H3	106.8	C18—C19—C20	119.6 (4)
C3—C4—C5	107.3 (4)	C18—C19—H19	120.2
C3—C4—H4A	110.2	C20—C19—H19	120.2
C5—C4—H4A	110.2	C19—C20—C21	120.7 (4)
C3—C4—H4B	110.2	C19—C20—H20	119.6
C5—C4—H4B	110.2	C21—C20—H20	119.6
H4A—C4—H4B	108.5	O4—C21—C22	125.6 (4)
C1—C5—C4	101.6 (4)	O4—C21—C20	115.2 (4)
C1—C5—H5A	111.4	C22—C21—C20	119.2 (4)
C4—C5—H5A	111.4	C21—C22—C23	119.9 (4)
C1—C5—H5B	111.4	C21—C22—H22	120.1
C4—C5—H5B	111.4	C23—C22—H22	120.1
H5A—C5—H5B	109.3	C18—C23—C22	120.6 (4)
C11—C6—C7	121.4 (4)	C18—C23—H23	119.7
C11—C6—S1	119.1 (3)	C22—C23—H23	119.7
C7—C6—S1	119.4 (3)	O4—C24—H24A	109.5
C8—C7—C6	118.8 (4)	O4—C24—H24B	109.5
C8—C7—H7	120.6	H24A—C24—H24B	109.5
C6—C7—H7	120.6	O4—C24—H24C	109.5
C7—C8—C9	120.2 (4)	H24A—C24—H24C	109.5
C7—C8—H8	119.9	H24B—C24—H24C	109.5
C9—C8—H8	119.9	Cl3—C25—Cl2	112.4 (3)
C10—C9—C8	120.4 (4)	Cl3—C25—Cl1	110.8 (3)
C10—C9—H9	119.8	Cl2—C25—Cl1	110.3 (3)
C8—C9—H9	119.8	Cl3—C25—H25	107.7
C9—C10—C11	120.0 (4)	Cl2—C25—H25	107.7
C9—C10—H10	120.0	Cl1—C25—H25	107.7
O3—S1—C1—C5	-177.8 (3)	C8—C9—C10—C11	2.1 (6)
O2—S1—C1—C5	-45.9 (4)	C7—C6—C11—C10	-0.8 (6)
C6—S1—C1—C5	67.5 (3)	S1—C6—C11—C10	177.1 (3)
O3—S1—C1—C2	-56.5 (3)	C9—C10—C11—C6	-1.1 (6)
O2—S1—C1—C2	75.4 (3)	C4—C3—C12—C17	46.2 (6)
C6—S1—C1—C2	-171.2 (3)	C2—C3—C12—C17	-77.6 (5)
C18—O1—C2—C1	131.6 (3)	C4—C3—C12—C13	-134.6 (4)
C18—O1—C2—C3	-116.5 (4)	C2—C3—C12—C13	101.6 (5)
C5—C1—C2—O1	76.7 (4)	C17—C12—C13—	1.5 (6)

		C14	
S1—C1—C2—O1	-50.2 (4)	C3—C12—C13—C14	-177.7 (4)
C5—C1—C2—C3	-42.7 (4)	C12—C13—C14—C15	-1.2 (6)
S1—C1—C2—C3	-169.6 (3)	C13—C14—C15—C16	-0.4 (6)
O1—C2—C3—C12	39.9 (5)	C14—C15—C16—C17	1.6 (6)
C1—C2—C3—C12	155.9 (4)	C15—C16—C17—C12	-1.3 (6)
O1—C2—C3—C4	-88.4 (4)	C13—C12—C17—C16	-0.3 (6)
C1—C2—C3—C4	27.7 (4)	C3—C12—C17—C16	178.8 (4)
C12—C3—C4—C5	-131.3 (4)	C2—O1—C18—C23	-130.3 (4)
C2—C3—C4—C5	-3.2 (5)	C2—O1—C18—C19	52.6 (5)
C2—C1—C5—C4	40.5 (4)	C23—C18—C19—C20	0.6 (6)
S1—C1—C5—C4	167.5 (3)	O1—C18—C19—C20	177.6 (4)
C3—C4—C5—C1	-22.7 (5)	C18—C19—C20—C21	-0.9 (6)
O3—S1—C6—C11	141.7 (3)	C24—O4—C21—C22	-4.1 (6)
O2—S1—C6—C11	11.5 (4)	C24—O4—C21—C20	176.2 (4)
C1—S1—C6—C11	-105.0 (3)	C19—C20—C21—O4	-179.3 (4)
O3—S1—C6—C7	-40.4 (4)	C19—C20—C21—C22	1.0 (6)
O2—S1—C6—C7	-170.5 (3)	O4—C21—C22—C23	179.6 (4)
C1—S1—C6—C7	72.9 (3)	C20—C21—C22—C23	-0.7 (6)
C11—C6—C7—C8	1.8 (6)	C19—C18—C23—C22	-0.3 (6)
S1—C6—C7—C8	-176.1 (3)	O1—C18—C23—C22	-177.4 (3)
C6—C7—C8—C9	-0.8 (6)	C21—C22—C23—C18	0.4 (6)

C7—C8—C9—C10	-1.1 (6)		

Compound 16b

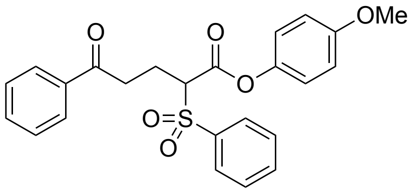

16b

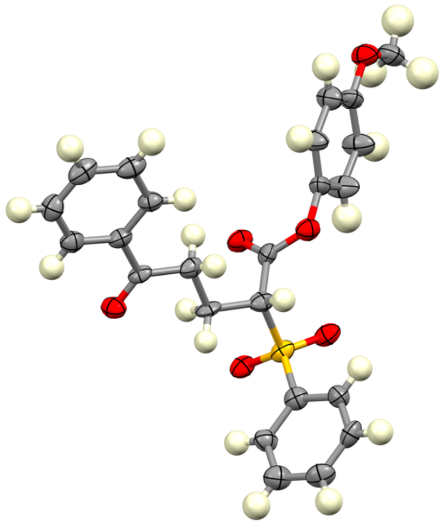

Sample Name: CCDC 2517780 / Aldas3

Crystal Growth: Slow evaporation using a mixture of hexanes and dichloromethane

Crystal data

$C_{24}H_{22}O_6S$	*F*(000) = 2760
M_r = 438.47	D_x = 1.381 Mg m^{-3}
Monoclinic, $P2_1/n$	Cu *K*a radiation, l = 1.54184 Å
a = 5.1934 (8) Å	Cell parameters from 1174 reflections
b = 37.766 (7) Å	q = 4.7–56.0°
c = 32.270 (6) Å	m = 1.70 mm^{-1}
b = 91.586 (12)°	*T* = 100 K
V = 6326.9 (18) Å^3	Needle, colourless
Z = 12	0.24 × 0.04 × 0.04 mm

Data collection

Bruker D8 Venture DUO with Photon III C14 diffractometer	4144 reflections with *I* > 2s(*I*)
Radiation source: ImS 3.0 microfocus	R_{int} = 0.552
f and w scans	q_{max} = 60.5°, q_{min} = 2.3°
Absorption correction: multi-scan *SADABS* (Krause *et al.*, 2015)	*h* = -5 - 5
T_{min} = 0.756, T_{max} = 0.935	*k* = -42 - 41
81274 measured reflections	*l* = -35 - 35
9259 independent reflections	

Refinement

Refinement on F^2	Hydrogen site location: inferred from neighbouring sites
Least-squares matrix: full	H-atom parameters constrained
$R[F^2 > 2s(F^2)] = 0.106$	$w = 1/[s^2(F_o^2) + (0.0698P)^2]$ where $P = (F_o^2 + 2F_c^2)/3$
$wR(F^2) = 0.298$	$(D/s)_{max} < 0.001$
$S = 1.03$	$Dr_{max} = 0.44$ e Å^{-3}
9259 reflections	$Dr_{min} = -0.50$ e Å^{-3}
842 parameters	Extinction correction: *SHELXL2019*/1 (Sheldrick 2019), $Fc^* = kFc[1+0.001xFc^2l^3/\sin(2q)]^{-1/4}$
0 restraints	Extinction coefficient: 0.00159 (18)

Fractional atomic coordinates and isotropic or equivalent isotropic displacement parameters (Å^2) for (Aldas3)

	x	*y*	*z*	U_{iso}*/U_{eq}
S2	0.6203 (5)	0.61979 (7)	0.28162 (7)	0.0367 (6)
O7	0.5783 (14)	0.58314 (19)	0.2752 (2)	0.0450 (18)
O8	0.4040 (13)	0.64327 (18)	0.2816 (2)	0.0404 (17)
O9	0.9326 (14)	0.74483 (18)	0.2233 (2)	0.0429 (17)
O10	0.8905 (13)	0.58950 (18)	0.1931 (2)	0.0418 (17)
O11	0.5607 (13)	0.62772 (19)	0.1815 (2)	0.0426 (17)
O12	0.6771 (16)	0.50796 (19)	0.0542 (2)	0.053 (2)
C25	0.8369 (19)	0.6341 (3)	0.2428 (3)	0.038 (2)
H25	1.012844	0.624731	0.249801	0.045*
C26	0.7411 (19)	0.6174 (2)	0.2024 (3)	0.035 (2)
C27	0.8507 (19)	0.6740 (3)	0.2397 (3)	0.035 (2)
H27A	0.901320	0.683922	0.267110	0.042*
H27B	0.678005	0.683401	0.231914	0.042*
C28	1.0450 (19)	0.6862 (3)	0.2075 (3)	0.038 (2)
H28A	1.215168	0.675754	0.214693	0.046*
H28B	0.989923	0.676790	0.179980	0.046*
C29	1.0743 (18)	0.7252 (2)	0.2042 (3)	0.029 (2)
C30	1.2677 (19)	0.7401 (3)	0.1759 (3)	0.036 (2)
C31	1.283 (2)	0.7758 (3)	0.1706 (3)	0.041 (3)
H31	1.169904	0.790947	0.185043	0.049*
C32	1.464 (2)	0.7905 (3)	0.1438 (3)	0.044 (3)
H32	1.473949	0.815386	0.139756	0.053*
C33	1.626 (2)	0.7681 (3)	0.1236 (3)	0.049 (3)

H33	1.748825	0.777730	0.105485	0.058*
C34	1.616 (2)	0.7324 (3)	0.1288 (3)	0.048 (3)
H34	1.729733	0.717360	0.114409	0.058*
C35	1.4366 (19)	0.7180 (3)	0.1556 (3)	0.037 (2)
H35	1.430326	0.693124	0.159862	0.045*
C36	0.7961 (19)	0.6253 (3)	0.3277 (3)	0.038 (2)
C37	0.992 (2)	0.6020 (3)	0.3388 (3)	0.041 (2)
H37	1.032878	0.583211	0.320541	0.049*
C38	1.128 (2)	0.6055 (3)	0.3756 (3)	0.044 (3)
H38	1.261328	0.589050	0.382500	0.053*
C39	1.074 (2)	0.6326 (3)	0.4026 (3)	0.048 (3)
H39	1.168698	0.635110	0.428085	0.058*
C40	0.877 (2)	0.6565 (3)	0.3917 (3)	0.050 (3)
H40	0.838798	0.675459	0.409925	0.060*
C41	0.737 (2)	0.6528 (3)	0.3551 (3)	0.041 (2)
H41	0.601812	0.668899	0.348326	0.049*
C42	0.820 (2)	0.5700 (3)	0.1572 (3)	0.040 (2)
C43	0.976 (2)	0.5717 (3)	0.1230 (3)	0.049 (3)
H43	1.120321	0.587228	0.122745	0.059*
C44	0.916 (2)	0.5503 (3)	0.0894 (3)	0.049 (3)
H44	1.021029	0.551528	0.065809	0.059*
C45	0.715 (2)	0.5276 (3)	0.0889 (3)	0.043 (3)
C46	0.565 (2)	0.5251 (3)	0.1240 (3)	0.056 (3)
H46	0.426567	0.508673	0.124881	0.067*
C47	0.620 (2)	0.5466 (3)	0.1571 (3)	0.057 (3)
H47	0.516483	0.545202	0.180835	0.069*
C48	0.475 (2)	0.4817 (3)	0.0536 (3)	0.054 (3)
H48A	0.465848	0.470215	0.026334	0.081*
H48B	0.309911	0.493134	0.058964	0.081*
H48C	0.512042	0.463874	0.075006	0.081*
S3	1.0900 (5)	0.37878 (7)	0.03454 (7)	0.0375 (6)
O13	1.1306 (14)	0.41596 (19)	0.0358 (2)	0.0473 (18)
O14	1.3048 (13)	0.35574 (18)	0.0373 (2)	0.0395 (16)
O15	0.7441 (14)	0.26103 (19)	0.1119 (2)	0.0445 (17)
O16	0.7899 (13)	0.41471 (18)	0.12108 (19)	0.0396 (16)
O17	1.1510 (13)	0.38354 (19)	0.1337 (2)	0.0428 (17)
O18	0.9452 (15)	0.49721 (19)	0.2608 (2)	0.0469 (18)
C49	0.8776 (17)	0.3688 (2)	0.0755 (3)	0.031 (2)
H49	0.701868	0.377344	0.066842	0.037*

C50	0.9604 (19)	0.3892 (2)	0.1136 (3)	0.034 (2)
C51	0.8580 (19)	0.3294 (3)	0.0845 (3)	0.036 (2)
H51A	1.028749	0.320221	0.093732	0.043*
H51B	0.803849	0.316640	0.058942	0.043*
C52	0.6625 (18)	0.3227 (3)	0.1183 (3)	0.036 (2)
H52A	0.721880	0.335048	0.143910	0.043*
H52B	0.495597	0.333332	0.109403	0.043*
C53	0.6205 (17)	0.2845 (3)	0.1281 (3)	0.035 (2)
C54	0.4153 (18)	0.2759 (3)	0.1585 (3)	0.034 (2)
C55	0.361 (2)	0.2409 (3)	0.1662 (3)	0.039 (2)
H55	0.450808	0.222732	0.152363	0.047*
C56	0.172 (2)	0.2323 (3)	0.1945 (3)	0.046 (3)
H56	0.136018	0.208134	0.200075	0.055*
C57	0.039 (2)	0.2579 (4)	0.2140 (3)	0.053 (3)
H57	-0.091993	0.251577	0.232675	0.064*
C58	0.092 (2)	0.2930 (3)	0.2070 (3)	0.050 (3)
H58	0.000256	0.310784	0.221340	0.060*
C59	0.2819 (19)	0.3029 (3)	0.1786 (3)	0.040 (2)
H59	0.318060	0.327084	0.173368	0.049*
C60	0.9033 (19)	0.3685 (3)	-0.0106 (3)	0.039 (2)
C61	0.717 (2)	0.3920 (3)	-0.0236 (3)	0.041 (2)
H61	0.689243	0.413527	-0.009184	0.050*
C62	0.567 (2)	0.3831 (3)	-0.0591 (3)	0.050 (3)
H62	0.436249	0.398724	-0.068901	0.060*
C63	0.611 (2)	0.3515 (3)	-0.0798 (3)	0.047 (3)
H63	0.506031	0.345391	-0.103355	0.056*
C64	0.804 (2)	0.3292 (3)	-0.0665 (3)	0.045 (3)
H64	0.834435	0.308054	-0.081646	0.054*
C65	0.9573 (19)	0.3366 (3)	-0.0312 (3)	0.040 (2)
H65	1.090075	0.321057	-0.021754	0.048*
C66	0.8385 (19)	0.4359 (2)	0.1559 (3)	0.034 (2)
C67	1.032 (2)	0.4602 (3)	0.1570 (3)	0.055 (3)
H67	1.143488	0.461882	0.134180	0.066*
C68	1.071 (2)	0.4822 (3)	0.1905 (3)	0.050 (3)
H68	1.198528	0.500180	0.190100	0.061*
C69	0.918 (2)	0.4778 (3)	0.2253 (3)	0.037 (2)
C70	0.722 (2)	0.4532 (3)	0.2240 (3)	0.048 (3)
H70	0.612738	0.450796	0.246991	0.058*
C71	0.682 (2)	0.4319 (3)	0.1897 (3)	0.049 (3)

H71	0.549376	0.414663	0.189236	0.058*
C72	1.148 (2)	0.5218 (3)	0.2634 (3)	0.049 (3)
H72A	1.148038	0.533475	0.290523	0.074*
H72B	1.123908	0.539631	0.241504	0.074*
H72C	1.311857	0.509582	0.259902	0.074*
S1	0.2559 (5)	0.59997 (7)	0.61313 (7)	0.0358 (6)
O1	0.1988 (12)	0.56396 (18)	0.6067 (2)	0.0398 (16)
O2	0.0497 (12)	0.62477 (17)	0.61448 (19)	0.0360 (15)
O3	0.6006 (14)	0.72384 (17)	0.5562 (2)	0.0408 (17)
O4	0.5343 (13)	0.56960 (17)	0.52327 (19)	0.0396 (17)
O5	0.2069 (14)	0.60753 (18)	0.5120 (2)	0.0418 (17)
O6	0.4283 (14)	0.50259 (18)	0.37209 (19)	0.0428 (17)
C1	0.4737 (19)	0.6136 (3)	0.5743 (3)	0.037 (2)
H1	0.647809	0.603888	0.581793	0.045*
C2	0.3855 (19)	0.5972 (3)	0.5327 (3)	0.035 (2)
C3	0.4967 (18)	0.6528 (3)	0.5711 (3)	0.034 (2)
H3A	0.543205	0.662652	0.598752	0.041*
H3B	0.327792	0.662878	0.562302	0.041*
C4	0.7010 (19)	0.6641 (2)	0.5400 (3)	0.034 (2)
H4A	0.868592	0.653537	0.548691	0.041*
H4B	0.652802	0.654264	0.512417	0.041*
C5	0.7335 (19)	0.7035 (3)	0.5361 (3)	0.038 (2)
C6	0.9332 (18)	0.7176 (3)	0.5081 (3)	0.034 (2)
C7	0.949 (2)	0.7535 (2)	0.5016 (3)	0.037 (2)
H7	0.838262	0.769082	0.515696	0.044*
C8	1.129 (2)	0.7670 (3)	0.4742 (3)	0.045 (3)
H8	1.137194	0.791823	0.469475	0.054*
C9	1.295 (2)	0.7446 (3)	0.4539 (3)	0.042 (3)
H9	1.418868	0.753667	0.435565	0.050*
C10	1.2768 (19)	0.7089 (3)	0.4611 (3)	0.041 (3)
H10	1.392056	0.693506	0.447598	0.050*
C11	1.097 (2)	0.6943 (3)	0.4873 (3)	0.038 (2)
H11	1.085696	0.669410	0.491000	0.045*
C12	0.4425 (17)	0.6038 (2)	0.6588 (3)	0.032 (2)
C13	0.6372 (19)	0.5790 (3)	0.6667 (3)	0.045 (3)
H13	0.665689	0.560163	0.647899	0.054*
C14	0.789 (2)	0.5825 (3)	0.7029 (3)	0.047 (3)
H14	0.920793	0.565713	0.709074	0.057*
C15	0.749 (2)	0.6105 (3)	0.7300 (3)	0.053 (3)

H15	0.853723	0.612842	0.754416	0.064*
C16	0.555 (2)	0.6352 (3)	0.7215 (3)	0.054 (3)
H16	0.527355	0.654107	0.740307	0.064*
C17	0.403 (2)	0.6324 (3)	0.6863 (3)	0.045 (3)
H17	0.272841	0.649443	0.680342	0.054*
C18	0.496 (2)	0.5533 (3)	0.4840 (3)	0.037 (2)
C19	0.678 (2)	0.5615 (3)	0.4545 (3)	0.041 (2)
H19	0.810923	0.578321	0.460065	0.049*
C20	0.658 (2)	0.5439 (3)	0.4159 (3)	0.039 (2)
H20	0.781871	0.548077	0.395333	0.047*
C21	0.461 (2)	0.5208 (3)	0.4085 (3)	0.041 (3)
C22	0.282 (2)	0.5129 (3)	0.4390 (3)	0.039 (2)
H22	0.151283	0.495716	0.433775	0.047*
C23	0.297 (2)	0.5302 (3)	0.4770 (3)	0.043 (3)
H23	0.173116	0.526161	0.497540	0.051*
C24	0.630 (2)	0.5065 (3)	0.3421 (3)	0.047 (3)
H24A	0.589789	0.491773	0.317816	0.070*
H24B	0.795072	0.499047	0.354733	0.070*
H24C	0.641228	0.531368	0.333652	0.070*

Atomic displacement parameters (Å^2) for (Aldas3)

	U^{11}	U^{22}	U^{33}	U^{12}	U^{13}	U^{23}
S2	0.0326 (14)	0.0433 (15)	0.0340 (13)	-0.0086 (11)	-0.0004 (10)	0.0042 (11)
O7	0.046 (5)	0.053 (4)	0.037 (4)	-0.012 (4)	0.001 (3)	0.008 (3)
O8	0.031 (4)	0.052 (4)	0.039 (4)	-0.001 (3)	0.005 (3)	0.008 (3)
O9	0.043 (4)	0.045 (4)	0.041 (4)	-0.004 (4)	0.009 (3)	-0.004 (3)
O10	0.039 (4)	0.049 (4)	0.037 (4)	0.002 (3)	-0.003 (3)	0.001 (3)
O11	0.037 (4)	0.056 (4)	0.034 (4)	0.004 (3)	-0.009 (3)	0.004 (3)
O12	0.069 (6)	0.051 (4)	0.039 (4)	-0.017 (4)	0.010 (4)	-0.002 (4)
C25	0.030 (6)	0.052 (6)	0.033 (5)	0.002 (5)	0.007 (4)	0.002 (5)
C26	0.040 (6)	0.030 (5)	0.035 (5)	-0.001 (5)	0.000 (4)	0.009 (4)
C27	0.029 (5)	0.054 (6)	0.021 (5)	-0.002 (5)	0.010 (4)	0.006 (4)
C28	0.030 (6)	0.054 (7)	0.031 (5)	0.008 (5)	0.001 (4)	0.006 (5)
C29	0.023 (5)	0.041 (6)	0.025 (5)	-0.012 (4)	0.004 (4)	0.003 (4)
C30	0.035 (6)	0.046 (6)	0.027 (5)	-0.002 (5)	0.002 (4)	0.004 (4)
C31	0.040 (6)	0.047 (7)	0.035 (6)	0.001 (5)	-0.003 (5)	0.001 (5)
C32	0.041 (6)	0.041 (6)	0.052 (7)	-0.009 (5)	-0.003 (5)	0.006 (5)
C33	0.024 (6)	0.078 (9)	0.044 (6)	0.000 (5)	-0.003 (5)	0.017 (6)

C34	0.034 (6)	0.075 (8)	0.036 (6)	-0.003 (6)	0.001 (5)	0.008 (6)
C35	0.035 (6)	0.045 (6)	0.032 (5)	-0.006 (5)	0.003 (4)	-0.001 (4)
C36	0.032 (6)	0.044 (6)	0.040 (6)	-0.005 (5)	0.001 (4)	0.001 (5)
C37	0.049 (7)	0.035 (6)	0.039 (6)	0.001 (5)	0.003 (5)	-0.002 (5)
C38	0.040 (6)	0.046 (6)	0.045 (6)	-0.002 (5)	-0.009 (5)	0.018 (5)
C39	0.041 (7)	0.063 (7)	0.040 (6)	-0.011 (6)	-0.010 (5)	-0.004 (5)
C40	0.041 (7)	0.063 (7)	0.046 (7)	0.002 (6)	-0.001 (5)	-0.005 (6)
C41	0.038 (6)	0.047 (6)	0.039 (6)	0.008 (5)	0.000 (5)	-0.004 (5)
C42	0.054 (7)	0.036 (5)	0.029 (5)	0.008 (5)	-0.003 (5)	-0.003 (4)
C43	0.051 (7)	0.056 (7)	0.041 (6)	-0.012 (6)	0.016 (5)	-0.009 (5)
C44	0.053 (7)	0.048 (7)	0.048 (7)	-0.013 (6)	0.009 (5)	-0.005 (5)
C45	0.056 (7)	0.042 (6)	0.029 (5)	-0.005 (5)	-0.003 (5)	-0.007 (5)
C46	0.060 (8)	0.064 (8)	0.044 (7)	-0.033 (6)	0.007 (6)	-0.014 (6)
C47	0.061 (8)	0.066 (8)	0.045 (7)	-0.045 (7)	0.019 (6)	-0.014 (6)
C48	0.071 (9)	0.050 (7)	0.041 (6)	-0.003 (6)	0.003 (6)	-0.015 (5)
S3	0.0327 (14)	0.0454 (15)	0.0344 (13)	-0.0079 (11)	0.0010 (10)	0.0003 (11)
O13	0.045 (4)	0.058 (5)	0.039 (4)	-0.011 (4)	0.001 (3)	0.005 (3)
O14	0.032 (4)	0.049 (4)	0.037 (4)	-0.008 (3)	0.006 (3)	0.002 (3)
O15	0.036 (4)	0.051 (4)	0.046 (4)	0.001 (4)	0.004 (3)	-0.001 (4)
O16	0.038 (4)	0.048 (4)	0.033 (4)	0.000 (3)	0.001 (3)	-0.004 (3)
O17	0.037 (4)	0.050 (4)	0.041 (4)	0.002 (3)	-0.003 (3)	-0.006 (3)
O18	0.048 (5)	0.052 (5)	0.040 (4)	-0.005 (4)	0.006 (3)	-0.012 (3)
C49	0.016 (5)	0.047 (6)	0.030 (5)	-0.005 (4)	0.006 (4)	-0.010 (4)
C50	0.033 (6)	0.037 (5)	0.033 (5)	0.000 (5)	0.004 (4)	0.010 (4)
C51	0.030 (5)	0.052 (6)	0.027 (5)	-0.003 (5)	0.006 (4)	-0.001 (4)
C52	0.024 (5)	0.045 (6)	0.039 (5)	0.011 (4)	-0.001 (4)	0.004 (5)
C53	0.015 (5)	0.059 (7)	0.030 (5)	-0.006 (5)	0.001 (4)	0.013 (5)
C54	0.027 (5)	0.050 (6)	0.025 (5)	-0.003 (5)	0.000 (4)	0.007 (4)
C55	0.040 (6)	0.043 (6)	0.035 (6)	-0.004 (5)	-0.005 (5)	0.002 (5)
C56	0.037 (6)	0.055 (7)	0.046 (6)	-0.010 (5)	-0.001 (5)	0.026 (5)
C57	0.034 (6)	0.086 (9)	0.040 (6)	-0.009 (6)	-0.006 (5)	0.026 (6)
C58	0.036 (6)	0.074 (9)	0.039 (6)	0.006 (6)	-0.001 (5)	0.004 (6)
C59	0.027 (6)	0.051 (6)	0.044 (6)	-0.007 (5)	0.007 (4)	0.004 (5)
C60	0.024 (5)	0.055 (7)	0.038 (6)	-0.017 (5)	-0.001 (4)	0.002 (5)
C61	0.042 (6)	0.037 (6)	0.045 (6)	-0.004 (5)	0.004 (5)	0.005 (5)
C62	0.047 (7)	0.059 (7)	0.044 (6)	0.005 (6)	-0.009 (5)	0.012 (6)
C63	0.052 (7)	0.058 (7)	0.030 (5)	-0.008 (6)	-0.003 (5)	0.003 (5)
C64	0.039 (6)	0.050 (6)	0.045 (6)	0.008 (5)	0.003 (5)	-0.008 (5)

C65	0.032 (6)	0.047 (6)	0.042 (6)	-0.002 (5)	-0.008 (4)	0.004 (5)
C66	0.041 (6)	0.036 (5)	0.024 (5)	0.004 (5)	-0.003 (4)	-0.010 (4)
C67	0.074 (9)	0.053 (7)	0.039 (6)	-0.028 (6)	0.021 (6)	-0.014 (5)
C68	0.048 (7)	0.057 (7)	0.047 (7)	-0.017 (6)	0.006 (5)	-0.012 (5)
C69	0.053 (7)	0.039 (5)	0.020 (5)	-0.004 (5)	0.005 (4)	-0.007 (4)
C70	0.043 (7)	0.070 (8)	0.032 (6)	-0.009 (6)	0.005 (5)	-0.005 (5)
C71	0.056 (7)	0.051 (7)	0.039 (6)	-0.007 (6)	0.005 (5)	0.000 (5)
C72	0.053 (7)	0.041 (6)	0.055 (7)	-0.003 (5)	-0.001 (5)	-0.010 (5)
S1	0.0323 (14)	0.0425 (14)	0.0326 (13)	-0.0023 (11)	0.0003 (10)	0.0030 (11)
O1	0.028 (4)	0.050 (4)	0.041 (4)	-0.008 (3)	-0.001 (3)	0.005 (3)
O2	0.035 (4)	0.039 (4)	0.034 (4)	-0.004 (3)	0.005 (3)	0.009 (3)
O3	0.044 (4)	0.039 (4)	0.040 (4)	0.008 (3)	0.007 (3)	-0.001 (3)
O4	0.039 (4)	0.045 (4)	0.034 (4)	0.005 (3)	0.001 (3)	-0.005 (3)
O5	0.042 (4)	0.047 (4)	0.036 (4)	-0.001 (3)	0.001 (3)	-0.003 (3)
O6	0.053 (5)	0.048 (4)	0.027 (4)	-0.006 (3)	0.006 (3)	-0.009 (3)
C1	0.026 (5)	0.052 (6)	0.034 (5)	-0.004 (5)	0.005 (4)	0.005 (5)
C2	0.038 (6)	0.035 (5)	0.032 (5)	-0.007 (5)	0.000 (5)	0.011 (4)
C3	0.026 (5)	0.048 (6)	0.029 (5)	-0.005 (4)	0.009 (4)	0.001 (4)
C4	0.039 (6)	0.041 (6)	0.024 (5)	-0.004 (4)	0.009 (4)	-0.005 (4)
C5	0.029 (6)	0.052 (6)	0.033 (5)	-0.005 (5)	0.003 (4)	0.007 (5)
C6	0.026 (5)	0.047 (6)	0.028 (5)	-0.010 (4)	-0.006 (4)	0.005 (4)
C7	0.045 (6)	0.030 (5)	0.036 (5)	-0.001 (4)	0.004 (5)	0.006 (4)
C8	0.028 (6)	0.037 (6)	0.070 (7)	-0.008 (5)	0.002 (5)	0.008 (5)
C9	0.034 (6)	0.056 (7)	0.036 (6)	-0.007 (5)	-0.007 (4)	0.005 (5)
C10	0.023 (5)	0.052 (7)	0.049 (6)	-0.004 (5)	0.004 (5)	0.000 (5)
C11	0.037 (6)	0.041 (6)	0.036 (5)	-0.005 (5)	0.004 (4)	-0.002 (4)
C12	0.021 (5)	0.037 (5)	0.040 (5)	-0.002 (4)	0.005 (4)	0.005 (4)
C13	0.029 (6)	0.062 (7)	0.044 (6)	-0.007 (5)	0.011 (5)	-0.006 (5)
C14	0.040 (6)	0.056 (7)	0.046 (6)	-0.009 (5)	-0.002 (5)	0.009 (6)
C15	0.046 (7)	0.068 (8)	0.046 (6)	-0.028 (6)	-0.005 (5)	0.002 (6)
C16	0.072 (9)	0.057 (7)	0.032 (6)	-0.010 (6)	0.009 (6)	-0.008 (5)
C17	0.055 (7)	0.050 (7)	0.029 (5)	-0.005 (5)	0.008 (5)	-0.002 (5)
C18	0.043 (6)	0.035 (5)	0.032 (5)	0.010 (5)	0.003 (4)	0.008 (4)
C19	0.039 (6)	0.046 (6)	0.038 (6)	-0.005 (5)	-0.002 (5)	-0.001 (5)
C20	0.038 (6)	0.042 (6)	0.038 (6)	0.004 (5)	0.012 (4)	0.000 (5)
C21	0.044 (7)	0.041 (6)	0.037 (6)	0.001 (5)	-0.009 (5)	0.006 (5)
C22	0.043 (6)	0.036 (5)	0.038 (6)	0.000 (5)	0.000 (5)	0.001 (4)
C23	0.036 (6)	0.057 (7)	0.036 (6)	0.006 (5)	0.003 (4)	-0.012 (5)

C24	0.052 (7)	0.055 (7)	0.033 (5)	0.000 (6)	0.005 (5)	0.002 (5)

Geometric parameters (Å, º) for (Aldas3)

S2—O7	1.416 (8)	C58—C59	1.415 (15)
S2—O8	1.431 (7)	C58—H58	0.9500
S2—C36	1.736 (10)	C59—H59	0.9500
S2—C25	1.791 (10)	C60—C61	1.372 (15)
O9—C29	1.224 (11)	C60—C65	1.407 (14)
O10—C26	1.345 (12)	C61—C62	1.405 (14)
O10—C42	1.412 (11)	C61—H61	0.9500
O11—C26	1.204 (11)	C62—C63	1.389 (15)
O12—C45	1.353 (12)	C62—H62	0.9500
O12—C48	1.444 (13)	C63—C64	1.371 (15)
C25—C27	1.513 (14)	C63—H63	0.9500
C25—C26	1.521 (13)	C64—C65	1.401 (14)
C25—H25	1.0000	C64—H64	0.9500
C27—C28	1.539 (13)	C65—H65	0.9500
C27—H27A	0.9900	C66—C67	1.360 (15)
C27—H27B	0.9900	C66—C71	1.385 (15)
C28—C29	1.484 (14)	C67—C68	1.375 (14)
C28—H28A	0.9900	C67—H67	0.9500
C28—H28B	0.9900	C68—C69	1.404 (15)
C29—C30	1.486 (13)	C68—H68	0.9500
C30—C31	1.364 (14)	C69—C70	1.377 (15)
C30—C35	1.387 (14)	C70—C71	1.377 (14)
C31—C32	1.411 (15)	C70—H70	0.9500
C31—H31	0.9500	C71—H71	0.9500
C32—C33	1.370 (16)	C72—H72A	0.9800
C32—H32	0.9500	C72—H72B	0.9800
C33—C34	1.360 (16)	C72—H72C	0.9800
C33—H33	0.9500	S1—O1	1.406 (7)
C34—C35	1.397 (14)	S1—O2	1.424 (7)
C34—H34	0.9500	S1—C12	1.748 (9)
C35—H35	0.9500	S1—C1	1.788 (9)
C36—C37	1.384 (14)	O3—C5	1.228 (12)
C36—C41	1.404 (14)	O4—C2	1.337 (12)
C37—C38	1.372 (14)	O4—C18	1.417 (12)
C37—H37	0.9500	O5—C2	1.193 (12)
C38—C39	1.381 (15)	O6—C21	1.367 (12)

C38—H38	0.9500	O6—C24	1.452 (12)
C39—C40	1.402 (16)	C1—C3	1.489 (14)
C39—H39	0.9500	C1—C2	1.538 (13)
C40—C41	1.377 (14)	C1—H1	1.0000
C40—H40	0.9500	C3—C4	1.540 (12)
C41—H41	0.9500	C3—H3A	0.9900
C42—C47	1.361 (15)	C3—H3B	0.9900
C42—C43	1.390 (15)	C4—C5	1.506 (14)
C43—C44	1.381 (14)	C4—H4A	0.9900
C43—H43	0.9500	C4—H4B	0.9900
C44—C45	1.352 (15)	C5—C6	1.493 (14)
C44—H44	0.9500	C6—C7	1.372 (14)
C45—C46	1.396 (15)	C6—C11	1.409 (14)
C46—C47	1.367 (14)	C7—C8	1.400 (14)
C46—H46	0.9500	C7—H7	0.9500
C47—H47	0.9500	C8—C9	1.387 (15)
C48—H48A	0.9800	C8—H8	0.9500
C48—H48B	0.9800	C9—C10	1.369 (15)
C48—H48C	0.9800	C9—H9	0.9500
S3—O14	1.415 (7)	C10—C11	1.389 (14)
S3—O13	1.421 (8)	C10—H10	0.9500
S3—C60	1.770 (10)	C11—H11	0.9500
S3—C49	1.786 (9)	C12—C13	1.397 (14)
O15—C53	1.221 (12)	C12—C17	1.414 (14)
O16—C50	1.335 (12)	C13—C14	1.398 (14)
O16—C66	1.397 (11)	C13—H13	0.9500
O17—C50	1.189 (11)	C14—C15	1.391 (16)
O18—C69	1.365 (11)	C14—H14	0.9500
O18—C72	1.404 (13)	C15—C16	1.394 (17)
C49—C50	1.502 (13)	C15—H15	0.9500
C49—C51	1.522 (13)	C16—C17	1.368 (15)
C49—H49	1.0000	C16—H16	0.9500
C51—C52	1.530 (13)	C17—H17	0.9500
C51—H51A	0.9900	C18—C23	1.365 (15)
C51—H51B	0.9900	C18—C19	1.397 (14)
C52—C53	1.496 (14)	C19—C20	1.412 (13)
C52—H52A	0.9900	C19—H19	0.9500
C52—H52B	0.9900	C20—C21	1.361 (15)
C53—C54	1.503 (13)	C20—H20	0.9500

C54—C55	1.374 (14)	C21—C22	1.407 (15)
C54—C59	1.402 (14)	C22—C23	1.391 (13)
C55—C56	1.397 (15)	C22—H22	0.9500
C55—H55	0.9500	C23—H23	0.9500
C56—C57	1.355 (16)	C24—H24A	0.9800
C56—H56	0.9500	C24—H24B	0.9800
C57—C58	1.374 (17)	C24—H24C	0.9800
C57—H57	0.9500		
O7—S2—O8	119.2 (5)	C59—C58—H58	119.8
O7—S2—C36	108.5 (5)	C54—C59—C58	118.1 (10)
O8—S2—C36	108.7 (5)	C54—C59—H59	120.9
O7—S2—C25	106.8 (5)	C58—C59—H59	120.9
O8—S2—C25	108.8 (4)	C61—C60—C65	123.8 (9)
C36—S2—C25	103.8 (5)	C61—C60—S3	118.4 (8)
C26—O10—C42	116.8 (8)	C65—C60—S3	117.7 (8)
C45—O12—C48	118.5 (8)	C60—C61—C62	117.6 (10)
C27—C25—C26	111.9 (8)	C60—C61—H61	121.2
C27—C25—S2	112.3 (7)	C62—C61—H61	121.2
C26—C25—S2	106.1 (7)	C63—C62—C61	120.4 (10)
C27—C25—H25	108.8	C63—C62—H62	119.8
C26—C25—H25	108.8	C61—C62—H62	119.8
S2—C25—H25	108.8	C64—C63—C62	120.3 (10)
O11—C26—O10	125.0 (9)	C64—C63—H63	119.9
O11—C26—C25	125.2 (9)	C62—C63—H63	119.9
O10—C26—C25	109.8 (8)	C63—C64—C65	121.7 (10)
C25—C27—C28	112.1 (8)	C63—C64—H64	119.1
C25—C27—H27A	109.2	C65—C64—H64	119.1
C28—C27—H27A	109.2	C64—C65—C60	116.1 (9)
C25—C27—H27B	109.2	C64—C65—H65	121.9
C28—C27—H27B	109.2	C60—C65—H65	121.9
H27A—C27—H27B	107.9	C67—C66—C71	120.1 (9)
C29—C28—C27	114.5 (8)	C67—C66—O16	121.6 (9)
C29—C28—H28A	108.6	C71—C66—O16	118.3 (9)
C27—C28—H28A	108.6	C66—C67—C68	121.2 (10)
C29—C28—H28B	108.6	C66—C67—H67	119.4
C27—C28—H28B	108.6	C68—C67—H67	119.4
H28A—C28—H28B	107.6	C67—C68—C69	119.0 (10)
O9—C29—C28	120.1 (8)	C67—C68—H68	120.5

O9—C29—C30	120.4 (9)	C69—C68—H68	120.5
C28—C29—C30	119.4 (9)	O18—C69—C70	116.7 (9)
C31—C30—C35	119.8 (9)	O18—C69—C68	123.9 (9)
C31—C30—C29	119.5 (9)	C70—C69—C68	119.3 (9)
C35—C30—C29	120.7 (9)	C69—C70—C71	120.7 (10)
C30—C31—C32	120.5 (10)	C69—C70—H70	119.6
C30—C31—H31	119.8	C71—C70—H70	119.6
C32—C31—H31	119.8	C70—C71—C66	119.5 (10)
C33—C32—C31	118.5 (10)	C70—C71—H71	120.2
C33—C32—H32	120.8	C66—C71—H71	120.2
C31—C32—H32	120.8	O18—C72—H72A	109.5
C34—C33—C32	121.9 (11)	O18—C72—H72B	109.5
C34—C33—H33	119.1	H72A—C72—H72B	109.5
C32—C33—H33	119.1	O18—C72—H72C	109.5
C33—C34—C35	119.4 (11)	H72A—C72—H72C	109.5
C33—C34—H34	120.3	H72B—C72—H72C	109.5
C35—C34—H34	120.3	O1—S1—O2	119.0 (4)
C30—C35—C34	119.9 (10)	O1—S1—C12	108.4 (4)
C30—C35—H35	120.0	O2—S1—C12	108.6 (4)
C34—C35—H35	120.0	O1—S1—C1	108.1 (4)
C37—C36—C41	118.6 (9)	O2—S1—C1	108.8 (4)
C37—C36—S2	120.8 (8)	C12—S1—C1	102.8 (5)
C41—C36—S2	120.6 (8)	C2—O4—C18	118.2 (7)
C38—C37—C36	121.4 (10)	C21—O6—C24	116.4 (8)
C38—C37—H37	119.3	C3—C1—C2	111.3 (8)
C36—C37—H37	119.3	C3—C1—S1	112.9 (7)
C37—C38—C39	120.6 (10)	C2—C1—S1	108.5 (7)
C37—C38—H38	119.7	C3—C1—H1	108.0
C39—C38—H38	119.7	C2—C1—H1	108.0
C38—C39—C40	118.7 (10)	S1—C1—H1	108.0
C38—C39—H39	120.7	O5—C2—O4	125.0 (9)
C40—C39—H39	120.7	O5—C2—C1	124.3 (9)
C41—C40—C39	120.9 (11)	O4—C2—C1	110.6 (8)
C41—C40—H40	119.5	C1—C3—C4	112.1 (8)
C39—C40—H40	119.5	C1—C3—H3A	109.2
C40—C41—C36	119.8 (10)	C4—C3—H3A	109.2
C40—C41—H41	120.1	C1—C3—H3B	109.2
C36—C41—H41	120.1	C4—C3—H3B	109.2
C47—C42—C43	119.3 (10)	H3A—C3—H3B	107.9

C47—C42—O10	121.4 (9)	C5—C4—C3	114.1 (8)
C43—C42—O10	118.8 (10)	C5—C4—H4A	108.7
C44—C43—C42	118.1 (11)	C3—C4—H4A	108.7
C44—C43—H43	120.9	C5—C4—H4B	108.7
C42—C43—H43	120.9	C3—C4—H4B	108.7
C45—C44—C43	122.6 (11)	H4A—C4—H4B	107.6
C45—C44—H44	118.7	O3—C5—C6	120.4 (9)
C43—C44—H44	118.7	O3—C5—C4	120.6 (9)
C44—C45—O12	117.0 (10)	C6—C5—C4	119.0 (9)
C44—C45—C46	118.8 (10)	C7—C6—C11	120.2 (9)
O12—C45—C46	124.2 (10)	C7—C6—C5	119.4 (10)
C47—C46—C45	118.9 (10)	C11—C6—C5	120.4 (9)
C47—C46—H46	120.5	C6—C7—C8	120.1 (10)
C45—C46—H46	120.5	C6—C7—H7	119.9
C42—C47—C46	122.1 (10)	C8—C7—H7	119.9
C42—C47—H47	118.9	C9—C8—C7	120.6 (10)
C46—C47—H47	118.9	C9—C8—H8	119.7
O12—C48—H48A	109.5	C7—C8—H8	119.7
O12—C48—H48B	109.5	C10—C9—C8	118.3 (10)
H48A—C48—H48B	109.5	C10—C9—H9	120.9
O12—C48—H48C	109.5	C8—C9—H9	120.9
H48A—C48—H48C	109.5	C9—C10—C11	123.0 (10)
H48B—C48—H48C	109.5	C9—C10—H10	118.5
O14—S3—O13	119.3 (4)	C11—C10—H10	118.5
O14—S3—C60	109.2 (5)	C10—C11—C6	117.8 (9)
O13—S3—C60	108.5 (5)	C10—C11—H11	121.1
O14—S3—C49	109.0 (4)	C6—C11—H11	121.1
O13—S3—C49	106.4 (4)	C13—C12—C17	120.9 (9)
C60—S3—C49	103.2 (4)	C13—C12—S1	118.6 (8)
C50—O16—C66	117.0 (8)	C17—C12—S1	120.4 (7)
C69—O18—C72	117.8 (8)	C12—C13—C14	118.5 (10)
C50—C49—C51	111.4 (8)	C12—C13—H13	120.7
C50—C49—S3	109.2 (6)	C14—C13—H13	120.7
C51—C49—S3	113.2 (6)	C15—C14—C13	120.3 (11)
C50—C49—H49	107.6	C15—C14—H14	119.8
C51—C49—H49	107.6	C13—C14—H14	119.8
S3—C49—H49	107.6	C14—C15—C16	120.4 (10)
O17—C50—O16	125.3 (9)	C14—C15—H15	119.8
O17—C50—C49	124.7 (9)	C16—C15—H15	119.8

O16—C50—C49	110.0 (8)	C17—C16—C15	120.5 (11)
C49—C51—C52	110.2 (8)	C17—C16—H16	119.8
C49—C51—H51A	109.6	C15—C16—H16	119.8
C52—C51—H51A	109.6	C16—C17—C12	119.4 (11)
C49—C51—H51B	109.6	C16—C17—H17	120.3
C52—C51—H51B	109.6	C12—C17—H17	120.3
H51A—C51—H51B	108.1	C23—C18—C19	123.5 (10)
C53—C52—C51	114.4 (8)	C23—C18—O4	120.7 (9)
C53—C52—H52A	108.7	C19—C18—O4	115.7 (9)
C51—C52—H52A	108.7	C18—C19—C20	117.5 (10)
C53—C52—H52B	108.7	C18—C19—H19	121.2
C51—C52—H52B	108.7	C20—C19—H19	121.2
H52A—C52—H52B	107.6	C21—C20—C19	119.7 (10)
O15—C53—C52	121.9 (8)	C21—C20—H20	120.2
O15—C53—C54	120.8 (9)	C19—C20—H20	120.2
C52—C53—C54	117.2 (9)	C20—C21—O6	123.3 (10)
C55—C54—C59	120.5 (9)	C20—C21—C22	121.4 (10)
C55—C54—C53	118.7 (9)	O6—C21—C22	115.2 (9)
C59—C54—C53	120.9 (9)	C23—C22—C21	119.6 (10)
C54—C55—C56	119.6 (10)	C23—C22—H22	120.2
C54—C55—H55	120.2	C21—C22—H22	120.2
C56—C55—H55	120.2	C18—C23—C22	118.2 (10)
C57—C56—C55	121.0 (10)	C18—C23—H23	120.9
C57—C56—H56	119.5	C22—C23—H23	120.9
C55—C56—H56	119.5	O6—C24—H24A	109.5
C56—C57—C58	120.3 (10)	O6—C24—H24B	109.5
C56—C57—H57	119.8	H24A—C24—H24B	109.5
C58—C57—H57	119.8	O6—C24—H24C	109.5
C57—C58—C59	120.5 (11)	H24A—C24—H24C	109.5
C57—C58—H58	119.8	H24B—C24—H24C	109.5
O7—S2—C25—C27	166.8 (7)	O14—S3—C60—C61	-167.0 (8)
O8—S2—C25—C27	36.9 (8)	O13—S3—C60—C61	-35.4 (9)
C36—S2—C25—C27	-78.6 (8)	C49—S3—C60—C61	77.1 (9)
O7—S2—C25—C26	44.3 (8)	O14—S3—C60—C65	12.5 (9)
O8—S2—C25—C26	-85.6 (7)	O13—S3—C60—C65	144.1 (8)
C36—S2—C25—C26	158.8 (7)	C49—S3—C60—C65	-103.4 (8)

C42—O10—C26—O11	-3.6 (14)	C65—C60—C61—C62	1.6 (16)
C42—O10—C26—C25	177.3 (8)	S3—C60—C61—C62	-178.9 (8)
C27—C25—C26—O11	-44.9 (13)	C60—C61—C62—C63	-0.2 (16)
S2—C25—C26—O11	77.9 (11)	C61—C62—C63—C64	-1.6 (17)
C27—C25—C26—O10	134.3 (8)	C62—C63—C64—C65	2.1 (17)
S2—C25—C26—O10	-103.0 (8)	C63—C64—C65—C60	-0.7 (16)
C26—C25—C27—C28	-63.0 (11)	C61—C60—C65—C64	-1.2 (15)
S2—C25—C27—C28	177.8 (7)	S3—C60—C65—C64	179.3 (8)
C25—C27—C28—C29	-177.8 (8)	C50—O16—C66—C67	71.4 (13)
C27—C28—C29—O9	-6.6 (13)	C50—O16—C66—C71	-108.9 (11)
C27—C28—C29—C30	176.4 (8)	C71—C66—C67—C68	-3.1 (19)
O9—C29—C30—C31	-1.5 (14)	O16—C66—C67—C68	176.6 (10)
C28—C29—C30—C31	175.4 (9)	C66—C67—C68—C69	4.6 (19)
O9—C29—C30—C35	177.0 (9)	C72—O18—C69—C70	178.4 (10)
C28—C29—C30—C35	-6.0 (14)	C72—O18—C69—C68	-3.4 (16)
C35—C30—C31—C32	1.7 (15)	C67—C68—C69—O18	177.4 (11)
C29—C30—C31—C32	-179.8 (9)	C67—C68—C69—C70	-4.4 (18)
C30—C31—C32—C33	-0.7 (15)	O18—C69—C70—C71	-178.9 (10)
C31—C32—C33—C34	0.0 (16)	C68—C69—C70—C71	2.8 (18)
C32—C33—C34—C35	-0.3 (16)	C69—C70—C71—C66	-1.3 (18)
C31—C30—C35—C34	-2.0 (15)	C67—C66—C71—C70	1.4 (17)

C29—C30—C35—C34	179.5 (9)	O16—C66—C71—C70	-178.3 (10)
C33—C34—C35—C30	1.3 (15)	O1—S1—C1—C3	165.5 (7)
O7—S2—C36—C37	38.7 (10)	O2—S1—C1—C3	34.9 (8)
O8—S2—C36—C37	169.7 (8)	C12—S1—C1—C3	-80.1 (8)
C25—S2—C36—C37	-74.7 (9)	O1—S1—C1—C2	41.6 (8)
O7—S2—C36—C41	-139.4 (8)	O2—S1—C1—C2	-89.0 (7)
O8—S2—C36—C41	-8.4 (10)	C12—S1—C1—C2	156.0 (7)
C25—S2—C36—C41	107.2 (9)	C18—O4—C2—O5	6.8 (14)
C41—C36—C37—C38	-0.2 (16)	C18—O4—C2—C1	-174.0 (8)
S2—C36—C37—C38	-178.4 (8)	C3—C1—C2—O5	-48.8 (13)
C36—C37—C38—C39	-0.3 (16)	S1—C1—C2—O5	76.0 (11)
C37—C38—C39—C40	0.1 (16)	C3—C1—C2—O4	132.0 (8)
C38—C39—C40—C41	0.7 (17)	S1—C1—C2—O4	-103.2 (8)
C39—C40—C41—C36	-1.3 (17)	C2—C1—C3—C4	-62.3 (11)
C37—C36—C41—C40	1.0 (16)	S1—C1—C3—C4	175.4 (7)
S2—C36—C41—C40	179.2 (9)	C1—C3—C4—C5	-179.3 (8)
C26—O10—C42—C47	-77.2 (13)	C3—C4—C5—O3	-1.2 (13)
C26—O10—C42—C43	110.7 (11)	C3—C4—C5—C6	177.8 (8)
C47—C42—C43—C44	2.4 (18)	O3—C5—C6—C7	-6.6 (14)
O10—C42—C43—C44	174.7 (10)	C4—C5—C6—C7	174.4 (9)
C42—C43—C44—C45	-0.8 (18)	O3—C5—C6—C11	175.7 (9)
C43—C44—C45—O12	-179.7 (11)	C4—C5—C6—C11	-3.3 (14)
C43—C44—C45—C46	-1.8 (19)	C11—C6—C7—C8	0.1 (15)

C48—O12—C45—C44	175.7 (10)	C5—C6—C7—C8	-177.7 (9)
C48—O12—C45—C46	-2.1 (17)	C6—C7—C8—C9	-1.1 (16)
C44—C45—C46—C47	2.7 (19)	C7—C8—C9—C10	0.7 (15)
O12—C45—C46—C47	-179.5 (12)	C8—C9—C10—C11	0.9 (15)
C43—C42—C47—C46	-1 (2)	C9—C10—C11—C6	-1.9 (15)
O10—C42—C47—C46	-173.5 (11)	C7—C6—C11—C10	1.4 (14)
C45—C46—C47—C42	-1 (2)	C5—C6—C11—C10	179.1 (9)
O14—S3—C49—C50	86.7 (7)	O1—S1—C12—C13	41.4 (9)
O13—S3—C49—C50	-43.2 (8)	O2—S1—C12—C13	172.0 (7)
C60—S3—C49—C50	-157.3 (7)	C1—S1—C12—C13	-72.8 (9)
O14—S3—C49—C51	-38.0 (8)	O1—S1—C12—C17	-141.1 (8)
O13—S3—C49—C51	-167.8 (7)	O2—S1—C12—C17	-10.5 (9)
C60—S3—C49—C51	78.1 (8)	C1—S1—C12—C17	104.7 (8)
C66—O16—C50—O17	-1.1 (14)	C17—C12—C13—C14	1.4 (15)
C66—O16—C50—C49	179.4 (7)	S1—C12—C13—C14	178.9 (8)
C51—C49—C50—O17	55.8 (12)	C12—C13—C14—C15	-1.0 (15)
S3—C49—C50—O17	-69.9 (11)	C13—C14—C15—C16	0.6 (16)
C51—C49—C50—O16	-124.7 (8)	C14—C15—C16—C17	-0.6 (17)
S3—C49—C50—O16	109.6 (8)	C15—C16—C17—C12	1.0 (16)
C50—C49—C51—C52	59.5 (10)	C13—C12—C17—C16	-1.4 (15)
S3—C49—C51—C52	-177.0 (7)	S1—C12—C17—C16	-178.9 (8)

C49—C51—C52—C53	177.5 (7)	C2—O4—C18—C23	-79.0 (11)
C51—C52—C53—O15	3.9 (13)	C2—O4—C18—C19	103.4 (10)
C51—C52—C53—C54	-175.1 (8)	C23—C18—C19—C20	-2.1 (16)
O15—C53—C54—C55	-3.1 (14)	O4—C18—C19—C20	175.5 (8)
C52—C53—C54—C55	175.9 (8)	C18—C19—C20—C21	2.1 (15)
O15—C53—C54—C59	176.9 (9)	C19—C20—C21—O6	-179.8 (9)
C52—C53—C54—C59	-4.1 (13)	C19—C20—C21—C22	-2.9 (16)
C59—C54—C55—C56	-0.4 (14)	C24—O6—C21—C20	5.3 (14)
C53—C54—C55—C56	179.6 (9)	C24—O6—C21—C22	-171.8 (9)
C54—C55—C56—C57	1.0 (15)	C20—C21—C22—C23	3.5 (16)
C55—C56—C57—C58	-1.5 (16)	O6—C21—C22—C23	-179.4 (9)
C56—C57—C58—C59	1.4 (16)	C19—C18—C23—C22	2.7 (16)
C55—C54—C59—C58	0.3 (14)	O4—C18—C23—C22	-174.7 (8)
C53—C54—C59—C58	-179.7 (9)	C21—C22—C23—C18	-3.3 (15)
C57—C58—C59—C54	-0.8 (15)		

Compound 16d

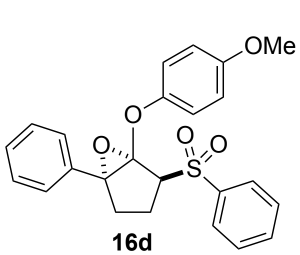

16d

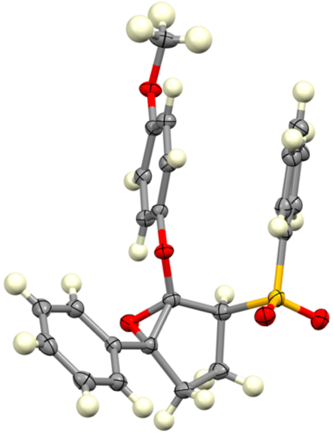

Sample Name: CCDC 2517781 / AA1199P

Crystal Growth: Slow evaporation using a mixture of hexanes and dichloromethane

Crystal data

$C_{24}H_{22}O_5S$	*F*(000) = 888
M_r = 422.47	D_x = 1.387 Mg m^{-3}
Monoclinic, *P*2$_1$/*c*	Cu *K*a radiation, l = 1.54184 Å
a = 14.0777 (5) Å	Cell parameters from 9866 reflections
b = 11.9003 (4) Å	q = 3.6–79.3°
c = 13.6909 (5) Å	m = 1.71 mm^{-1}
b = 118.096 (2)°	*T* = 100 K
V = 2023.34 (13) Å^3	Lath, colourless
Z = 4	0.24 × 0.09 × 0.05 mm

Data collection

Bruker D8 Venture DUO with Photon III C14 diffractometer	3668 reflections with *I* > 2s(*I*)
Radiation source: ImS 3.0 microfocus	R_{int} = 0.061
f and w scans	q_{max} = 80.3°, q_{min} = 3.6°
Absorption correction: multi-scan *SADABS* (Krause *et al.*, 2015)	*h* = -17 - 17
T_{min} = 0.753, T_{max} = 0.919	*k* = -12 - 14
45808 measured reflections	*l* = -17 - 17
4325 independent reflections	

Refinement

Refinement on F^2	0 restraints
Least-squares matrix: full	Hydrogen site location: inferred from neighbouring sites
$R[F^2 > 2s(F^2)] = 0.037$	H-atom parameters constrained
$wR(F^2) = 0.095$	$w = 1/[s^2(F_o^2) + (0.0417P)^2 + 1.116P]$ where $P = (F_o^2 + 2F_c^2)/3$
$S = 1.04$	$(D/s)_{max} = 0.001$
4325 reflections	$Dr_{max} = 0.29$ e Å^{-3}
272 parameters	$Dr_{min} = -0.44$ e Å^{-3}

Fractional atomic coordinates and isotropic or equivalent isotropic displacement parameters (Å^2) for (AA1199P)

	x	*y*	*z*	U_{iso}*/U_{eq}
S1	0.36033 (3)	0.75455 (3)	0.47765 (3)	0.02309 (11)
O1	0.07369 (8)	0.59572 (9)	0.32435 (9)	0.0236 (2)
O2	0.24648 (8)	0.51460 (9)	0.44181 (8)	0.0214 (2)
O3	0.38299 (9)	0.71606 (10)	0.39092 (9)	0.0290 (3)
O4	0.37538 (10)	0.87169 (10)	0.50708 (10)	0.0344 (3)
O5	0.23230 (9)	0.39906 (9)	0.82716 (9)	0.0263 (2)
C1	0.18795 (12)	0.60724 (12)	0.38459 (12)	0.0210 (3)
C2	0.22109 (13)	0.72365 (13)	0.43481 (13)	0.0231 (3)
H2	0.203954	0.734569	0.497242	0.028*
C3	0.15189 (14)	0.80205 (13)	0.33642 (13)	0.0275 (3)
H3A	0.083461	0.821065	0.336500	0.033*
H3B	0.191177	0.872536	0.341115	0.033*
C4	0.12980 (14)	0.73624 (13)	0.23073 (13)	0.0261 (3)
H4A	0.057953	0.755173	0.169283	0.031*
H4B	0.185072	0.753248	0.207530	0.031*
C5	0.13478 (12)	0.61376 (13)	0.26314 (12)	0.0218 (3)
C6	0.13086 (12)	0.52003 (13)	0.18951 (12)	0.0224 (3)
C7	0.11324 (13)	0.41003 (13)	0.21202 (13)	0.0262 (3)
H7	0.103123	0.394694	0.274599	0.031*
C8	0.11040 (14)	0.32289 (14)	0.14348 (15)	0.0312 (4)
H8	0.097159	0.248297	0.158674	0.037*
C9	0.12673 (13)	0.34386 (15)	0.05302 (15)	0.0324 (4)
H9	0.126668	0.283546	0.007536	0.039*
C10	0.14313 (14)	0.45273 (16)	0.02900 (14)	0.0316 (4)
H10	0.153260	0.467431	-0.033678	0.038*

C11	0.14479 (13)	0.54050 (14)	0.09650 (13)	0.0266 (3)
H11	0.155484	0.615249	0.079334	0.032*
C12	0.43922 (12)	0.67611 (12)	0.59813 (12)	0.0227 (3)
C13	0.42877 (13)	0.69196 (13)	0.69339 (13)	0.0251 (3)
H13	0.377904	0.744005	0.694042	0.030*
C14	0.49404 (13)	0.63030 (14)	0.78727 (13)	0.0282 (3)
H14	0.486672	0.638551	0.852401	0.034*
C15	0.57004 (14)	0.55664 (14)	0.78652 (14)	0.0289 (3)
H15	0.614381	0.514685	0.851204	0.035*
C16	0.58185 (13)	0.54369 (14)	0.69202 (14)	0.0280 (3)
H16	0.635270	0.494444	0.692692	0.034*
C17	0.51549 (13)	0.60282 (13)	0.59664 (13)	0.0252 (3)
H17	0.522041	0.593384	0.531165	0.030*
C18	0.23918 (12)	0.48545 (12)	0.53728 (12)	0.0207 (3)
C19	0.15436 (12)	0.51831 (12)	0.55650 (13)	0.0224 (3)
H19	0.096665	0.561754	0.503063	0.027*
C20	0.15505 (12)	0.48693 (12)	0.65446 (12)	0.0224 (3)
H20	0.097395	0.508874	0.667972	0.027*
C21	0.23956 (12)	0.42355 (12)	0.73303 (12)	0.0215 (3)
C22	0.32230 (13)	0.38825 (13)	0.71193 (13)	0.0243 (3)
H22	0.379187	0.343275	0.764455	0.029*
C23	0.32129 (12)	0.41923 (13)	0.61334 (13)	0.0232 (3)
H23	0.377328	0.394697	0.598338	0.028*
C24	0.32001 (15)	0.33805 (17)	0.91131 (14)	0.0366 (4)
H24A	0.307933	0.328686	0.975727	0.055*
H24B	0.387148	0.379556	0.933185	0.055*
H24C	0.325135	0.264032	0.882757	0.055*

Atomic displacement parameters (Å^2) for (AA1199P)

	U^{11}	U^{22}	U^{33}	U^{12}	U^{13}	U^{23}
S1	0.0276 (2)	0.02138 (19)	0.02077 (19)	-0.00315 (14)	0.01173 (15)	-0.00054 (13)
O1	0.0211 (5)	0.0292 (6)	0.0220 (5)	0.0005 (4)	0.0113 (4)	0.0024 (4)
O2	0.0250 (5)	0.0218 (5)	0.0196 (5)	0.0025 (4)	0.0121 (4)	0.0034 (4)
O3	0.0327 (6)	0.0354 (6)	0.0236 (6)	-0.0039 (5)	0.0171 (5)	0.0006 (5)
O4	0.0426 (7)	0.0215 (6)	0.0328 (6)	-0.0055 (5)	0.0126 (6)	-0.0021 (5)
O5	0.0301 (6)	0.0322 (6)	0.0183 (5)	0.0052 (5)	0.0128 (5)	0.0037 (4)
C1	0.0216 (7)	0.0216 (7)	0.0212 (7)	0.0012 (5)	0.0111 (6)	0.0025 (5)
C2	0.0259 (8)	0.0227 (7)	0.0216 (7)	0.0023 (6)	0.0120 (6)	0.0004 (6)

C3	0.0308 (8)	0.0234 (8)	0.0265 (8)	0.0043 (6)	0.0121 (7)	0.0026 (6)
C4	0.0296 (8)	0.0236 (8)	0.0226 (8)	0.0015 (6)	0.0101 (7)	0.0041 (6)
C5	0.0206 (7)	0.0240 (7)	0.0222 (7)	-0.0005 (5)	0.0113 (6)	0.0029 (6)
C6	0.0183 (7)	0.0249 (7)	0.0205 (7)	0.0005 (5)	0.0064 (6)	-0.0001 (6)
C7	0.0247 (8)	0.0281 (8)	0.0239 (8)	-0.0007 (6)	0.0098 (6)	0.0016 (6)
C8	0.0272 (8)	0.0245 (8)	0.0341 (9)	0.0013 (6)	0.0081 (7)	-0.0010 (6)
C9	0.0251 (8)	0.0358 (9)	0.0303 (9)	0.0045 (7)	0.0080 (7)	-0.0089 (7)
C10	0.0282 (8)	0.0429 (10)	0.0234 (8)	0.0040 (7)	0.0121 (7)	-0.0008 (7)
C11	0.0248 (8)	0.0298 (8)	0.0243 (8)	0.0009 (6)	0.0110 (6)	0.0037 (6)
C12	0.0241 (8)	0.0231 (7)	0.0206 (7)	-0.0036 (6)	0.0103 (6)	-0.0033 (5)
C13	0.0246 (8)	0.0282 (8)	0.0231 (8)	0.0024 (6)	0.0119 (6)	-0.0032 (6)
C14	0.0294 (8)	0.0346 (8)	0.0198 (8)	0.0012 (7)	0.0109 (7)	-0.0028 (6)
C15	0.0268 (8)	0.0314 (8)	0.0238 (8)	0.0021 (6)	0.0081 (7)	0.0004 (6)
C16	0.0246 (8)	0.0281 (8)	0.0307 (8)	0.0019 (6)	0.0127 (7)	-0.0043 (6)
C17	0.0262 (8)	0.0264 (8)	0.0256 (8)	-0.0039 (6)	0.0143 (7)	-0.0060 (6)
C18	0.0260 (7)	0.0196 (7)	0.0185 (7)	-0.0023 (6)	0.0121 (6)	-0.0009 (5)
C19	0.0232 (7)	0.0220 (7)	0.0216 (7)	0.0020 (6)	0.0102 (6)	0.0022 (5)
C20	0.0249 (8)	0.0214 (7)	0.0229 (7)	0.0003 (6)	0.0130 (6)	-0.0016 (5)
C21	0.0270 (8)	0.0217 (7)	0.0169 (7)	-0.0020 (6)	0.0112 (6)	-0.0011 (5)
C22	0.0256 (8)	0.0244 (7)	0.0221 (7)	0.0032 (6)	0.0107 (6)	0.0028 (6)
C23	0.0250 (8)	0.0224 (7)	0.0251 (8)	0.0009 (6)	0.0140 (6)	0.0002 (6)
C24	0.0384 (10)	0.0495 (11)	0.0233 (8)	0.0142 (8)	0.0157 (8)	0.0113 (7)

Geometric parameters (Å, º) for (AA1199P)

S1—O4	1.4387 (12)	C9—H9	0.9500
S1—O3	1.4417 (12)	C10—C11	1.388 (2)
S1—C12	1.7607 (16)	C10—H10	0.9500
S1—C2	1.7995 (16)	C11—H11	0.9500
O1—C1	1.4271 (18)	C12—C17	1.391 (2)
O1—C5	1.4720 (18)	C12—C13	1.392 (2)
O2—C1	1.3784 (17)	C13—C14	1.386 (2)
O2—C18	1.4022 (17)	C13—H13	0.9500
O5—C21	1.3712 (18)	C14—C15	1.387 (2)
O5—C24	1.428 (2)	C14—H14	0.9500
C1—C5	1.469 (2)	C15—C16	1.388 (2)
C1—C2	1.519 (2)	C15—H15	0.9500
C2—C3	1.549 (2)	C16—C17	1.387 (2)
C2—H2	1.0000	C16—H16	0.9500

C3—C4	1.543 (2)	C17—H17	0.9500
C3—H3A	0.9900	C18—C23	1.381 (2)
C3—H3B	0.9900	C18—C19	1.394 (2)
C4—C5	1.516 (2)	C19—C20	1.388 (2)
C4—H4A	0.9900	C19—H19	0.9500
C4—H4B	0.9900	C20—C21	1.391 (2)
C5—C6	1.487 (2)	C20—H20	0.9500
C6—C7	1.394 (2)	C21—C22	1.391 (2)
C6—C11	1.398 (2)	C22—C23	1.393 (2)
C7—C8	1.387 (2)	C22—H22	0.9500
C7—H7	0.9500	C23—H23	0.9500
C8—C9	1.385 (3)	C24—H24A	0.9800
C8—H8	0.9500	C24—H24B	0.9800
C9—C10	1.383 (3)	C24—H24C	0.9800
O4—S1—O3	118.55 (7)	C8—C9—H9	120.1
O4—S1—C12	107.85 (7)	C9—C10—C11	119.95 (16)
O3—S1—C12	108.45 (7)	C9—C10—H10	120.0
O4—S1—C2	106.47 (8)	C11—C10—H10	120.0
O3—S1—C2	107.23 (7)	C10—C11—C6	120.66 (15)
C12—S1—C2	107.86 (7)	C10—C11—H11	119.7
C1—O1—C5	60.87 (9)	C6—C11—H11	119.7
C1—O2—C18	116.17 (11)	C17—C12—C13	121.43 (15)
C21—O5—C24	116.58 (12)	C17—C12—S1	117.99 (12)
O2—C1—O1	117.44 (12)	C13—C12—S1	120.49 (12)
O2—C1—C5	122.34 (13)	C14—C13—C12	118.72 (15)
O1—C1—C5	61.07 (9)	C14—C13—H13	120.6
O2—C1—C2	119.88 (12)	C12—C13—H13	120.6
O1—C1—C2	111.81 (12)	C13—C14—C15	120.28 (15)
C5—C1—C2	110.38 (12)	C13—C14—H14	119.9
C1—C2—C3	102.85 (12)	C15—C14—H14	119.9
C1—C2—S1	112.90 (11)	C14—C15—C16	120.56 (15)
C3—C2—S1	107.63 (11)	C14—C15—H15	119.7
C1—C2—H2	111.0	C16—C15—H15	119.7
C3—C2—H2	111.0	C17—C16—C15	119.85 (15)
S1—C2—H2	111.0	C17—C16—H16	120.1
C4—C3—C2	106.04 (12)	C15—C16—H16	120.1
C4—C3—H3A	110.5	C16—C17—C12	119.12 (15)
C2—C3—H3A	110.5	C16—C17—H17	120.4

C4—C3—H3B	110.5	C12—C17—H17	120.4
C2—C3—H3B	110.5	C23—C18—C19	120.32 (14)
H3A—C3—H3B	108.7	C23—C18—O2	116.18 (13)
C5—C4—C3	104.63 (12)	C19—C18—O2	123.49 (13)
C5—C4—H4A	110.8	C20—C19—C18	119.34 (14)
C3—C4—H4A	110.8	C20—C19—H19	120.3
C5—C4—H4B	110.8	C18—C19—H19	120.3
C3—C4—H4B	110.8	C19—C20—C21	120.48 (14)
H4A—C4—H4B	108.9	C19—C20—H20	119.8
C1—C5—O1	58.05 (9)	C21—C20—H20	119.8
C1—C5—C6	124.34 (13)	O5—C21—C22	124.86 (14)
O1—C5—C6	115.56 (12)	O5—C21—C20	115.26 (13)
C1—C5—C4	108.10 (13)	C22—C21—C20	119.87 (14)
O1—C5—C4	110.37 (12)	C21—C22—C23	119.61 (14)
C6—C5—C4	122.76 (13)	C21—C22—H22	120.2
C7—C6—C11	118.76 (15)	C23—C22—H22	120.2
C7—C6—C5	120.55 (14)	C18—C23—C22	120.31 (14)
C11—C6—C5	120.69 (14)	C18—C23—H23	119.8
C8—C7—C6	120.28 (15)	C22—C23—H23	119.8
C8—C7—H7	119.9	O5—C24—H24A	109.5
C6—C7—H7	119.9	O5—C24—H24B	109.5
C9—C8—C7	120.43 (16)	H24A—C24—H24B	109.5
C9—C8—H8	119.8	O5—C24—H24C	109.5
C7—C8—H8	119.8	H24A—C24—H24C	109.5
C10—C9—C8	119.89 (16)	H24B—C24—H24C	109.5
C10—C9—H9	120.1		
C18—O2—C1—O1	-72.02 (16)	C4—C5—C6—C11	13.4 (2)
C18—O2—C1—C5	-143.58 (14)	C11—C6—C7—C8	0.4 (2)
C18—O2—C1—C2	69.36 (17)	C5—C6—C7—C8	-179.56 (14)
C5—O1—C1—O2	-113.69 (15)	C6—C7—C8—C9	1.0 (2)
C5—O1—C1—C2	101.97 (13)	C7—C8—C9—C10	-1.7 (3)
O2—C1—C2—C3	168.45 (13)	C8—C9—C10—C11	1.0 (3)
O1—C1—C2—C3	-48.18 (15)	C9—C10—C11—C6	0.5 (2)
C5—C1—C2—C3	17.80 (16)	C7—C6—C11—C10	-1.2 (2)
O2—C1—C2—S1	52.75 (16)	C5—C6—C11—C10	178.78 (14)

O1—C1—C2—S1	-163.88 (10)	O4—S1—C12—C17	-122.76 (13)
C5—C1—C2—S1	-97.90 (13)	O3—S1—C12—C17	6.79 (14)
O4—S1—C2—C1	171.37 (11)	C2—S1—C12—C17	122.61 (12)
O3—S1—C2—C1	43.53 (12)	O4—S1—C12—C13	53.97 (15)
C12—S1—C2—C1	-73.09 (12)	O3—S1—C12—C13	-176.47 (12)
O4—S1—C2—C3	58.56 (12)	C2—S1—C12—C13	-60.65 (14)
O3—S1—C2—C3	-69.28 (11)	C17—C12—C13—C14	-2.0 (2)
C12—S1—C2—C3	174.10 (10)	S1—C12—C13—C14	-178.61 (12)
C1—C2—C3—C4	-27.35 (16)	C12—C13—C14—C15	1.7 (2)
S1—C2—C3—C4	92.08 (13)	C13—C14—C15—C16	0.0 (3)
C2—C3—C4—C5	27.17 (17)	C14—C15—C16—C17	-1.5 (3)
O2—C1—C5—O1	105.86 (15)	C15—C16—C17—C12	1.2 (2)
C2—C1—C5—O1	-104.33 (13)	C13—C12—C17—C16	0.5 (2)
O2—C1—C5—C6	4.9 (2)	S1—C12—C17—C16	177.23 (12)
O1—C1—C5—C6	-100.94 (15)	C1—O2—C18—C23	-159.46 (13)
C2—C1—C5—C6	154.73 (14)	C1—O2—C18—C19	21.5 (2)
O2—C1—C5—C4	-150.98 (14)	C23—C18—C19—C20	2.1 (2)
O1—C1—C5—C4	103.16 (13)	O2—C18—C19—C20	-178.91 (13)
C2—C1—C5—C4	-1.17 (17)	C18—C19—C20—C21	0.2 (2)
C1—O1—C5—C6	116.02 (15)	C24—O5—C21—C22	3.7 (2)
C1—O1—C5—C4	-99.15 (14)	C24—O5—C21—C20	-177.52 (14)
C3—C4—C5—C1	-16.16 (17)	C19—C20—C21—O5	179.05 (13)
C3—C4—C5—O1	45.65 (16)	C19—C20—C21—C22	-2.1 (2)
C3—C4—C5—C6	-172.52 (14)	O5—C21—C22—C23	-179.54 (14)
C1—C5—C6—C7	40.9 (2)	C20—C21—C22—	1.7 (2)

		C23	
O1—C5—C6—C7	-26.6 (2)	C19—C18—C23—C22	-2.5 (2)
C4—C5—C6—C7	-166.62 (15)	O2—C18—C23—C22	178.46 (13)
C1—C5—C6—C11	-139.12 (16)	C21—C22—C23—C18	0.6 (2)
O1—C5—C6—C11	153.44 (13)		

Compound 16e

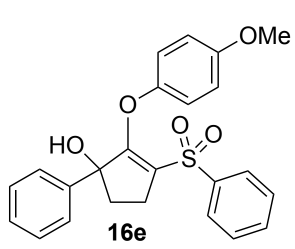

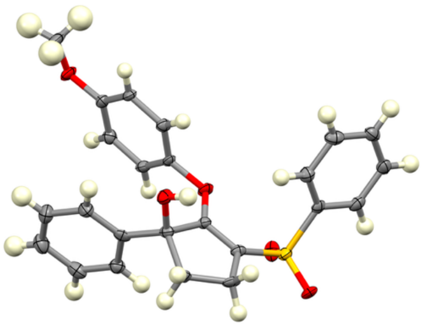

Sample Name: CCDC 2517782 / AA1188P2
Crystal Growth: Slow evaporation using a mixture of hexanes and dichloromethane

Crystal data

$C_{24}H_{22}O_5S$	$F(000) = 888$
$M_r = 422.47$	$D_x = 1.386$ Mg m^{-3}
Monoclinic, $P2_1/c$	Cu *Ka* radiation, l = 1.54184 Å
a = 16.1204 (9) Å	Cell parameters from 2062 reflections
b = 5.8235 (4) Å	q = 2.9–67.2°
c = 22.8744 (13) Å	m = 1.71 mm^{-1}
b = 109.409 (4)°	*T* = 100 K
V = 2025.3 (2) Å^3	Needle, colourless
Z = 4	0.18 × 0.03 × 0.01 mm

Data collection

Bruker D8 Venture DUO with Photon III C14 diffractometer	2426 reflections with *I* > 2s(*I*)
Radiation source: ImS 3.0 microfocus	$R_{int} = 0.129$
f and w scans	$q_{max} = 68.4°$, $q_{min} = 2.9°$
Absorption correction: multi-scan *SADABS* (Krause *et al.*, 2015)	*h* = -19 - 19
$T_{min} = 0.769$, $T_{max} = 0.983$	*k* = -4 - 6
22035 measured reflections	*l* = -27 - 27
3591 independent reflections	

Refinement

Refinement on F^2	Hydrogen site location: inferred from neighbouring sites
Least-squares matrix: full	H-atom parameters constrained

$R[F^2 > 2s(F^2)] = 0.056$	$w = 1/[s^2(F_o^2) + (0.0626P)^2 + 0.9243P]$ where $P = (F_o^2 + 2F_c^2)/3$
$wR(F^2) = 0.145$	$(D/s)_{max} = 0.001$
$S = 1.01$	$Dr_{max} = 0.48$ e Å^{-3}
3591 reflections	$Dr_{min} = -0.45$ e Å^{-3}
274 parameters	Extinction correction: *SHELXL2019*/1 (Sheldrick 2019), $Fc^* = kFc[1+0.001xFc^2l^3/\sin(2q)]^{-1/4}$
0 restraints	Extinction coefficient: 0.0023 (3)

Fractional atomic coordinates and isotropic or equivalent isotropic displacement parameters (Å^2) for (AA1188P2)

	x	*y*	*z*	$U_{iso}*/U_{eq}$
S1	0.07955 (5)	0.39956 (15)	0.64779 (4)	0.0210 (3)
O1	0.26764 (13)	0.5329 (4)	0.65403 (10)	0.0226 (6)
O2	0.24674 (14)	1.0169 (4)	0.59393 (10)	0.0222 (5)
H20H	0.211975	1.077416	0.609821	0.033*
O3	0.12525 (16)	0.1856 (4)	0.64909 (11)	0.0293 (6)
O4	-0.01466 (14)	0.3961 (5)	0.62183 (10)	0.0320 (6)
O5	0.61904 (14)	0.6143 (4)	0.67778 (10)	0.0268 (6)
C1	0.20047 (19)	0.6356 (6)	0.61008 (13)	0.0176 (7)
C2	0.11639 (19)	0.6004 (6)	0.60569 (13)	0.0181 (7)
C3	0.0533 (2)	0.7516 (6)	0.55796 (15)	0.0238 (8)
H3A	0.030713	0.877037	0.577695	0.029*
H3B	0.003161	0.662077	0.530652	0.029*
C4	0.1110 (2)	0.8452 (6)	0.52203 (14)	0.0250 (8)
H4A	0.098618	1.010275	0.512844	0.030*
H4B	0.098582	0.761841	0.482316	0.030*
C5	0.2091 (2)	0.8110 (6)	0.56302 (14)	0.0197 (7)
C6	0.2658 (2)	0.7245 (6)	0.52626 (13)	0.0208 (8)
C7	0.2445 (2)	0.5149 (6)	0.49513 (14)	0.0248 (8)
H7	0.194625	0.431506	0.496687	0.030*
C8	0.2954 (2)	0.4276 (7)	0.46206 (15)	0.0302 (9)
H8	0.279988	0.285468	0.440824	0.036*
C9	0.3684 (3)	0.5465 (7)	0.45984 (16)	0.0347 (10)
H9	0.403991	0.485527	0.437795	0.042*
C10	0.3890 (2)	0.7547 (7)	0.48997 (16)	0.0339 (9)
H10	0.438532	0.838369	0.487908	0.041*
C11	0.3386 (2)	0.8431 (7)	0.52312 (15)	0.0264 (8)

H11	0.354001	0.986099	0.543879	0.032*
C12	0.10923 (19)	0.4930 (6)	0.72552 (14)	0.0182 (7)
C13	0.1540 (2)	0.6955 (6)	0.74597 (15)	0.0233 (8)
H13	0.167922	0.795593	0.717781	0.028*
C14	0.1784 (2)	0.7509 (6)	0.80902 (15)	0.0278 (8)
H14	0.209630	0.888804	0.824122	0.033*
C15	0.1568 (2)	0.6044 (7)	0.84898 (15)	0.0259 (8)
H15	0.173851	0.642097	0.891752	0.031*
C16	0.1110 (2)	0.4037 (7)	0.82806 (15)	0.0260 (8)
H16	0.096240	0.305400	0.856273	0.031*
C17	0.0865 (2)	0.3460 (6)	0.76606 (15)	0.0246 (8)
H17	0.054809	0.208525	0.751214	0.030*
C18	0.35662 (19)	0.5688 (6)	0.65804 (13)	0.0182 (7)
C19	0.4007 (2)	0.7592 (6)	0.68700 (14)	0.0196 (7)
H19	0.371095	0.873874	0.702098	0.024*
C20	0.4900 (2)	0.7840 (6)	0.69427 (14)	0.0203 (7)
H20	0.521535	0.916546	0.713809	0.024*
C21	0.5319 (2)	0.6116 (6)	0.67243 (13)	0.0198 (7)
C22	0.4858 (2)	0.4172 (6)	0.64483 (14)	0.0222 (7)
H22	0.515115	0.298462	0.630913	0.027*
C23	0.3974 (2)	0.3949 (6)	0.63740 (14)	0.0210 (7)
H23	0.365575	0.262062	0.618403	0.025*
C24	0.6666 (2)	0.8194 (8)	0.6991 (2)	0.0423 (11)
H24A	0.724992	0.807318	0.695002	0.063*
H24B	0.672733	0.843637	0.742785	0.063*
H24C	0.634862	0.949233	0.674432	0.063*

Atomic displacement parameters (Å^2) for (AA1188P2)

	U^{11}	U^{22}	U^{33}	U^{12}	U^{13}	U^{23}
S1	0.0160 (4)	0.0239 (5)	0.0234 (4)	-0.0051 (4)	0.0070 (3)	-0.0054 (3)
O1	0.0113 (11)	0.0303 (15)	0.0229 (12)	0.0014 (10)	0.0014 (9)	0.0054 (10)
O2	0.0190 (12)	0.0203 (14)	0.0274 (12)	0.0001 (10)	0.0081 (9)	-0.0033 (10)
O3	0.0385 (15)	0.0198 (14)	0.0368 (14)	-0.0030 (11)	0.0221 (11)	-0.0024 (10)
O4	0.0131 (12)	0.0539 (19)	0.0264 (12)	-0.0140 (12)	0.0033 (9)	-0.0045 (12)

O5	0.0111 (11)	0.0351 (16)	0.0339 (13)	-0.0039 (11)	0.0072 (9)	-0.0091 (11)
C1	0.0133 (16)	0.021 (2)	0.0169 (15)	-0.0015 (14)	0.0022 (12)	-0.0017 (13)
C2	0.0135 (16)	0.0189 (19)	0.0196 (16)	0.0018 (14)	0.0024 (12)	-0.0005 (13)
C3	0.0106 (16)	0.029 (2)	0.0252 (17)	0.0021 (15)	-0.0026 (13)	-0.0071 (15)
C4	0.0203 (18)	0.030 (2)	0.0185 (16)	0.0014 (15)	-0.0024 (13)	0.0042 (14)
C5	0.0189 (17)	0.0199 (19)	0.0178 (16)	-0.0032 (14)	0.0027 (13)	0.0010 (13)
C6	0.0220 (17)	0.023 (2)	0.0125 (15)	0.0038 (15)	-0.0012 (13)	0.0034 (13)
C7	0.0236 (19)	0.027 (2)	0.0216 (17)	-0.0020 (16)	0.0047 (14)	0.0011 (14)
C8	0.039 (2)	0.030 (2)	0.0197 (17)	0.0020 (18)	0.0070 (15)	-0.0026 (15)
C9	0.038 (2)	0.041 (3)	0.030 (2)	0.0026 (19)	0.0176 (17)	-0.0057 (17)
C10	0.029 (2)	0.044 (3)	0.034 (2)	-0.0059 (18)	0.0176 (16)	-0.0025 (17)
C11	0.0276 (19)	0.030 (2)	0.0231 (17)	-0.0062 (16)	0.0102 (15)	-0.0035 (14)
C12	0.0101 (15)	0.0215 (19)	0.0227 (16)	0.0038 (14)	0.0051 (12)	-0.0003 (13)
C13	0.0229 (18)	0.023 (2)	0.0239 (17)	0.0006 (15)	0.0077 (14)	-0.0001 (14)
C14	0.026 (2)	0.025 (2)	0.0284 (18)	0.0016 (16)	0.0038 (15)	-0.0050 (15)
C15	0.0201 (18)	0.037 (2)	0.0193 (16)	0.0075 (17)	0.0046 (13)	-0.0033 (15)
C16	0.0176 (17)	0.037 (2)	0.0228 (17)	0.0027 (17)	0.0067 (13)	0.0048 (16)
C17	0.0140 (17)	0.027 (2)	0.0327 (19)	-0.0002 (15)	0.0072 (14)	-0.0019 (15)
C18	0.0088 (15)	0.027 (2)	0.0167 (15)	-0.0013 (14)	0.0015 (12)	0.0046 (13)
C19	0.0151 (16)	0.022 (2)	0.0201 (16)	0.0013 (14)	0.0034 (13)	0.0005 (13)
C20	0.0138 (16)	0.025 (2)	0.0196 (16)	-0.0065 (14)	0.0022 (13)	0.0001 (14)

C21	0.0143 (16)	0.027 (2)	0.0183 (16)	0.0016 (15)	0.0051 (12)	-0.0004 (14)
C22	0.0206 (17)	0.025 (2)	0.0216 (16)	0.0024 (15)	0.0077 (13)	-0.0018 (14)
C23	0.0151 (16)	0.024 (2)	0.0198 (16)	-0.0037 (15)	0.0006 (12)	-0.0008 (14)
C24	0.0157 (19)	0.053 (3)	0.060 (3)	-0.0149 (19)	0.0154 (18)	-0.027 (2)

Geometric parameters (Å, º) for (AA1188P2)

S1—O4	1.435 (2)	C10—C11	1.382 (5)
S1—O3	1.443 (3)	C10—H10	0.9500
S1—C2	1.740 (3)	C11—H11	0.9500
S1—C12	1.767 (3)	C12—C13	1.381 (5)
O1—C1	1.348 (4)	C12—C17	1.397 (5)
O1—C18	1.422 (4)	C13—C14	1.400 (5)
O2—C5	1.422 (4)	C13—H13	0.9500
O2—H20H	0.8400	C14—C15	1.376 (5)
O5—C21	1.369 (4)	C14—H14	0.9500
O5—C24	1.416 (5)	C15—C16	1.381 (5)
C1—C2	1.341 (4)	C15—H15	0.9500
C1—C5	1.523 (4)	C16—C17	1.381 (5)
C2—C3	1.504 (4)	C16—H16	0.9500
C3—C4	1.531 (5)	C17—H17	0.9500
C3—H3A	0.9900	C18—C19	1.364 (5)
C3—H3B	0.9900	C18—C23	1.374 (5)
C4—C5	1.560 (4)	C19—C20	1.401 (4)
C4—H4A	0.9900	C19—H19	0.9500
C4—H4B	0.9900	C20—C21	1.392 (5)
C5—C6	1.518 (5)	C20—H20	0.9500
C6—C11	1.384 (5)	C21—C22	1.386 (5)
C6—C7	1.397 (5)	C22—C23	1.384 (4)
C7—C8	1.384 (5)	C22—H22	0.9500
C7—H7	0.9500	C23—H23	0.9500
C8—C9	1.381 (5)	C24—H24A	0.9800
C8—H8	0.9500	C24—H24B	0.9800
C9—C10	1.380 (5)	C24—H24C	0.9800
C9—H9	0.9500		

O4—S1—O3	117.34 (16)	C11—C10—H10	119.6
O4—S1—C2	106.81 (15)	C10—C11—C6	120.4 (3)
O3—S1—C2	109.04 (15)	C10—C11—H11	119.8
O4—S1—C12	108.44 (14)	C6—C11—H11	119.8
O3—S1—C12	106.23 (15)	C13—C12—C17	121.4 (3)
C2—S1—C12	108.77 (16)	C13—C12—S1	122.7 (2)
C1—O1—C18	121.7 (2)	C17—C12—S1	115.9 (3)
C5—O2—H20H	109.5	C12—C13—C14	118.8 (3)
C21—O5—C24	117.5 (3)	C12—C13—H13	120.6
C2—C1—O1	122.0 (3)	C14—C13—H13	120.6
C2—C1—C5	112.1 (3)	C15—C14—C13	119.6 (3)
O1—C1—C5	125.8 (3)	C15—C14—H14	120.2
C1—C2—C3	112.8 (3)	C13—C14—H14	120.2
C1—C2—S1	125.7 (2)	C14—C15—C16	121.2 (3)
C3—C2—S1	121.5 (2)	C14—C15—H15	119.4
C2—C3—C4	102.3 (3)	C16—C15—H15	119.4
C2—C3—H3A	111.3	C15—C16—C17	119.9 (3)
C4—C3—H3A	111.3	C15—C16—H16	120.0
C2—C3—H3B	111.3	C17—C16—H16	120.0
C4—C3—H3B	111.3	C16—C17—C12	119.0 (3)
H3A—C3—H3B	109.2	C16—C17—H17	120.5
C3—C4—C5	107.9 (2)	C12—C17—H17	120.5
C3—C4—H4A	110.1	C19—C18—C23	122.3 (3)
C5—C4—H4A	110.1	C19—C18—O1	120.2 (3)
C3—C4—H4B	110.1	C23—C18—O1	117.2 (3)
C5—C4—H4B	110.1	C18—C19—C20	119.4 (3)
H4A—C4—H4B	108.4	C18—C19—H19	120.3
O2—C5—C6	108.6 (3)	C20—C19—H19	120.3
O2—C5—C1	109.8 (2)	C21—C20—C19	119.0 (3)
C6—C5—C1	112.5 (3)	C21—C20—H20	120.5
O2—C5—C4	111.8 (3)	C19—C20—H20	120.5
C6—C5—C4	112.7 (3)	O5—C21—C22	115.4 (3)
C1—C5—C4	101.4 (2)	O5—C21—C20	124.4 (3)
C11—C6—C7	118.6 (3)	C22—C21—C20	120.1 (3)
C11—C6—C5	122.7 (3)	C23—C22—C21	120.5 (3)
C7—C6—C5	118.7 (3)	C23—C22—H22	119.8
C8—C7—C6	120.6 (3)	C21—C22—H22	119.8
C8—C7—H7	119.7	C18—C23—C22	118.6 (3)
C6—C7—H7	119.7	C18—C23—H23	120.7

C9—C8—C7	120.2 (3)	C22—C23—H23	120.7
C9—C8—H8	119.9	O5—C24—H24A	109.5
C7—C8—H8	119.9	O5—C24—H24B	109.5
C10—C9—C8	119.3 (3)	H24A—C24—H24B	109.5
C10—C9—H9	120.4	O5—C24—H24C	109.5
C8—C9—H9	120.4	H24A—C24—H24C	109.5
C9—C10—C11	120.9 (4)	H24B—C24—H24C	109.5
C9—C10—H10	119.6		
C18—O1—C1—C2	179.3 (3)	C7—C8—C9—C10	-1.2 (5)
C18—O1—C1—C5	-4.5 (5)	C8—C9—C10—C11	1.2 (6)
O1—C1—C2—C3	173.7 (3)	C9—C10—C11—C6	-0.5 (6)
C5—C1—C2—C3	-3.0 (4)	C7—C6—C11—C10	-0.2 (5)
O1—C1—C2—S1	-7.9 (5)	C5—C6—C11—C10	178.7 (3)
C5—C1—C2—S1	175.4 (2)	O4—S1—C12—C13	-115.4 (3)
O4—S1—C2—C1	-171.3 (3)	O3—S1—C12—C13	117.6 (3)
O3—S1—C2—C1	-43.6 (3)	C2—S1—C12—C13	0.4 (3)
C12—S1—C2—C1	71.9 (3)	O4—S1—C12—C17	66.1 (3)
O4—S1—C2—C3	7.0 (3)	O3—S1—C12—C17	-60.9 (3)
O3—S1—C2—C3	134.7 (3)	C2—S1—C12—C17	-178.1 (2)
C12—S1—C2—C3	-109.8 (3)	C17—C12—C13—C14	1.3 (5)
C1—C2—C3—C4	13.5 (4)	S1—C12—C13—C14	-177.1 (2)
S1—C2—C3—C4	-165.0 (2)	C12—C13—C14—C15	-0.5 (5)
C2—C3—C4—C5	-18.4 (3)	C13—C14—C15—C16	-0.4 (5)
C2—C1—C5—O2	109.6 (3)	C14—C15—C16—C17	0.6 (5)
O1—C1—C5—O2	-67.0 (4)	C15—C16—C17—C12	0.2 (5)
C2—C1—C5—C6	-129.4 (3)	C13—C12—C17—C16	-1.2 (5)
O1—C1—C5—C6	54.1 (4)	S1—C12—C17—C16	177.3 (2)
C2—C1—C5—C4	-8.7 (4)	C1—O1—C18—C19	81.9 (4)
O1—C1—C5—C4	174.7 (3)	C1—O1—C18—C23	-104.4 (3)
C3—C4—C5—O2	-100.1 (3)	C23—C18—C19—	2.3 (5)

		C20	
C3—C4—C5—C6	137.3 (3)	O1—C18—C19—C20	175.6 (3)
C3—C4—C5—C1	16.8 (3)	C18—C19—C20—C21	-0.9 (4)
O2—C5—C6—C11	-3.0 (4)	C24—O5—C21—C22	173.0 (3)
C1—C5—C6—C11	-124.7 (3)	C24—O5—C21—C20	-9.1 (5)
C4—C5—C6—C11	121.4 (3)	C19—C20—C21—O5	-178.8 (3)
O2—C5—C6—C7	175.9 (3)	C19—C20—C21—C22	-1.0 (5)
C1—C5—C6—C7	54.2 (4)	O5—C21—C22—C23	179.5 (3)
C4—C5—C6—C7	-59.7 (4)	C20—C21—C22—C23	1.5 (5)
C11—C6—C7—C8	0.2 (5)	C19—C18—C23—C22	-1.8 (5)
C5—C6—C7—C8	-178.8 (3)	O1—C18—C23—C22	-175.3 (3)
C6—C7—C8—C9	0.5 (5)	C21—C22—C23—C18	-0.2 (5)

Hydrogen-bond geometry (Å, º) for (AA1188P2)

D—H···*A*	*D*—H	H···*A*	*D*···*A*	*D*—H···*A*
O2—H20*H*···O3^{i}	0.84	2.00	2.834 (3)	174

Symmetry code: (i) *x*, *y*+1, *z*.

Compound 16f

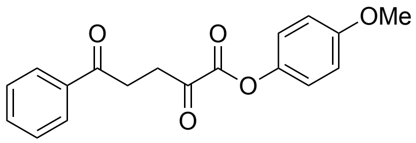

16f

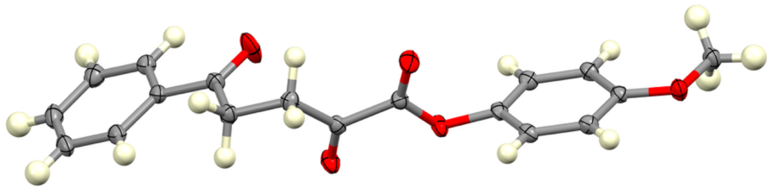

Sample Name: CCDC 2517783 / Aldas4
Crystal Growth: Slow evaporation using a mixture of hexanes and dichloromethane

Crystal data

$C_{18}H_{16}O_5$	*F*(000) = 656
M_r = 312.31	D_x = 1.379 Mg m^{-3}
Monoclinic, *P*2$_1$/*c*	Cu *K*a radiation, l = 1.54184 Å
a = 13.4825 (4) Å	Cell parameters from 9823 reflections
b = 5.3839 (2) Å	q = 6.6–79.5°
c = 20.7592 (6) Å	m = 0.84 mm^{-1}
b = 93.655 (2)°	*T* = 100 K
V = 1503.81 (8) $Å^3$	Needle, colourless
Z = 4	0.23 × 0.15 × 0.11 mm

Data collection

Bruker D8 Venture DUO with Photon III C14 diffractometer	3116 reflections with *I* > 2s(*I*)
Radiation source: ImS 3.0 microfocus	R_{int} = 0.030
f and w scans	q_{max} = 79.9°, q_{min} = 3.3°
Absorption correction: multi-scan *SADABS* (Krause *et al.*, 2015)	*h* = -17 - 16
T_{min} = 0.783, T_{max} = 0.914	*k* = -6 - 6
34200 measured reflections	*l* = -25 - 26
3232 independent reflections	

Refinement

Refinement on F^2	Hydrogen site location: inferred from neighbouring sites
Least-squares matrix: full	H-atom parameters constrained
$R[F^2 > 2s(F^2)]$ = 0.033	$w = 1/[s^2(F_o^2) + (0.0403P)^2 + 0.5499P]$ where $P = (F_o^2 + 2F_c^2)/3$
$wR(F^2)$ = 0.088	$(D/s)_{max}$ < 0.001

S = 1.05	$\Delta\rho_{max}$ = 0.33 e Å^{-3}
3232 reflections	$\Delta\rho_{min}$ = -0.18 e Å^{-3}
210 parameters	Extinction correction: *SHELXL2019*/1 (Sheldrick 2019), $Fc^{*}=kFc[1+0.001xFc^{2}l^{3}/sin(2q)]^{-1/4}$
0 restraints	Extinction coefficient: 0.0016 (3)

Fractional atomic coordinates and isotropic or equivalent isotropic displacement parameters (Å^2) for (Aldas4)

	x	*y*	*z*	U_{iso}*/U_{eq}
O1	0.64121 (5)	0.74183 (13)	0.26477 (3)	0.02138 (17)
O2	0.68428 (5)	0.41935 (15)	0.33034 (4)	0.02912 (19)
O3	0.45991 (5)	0.73262 (14)	0.30469 (4)	0.02468 (18)
O4	0.47537 (6)	0.75703 (18)	0.45809 (5)	0.0406 (2)
O5	0.99193 (5)	0.79742 (14)	0.14185 (3)	0.02246 (17)
C1	0.62417 (7)	0.56433 (18)	0.30796 (4)	0.0184 (2)
C2	0.51521 (7)	0.57371 (18)	0.32689 (4)	0.0181 (2)
C3	0.48778 (7)	0.37391 (19)	0.37261 (5)	0.0206 (2)
H3A	0.541273	0.357468	0.407295	0.025*
H3B	0.482402	0.213825	0.349186	0.025*
C4	0.39023 (7)	0.42697 (19)	0.40270 (5)	0.0213 (2)
H4A	0.337862	0.457523	0.367984	0.026*
H4B	0.370589	0.279272	0.427281	0.026*
C5	0.39718 (7)	0.6485 (2)	0.44717 (5)	0.0223 (2)
C6	0.30502 (7)	0.73491 (19)	0.47720 (4)	0.0197 (2)
C7	0.31146 (7)	0.94114 (19)	0.51804 (5)	0.0219 (2)
H7	0.373784	1.020117	0.527188	0.026*
C8	0.22723 (8)	1.0315 (2)	0.54539 (5)	0.0267 (2)
H8	0.231974	1.171676	0.573210	0.032*
C9	0.13591 (8)	0.9161 (2)	0.53192 (5)	0.0295 (2)
H9	0.078093	0.978654	0.550176	0.035*
C10	0.12923 (8)	0.7101 (2)	0.49193 (5)	0.0280 (2)
H10	0.066819	0.631110	0.483150	0.034*
C11	0.21311 (7)	0.6181 (2)	0.46457 (5)	0.0230 (2)
H11	0.208113	0.476212	0.437344	0.028*
C12	0.73546 (7)	0.74523 (18)	0.23885 (5)	0.0183 (2)
C13	0.75837 (7)	0.56568 (18)	0.19428 (5)	0.0192 (2)
H13	0.715008	0.429158	0.185360	0.023*
C14	0.84555 (7)	0.58893 (18)	0.16300 (5)	0.0190 (2)

H14	0.862243	0.467930	0.132186	0.023*
C15	0.90917 (7)	0.79019 (18)	0.17663 (4)	0.0175 (2)
C16	0.88586 (7)	0.96572 (18)	0.22239 (5)	0.0189 (2)
H16	0.929760	1.100317	0.232340	0.023*
C17	0.79748 (7)	0.94323 (18)	0.25370 (5)	0.0194 (2)
H17	0.780448	1.062837	0.284775	0.023*
C18	1.05980 (7)	0.9980 (2)	0.15487 (5)	0.0238 (2)
H18A	1.116366	0.981043	0.127797	0.036*
H18B	1.083586	0.994295	0.200465	0.036*
H18C	1.025972	1.156051	0.145273	0.036*

Atomic displacement parameters (Å^2) for (Aldas4)

	U^{11}	U^{22}	U^{33}	U^{12}	U^{13}	U^{23}
O1	0.0145 (3)	0.0210 (4)	0.0291 (4)	0.0034 (2)	0.0049 (3)	0.0055 (3)
O2	0.0222 (4)	0.0351 (4)	0.0305 (4)	0.0110 (3)	0.0059 (3)	0.0115 (3)
O3	0.0181 (3)	0.0228 (4)	0.0337 (4)	0.0048 (3)	0.0055 (3)	0.0049 (3)
O4	0.0197 (4)	0.0515 (5)	0.0516 (5)	-0.0140 (4)	0.0111 (3)	-0.0302 (4)
O5	0.0181 (3)	0.0257 (4)	0.0242 (3)	-0.0052 (3)	0.0058 (3)	-0.0052 (3)
C1	0.0175 (4)	0.0190 (4)	0.0186 (4)	0.0021 (3)	0.0017 (3)	-0.0006 (3)
C2	0.0163 (4)	0.0186 (4)	0.0195 (4)	0.0005 (3)	0.0018 (3)	-0.0031 (3)
C3	0.0215 (5)	0.0201 (5)	0.0202 (4)	0.0001 (4)	0.0024 (3)	-0.0003 (4)
C4	0.0204 (5)	0.0230 (5)	0.0206 (4)	-0.0056 (4)	0.0032 (3)	-0.0016 (4)
C5	0.0180 (5)	0.0276 (5)	0.0216 (4)	-0.0047 (4)	0.0021 (4)	-0.0033 (4)
C6	0.0166 (5)	0.0246 (5)	0.0179 (4)	-0.0019 (4)	0.0014 (3)	0.0034 (4)
C7	0.0197 (5)	0.0254 (5)	0.0207 (4)	-0.0009 (4)	0.0016 (3)	0.0023 (4)
C8	0.0278 (5)	0.0279 (5)	0.0250 (5)	0.0045 (4)	0.0057 (4)	0.0026 (4)
C9	0.0214 (5)	0.0373 (6)	0.0306 (5)	0.0068 (4)	0.0092 (4)	0.0099 (5)
C10	0.0166 (5)	0.0369 (6)	0.0306 (5)	-0.0038 (4)	0.0026 (4)	0.0112 (5)
C11	0.0195 (5)	0.0268 (5)	0.0226 (5)	-0.0044 (4)	0.0004 (4)	0.0048 (4)

C12	0.0134 (4)	0.0197 (5)	0.0219 (4)	0.0035 (3)	0.0013 (3)	0.0052 (3)
C13	0.0160 (4)	0.0172 (4)	0.0242 (5)	-0.0004 (3)	-0.0015 (3)	0.0017 (4)
C14	0.0186 (4)	0.0179 (4)	0.0204 (4)	0.0009 (4)	0.0001 (3)	-0.0017 (3)
C15	0.0149 (4)	0.0192 (4)	0.0184 (4)	0.0008 (3)	0.0011 (3)	0.0014 (3)
C16	0.0180 (4)	0.0165 (4)	0.0222 (4)	-0.0012 (3)	0.0000 (3)	-0.0001 (3)
C17	0.0199 (5)	0.0172 (4)	0.0213 (4)	0.0034 (4)	0.0018 (3)	0.0002 (3)
C18	0.0194 (5)	0.0274 (5)	0.0251 (5)	-0.0071 (4)	0.0039 (4)	-0.0019 (4)

Geometric parameters (Å, º) for (Aldas4)

O1—C1	1.3402 (12)	C8—C9	1.3915 (16)
O1—C12	1.4114 (11)	C8—H8	0.9500
O2—C1	1.1974 (12)	C9—C10	1.3849 (17)
O3—C2	1.2069 (12)	C9—H9	0.9500
O4—C5	1.2141 (13)	C10—C11	1.3889 (15)
O5—C15	1.3678 (11)	C10—H10	0.9500
O5—C18	1.4301 (12)	C11—H11	0.9500
C1—C2	1.5455 (13)	C12—C17	1.3775 (14)
C2—C3	1.4965 (13)	C12—C13	1.3866 (14)
C3—C4	1.5187 (13)	C13—C14	1.3842 (13)
C3—H3A	0.9900	C13—H13	0.9500
C3—H3B	0.9900	C14—C15	1.3997 (13)
C4—C5	1.5072 (14)	C14—H14	0.9500
C4—H4A	0.9900	C15—C16	1.3899 (13)
C4—H4B	0.9900	C16—C17	1.3985 (13)
C5—C6	1.4993 (13)	C16—H16	0.9500
C6—C7	1.3964 (14)	C17—H17	0.9500
C6—C11	1.3994 (14)	C18—H18A	0.9800
C7—C8	1.3896 (14)	C18—H18B	0.9800
C7—H7	0.9500	C18—H18C	0.9800
C1—O1—C12	117.46 (7)	C10—C9—H9	120.0
C15—O5—C18	117.09 (7)	C8—C9—H9	120.0
O2—C1—O1	125.71 (9)	C9—C10—C11	120.48 (10)
O2—C1—C2	123.60 (9)	C9—C10—H10	119.8
O1—C1—C2	110.67 (8)	C11—C10—H10	119.8

O3—C2—C3	125.46 (9)	C10—C11—C6	119.84 (10)
O3—C2—C1	120.02 (9)	C10—C11—H11	120.1
C3—C2—C1	114.52 (8)	C6—C11—H11	120.1
C2—C3—C4	112.40 (8)	C17—C12—C13	122.07 (9)
C2—C3—H3A	109.1	C17—C12—O1	118.24 (8)
C4—C3—H3A	109.1	C13—C12—O1	119.32 (8)
C2—C3—H3B	109.1	C14—C13—C12	118.81 (9)
C4—C3—H3B	109.1	C14—C13—H13	120.6
H3A—C3—H3B	107.9	C12—C13—H13	120.6
C5—C4—C3	112.28 (8)	C13—C14—C15	120.19 (9)
C5—C4—H4A	109.1	C13—C14—H14	119.9
C3—C4—H4A	109.1	C15—C14—H14	119.9
C5—C4—H4B	109.1	O5—C15—C16	124.68 (8)
C3—C4—H4B	109.1	O5—C15—C14	115.17 (8)
H4A—C4—H4B	107.9	C16—C15—C14	120.15 (9)
O4—C5—C6	120.37 (9)	C15—C16—C17	119.68 (9)
O4—C5—C4	120.97 (9)	C15—C16—H16	120.2
C6—C5—C4	118.65 (8)	C17—C16—H16	120.2
C7—C6—C11	119.42 (9)	C12—C17—C16	119.08 (9)
C7—C6—C5	118.57 (9)	C12—C17—H17	120.5
C11—C6—C5	122.00 (9)	C16—C17—H17	120.5
C8—C7—C6	120.37 (10)	O5—C18—H18A	109.5
C8—C7—H7	119.8	O5—C18—H18B	109.5
C6—C7—H7	119.8	H18A—C18—H18B	109.5
C7—C8—C9	119.82 (10)	O5—C18—H18C	109.5
C7—C8—H8	120.1	H18A—C18—H18C	109.5
C9—C8—H8	120.1	H18B—C18—H18C	109.5
C10—C9—C8	120.07 (10)		
C12—O1—C1—O2	6.06 (14)	C8—C9—C10—C11	-0.50 (16)
C12—O1—C1—C2	-175.18 (7)	C9—C10—C11—C6	-0.31 (15)
O2—C1—C2—O3	176.43 (10)	C7—C6—C11—C10	0.91 (15)
O1—C1—C2—O3	-2.37 (12)	C5—C6—C11—C10	-177.93 (9)
O2—C1—C2—C3	-4.07 (14)	C1—O1—C12—C17	-113.47 (10)
O1—C1—C2—C3	177.14 (8)	C1—O1—C12—C13	73.37 (11)
O3—C2—C3—C4	-14.93 (14)	C17—C12—C13—C14	-1.11 (14)
C1—C2—C3—C4	165.59 (8)	O1—C12—C13—C14	171.78 (8)

C2—C3—C4—C5	-67.10 (11)	C12—C13—C14—C15	0.25 (14)
C3—C4—C5—O4	-3.24 (15)	C18—O5—C15—C16	1.36 (13)
C3—C4—C5—C6	175.98 (8)	C18—O5—C15—C14	-179.12 (8)
O4—C5—C6—C7	-0.36 (15)	C13—C14—C15—O5	-178.56 (8)
C4—C5—C6—C7	-179.58 (9)	C13—C14—C15—C16	0.99 (14)
O4—C5—C6—C11	178.49 (10)	O5—C15—C16—C17	178.10 (8)
C4—C5—C6—C11	-0.73 (14)	C14—C15—C16—C17	-1.40 (14)
C11—C6—C7—C8	-0.70 (15)	C13—C12—C17—C16	0.70 (14)
C5—C6—C7—C8	178.17 (9)	O1—C12—C17—C16	-172.26 (8)
C6—C7—C8—C9	-0.10 (15)	C15—C16—C17—C12	0.57 (14)
C7—C8—C9—C10	0.71 (16)		

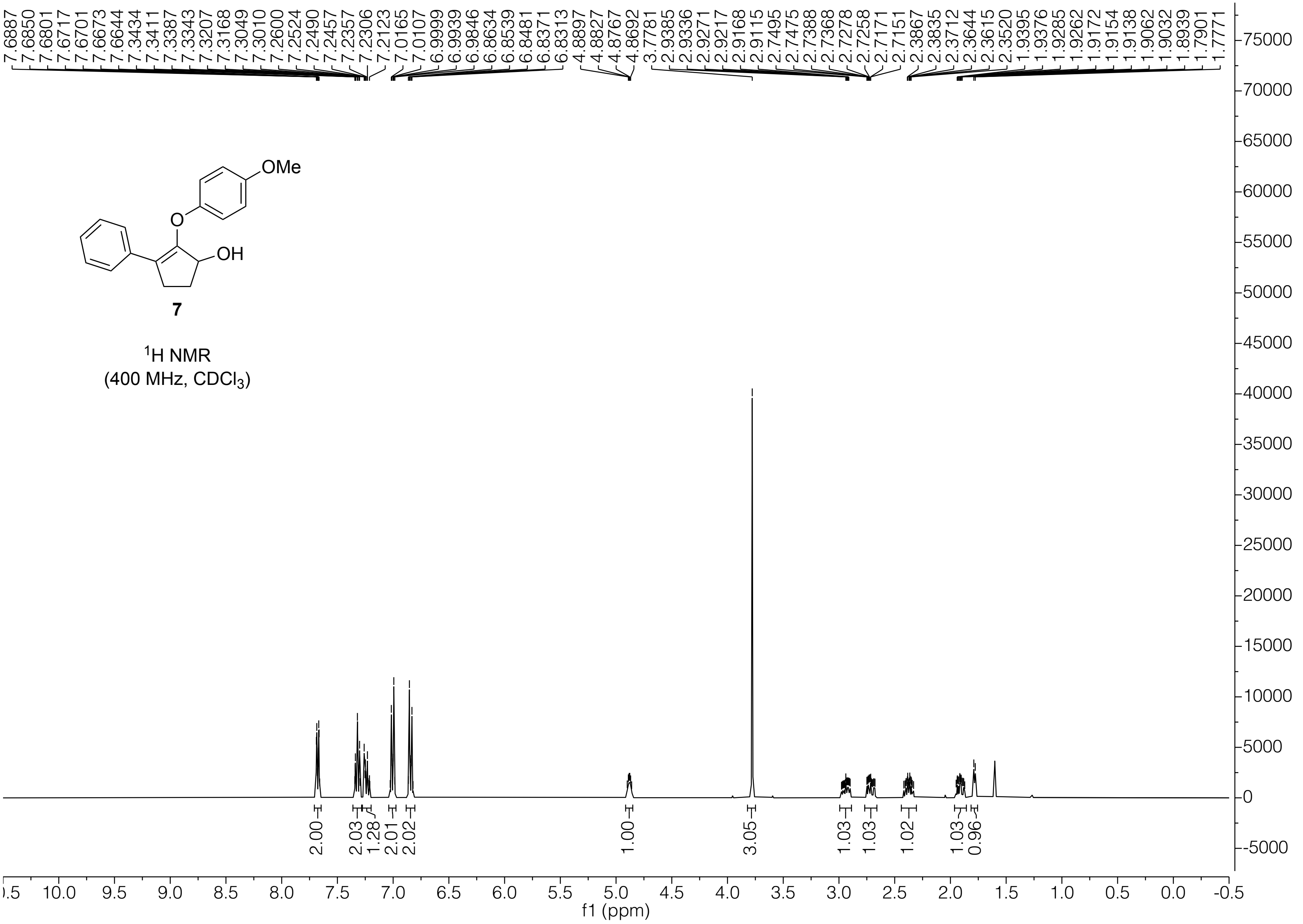
7.6887
7.6850
7.6801
7.6717
7.6701
7.6673
7.6644
7.3434
7.3411
7.3387
7.3343
7.3207
7.3168
7.3049
7.3010
7.2600
7.2524
7.2490
7.2457
7.2357
7.2306
7.2123
7.0165
7.0107
6.9999
6.9939
6.9846
6.8634
6.8539
6.8481
6.8371
6.8313
4.8897
4.8827
4.8767
4.8692
3.7781
2.9385
2.9336
2.9271
2.9217
2.9168
2.9115
2.7495
2.7475
2.7388
2.7368
2.7278
2.7258
2.7171
2.7151
2.3867
2.3835
2.3712
2.3644
2.3615
2.3520
1.9395
1.9376
1.9285
1.9262
1.9172
1.9154
1.9138
1.9062
1.9032
1.8939
1.7901
1.7771
OMe
O
OH
7
^{1}H NMR
(400 MHz, $CDCl_3$)
2.00
2.03
1.28
2.01
2.02
1.00
3.05
1.03
1.03
1.02
1.03
0.96
f1 (ppm)

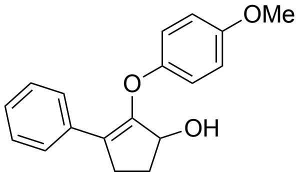

7

$^{13}C\{^1H\}$ NMR
(100 MHz, $CDCl_3$)

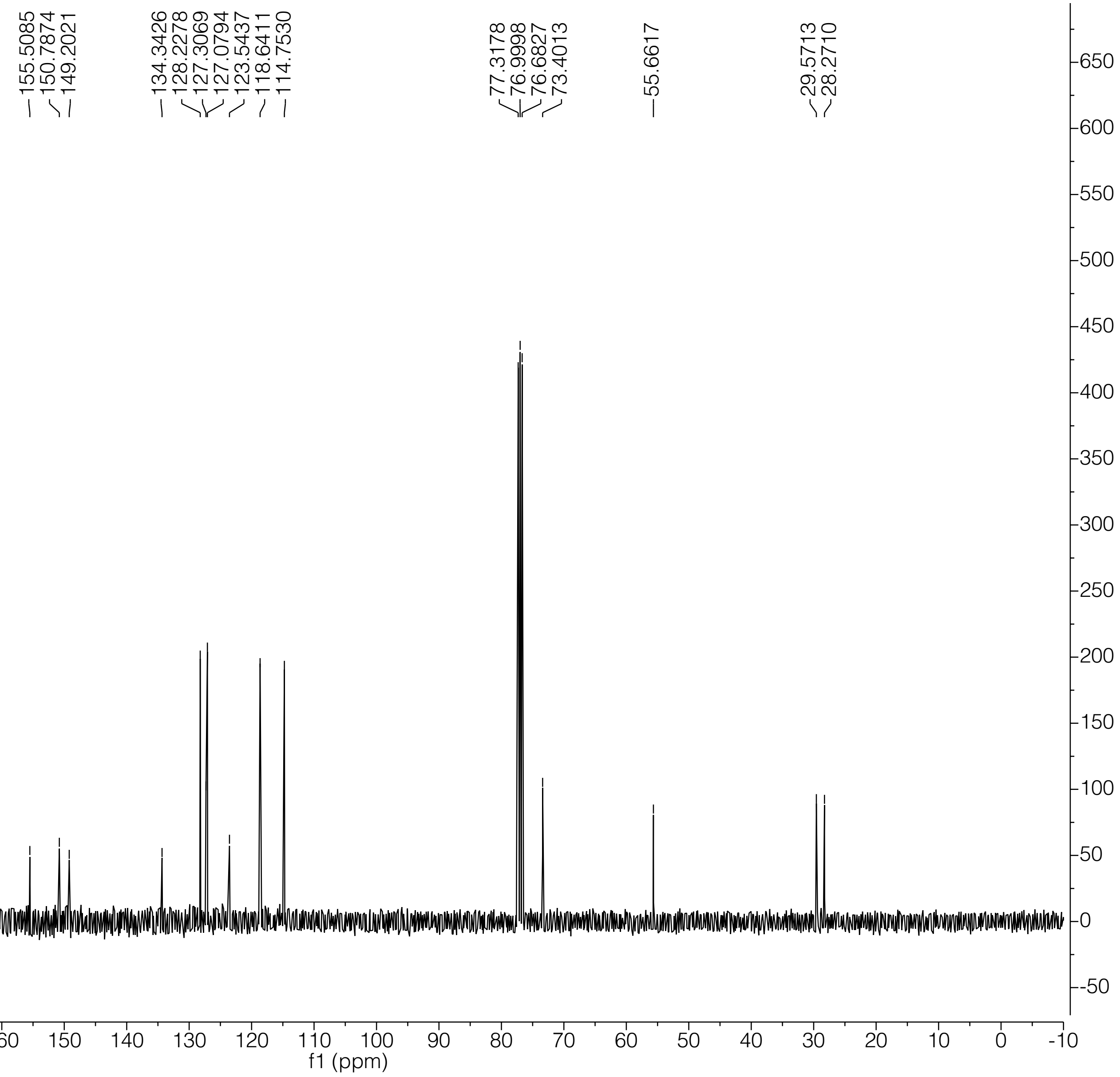

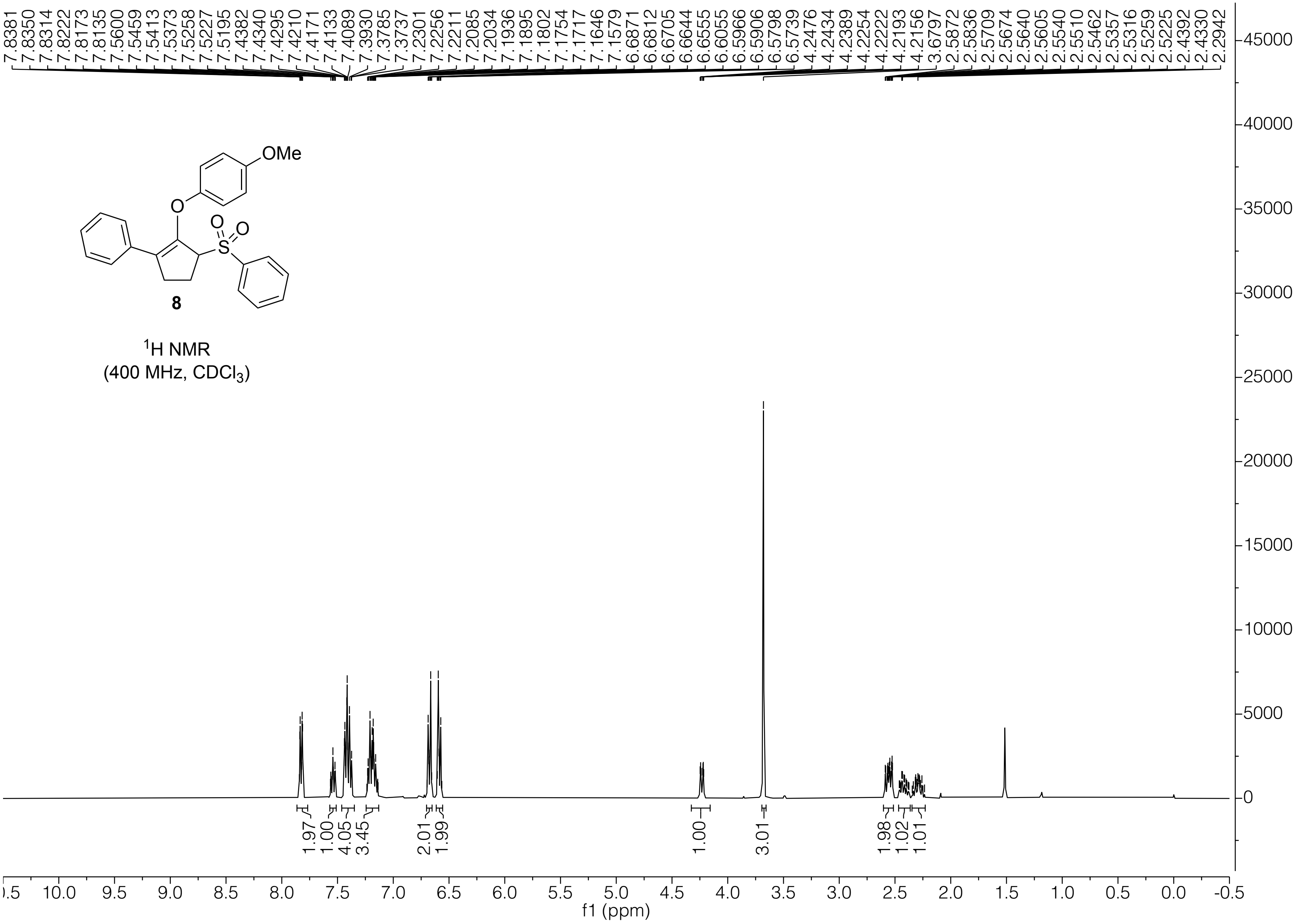
7.8381
7.8350
7.8314
7.8222
7.8173
7.8135
7.5600
7.5459
7.5413
7.5373
7.5258
7.5227
7.5195
7.4382
7.4340
7.4295
7.4210
7.4171
7.4133
7.4089
7.3930
7.3785
7.3737
7.2301
7.2256
7.2211
7.2085
7.2034
7.1936
7.1895
7.1802
7.1754
7.1717
7.1646
7.1579
6.6871
6.6812
6.6705
6.6644
6.6555
6.6055
6.5966
6.5906
6.5798
6.5739
4.2476
4.2434
4.2389
4.2254
4.2222
4.2193
4.2156
3.6797
2.5872
2.5836
2.5709
2.5674
2.5640
2.5605
2.5540
2.5510
2.5462
2.5357
2.5316
2.5259
2.5225
2.4392
2.4330
2.2942
OMe
O
O
S
O
8
^{1}H NMR
(400 MHz, CDCl$_3$)
1.97
1.00
4.05
3.45
2.01
1.99
1.00
3.01
1.98
1.02
1.01
f1 (ppm)

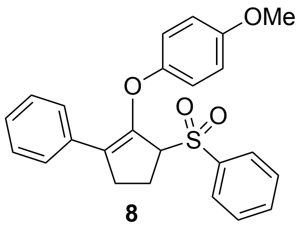

8

$^{13}C\{^{1}H\}$ NMR
(100 MHz, $CDCl_3$)

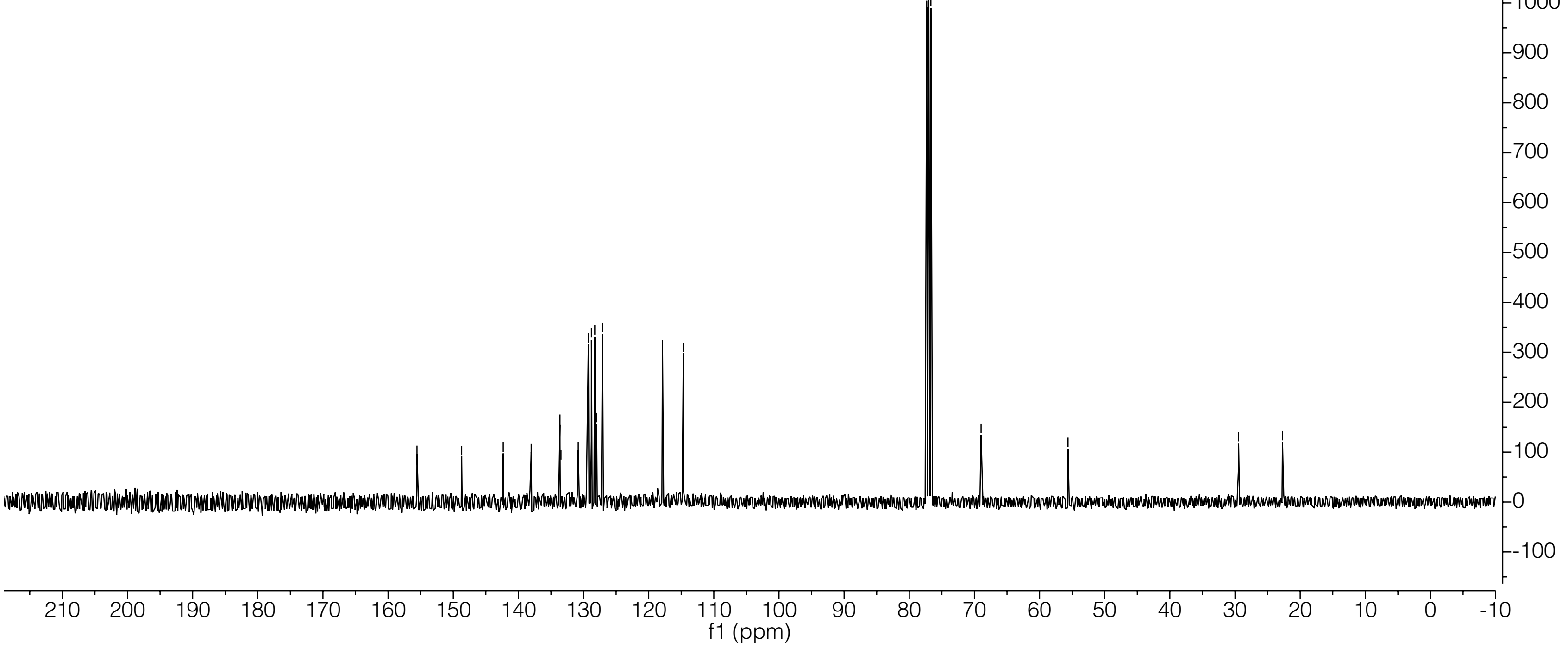

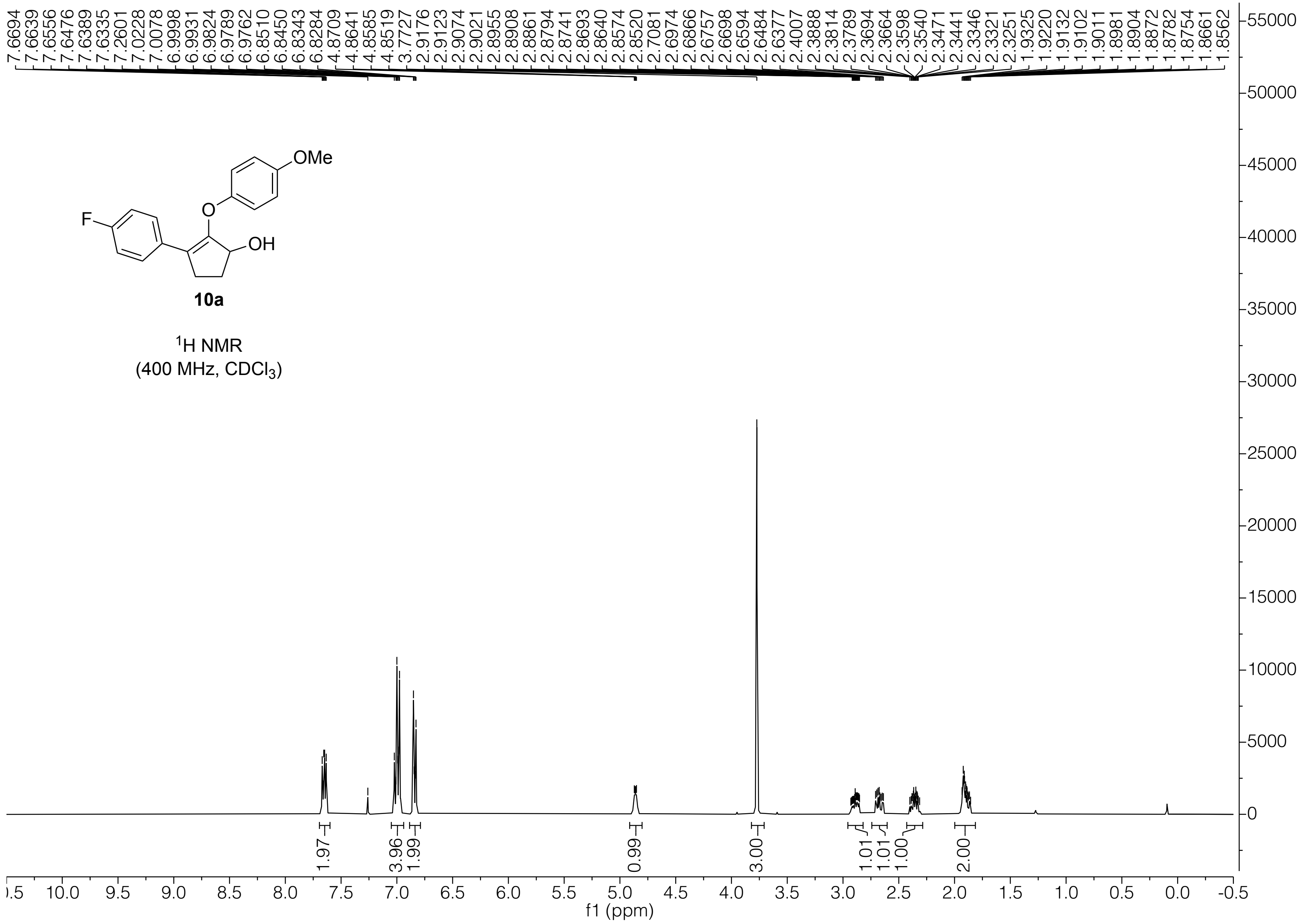
7.6694
7.6639
7.6556
7.6476
7.6389
7.6335
7.2601
7.0228
7.0078
6.9998
6.9931
6.9824
6.9789
6.9762
6.8510
6.8450
6.8343
6.8284
4.8709
4.8641
4.8585
4.8519
3.7727
2.9176
2.9123
2.9074
2.9021
2.8955
2.8908
2.8861
2.8794
2.8741
2.8693
2.8640
2.8574
2.8520
2.7081
2.6974
2.6866
2.6757
2.6698
2.6594
2.6484
2.6377
2.4007
2.3888
2.3814
2.3789
2.3694
2.3664
2.3598
2.3540
2.3471
2.3441
2.3346
2.3321
2.3251
1.9325
1.9220
1.9132
1.9102
1.9011
1.8981
1.8904
1.8872
1.8782
1.8754
1.8661
1.8562
OMe
O
F
OH
10a
1H NMR
(400 MHz, CDCl3)
1.97
3.96
1.99
0.99
3.00
1.01
1.01
1.00
2.00
f1 (ppm)

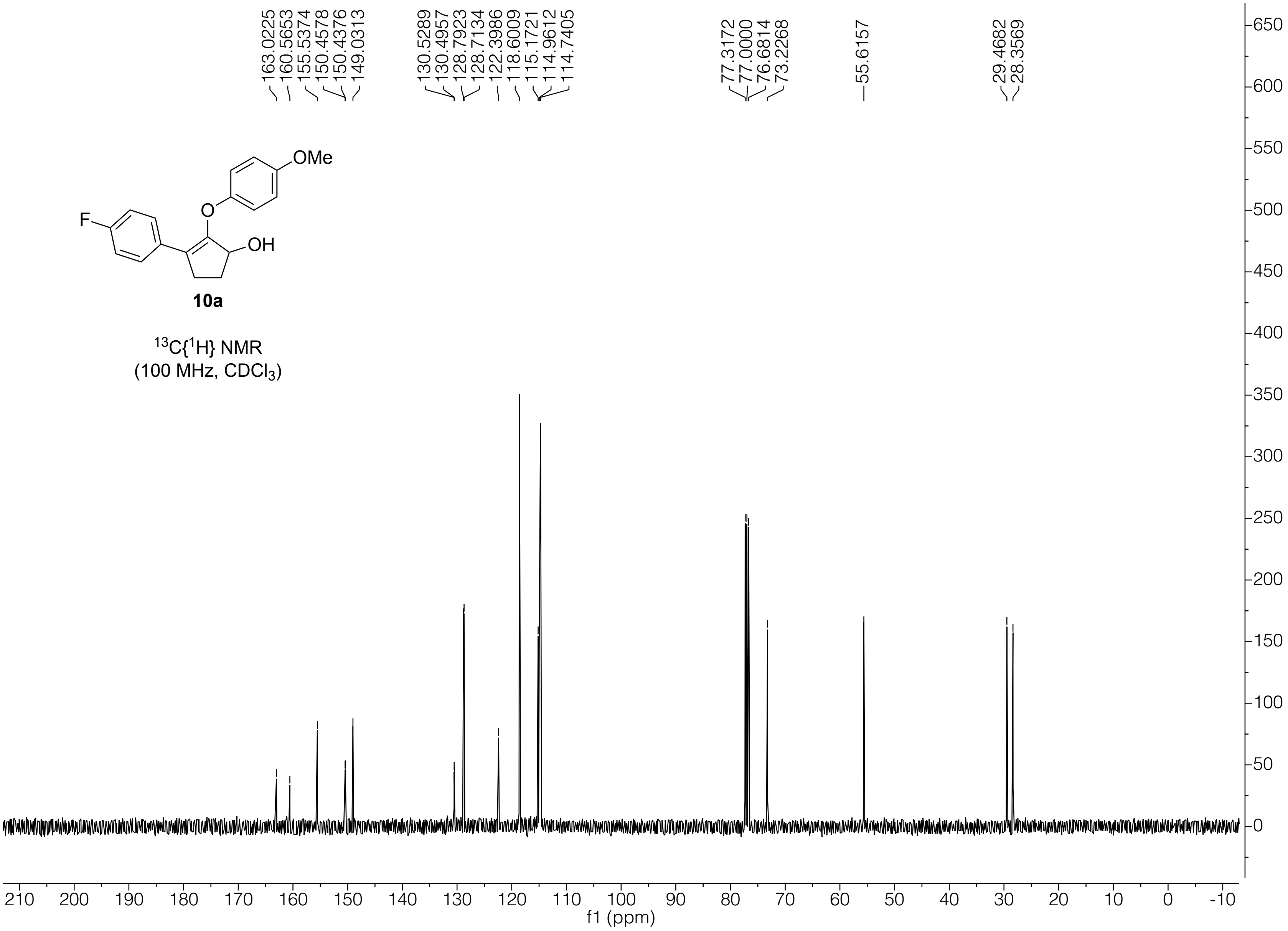
163.0225
160.5653
155.5374
150.4578
150.4376
149.0313
130.5289
130.4957
128.7923
128.7134
122.3986
118.6009
115.1721
114.9612
114.7405
77.3172
77.0000
76.6814
73.2268
55.6157
29.4682
28.3569
OMe
O
F
OH
10a
$^{13}C\{^1H\}$ NMR
(100 MHz, $CDCl_3$)
f1 (ppm)

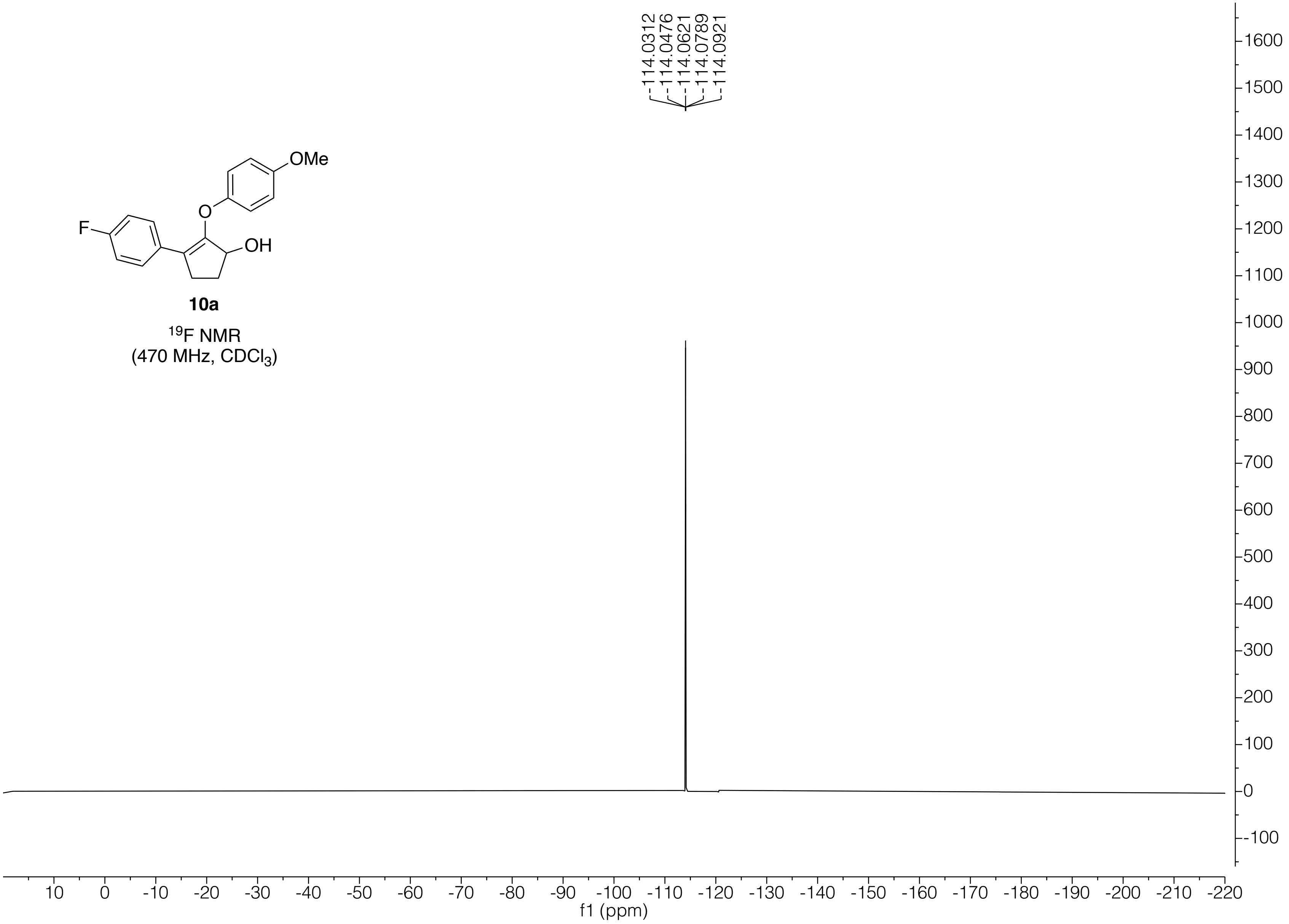

10a

^{19}F NMR
(470 MHz, $CDCl_3$)

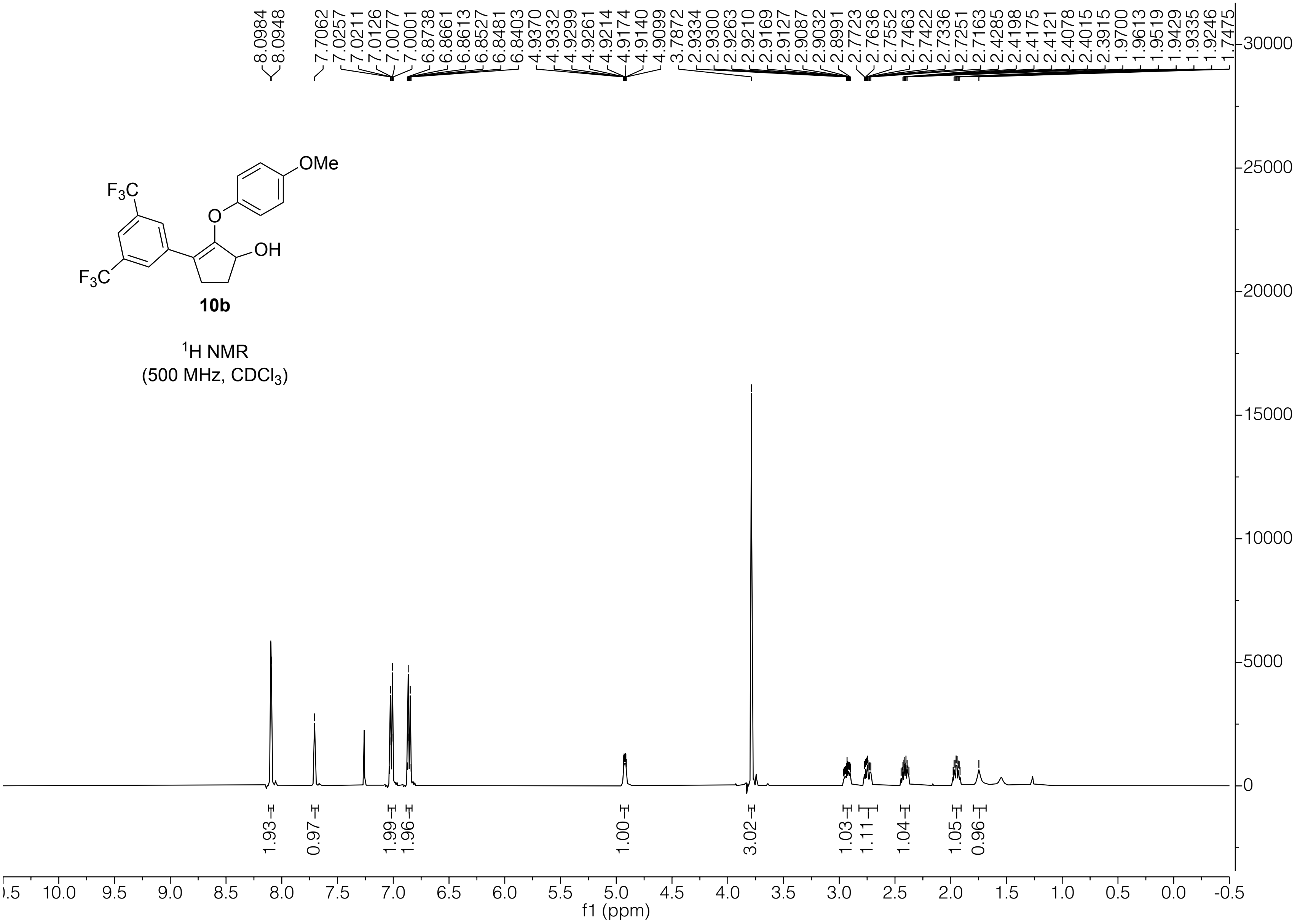
OMe
F3C
O
OH
F3C
10b
1H NMR
(500 MHz, CDCl3)
8.0984
8.0948
7.7062
7.0257
7.0211
7.0126
7.0077
7.0001
6.8738
6.8661
6.8613
6.8527
6.8481
6.8403
4.9370
4.9332
4.9299
4.9261
4.9214
4.9174
4.9140
4.9099
3.7872
2.9334
2.9300
2.9263
2.9210
2.9169
2.9127
2.9087
2.9032
2.8991
2.7723
2.7636
2.7552
2.7463
2.7422
2.7336
2.7251
2.7163
2.4285
2.4198
2.4175
2.4121
2.4078
2.4015
2.3915
1.9700
1.9613
1.9519
1.9429
1.9335
1.9246
1.7475
1.93
0.97
1.99
1.96
1.00
3.02
1.03
1.11
1.04
1.05
0.96
f1 (ppm)

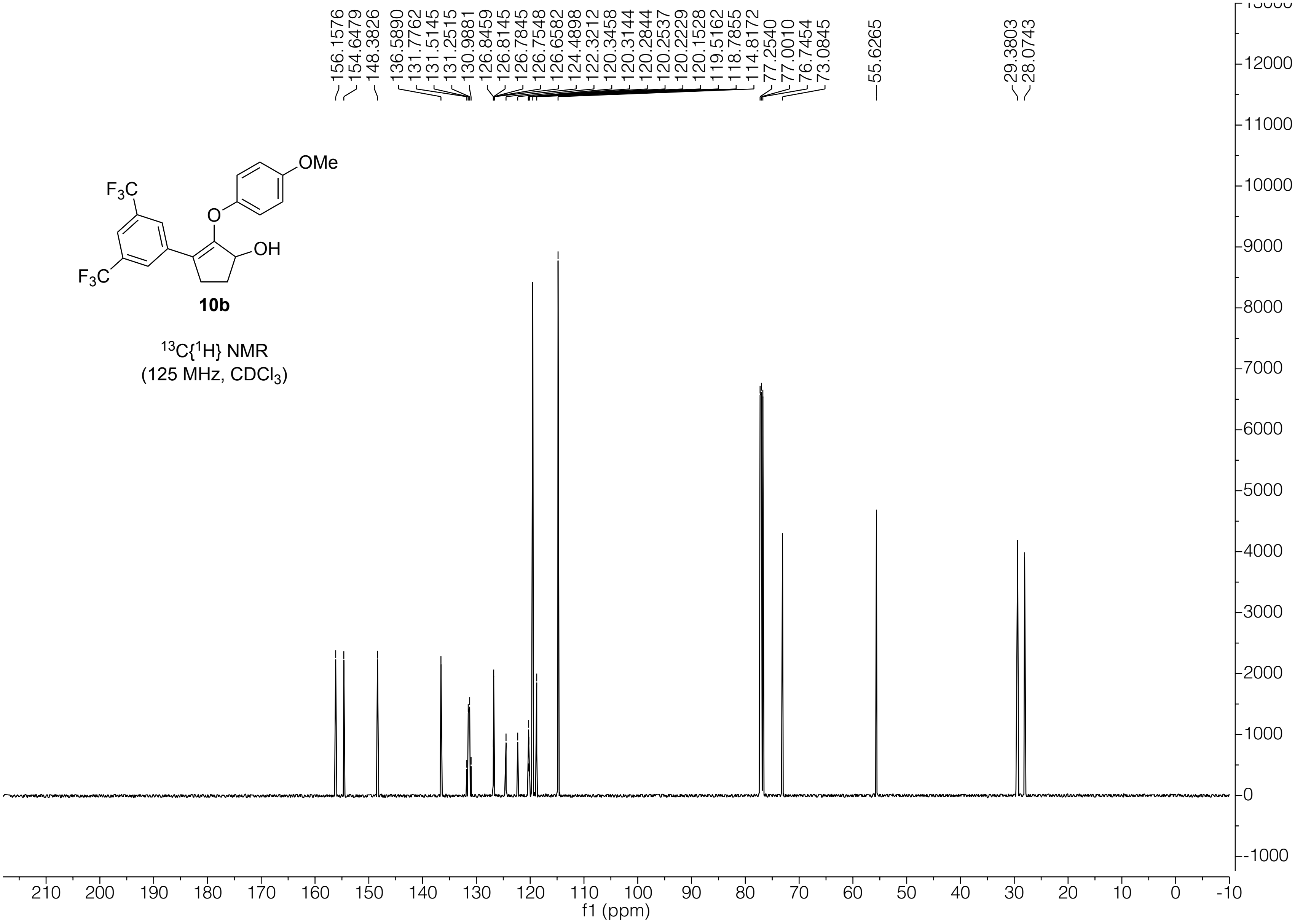
156.1576
154.6479
148.3826
136.5890
131.7762
131.5145
131.2515
130.9881
126.8459
126.8145
126.7845
126.7548
126.6582
124.4898
122.3212
120.3458
120.3144
120.2844
120.2537
120.2229
120.1528
119.5162
118.7855
114.8172
77.2540
77.0010
76.7454
73.0845
55.6265
29.3803
28.0743
OMe
F_3C
O
OH
F_3C
10b
$^{13}C\{^1H\}$ NMR
(125 MHz, $CDCl_3$)
f1 (ppm)

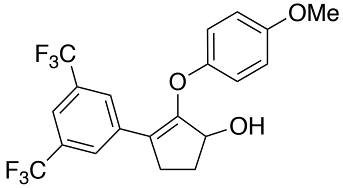

10b

^{19}F NMR
(470 MHz, $CDCl_3$)

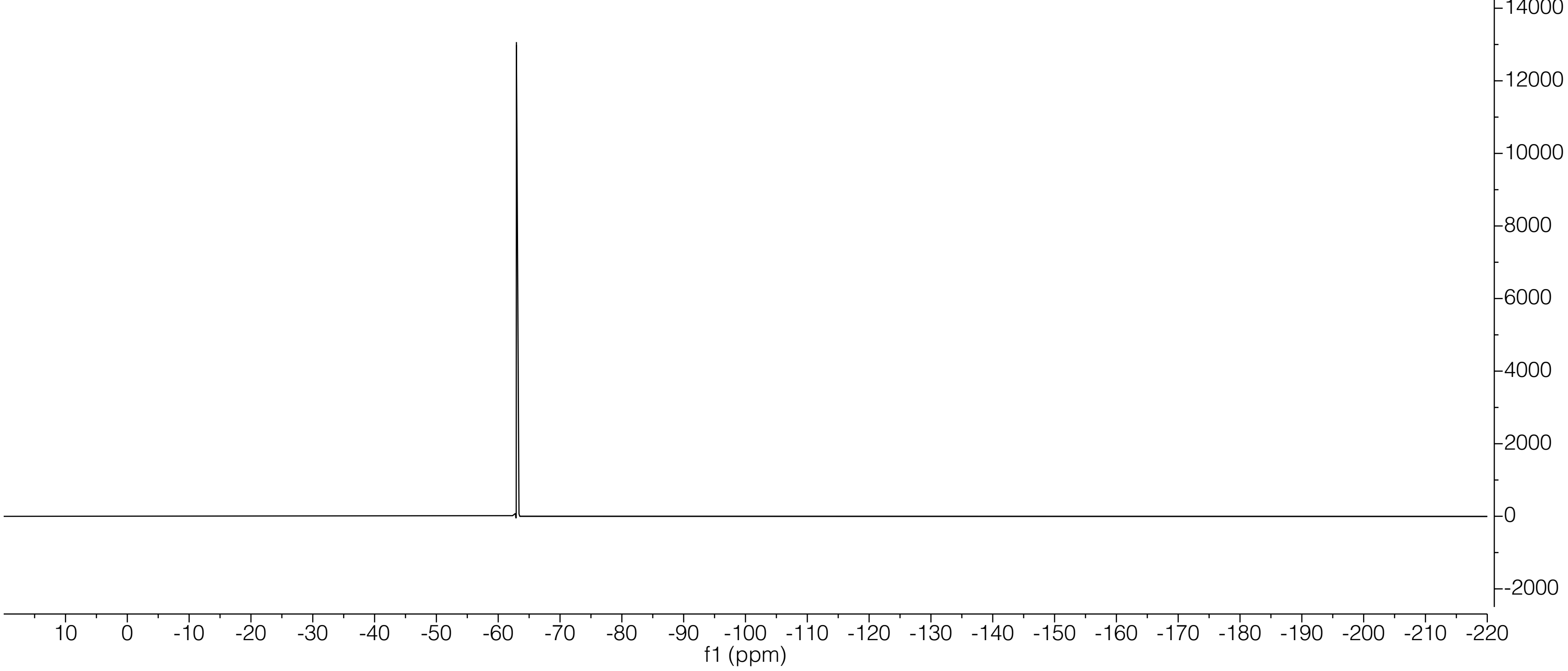

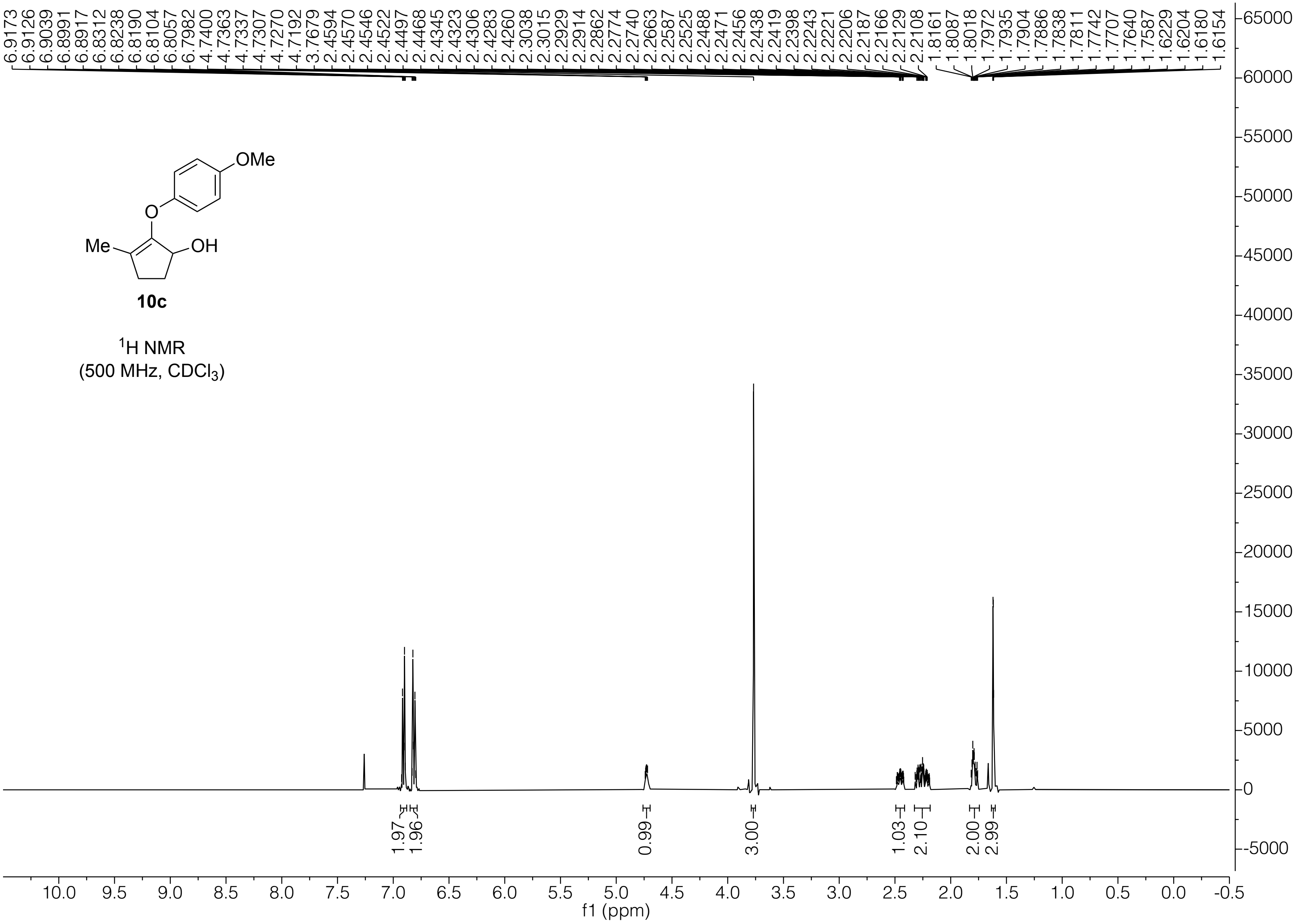
6.9173
6.9126
6.9039
6.8991
6.8917
6.8312
6.8238
6.8190
6.8104
6.8057
6.7982
4.7400
4.7363
4.7337
4.7307
4.7270
4.7192
3.7679
2.4594
2.4570
2.4546
2.4522
2.4497
2.4468
2.4345
2.4323
2.4306
2.4283
2.4260
2.3038
2.3015
2.2929
2.2914
2.2862
2.2774
2.2740
2.2663
2.2587
2.2525
2.2488
2.2471
2.2456
2.2438
2.2419
2.2398
2.2243
2.2221
2.2206
2.2187
2.2166
2.2129
2.2108
1.8161
1.8087
1.8018
1.7972
1.7935
1.7904
1.7886
1.7838
1.7811
1.7742
1.7707
1.7640
1.7587
1.6229
1.6204
1.6180
1.6154
OMe
O
Me
OH
10c
1H NMR
(500 MHz, CDCl3)
1.97
1.96
0.99
3.00
1.03
2.10
2.00
2.99
f1 (ppm)

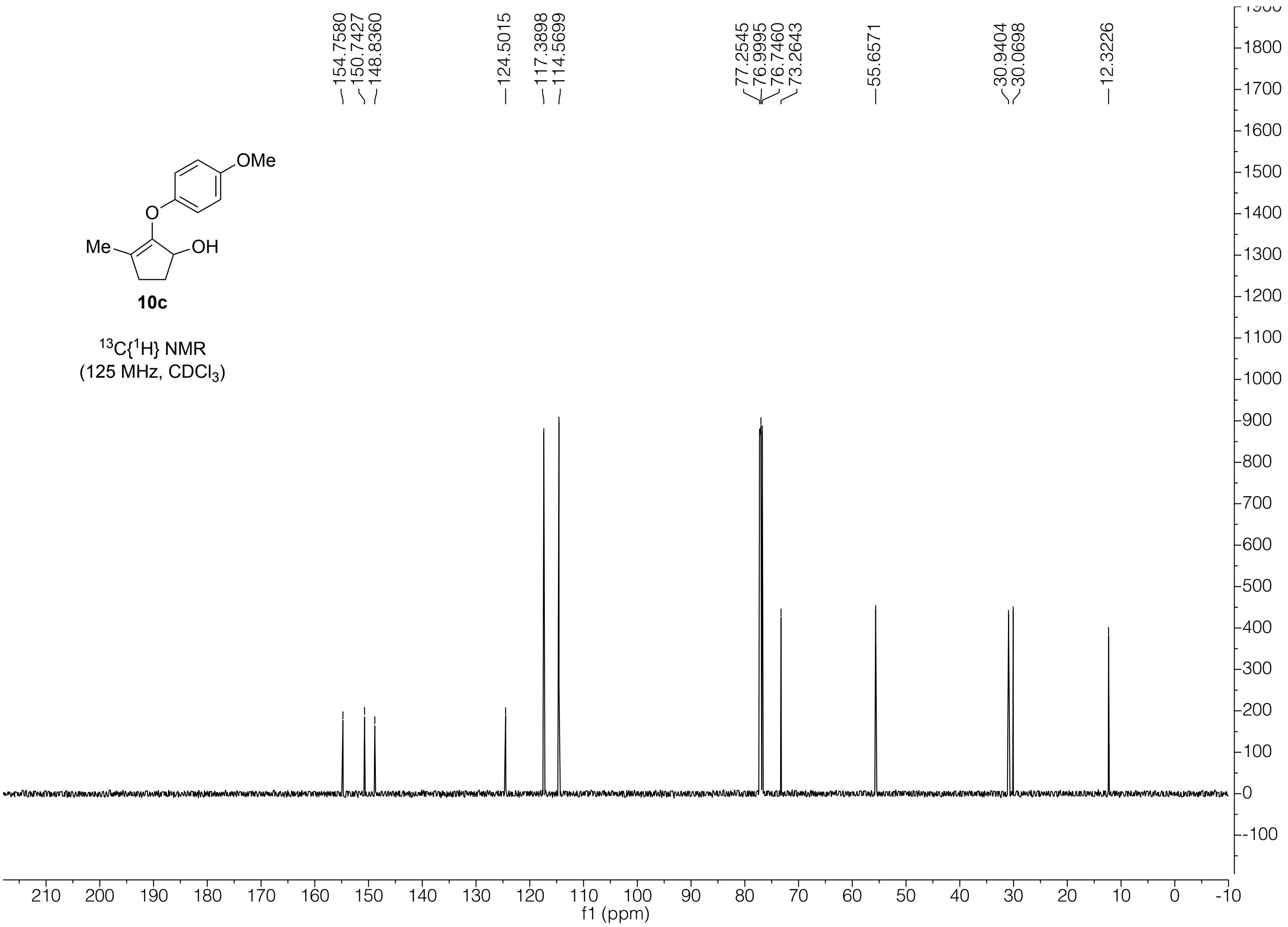
154.7580
150.7427
148.8360
124.5015
117.3898
114.5699
77.2545
76.9995
76.7460
73.2643
55.6571
30.9404
30.0698
12.3226
OMe
O
Me
OH
10c
$^{13}C\{^1H\}$ NMR
(125 MHz, $CDCl_3$)
f1 (ppm)

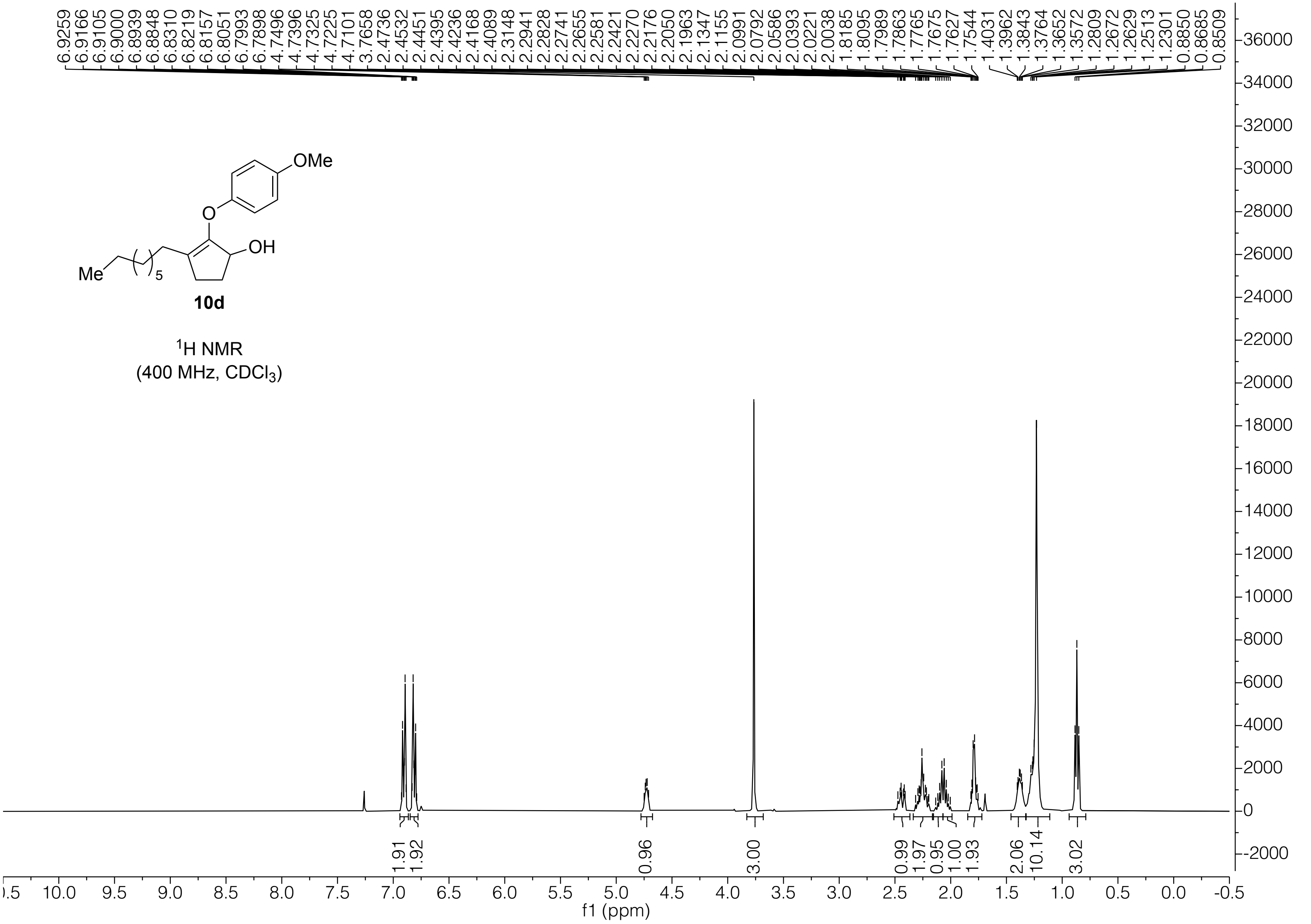

OMe
O
OH
Me
5
10d
^{1}H NMR
(400 MHz, $CDCl_3$)
6.9259
6.9166
6.9105
6.9000
6.8939
6.8848
6.8310
6.8219
6.8157
6.8051
6.7993
6.7898
4.7496
4.7396
4.7325
4.7225
4.7101
3.7658
2.4736
2.4532
2.4451
2.4395
2.4236
2.4168
2.4089
2.3148
2.2941
2.2828
2.2741
2.2655
2.2581
2.2421
2.2270
2.2176
2.2050
2.1963
2.1347
2.1155
2.0991
2.0792
2.0586
2.0393
2.0221
2.0038
1.8185
1.8095
1.7989
1.7863
1.7765
1.7675
1.7627
1.7544
1.4031
1.3962
1.3843
1.3764
1.3652
1.3572
1.2809
1.2672
1.2629
1.2513
1.2301
0.8850
0.8685
0.8509
1.91
1.92
0.96
3.00
0.99
1.97
0.95
1.00
1.93
2.06
10.14
3.02
f1 (ppm)

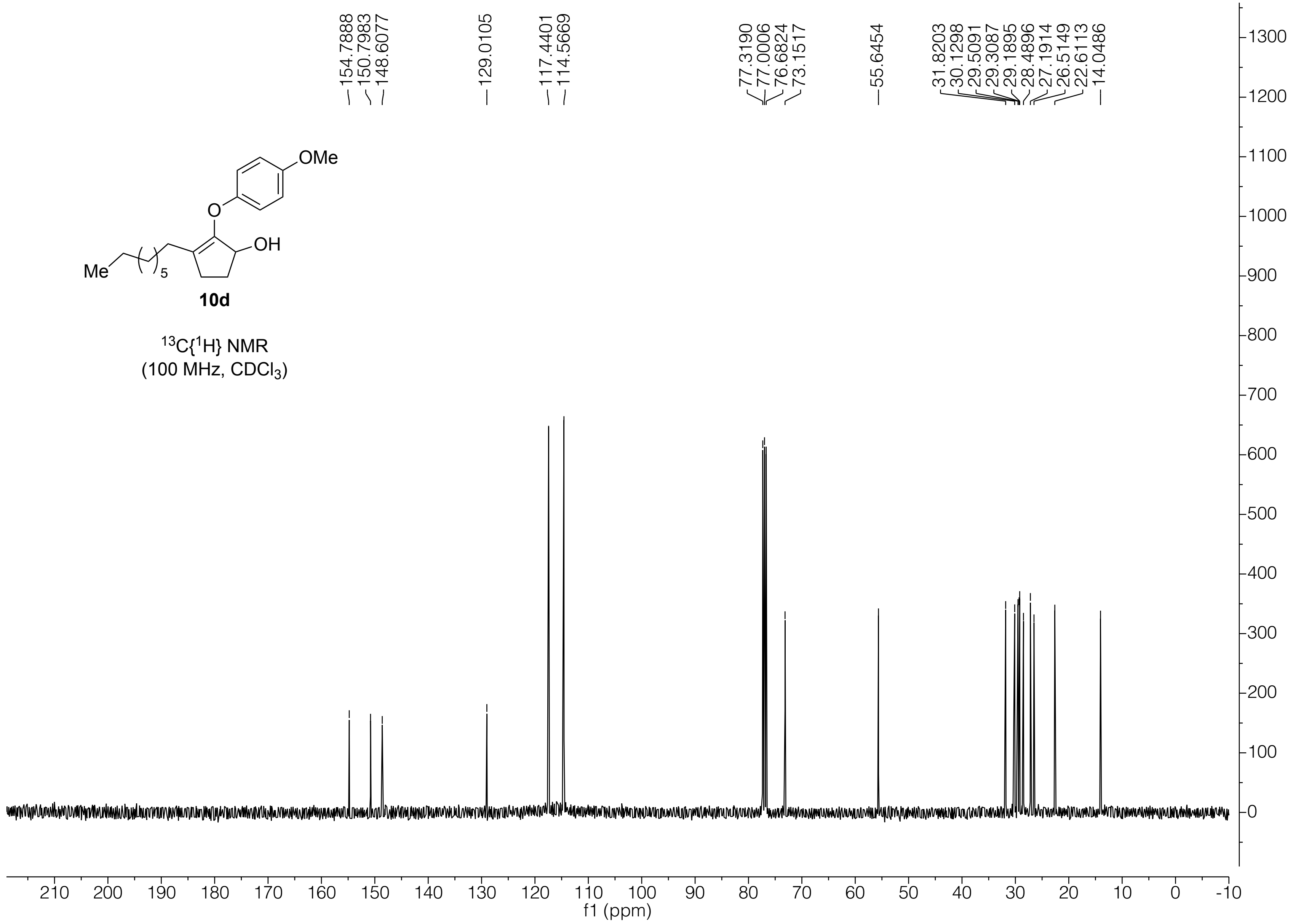

10d

$^{13}C\{^{1}H\}$ NMR
(100 MHz, $CDCl_3$)

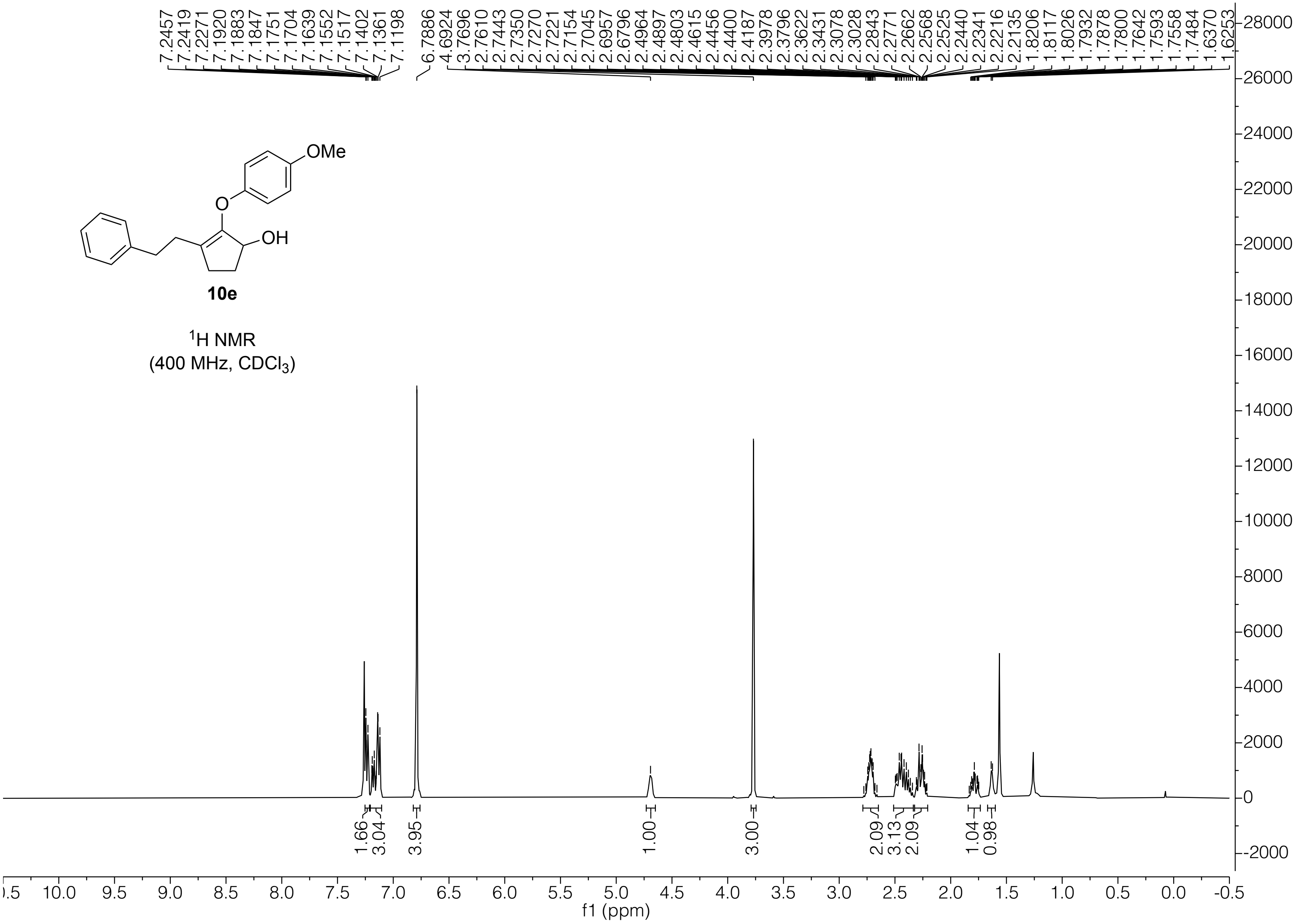
OMe
O
OH
10e
1H NMR
(400 MHz, CDCl3)
7.2457
7.2419
7.2271
7.1920
7.1883
7.1847
7.1751
7.1704
7.1639
7.1552
7.1517
7.1402
7.1361
7.1198
6.7886
4.6924
3.7696
2.7610
2.7443
2.7350
2.7270
2.7221
2.7154
2.7045
2.6957
2.6796
2.4964
2.4897
2.4803
2.4615
2.4456
2.4400
2.4187
2.3978
2.3796
2.3622
2.3431
2.3078
2.3028
2.2843
2.2771
2.2662
2.2568
2.2525
2.2440
2.2341
2.2216
2.2135
1.8206
1.8117
1.8026
1.7932
1.7878
1.7800
1.7642
1.7593
1.7558
1.7484
1.6370
1.6253
1.66
3.04
3.95
1.00
3.00
2.09
3.13
2.09
1.04
0.98
f1 (ppm)

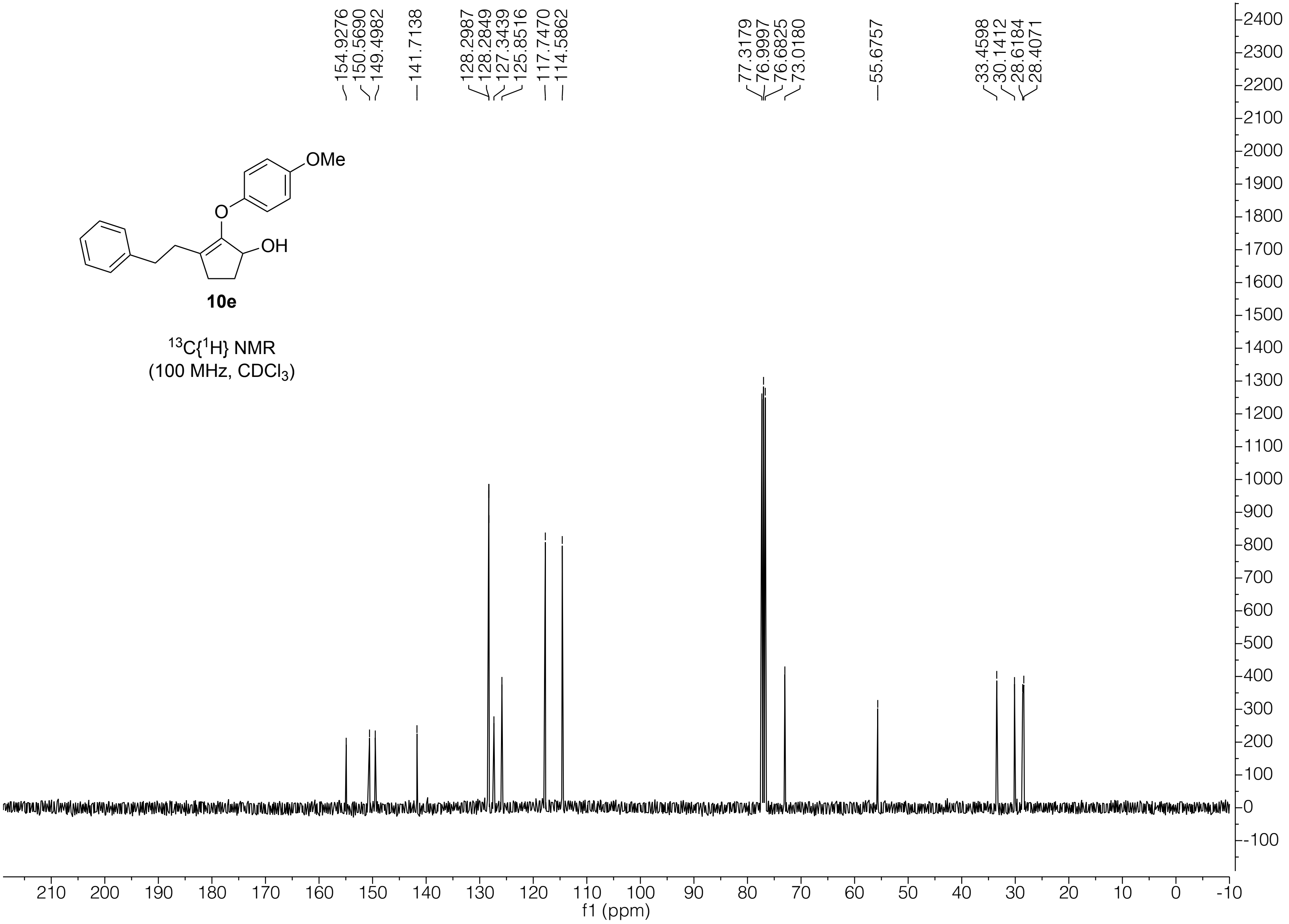

154.9276
150.5690
149.4982
141.7138
128.2987
128.2849
127.3439
125.8516
117.7470
114.5862
77.3179
76.9997
76.6825
73.0180
55.6757
33.4598
30.1412
28.6184
28.4071
OMe
O
OH
10e
13C{1H} NMR
(100 MHz, CDCl3)
f1 (ppm)

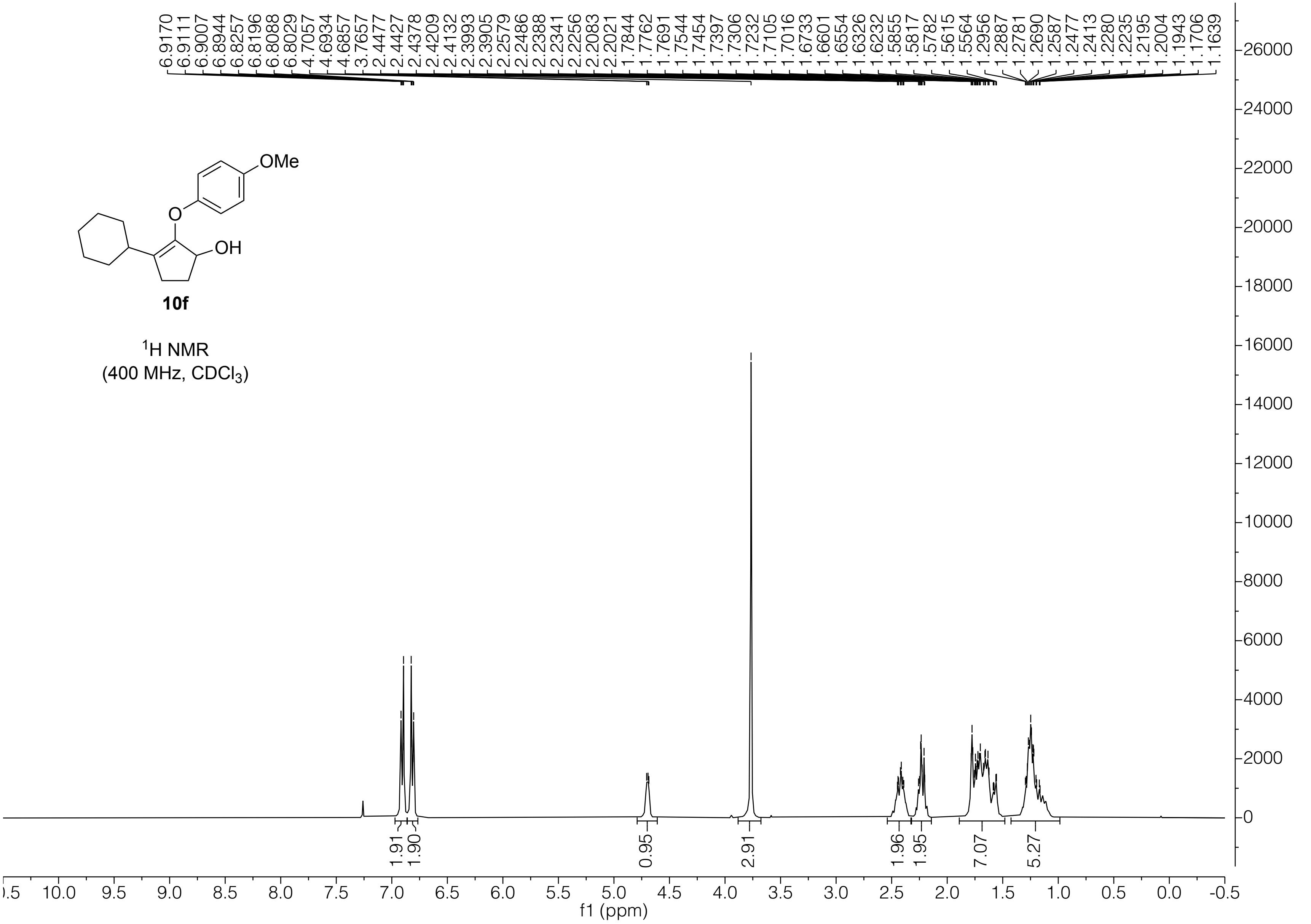

6.9170
6.9111
6.9007
6.8944
6.8257
6.8196
6.8088
6.8029
4.7057
4.6934
4.6857
3.7657
2.4477
2.4427
2.4378
2.4209
2.4132
2.3993
2.3905
2.2579
2.2486
2.2388
2.2341
2.2256
2.2083
2.2021
1.7844
1.7762
1.7691
1.7544
1.7454
1.7397
1.7306
1.7232
1.7105
1.7016
1.6733
1.6601
1.6554
1.6326
1.6232
1.5855
1.5817
1.5782
1.5615
1.5564
1.2956
1.2887
1.2781
1.2690
1.2587
1.2477
1.2413
1.2280
1.2235
1.2195
1.2004
1.1943
1.1706
1.1639
OMe
O
OH
10f
^{1}H NMR
(400 MHz, $CDCl_3$)
1.91
1.90
0.95
2.91
1.96
1.95
7.07
5.27
f1 (ppm)

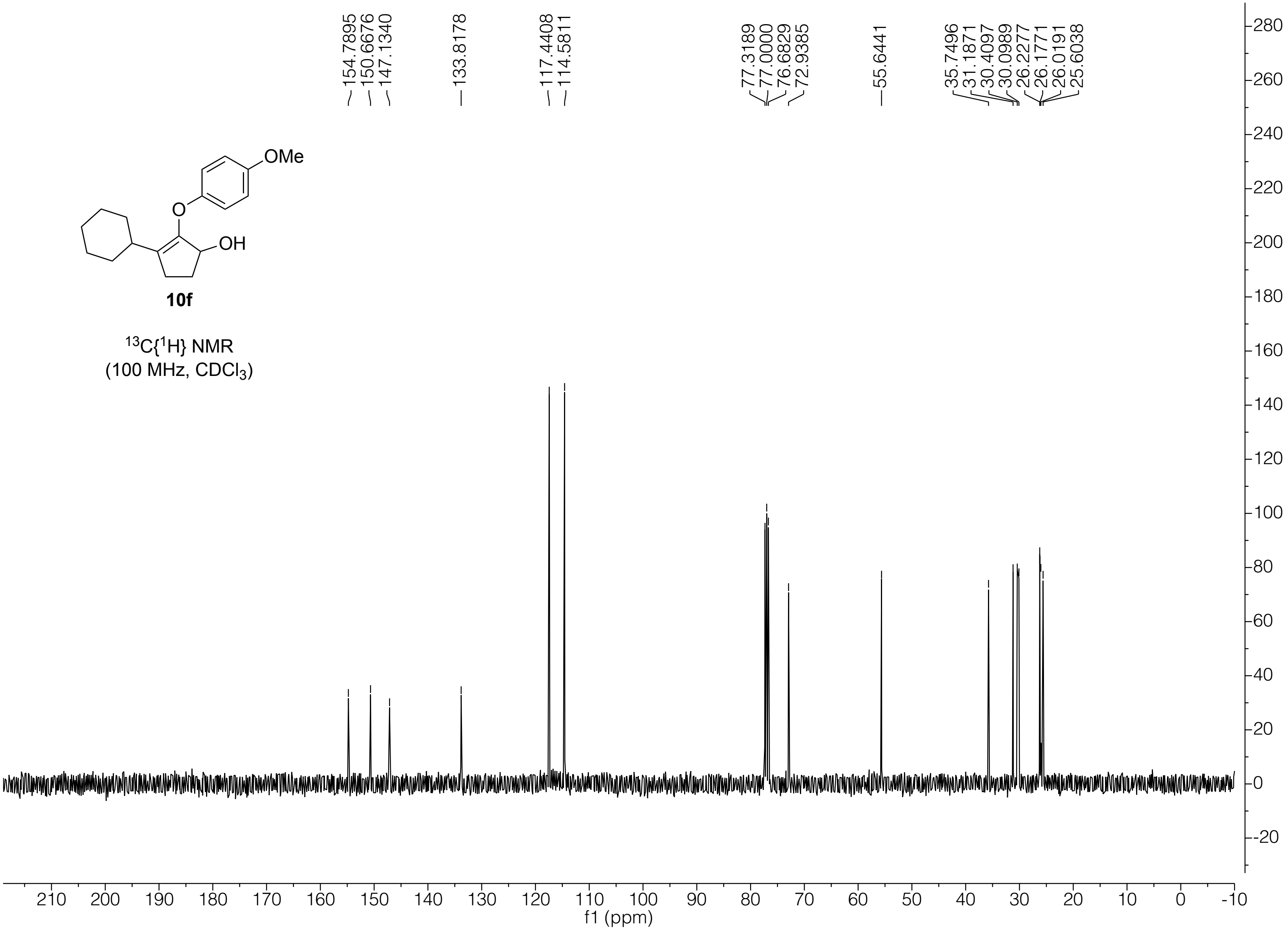
OMe
O
OH
10f
$^{13}C\{^1H\}$ NMR
(100 MHz, $CDCl_3$)
154.7895
150.6676
147.1340
133.8178
117.4408
114.5811
77.3189
77.0000
76.6829
72.9385
55.6441
35.7496
31.1871
30.4097
30.0989
26.2277
26.1771
26.0191
25.6038
f1 (ppm)

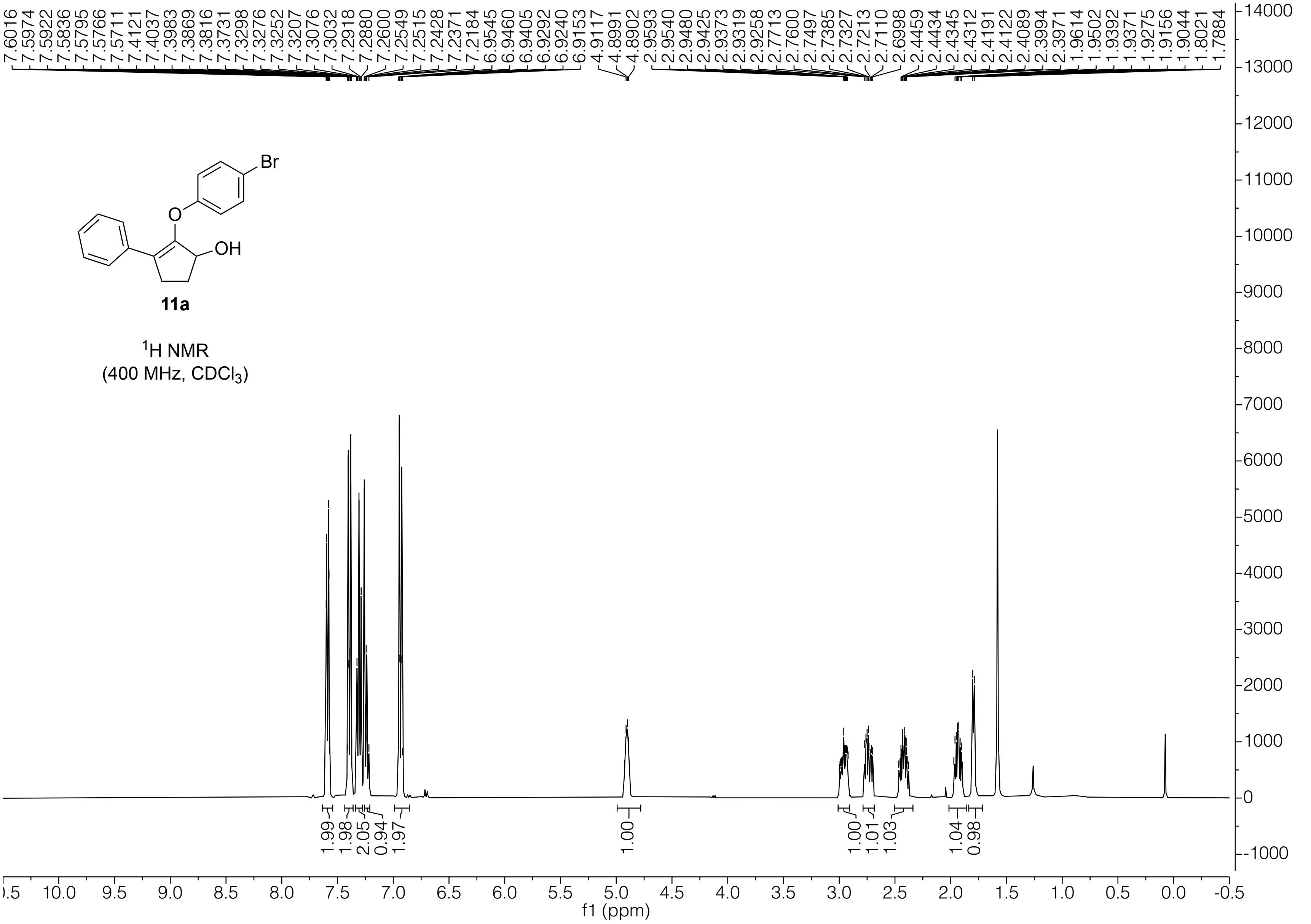

Br
O
OH
11a
1H NMR
(400 MHz, CDCl3)
f1 (ppm)

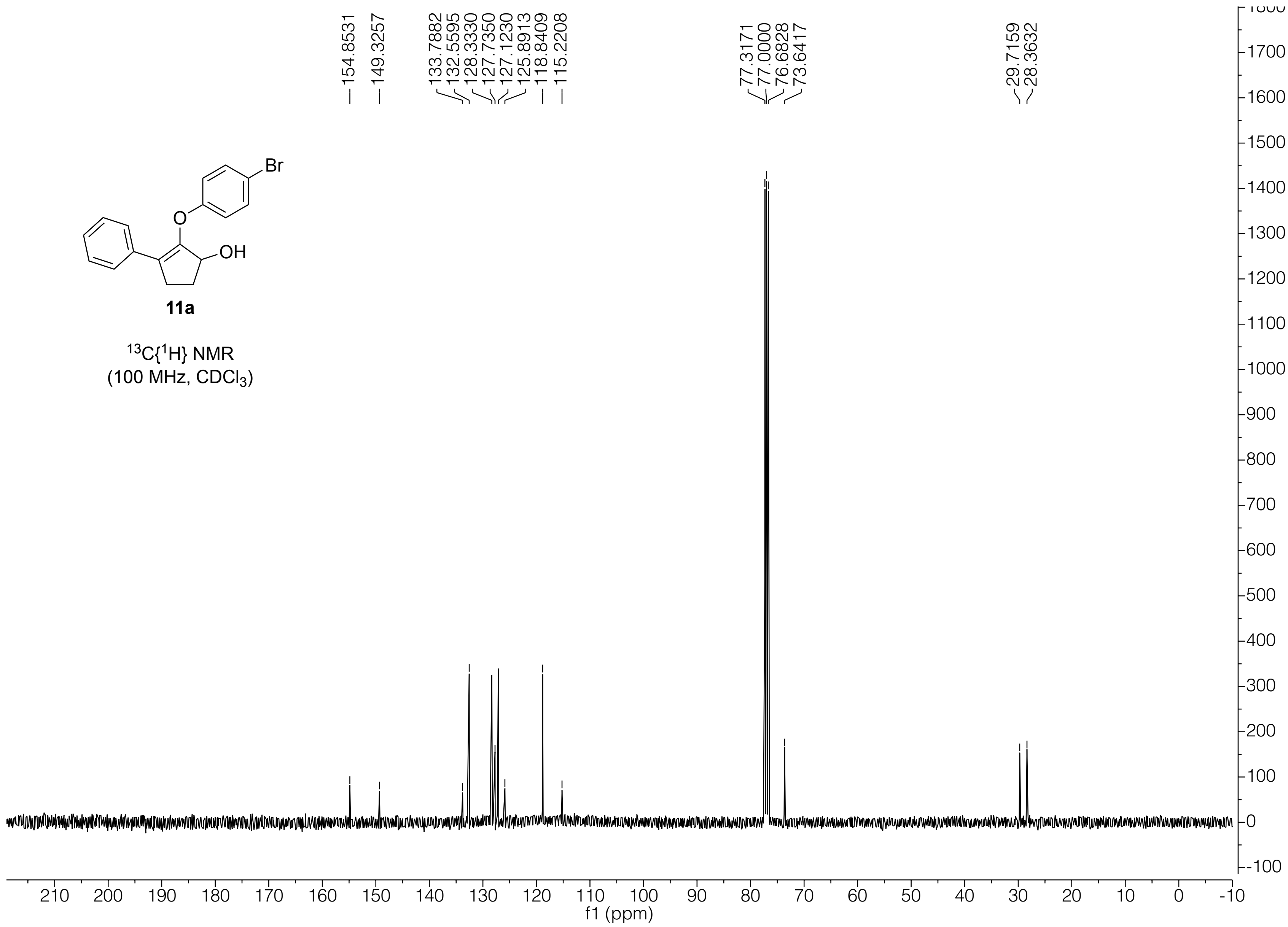
154.8531
149.3257
133.7882
132.5595
128.3330
127.7350
127.1230
125.8913
118.8409
115.2208
77.3171
77.0000
76.6828
73.6417
29.7159
28.3632
Br
O
OH
11a
$^{13}C\{^1H\}$ NMR
(100 MHz, $CDCl_3$)
f1 (ppm)

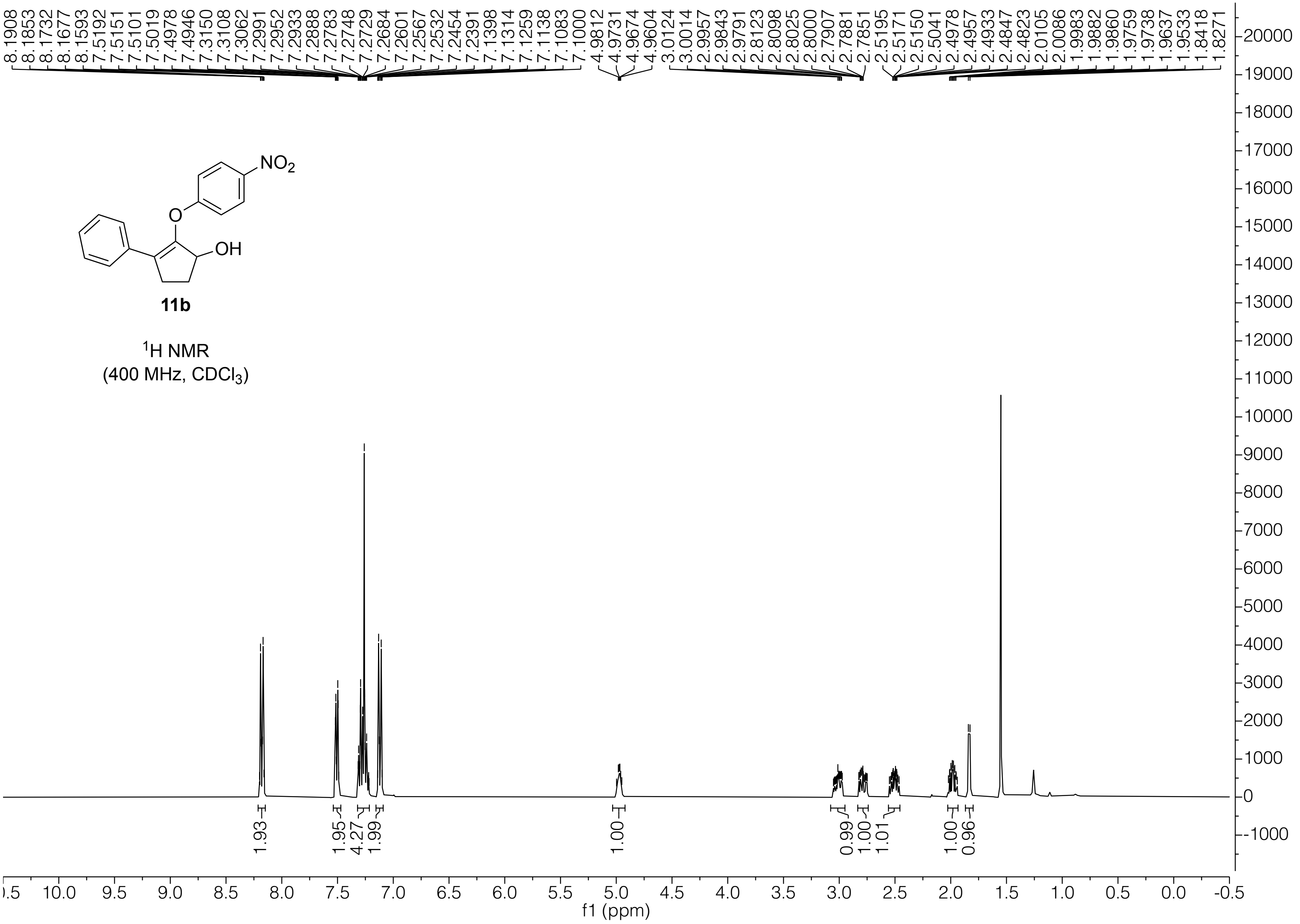
8.1908
8.1853
8.1732
8.1677
8.1593
7.5192
7.5151
7.5101
7.5019
7.4978
7.4946
7.3150
7.3108
7.3062
7.2991
7.2952
7.2933
7.2888
7.2783
7.2748
7.2729
7.2684
7.2601
7.2567
7.2532
7.2454
7.2391
7.1398
7.1314
7.1259
7.1138
7.1083
7.1000
4.9812
4.9731
4.9674
4.9604
3.0124
3.0014
2.9957
2.9843
2.9791
2.8123
2.8098
2.8025
2.8000
2.7907
2.7881
2.7851
2.5195
2.5171
2.5150
2.5041
2.4978
2.4957
2.4933
2.4847
2.4823
2.0105
2.0086
1.9983
1.9882
1.9860
1.9759
1.9738
1.9637
1.9533
1.8418
1.8271
NO2
O
OH
11b
1H NMR
(400 MHz, CDCl3)
1.93
1.95
4.27
1.99
1.00
0.99
1.00
1.01
1.00
0.96
f1 (ppm)

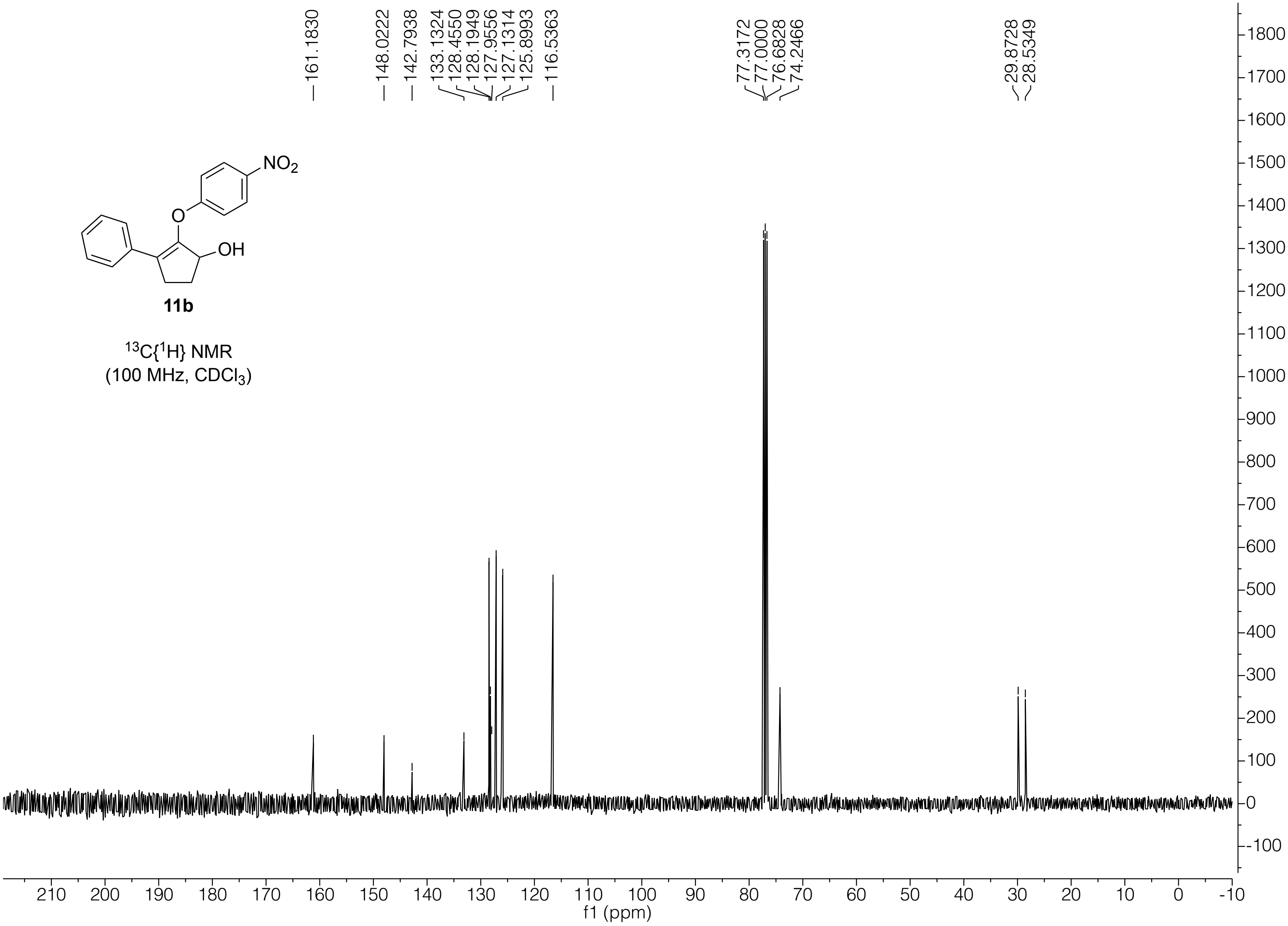

11b

$^{13}C\{^{1}H\}$ NMR
(100 MHz, $CDCl_3$)

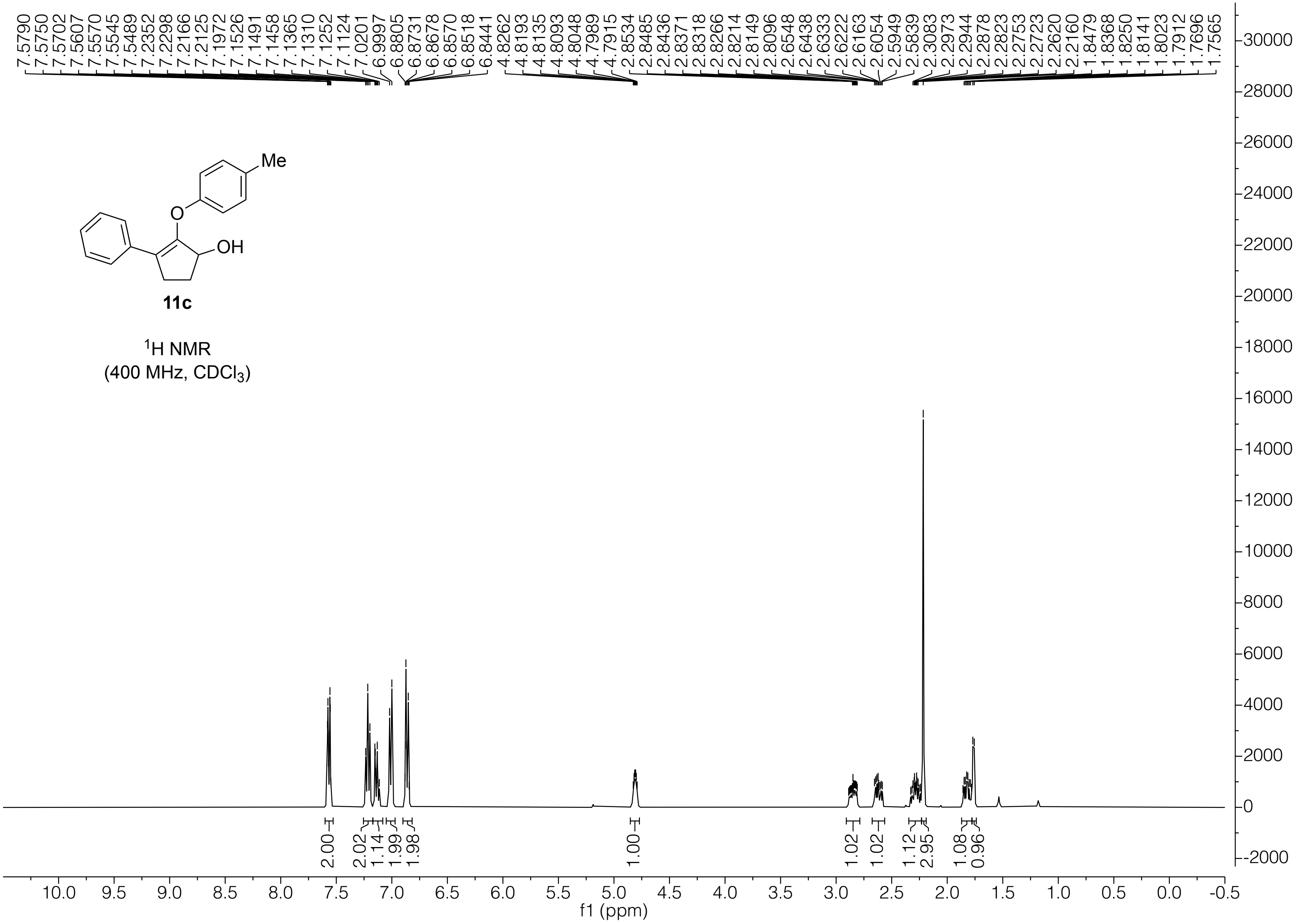
Me
O
OH
11c
^{1}H NMR
(400 MHz, $CDCl_3$)
f1 (ppm)

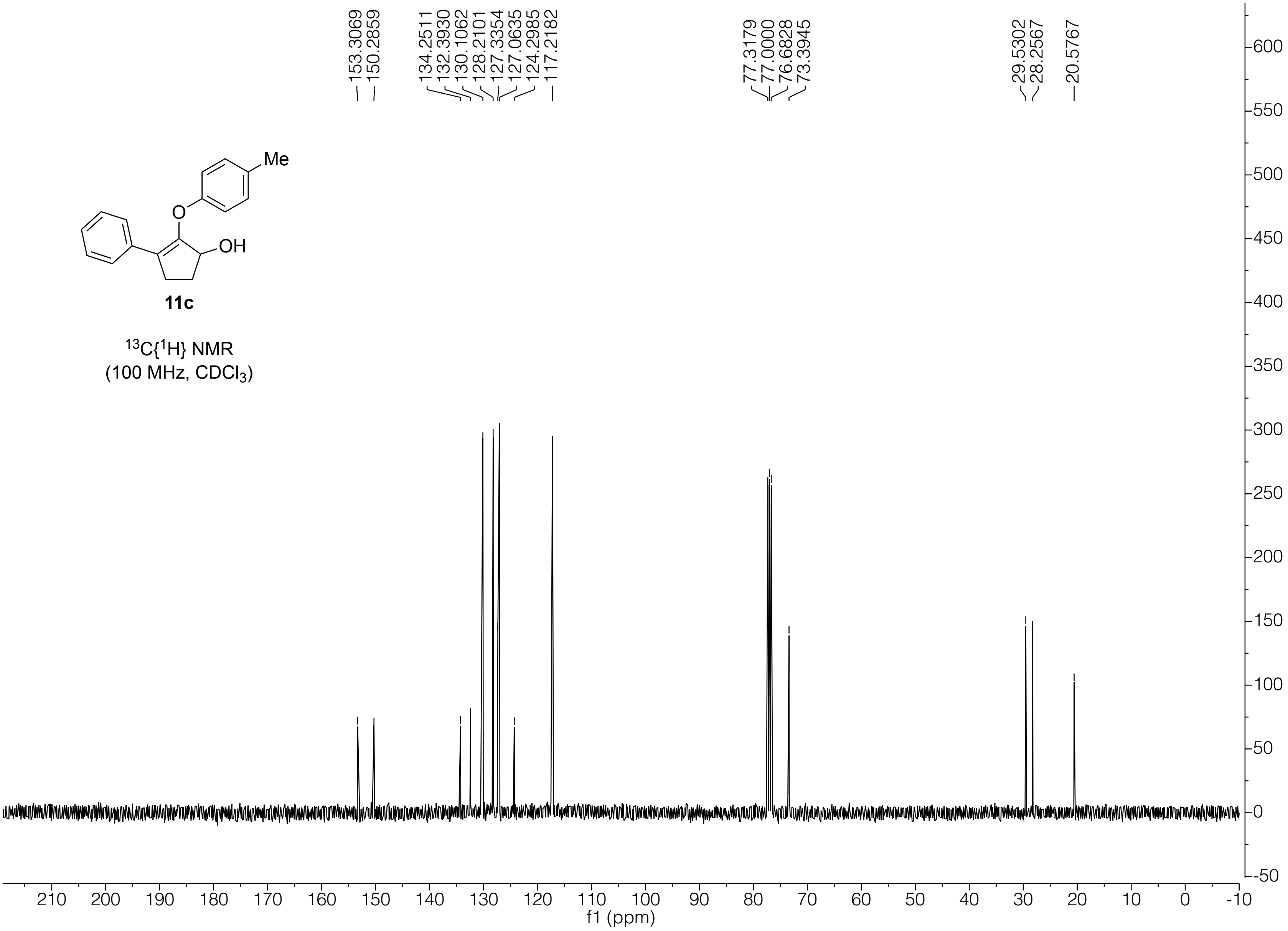
153.3069
150.2859
134.2511
132.3930
130.1062
128.2101
127.3354
127.0635
124.2985
117.2182
77.3179
77.0000
76.6828
73.3945
29.5302
28.2567
20.5767
Me
O
OH
11c
$^{13}C\{^1H\}$ NMR
(100 MHz, $CDCl_3$)
f1 (ppm)

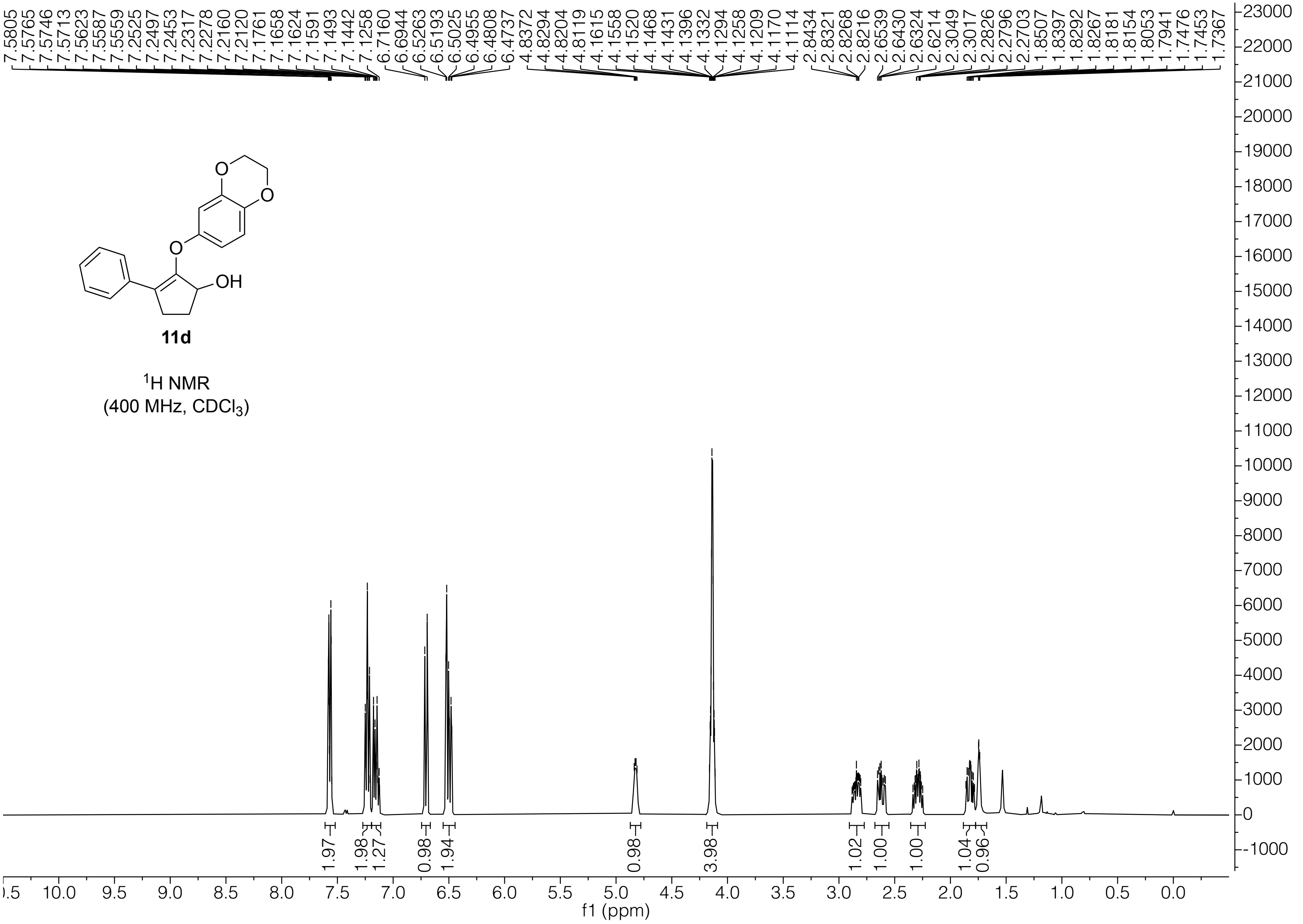
11d
^{1}H NMR
(400 MHz, $CDCl_3$)
OH
O
7.5805 7.5765 7.5746 7.5713 7.5623 7.5587 7.5559 7.2525 7.2497 7.2453 7.2317 7.2278 7.2160 7.2120 7.1761 7.1658 7.1624 7.1591 7.1493 7.1442 7.1258 7.1160 6.7160 6.6944 6.5263 6.5193 6.5025 6.4955 6.4808 6.4737 4.8372 4.8294 4.8204 4.8119 4.1615 4.1558 4.1520 4.1468 4.1431 4.1396 4.1332 4.1294 4.1258 4.1209 4.1170 4.1114 2.8434 2.8321 2.8268 2.8216 2.6539 2.6430 2.6324 2.6214 2.3049 2.3017 2.2826 2.2796 2.2703 1.8507 1.8397 1.8292 1.8267 1.8181 1.8154 1.8053 1.7941 1.7476 1.7453 1.7367
1.97 1.98 1.27 0.98 1.94 0.98 3.98 1.02 1.00 1.00 1.04 0.96
f1 (ppm)

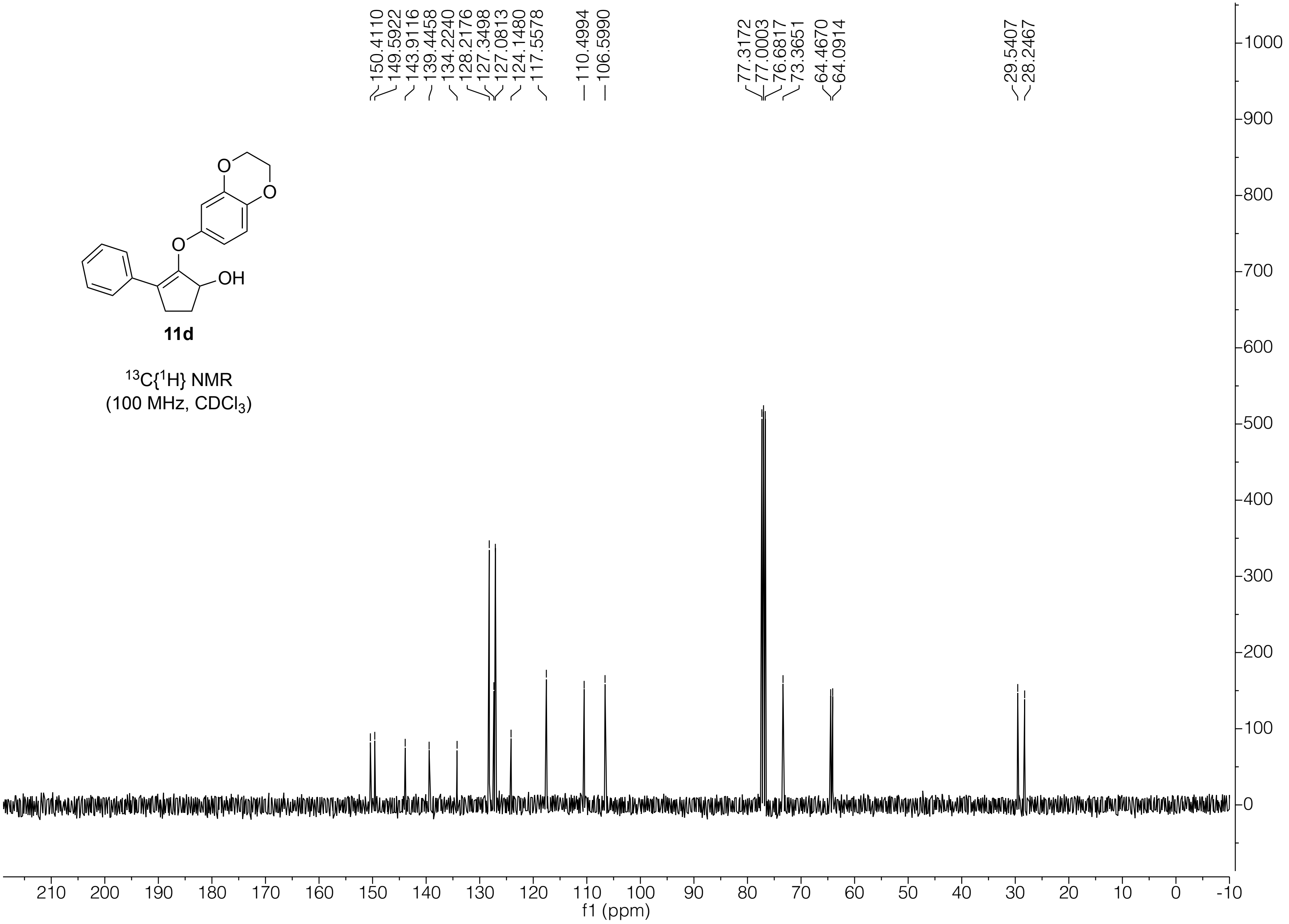

11d

$^{13}C\{^1H\}$ NMR
(100 MHz, $CDCl_3$)

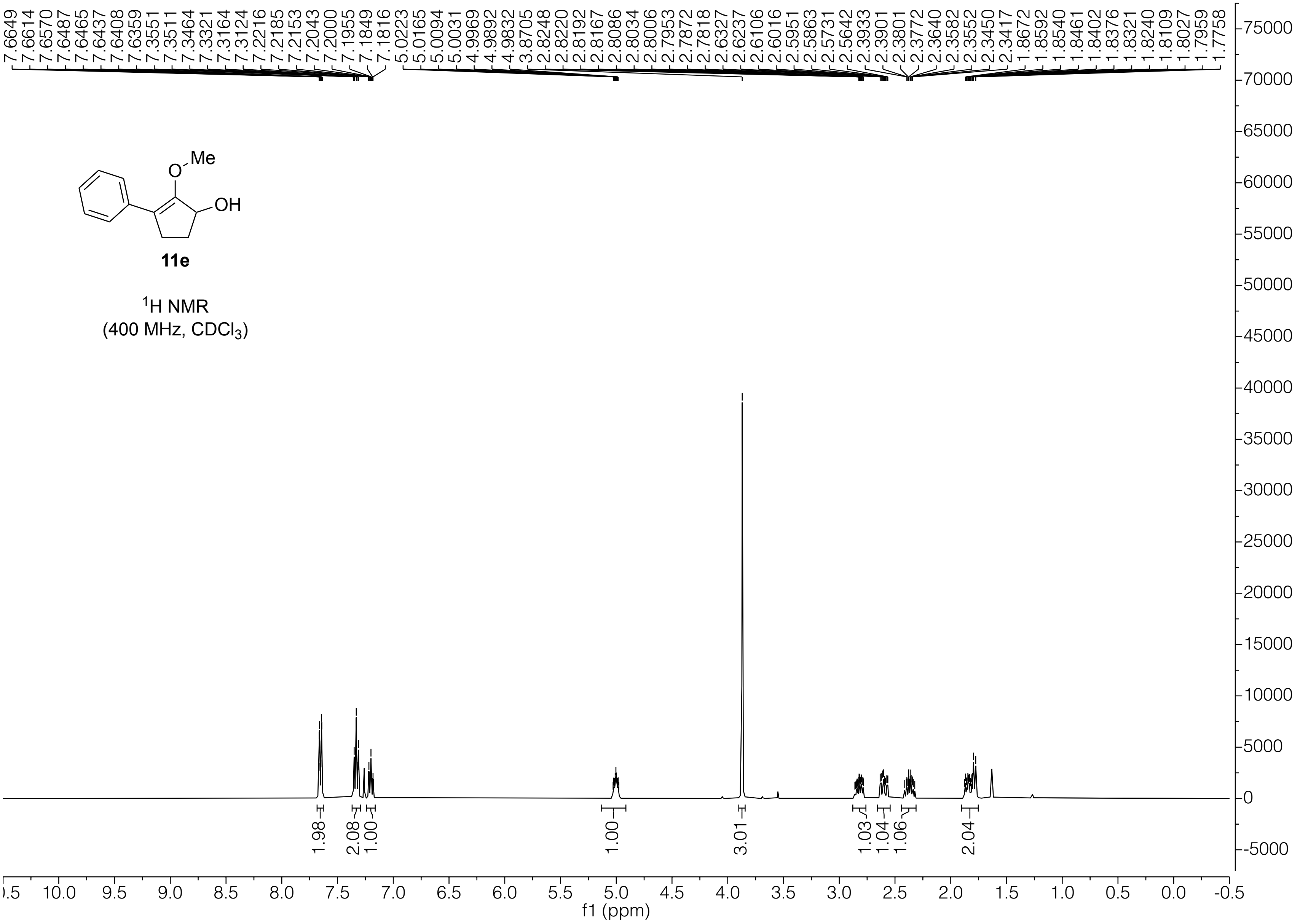
11e
^{1}H NMR
(400 MHz, CDCl$_3$)
7.6649 7.6614 7.6570 7.6487 7.6465 7.6437 7.6408 7.6359 7.3551 7.3511 7.3464 7.3321 7.3164 7.3124 7.2216 7.2185 7.2153 7.2043 7.2000 7.1955 7.1849 7.1816 5.0223 5.0165 5.0094 5.0031 4.9969 4.9892 4.9832 3.8705 2.8248 2.8220 2.8192 2.8167 2.8086 2.8034 2.8006 2.7953 2.7872 2.7818 2.6327 2.6237 2.6106 2.6016 2.5951 2.5863 2.5731 2.5642 2.3933 2.3901 2.3801 2.3772 2.3640 2.3582 2.3552 2.3450 2.3417 1.8672 1.8592 1.8540 1.8461 1.8402 1.8376 1.8321 1.8240 1.8109 1.8027 1.7959 1.7758
1.98 2.08 1.00 1.00 3.01 1.03 1.04 1.06 2.04
f1 (ppm)

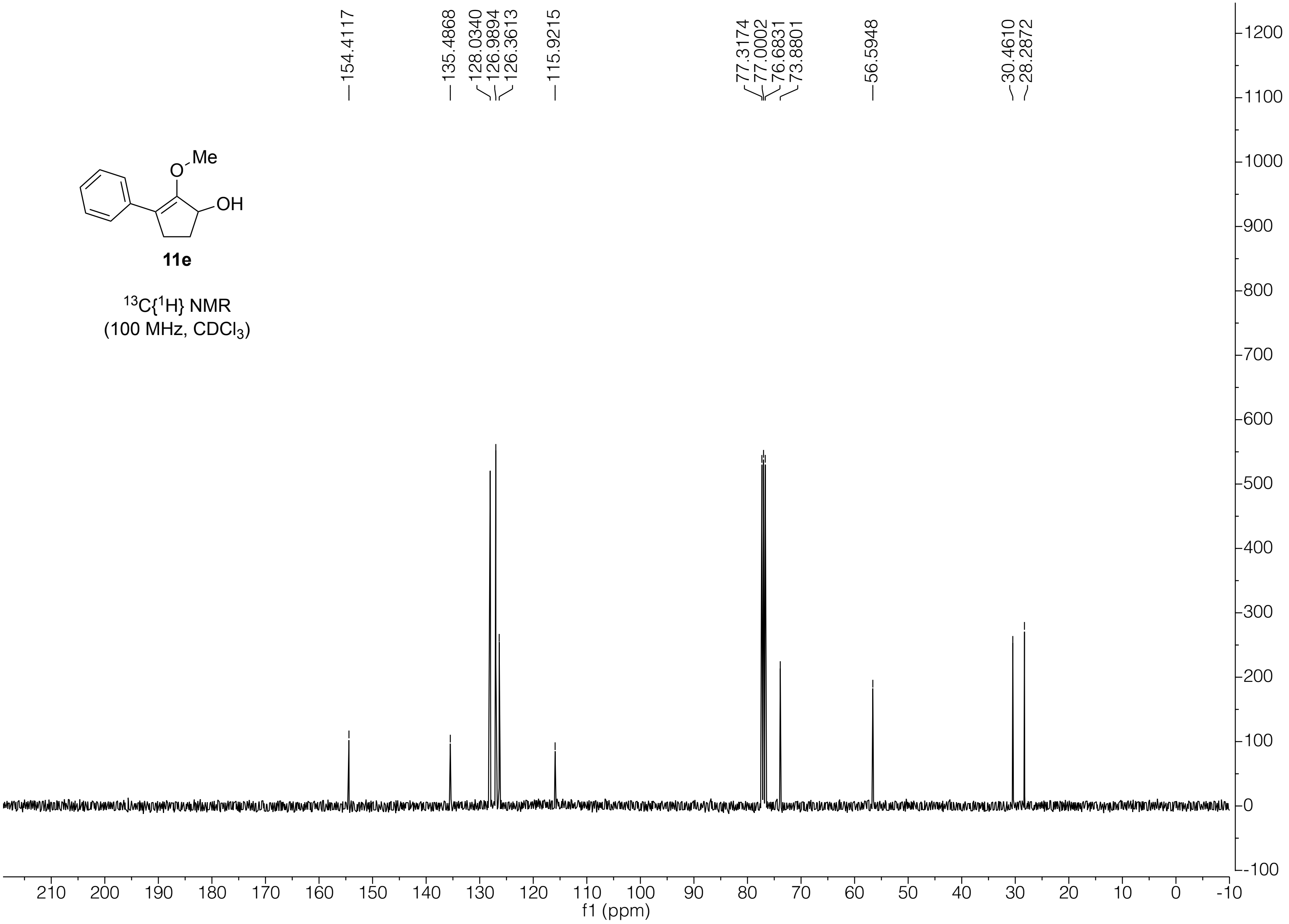

11e

$^{13}C\{^{1}H\}$ NMR
(100 MHz, $CDCl_3$)

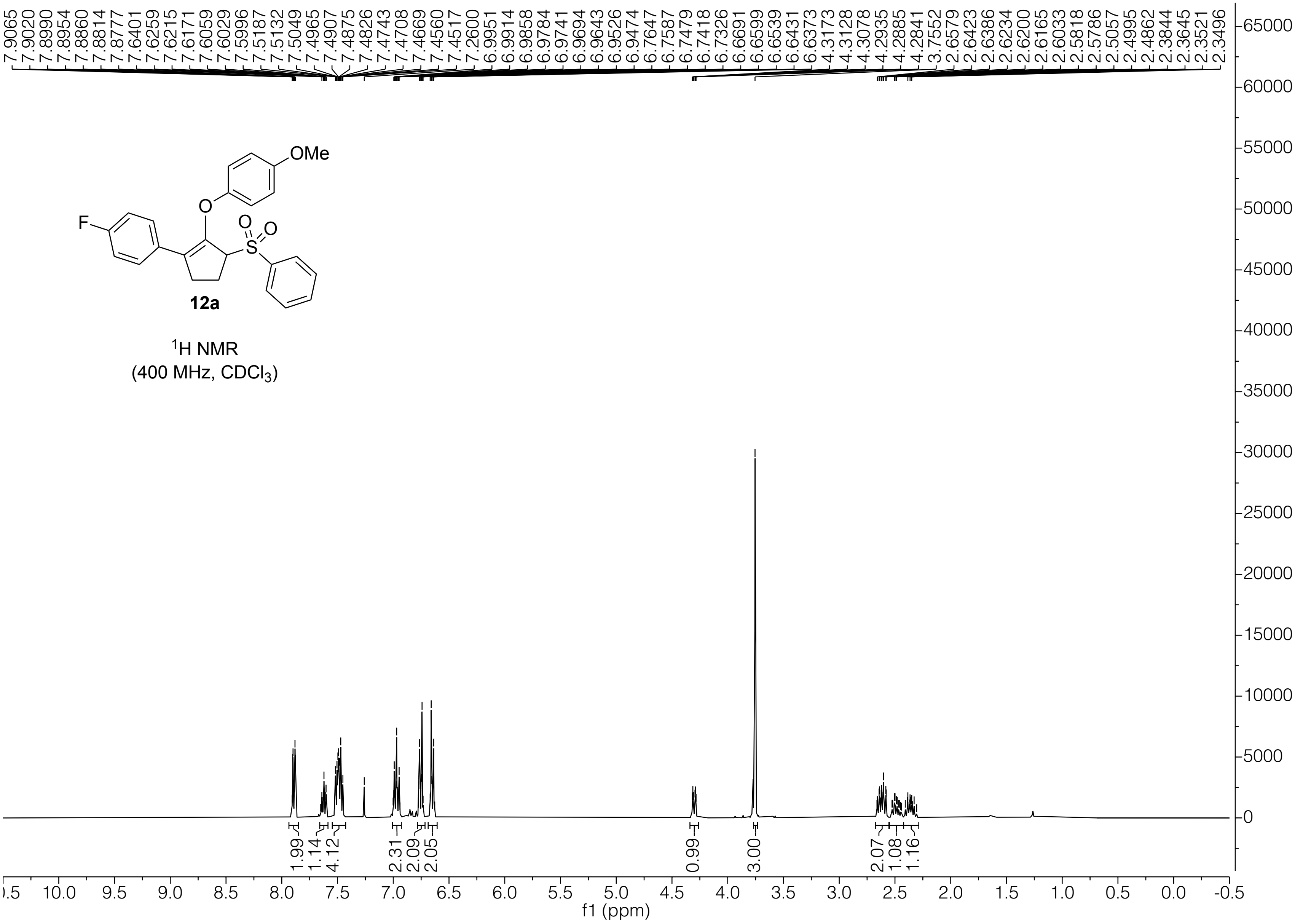
7.9065
7.9020
7.8990
7.8954
7.8860
7.8814
7.8777
7.6401
7.6259
7.6215
7.6171
7.6059
7.6029
7.5996
7.5187
7.5132
7.5049
7.4965
7.4907
7.4875
7.4826
7.4743
7.4708
7.4669
7.4560
7.4517
7.2600
6.9951
6.9914
6.9858
6.9784
6.9741
6.9694
6.9643
6.9526
6.9474
6.7647
6.7587
6.7479
6.7418
6.7326
6.6691
6.6599
6.6539
6.6431
6.6373
4.3173
4.3128
4.3078
4.2935
4.2885
4.2841
3.7552
2.6579
2.6423
2.6386
2.6234
2.6200
2.6165
2.6033
2.5818
2.5786
2.5057
2.4995
2.4862
2.3844
2.3645
2.3521
2.3496
OMe
F
O
S
12a
1H NMR
(400 MHz, CDCl3)
1.99
1.14
4.12
2.31
2.09
2.05
0.99
3.00
2.07
1.08
1.16
f1 (ppm)

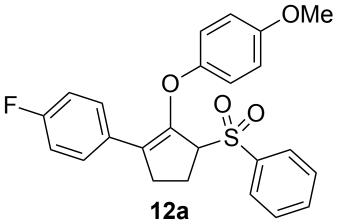

12a

$^{13}C\{^{1}H\}$ NMR
(100 MHz, $CDCl_3$)

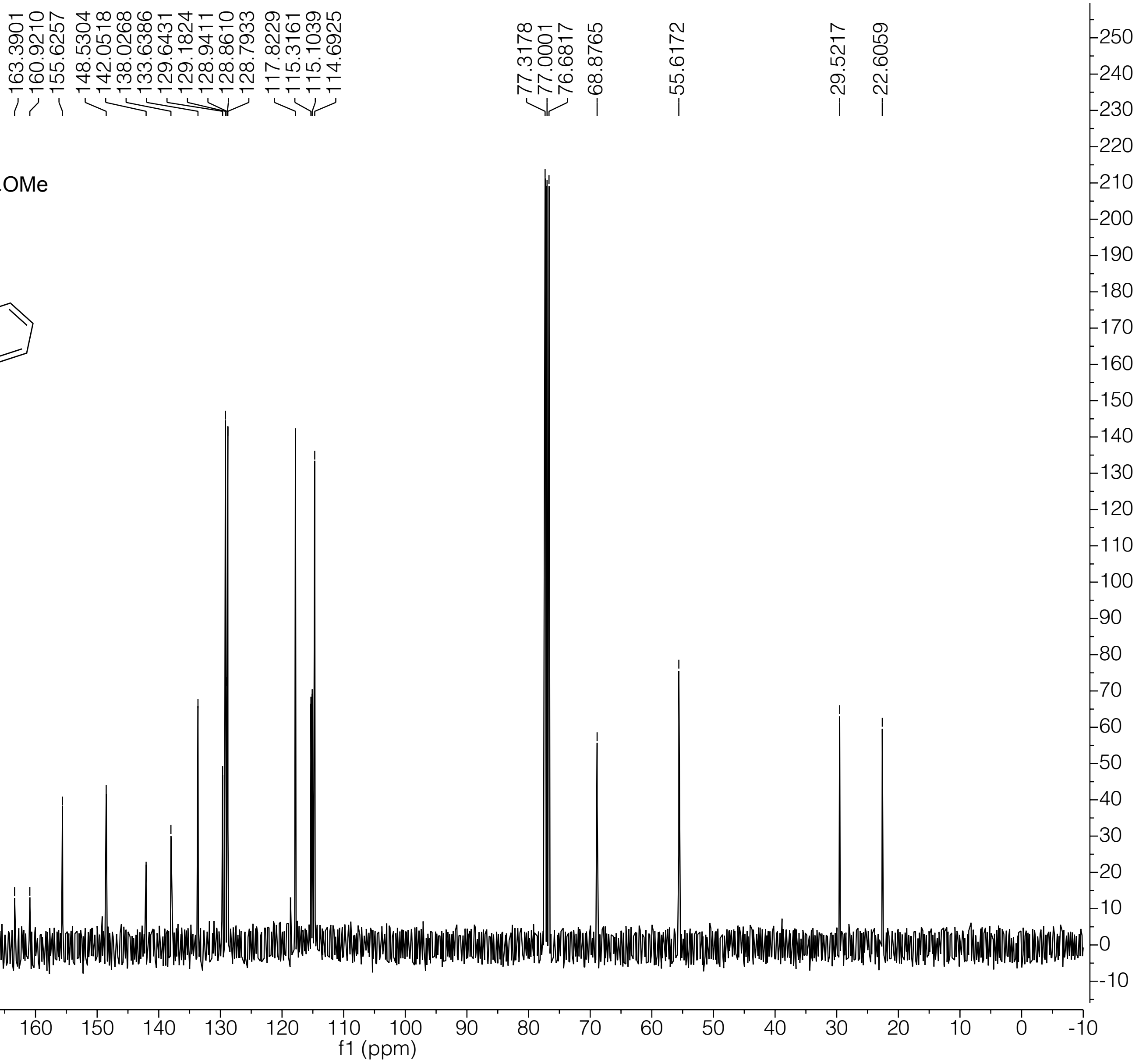

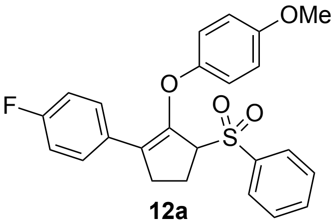

12a

^{19}F NMR
(470 MHz, $CDCl_3$)

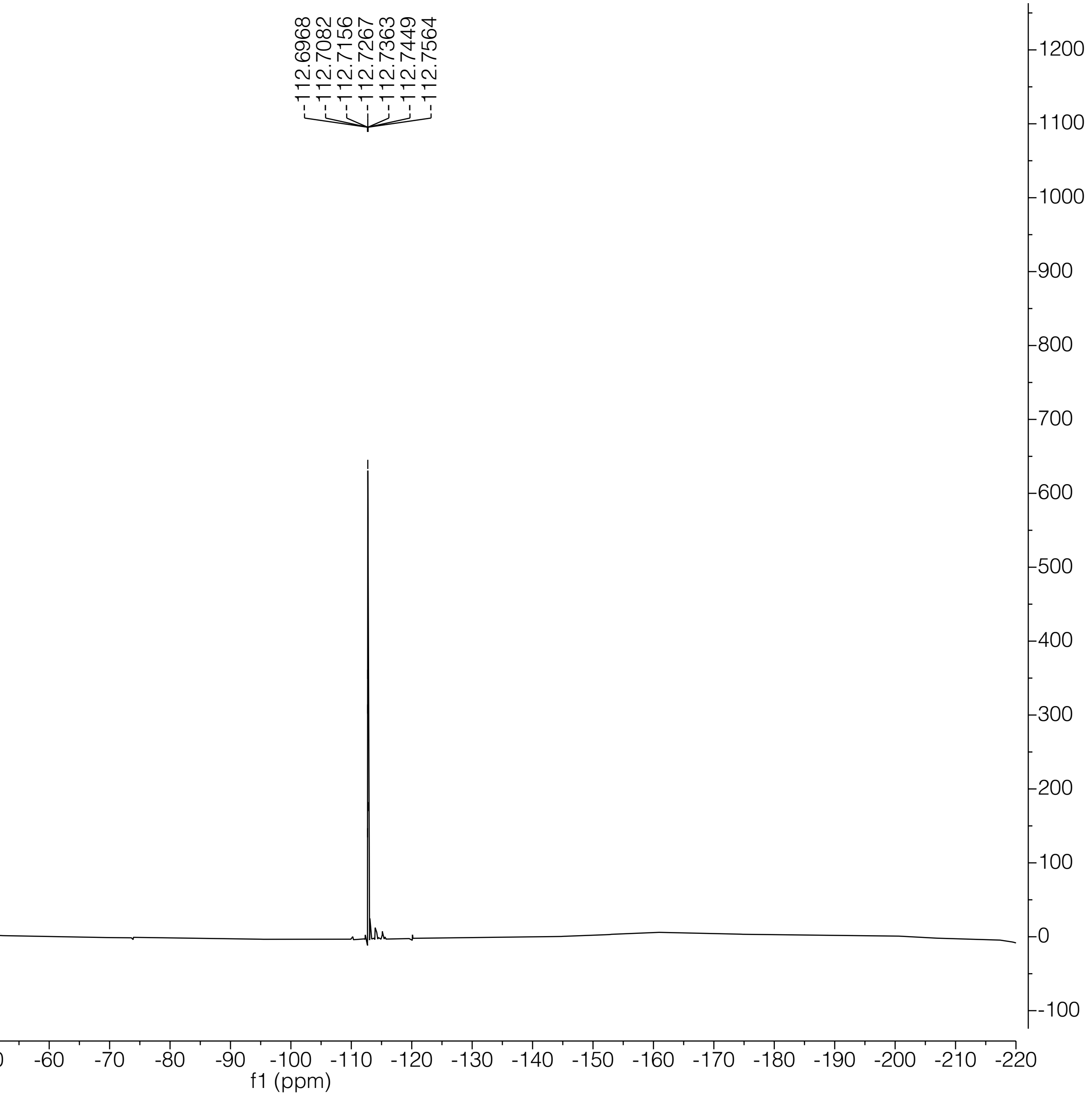

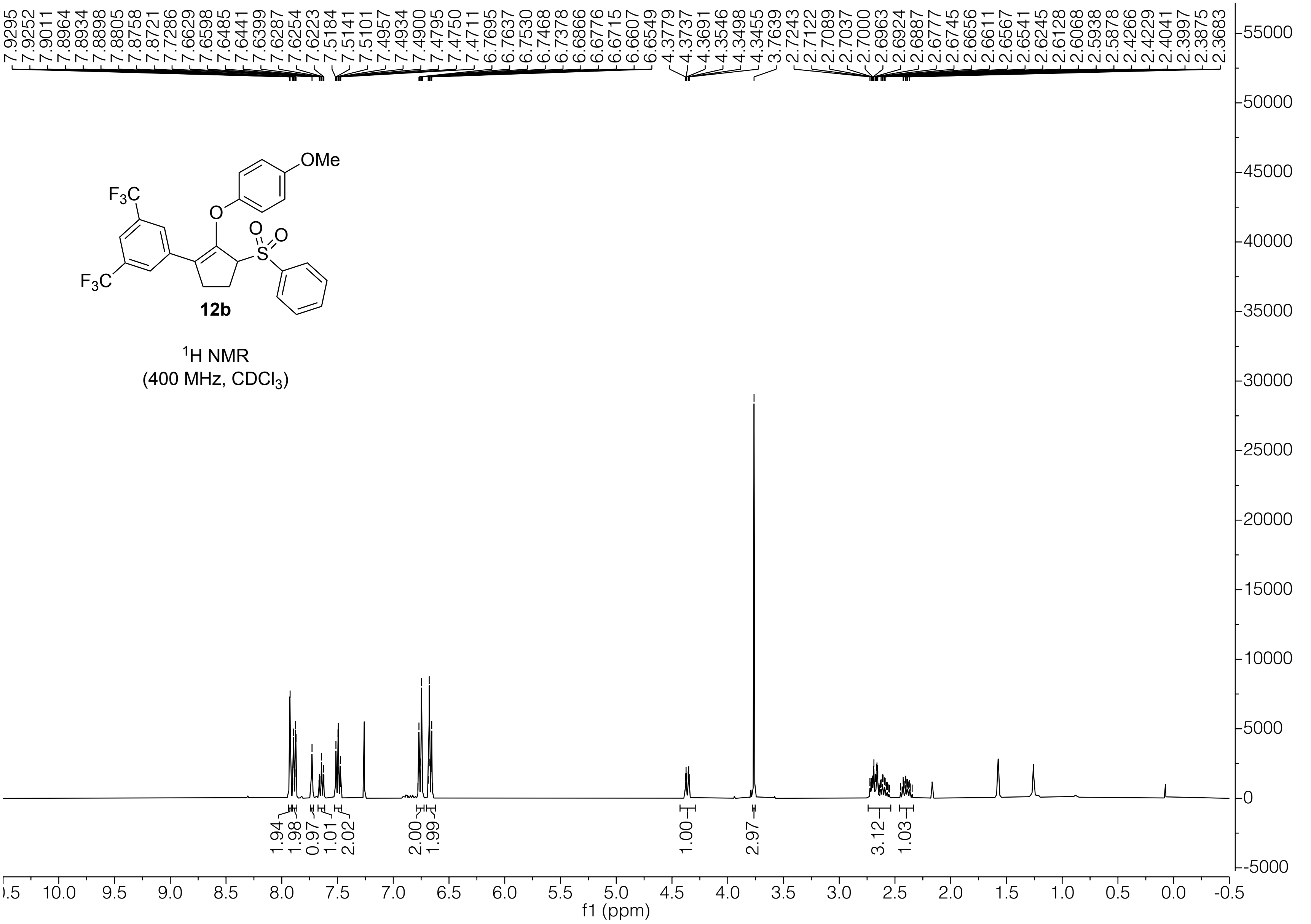

OMe
F_3C
O
O
O
S
F_3C
12b
^{1}H NMR
(400 MHz, $CDCl_3$)
7.9295
7.9252
7.9011
7.8964
7.8934
7.8898
7.8805
7.8758
7.8721
7.7286
7.6629
7.6598
7.6485
7.6441
7.6399
7.6287
7.6254
7.6223
7.5184
7.5141
7.5101
7.4957
7.4934
7.4900
7.4795
7.4750
7.4711
6.7695
6.7637
6.7530
6.7468
6.7378
6.6866
6.6776
6.6715
6.6607
6.6549
4.3779
4.3737
4.3691
4.3546
4.3498
4.3455
3.7639
2.7243
2.7122
2.7089
2.7037
2.7000
2.6963
2.6924
2.6887
2.6777
2.6745
2.6656
2.6611
2.6567
2.6541
2.6245
2.6128
2.6068
2.5938
2.5878
2.4266
2.4229
2.4041
2.3997
2.3875
2.3683
1.94
1.98
0.97
1.01
2.02
2.00
1.99
1.00
2.97
3.12
1.03
f1 (ppm)

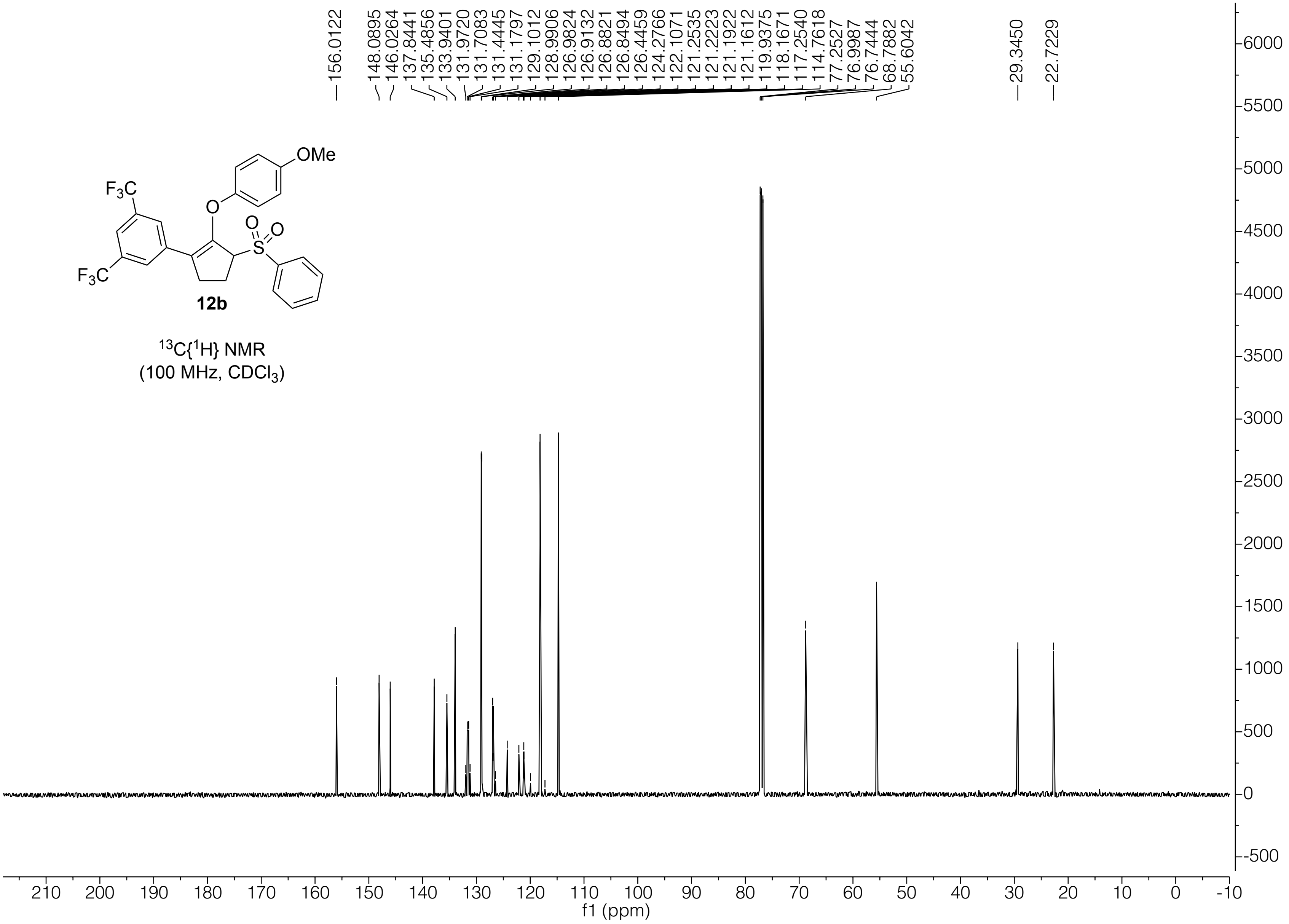
156.0122
148.0895
146.0264
137.8441
135.4856
133.9401
131.9720
131.7083
131.4445
131.1797
129.1012
128.9906
126.9824
126.9132
126.8821
126.8494
126.4459
124.2766
122.1071
121.2535
121.2223
121.1922
121.1612
119.9375
118.1671
117.2540
114.7618
77.2527
76.9987
76.7444
68.7882
55.6042
29.3450
22.7229
OMe
F3C
O
O
S
O
F3C
12b
13C{1H} NMR
(100 MHz, CDCl3)
f1 (ppm)

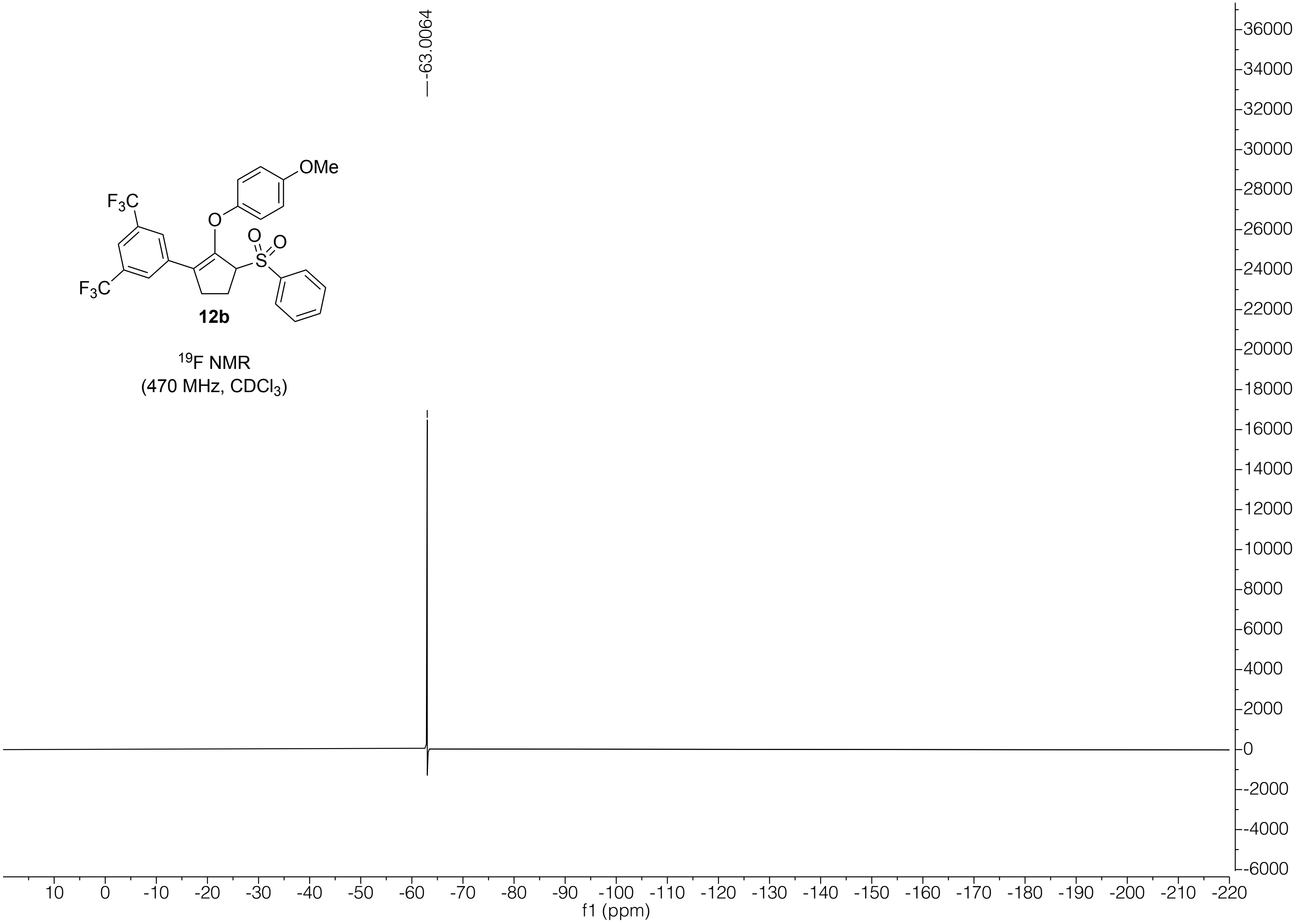

-63.0064
OMe
F_3C
O
O
S
O
F_3C
12b
^{19}F NMR
(470 MHz, $CDCl_3$)
36000
34000
32000
30000
28000
26000
24000
22000
20000
18000
16000
14000
12000
10000
8000
6000
4000
2000
0
-2000
-4000
-6000
10 0 -10 -20 -30 -40 -50 -60 -70 -80 -90 -100 -110 -120 -130 -140 -150 -160 -170 -180 -190 -200 -210 -220
f1 (ppm)

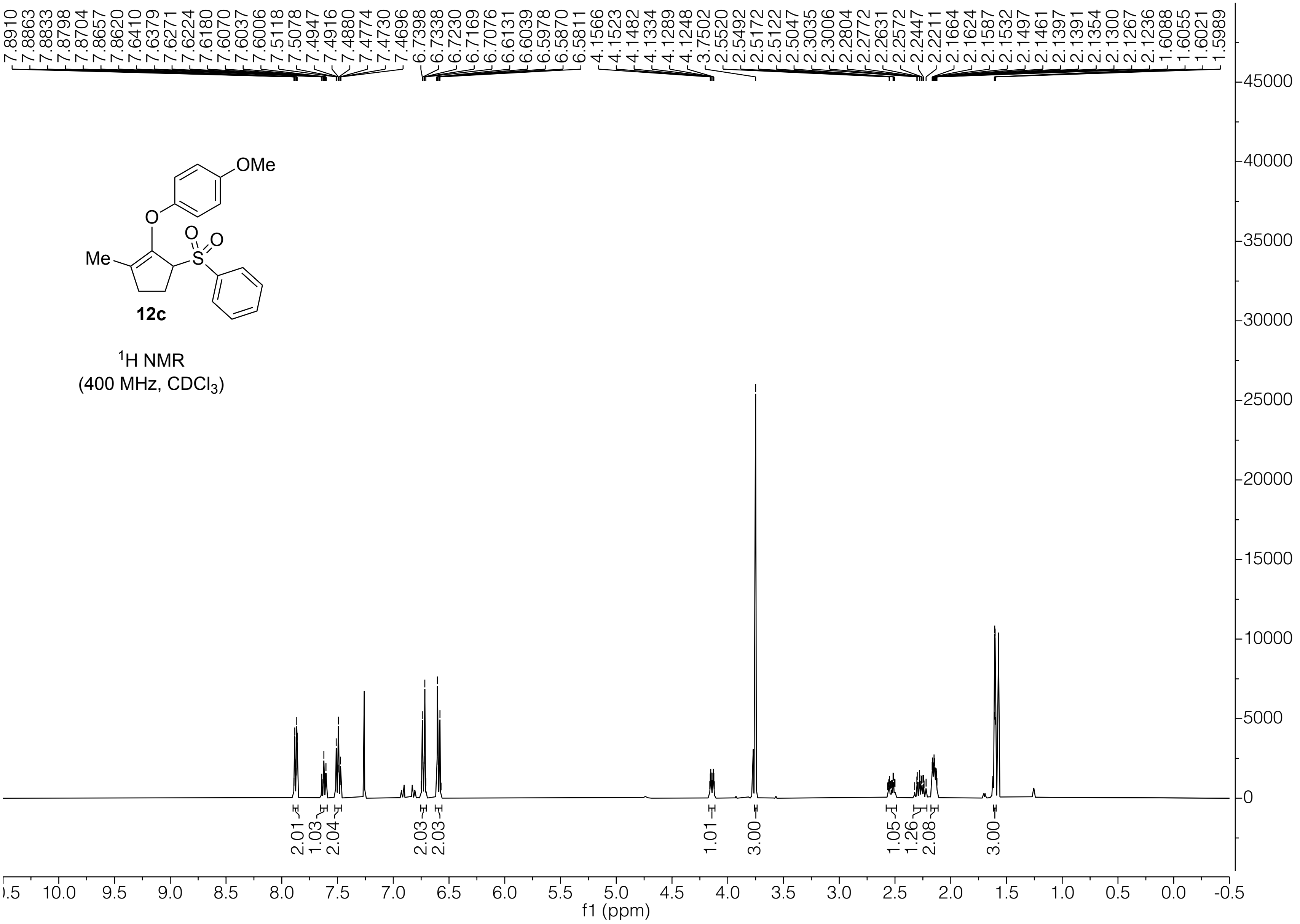

7.8910
7.8863
7.8833
7.8798
7.8704
7.8657
7.8620
7.6410
7.6379
7.6271
7.6224
7.6180
7.6070
7.6037
7.6006
7.5118
7.5078
7.4947
7.4916
7.4880
7.4774
7.4730
7.4696
6.7398
6.7338
6.7230
6.7169
6.7076
6.6131
6.6039
6.5978
6.5870
6.5811
4.1566
4.1523
4.1482
4.1334
4.1289
4.1248
3.7502
2.5520
2.5492
2.5172
2.5122
2.5047
2.3035
2.3006
2.2804
2.2772
2.2631
2.2572
2.2447
2.2211
2.1664
2.1624
2.1587
2.1532
2.1497
2.1461
2.1397
2.1391
2.1354
2.1300
2.1267
2.1236
1.6088
1.6055
1.6021
1.5989
OMe
O
O
S
O
Me
12c
^{1}H NMR
(400 MHz, CDCl$_3$)
2.01
1.03
2.04
2.03
2.03
1.01
3.00
1.05
1.26
2.08
3.00
f1 (ppm)

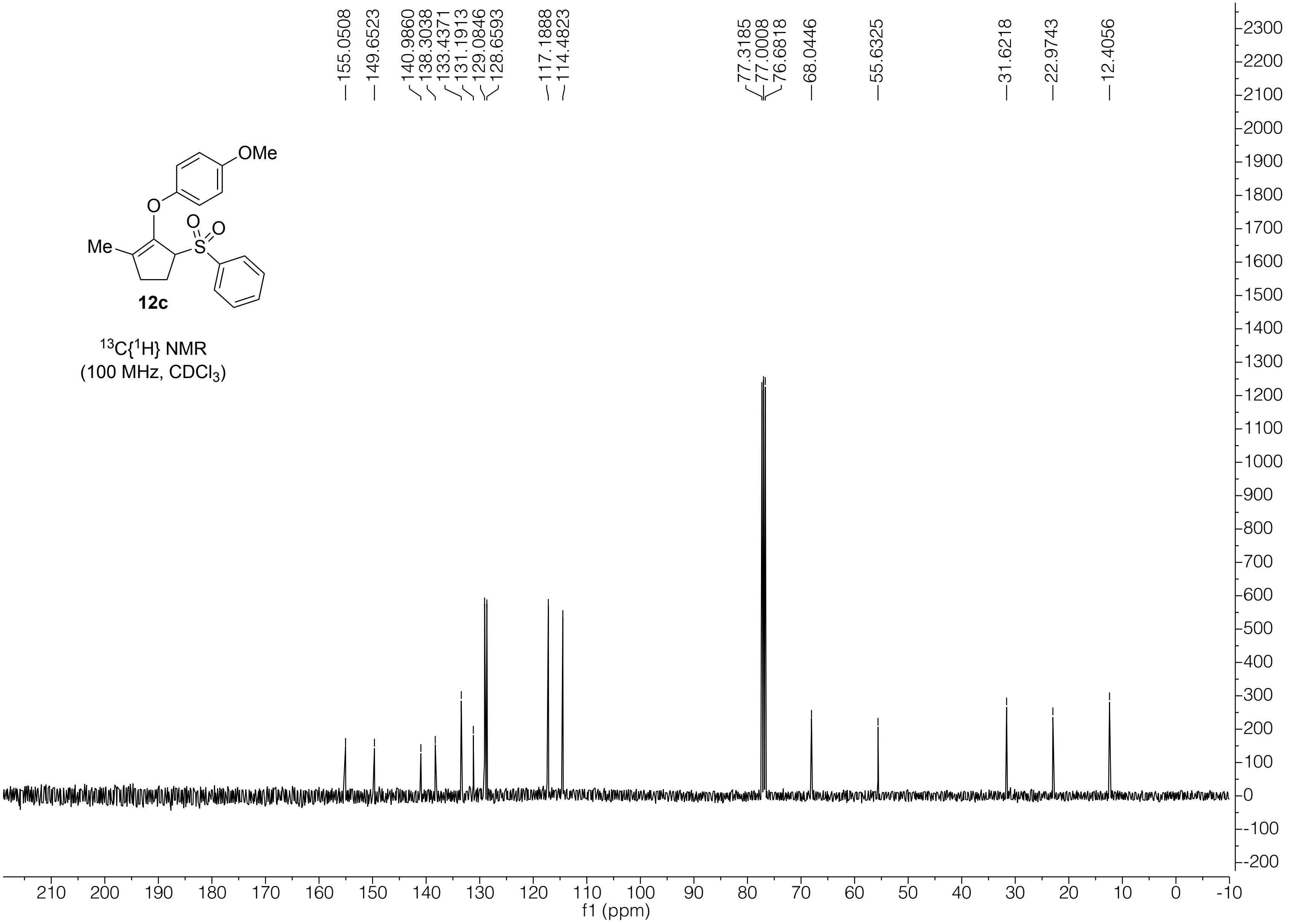
155.0508
149.6523
140.9860
138.3038
133.4371
131.1913
129.0846
128.6593
117.1888
114.4823
77.3185
77.0008
76.6818
68.0446
55.6325
31.6218
22.9743
12.4056
OMe
O
Me
O
O
S
12c
13C{1H} NMR
(100 MHz, CDCl3)
2300
2200
2100
2000
1900
1800
1700
1600
1500
1400
1300
1200
1100
1000
900
800
700
600
500
400
300
200
100
0
-100
-200
210
200
190
180
170
160
150
140
130
120
110
100
90
80
70
60
50
40
30
20
10
0
-10
f1 (ppm)

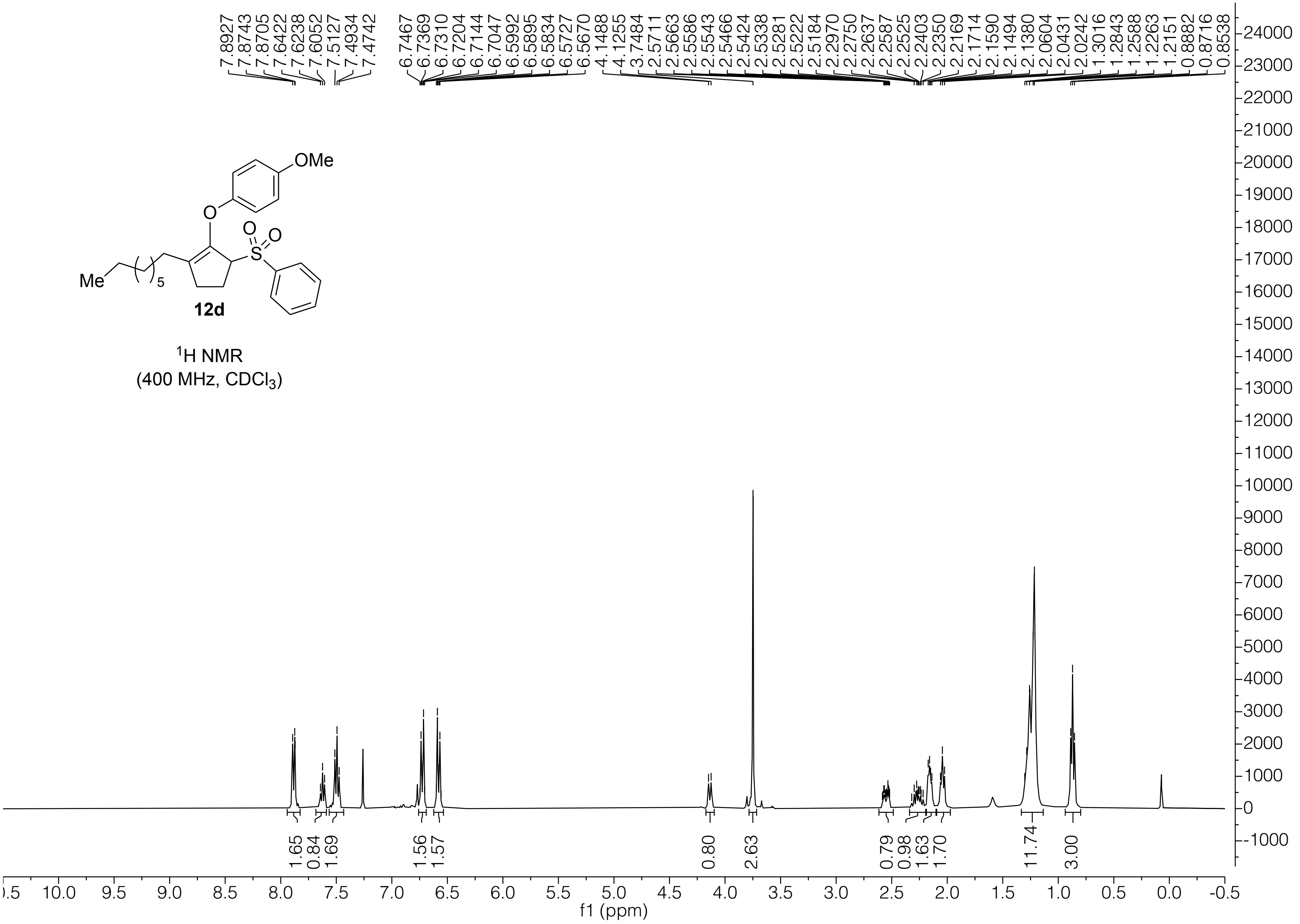
OMe
O
O
S
O
Me
5
12d
1H NMR
(400 MHz, CDCl3)
7.8927 7.8743 7.8705 7.6422 7.6238 7.6052 7.5127 7.4934 7.4742
6.7467 6.7369 6.7310 6.7204 6.7144 6.7047 6.5992 6.5895 6.5834 6.5727 6.5670
4.1488 4.1255 3.7484 2.5711 2.5663 2.5586 2.5543 2.5466 2.5424 2.5338 2.5281 2.5222 2.5184 2.2970 2.2750 2.2637 2.2587 2.2525 2.2403 2.2350 2.2169 2.1714 2.1590 2.1494 2.1380 2.0604 2.0431 2.0242 1.3016 1.2843 1.2588 1.2263 1.2151 0.8882 0.8716 0.8538
1.65 0.84 1.69 1.56 1.57 0.80 2.63 0.79 0.98 1.63 1.70 11.74 3.00
f1 (ppm)

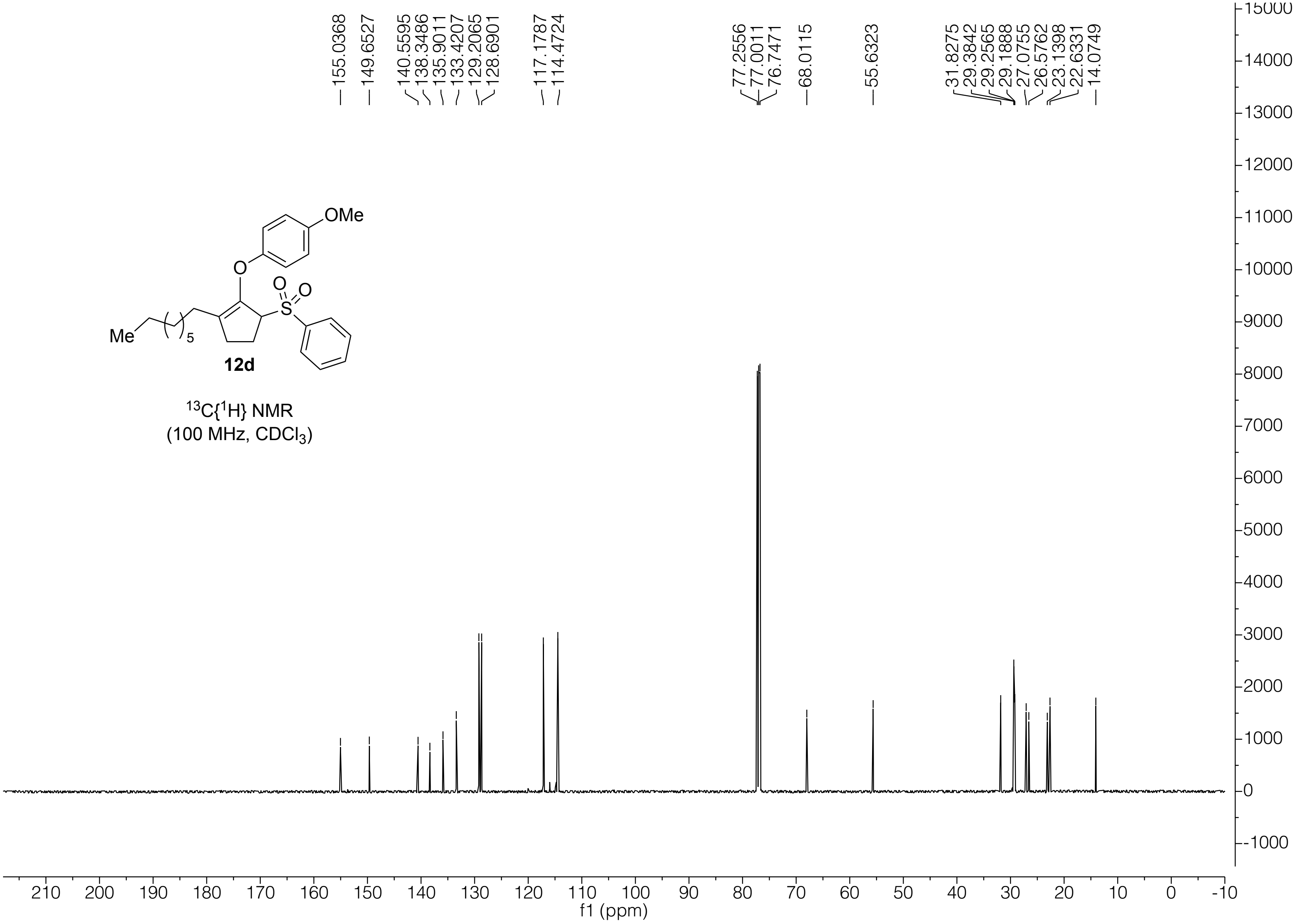

$^{13}C\{^{1}H\}$ NMR
(100 MHz, $CDCl_3$)

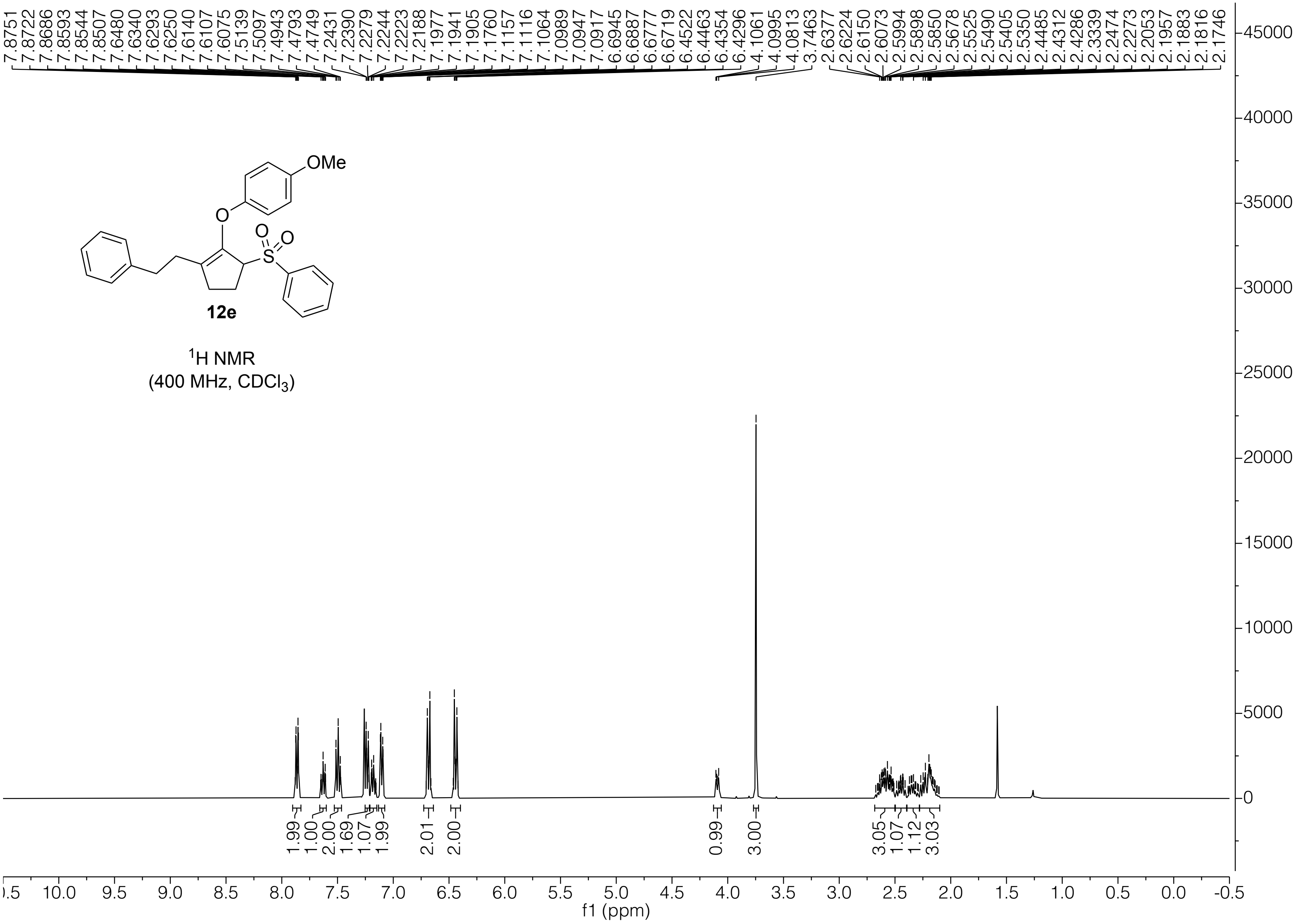
OMe
12e
1H NMR
(400 MHz, CDCl3)
7.8751 7.8722 7.8686 7.8593 7.8544 7.8507 7.6480 7.6340 7.6293 7.6250 7.6140 7.6107 7.6075 7.5139 7.5097 7.4943 7.4793 7.4749 7.2431 7.2390 7.2279 7.2244 7.2223 7.2188 7.1977 7.1941 7.1905 7.1760 7.1157 7.1116 7.1064 7.0989 7.0947 7.0917 6.6945 6.6887 6.6777 6.6719 6.4522 6.4463 6.4354 6.4296 4.1061 4.0995 4.0813 3.7463 2.6377 2.6224 2.6150 2.6073 2.5994 2.5898 2.5850 2.5678 2.5525 2.5490 2.5405 2.5350 2.4485 2.4312 2.4286 2.3339 2.2474 2.2273 2.2053 2.1957 2.1883 2.1816 2.1746
1.99 1.00 2.00 1.69 1.07 1.99 2.01 2.00 0.99 3.00 3.05 1.07 1.12 3.03
f1 (ppm)

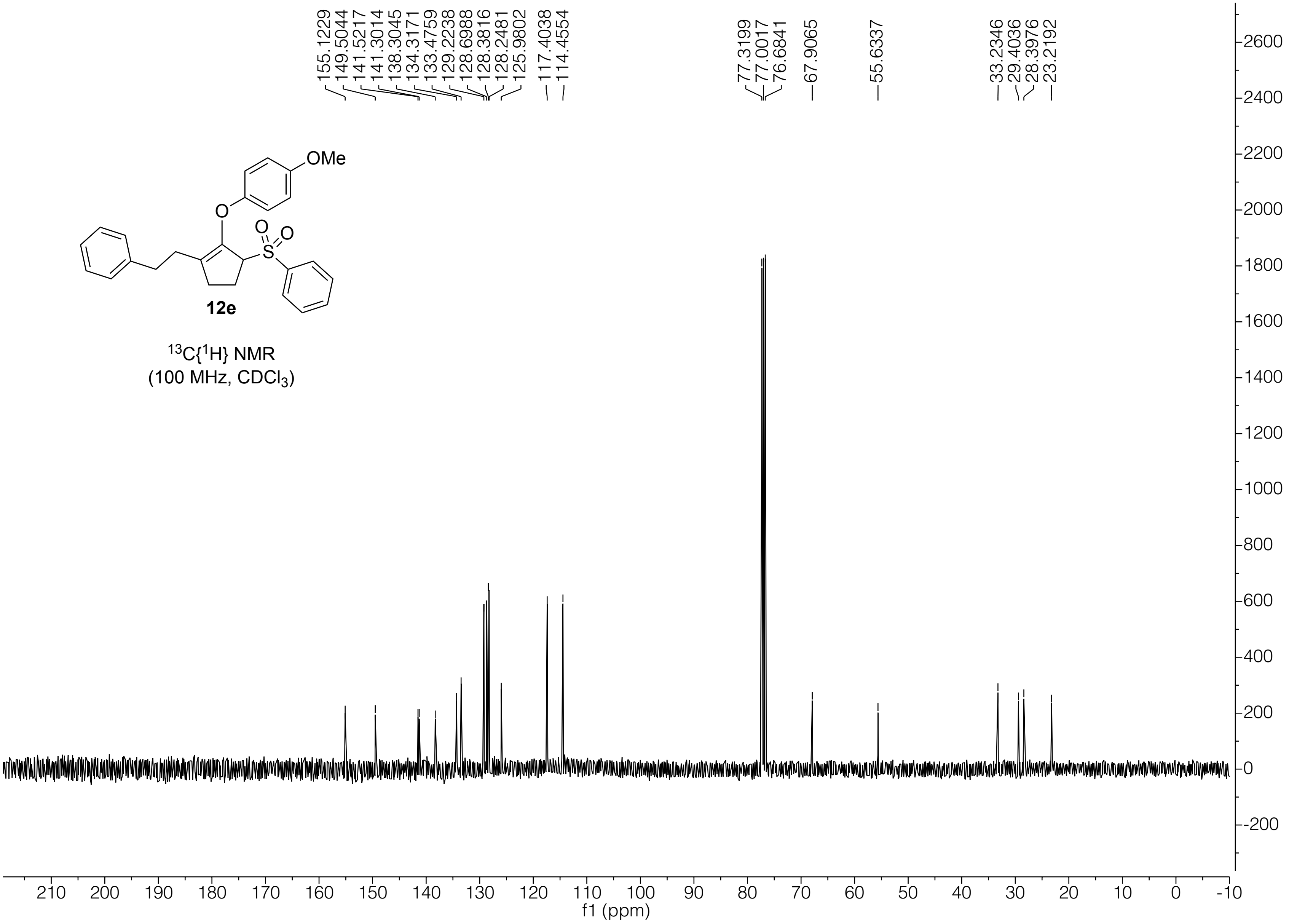
155.1229
149.5044
141.5217
141.3014
138.3045
134.3171
133.4759
129.2238
128.6988
128.3816
128.2481
125.9802
117.4038
114.4554
77.3199
77.0017
76.6841
67.9065
55.6337
33.2346
29.4036
28.3976
23.2192
OMe
O
O
S
O
12e
13C{1H} NMR
(100 MHz, CDCl3)
f1 (ppm)

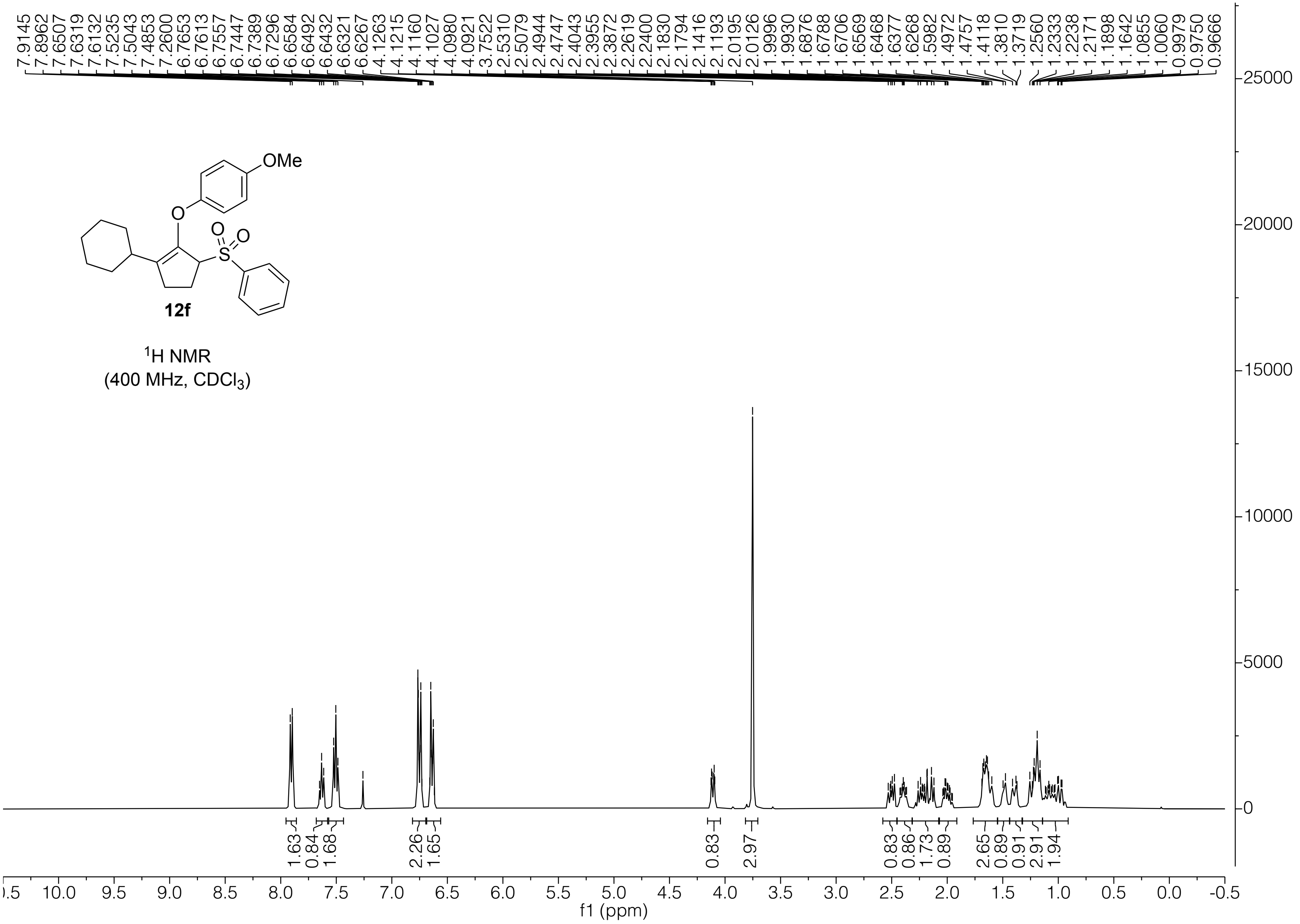

OMe
O
S
O
O
12f
^{1}H NMR
(400 MHz, CDCl3)
f1 (ppm)

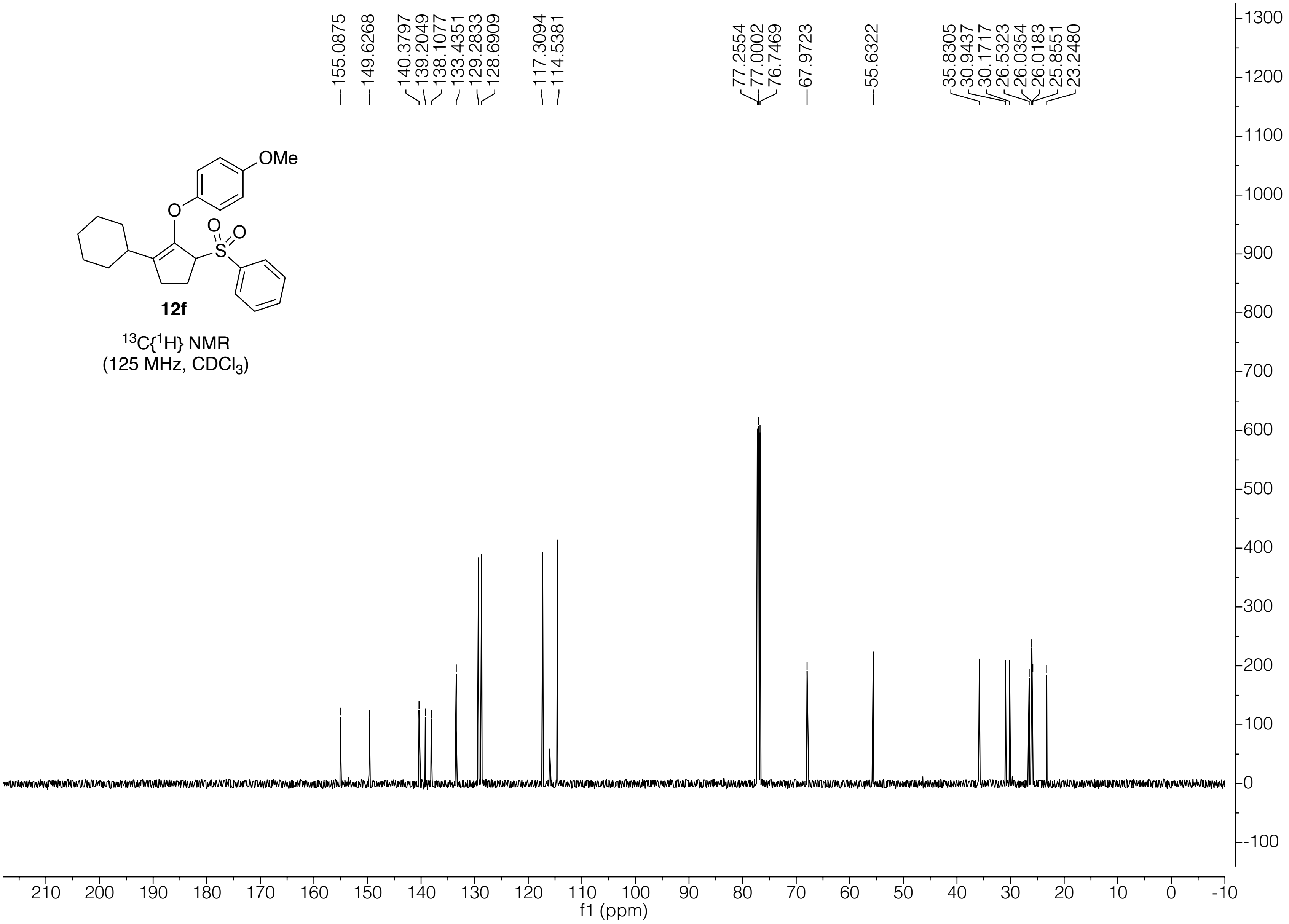
OMe
O
O
S
O
12f
$^{13}C\{^1H\}$ NMR
(125 MHz, $CDCl_3$)
155.0875
149.6268
140.3797
139.2049
138.1077
133.4351
129.2833
128.6909
117.3094
114.5381
77.2554
77.0002
76.7469
67.9723
55.6322
35.8305
30.9437
30.1717
26.5323
26.0354
26.0183
25.8551
23.2480
f1 (ppm)

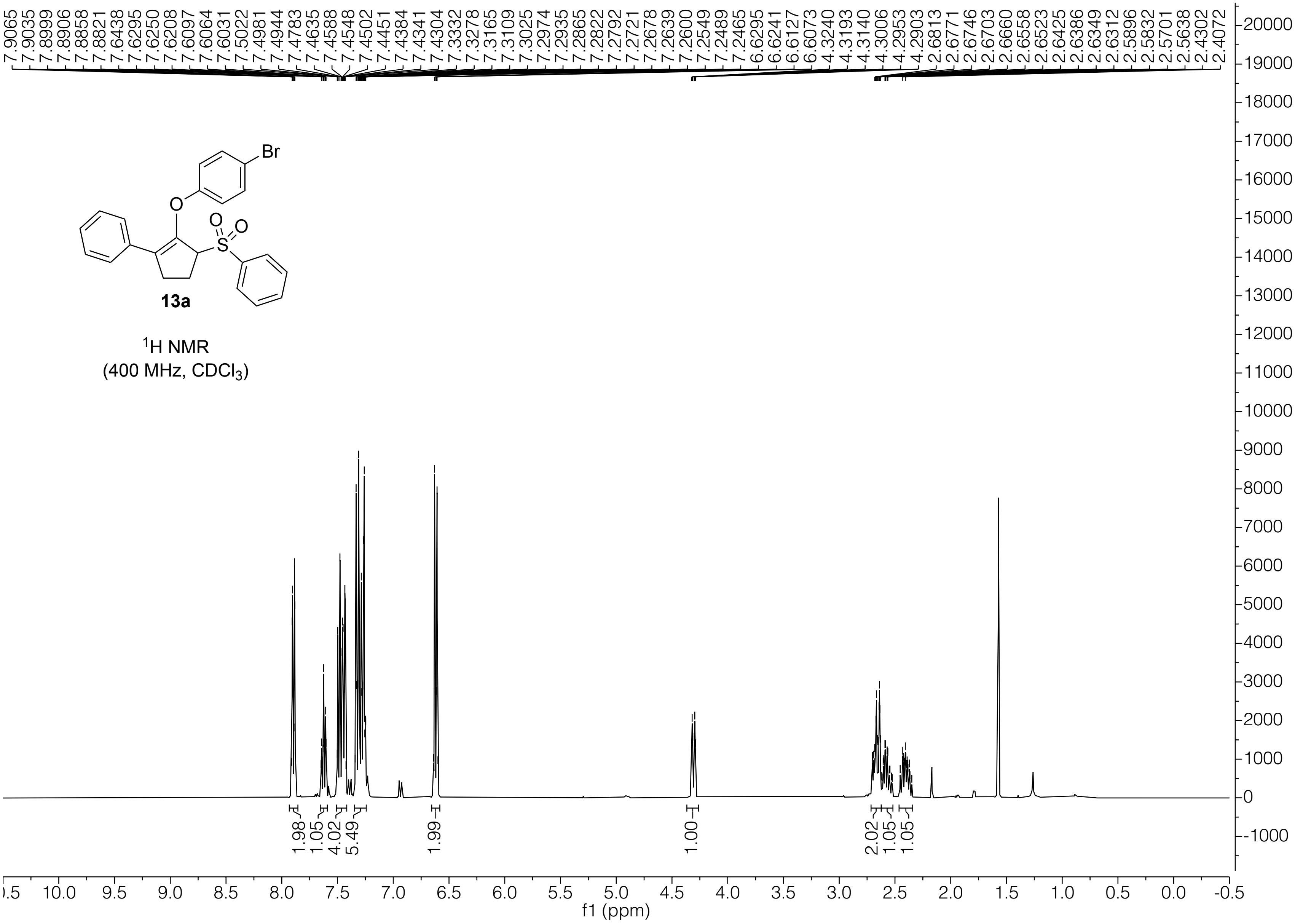
7.9065
7.9035
7.8999
7.8906
7.8858
7.8821
7.6438
7.6295
7.6250
7.6208
7.6097
7.6064
7.6031
7.5022
7.4981
7.4944
7.4783
7.4635
7.4588
7.4548
7.4502
7.4451
7.4384
7.4341
7.4304
7.3332
7.3278
7.3165
7.3109
7.3025
7.2974
7.2935
7.2865
7.2822
7.2792
7.2721
7.2678
7.2639
7.2600
7.2549
7.2489
7.2465
6.6295
6.6241
6.6127
6.6073
4.3240
4.3193
4.3140
4.3006
4.2953
4.2903
2.6813
2.6771
2.6746
2.6703
2.6660
2.6558
2.6523
2.6425
2.6386
2.6349
2.6312
2.5896
2.5832
2.5701
2.5638
2.4302
2.4072
Br
O
O
S
O
13a
1H NMR
(400 MHz, CDCl3)
1.98
1.05
4.02
5.49
1.99
1.00
2.02
1.05
1.05
f1 (ppm)

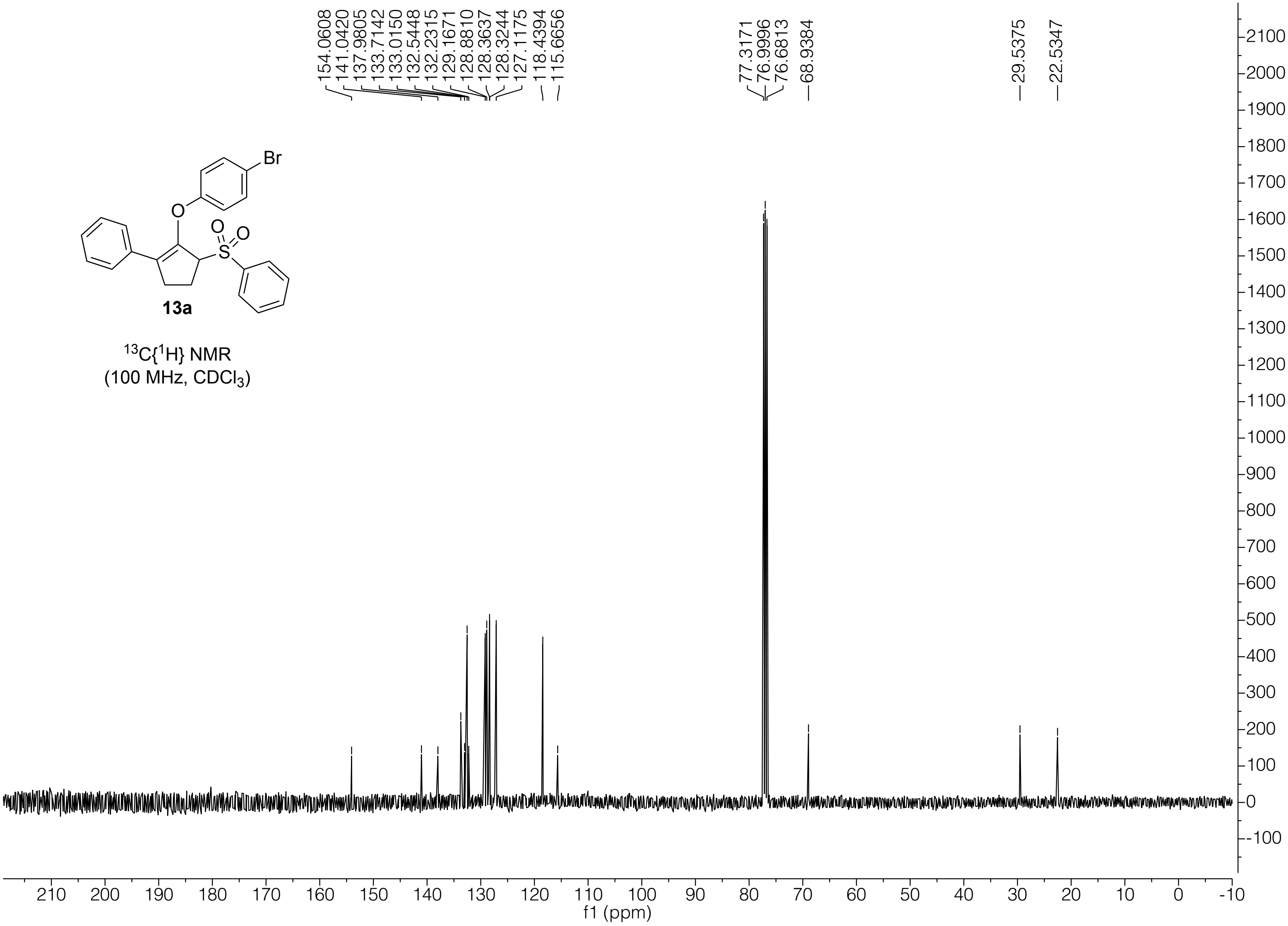

154.0608
141.0420
137.9805
133.7142
133.0150
132.5448
132.2315
129.1671
128.8810
128.3637
128.3244
127.1175
118.4394
115.6656
77.3171
76.9996
76.6813
68.9384
29.5375
22.5347
Br
O
S
13a
¹³C{¹H} NMR
(100 MHz, CDCl₃)
f1 (ppm)

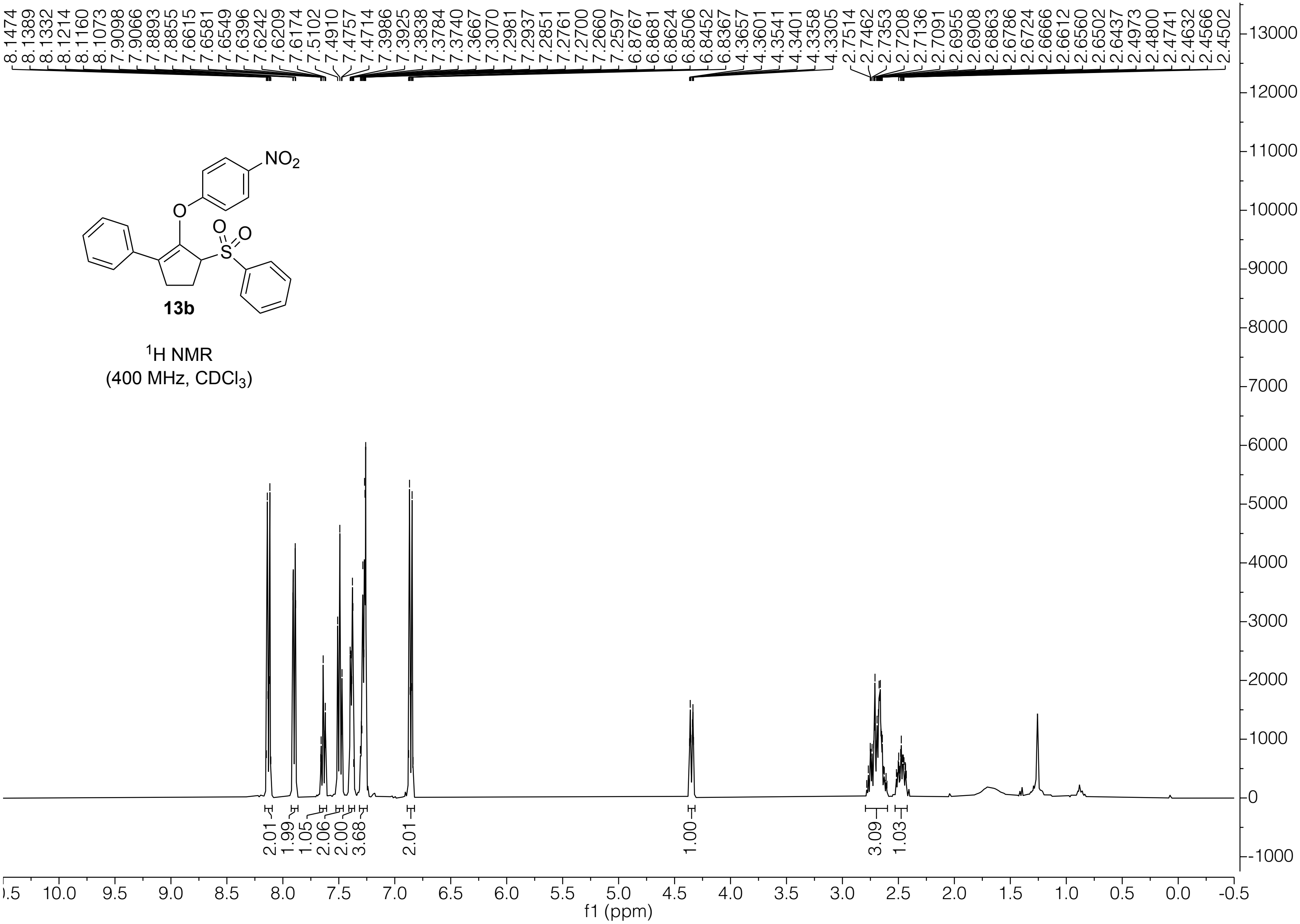
8.1474 8.1389 8.1332 8.1214 8.1160 8.1073 7.9098 7.9066 7.8893 7.8855 7.6615 7.6581 7.6549 7.6396 7.6242 7.6209 7.6174 7.5102 7.4910 7.4757 7.4714 7.3986 7.3925 7.3838 7.3784 7.3740 7.3667 7.3070 7.2981 7.2937 7.2851 7.2761 7.2700 7.2660 7.2597 6.8767 6.8681 6.8624 6.8506 6.8452 6.8367 4.3657 4.3601 4.3541 4.3401 4.3358 4.3305 2.7514 2.7462 2.7353 2.7208 2.7136 2.7091 2.6955 2.6908 2.6863 2.6786 2.6724 2.6666 2.6612 2.6560 2.6502 2.6437 2.4973 2.4800 2.4741 2.4632 2.4566 2.4502
NO2
O
O
S
O
13b
1H NMR
(400 MHz, CDCl3)
2.01
1.99
1.05
2.06
2.00
3.68
2.01
1.00
3.09
1.03
f1 (ppm)

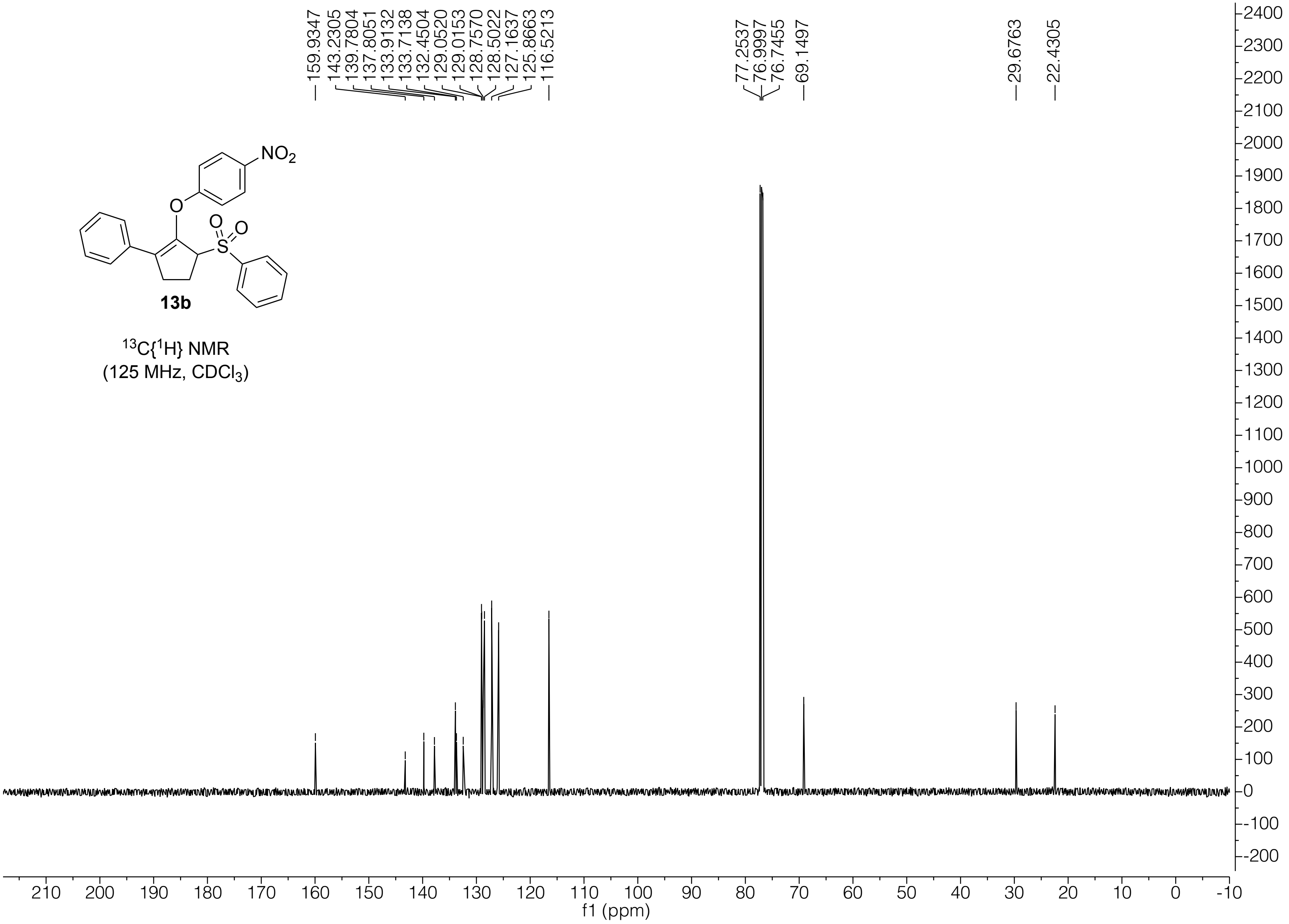
159.9347
143.2305
139.7804
137.8051
133.9132
133.7138
132.4504
129.0520
129.0153
128.7570
128.5022
127.1637
125.8663
116.5213
77.2537
76.9997
76.7455
69.1497
29.6763
22.4305
NO2
O
O
S
O
13b
13C{1H} NMR
(125 MHz, CDCl3)
f1 (ppm)

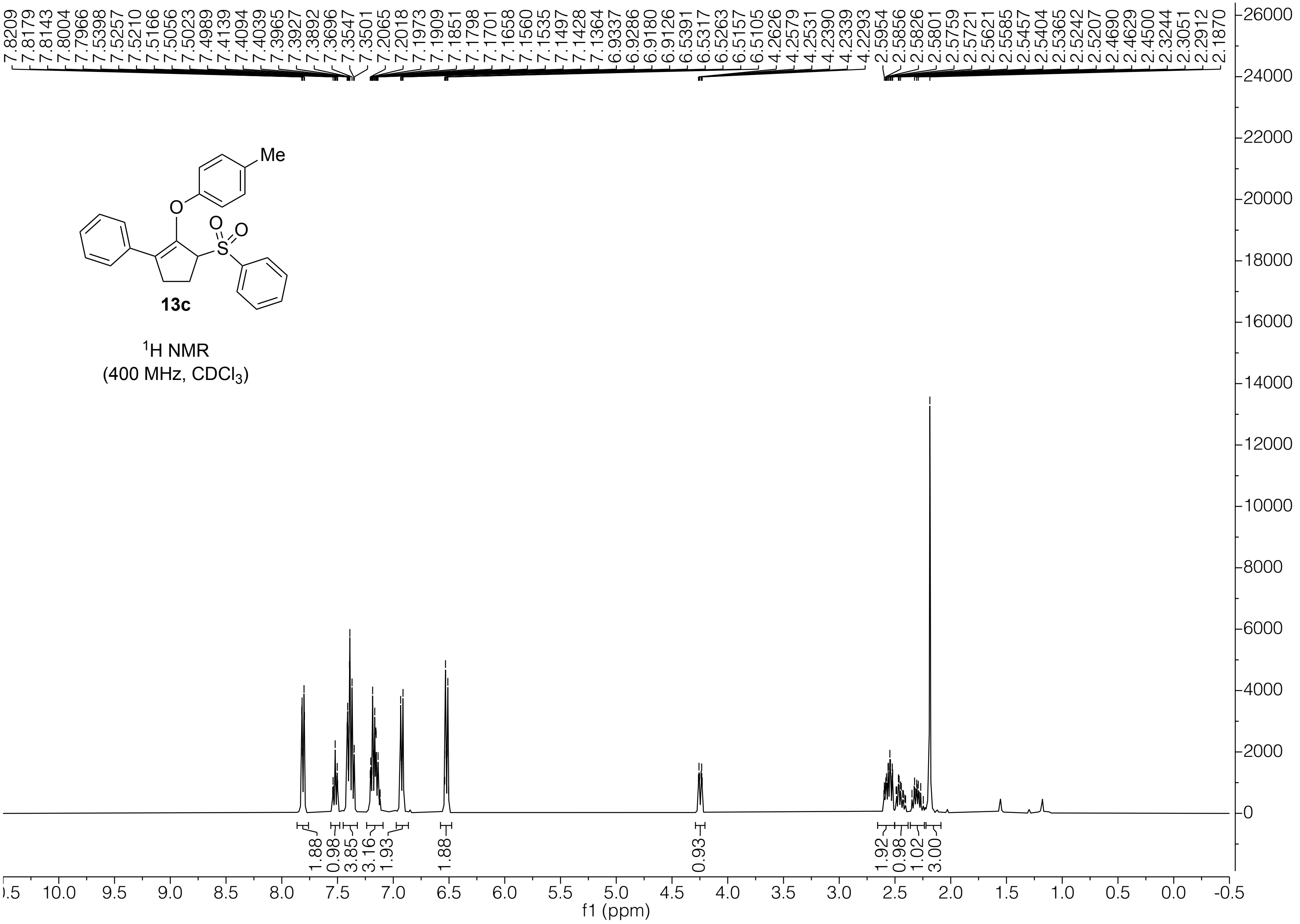
7.8209 7.8179 7.8143 7.8004 7.7966 7.5398 7.5257 7.5210 7.5166 7.5056 7.5023 7.4989 7.4139 7.4094 7.4039 7.3965 7.3927 7.3892 7.3696 7.3547 7.3501 7.2065 7.2018 7.1973 7.1909 7.1851 7.1798 7.1701 7.1658 7.1560 7.1535 7.1497 7.1428 7.1364 6.9337 6.9286 6.9180 6.9126 6.5391 6.5317 6.5263 6.5157 6.5105 4.2626 4.2579 4.2531 4.2390 4.2339 4.2293 2.5954 2.5856 2.5826 2.5801 2.5759 2.5721 2.5621 2.5585 2.5457 2.5404 2.5365 2.5242 2.5207 2.4690 2.4629 2.4500 2.3244 2.3051 2.2912 2.1870
Me
O
O
S
O
13c
1H NMR
(400 MHz, CDCl3)
1.88 0.98 3.85 3.16 1.93 1.88 0.93 1.92 0.98 1.02 3.00
f1 (ppm)

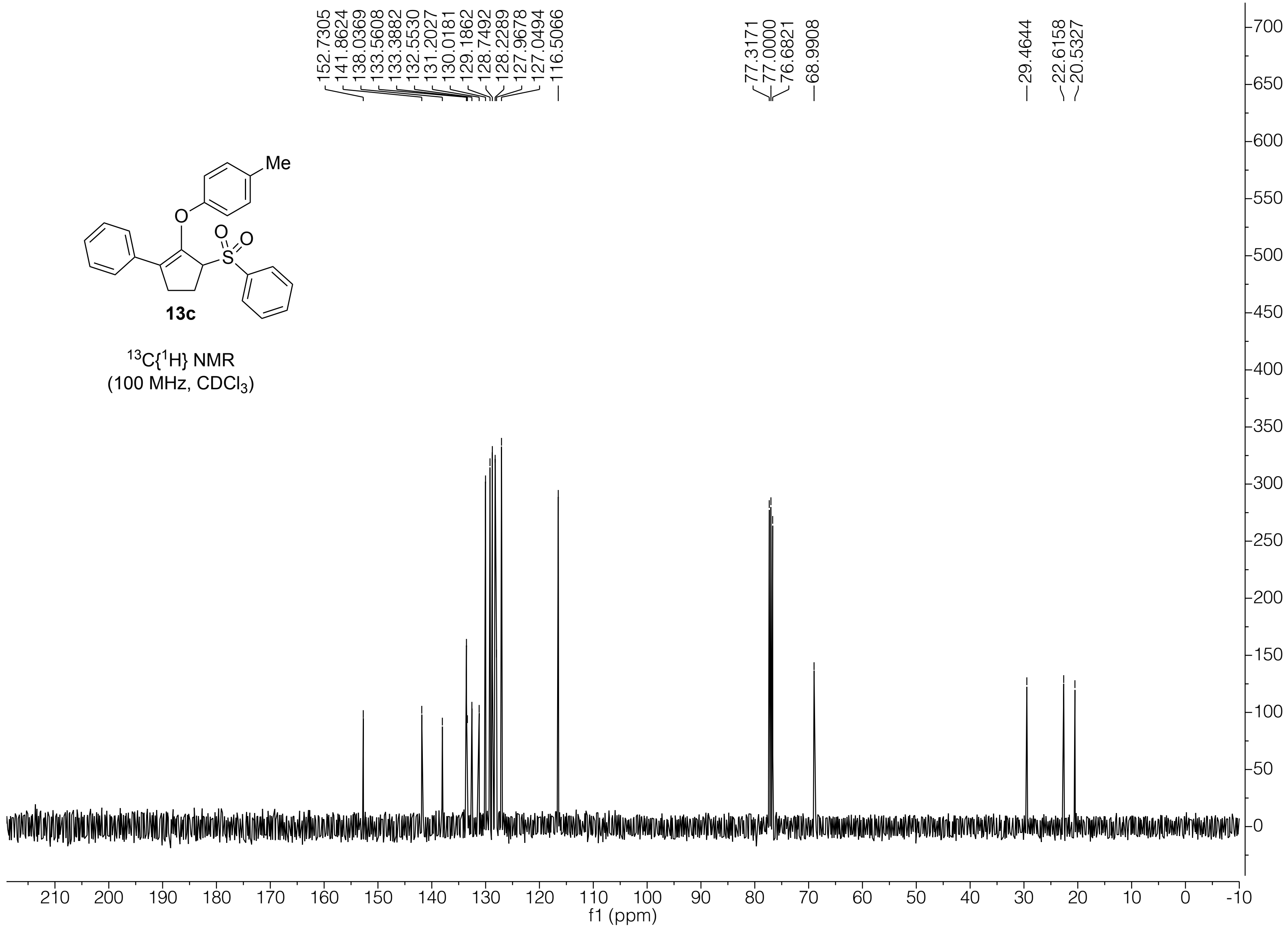
152.7305
141.8624
138.0369
133.5608
133.3882
132.5530
131.2027
130.0181
129.1862
128.7492
128.2289
127.9678
127.0494
116.5066
77.3171
77.0000
76.6821
68.9908
29.4644
22.6158
20.5327
Me
O
S
O
O
13c
$^{13}C\{^{1}H\}$ NMR
(100 MHz, $CDCl_3$)
f1 (ppm)

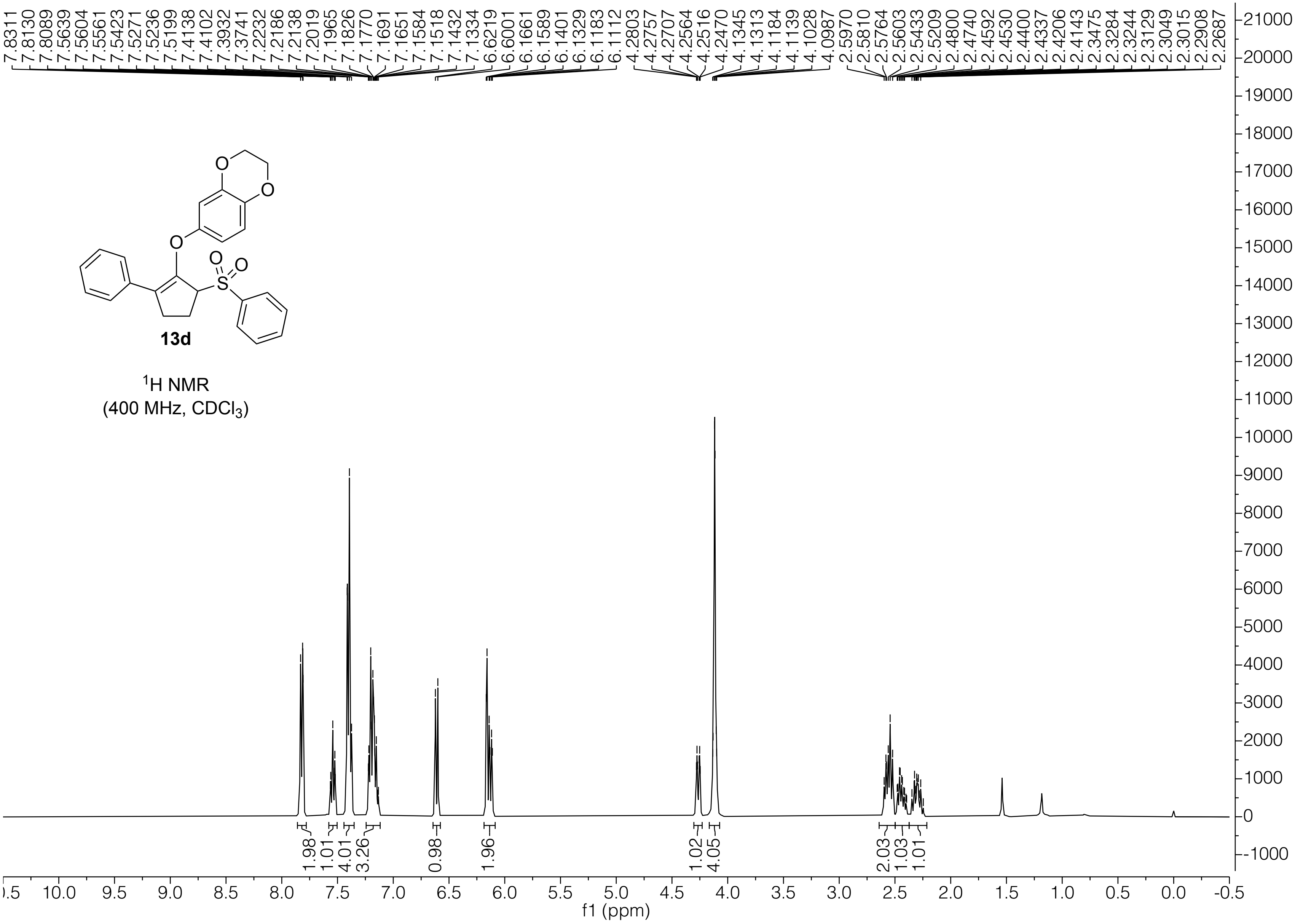
7.8311 7.8130 7.8089 7.5639 7.5604 7.5561 7.5423 7.5271 7.5236 7.5199 7.4138 7.4102 7.3932 7.3741 7.2232 7.2186 7.2138 7.2019 7.1965 7.1826 7.1770 7.1691 7.1651 7.1584 7.1518 7.1432 7.1334 6.6219 6.6001 6.1661 6.1589 6.1401 6.1329 6.1183 6.1112 4.2803 4.2757 4.2707 4.2564 4.2516 4.2470 4.1345 4.1313 4.1184 4.1139 4.1028 4.0987 2.5970 2.5810 2.5764 2.5603 2.5433 2.5209 2.4800 2.4740 2.4592 2.4530 2.4400 2.4337 2.4206 2.4143 2.3475 2.3284 2.3244 2.3129 2.3049 2.3015 2.2908 2.2687
13d
1H NMR
(400 MHz, CDCl3)
1.98 1.01 4.01 3.26 0.98 1.96 1.02 4.05 2.03 1.03 1.01
f1 (ppm)

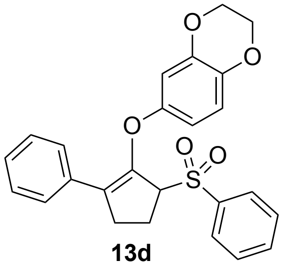

13d

$^{13}C\{^{1}H\}$ NMR
(100 MHz, $CDCl_3$)

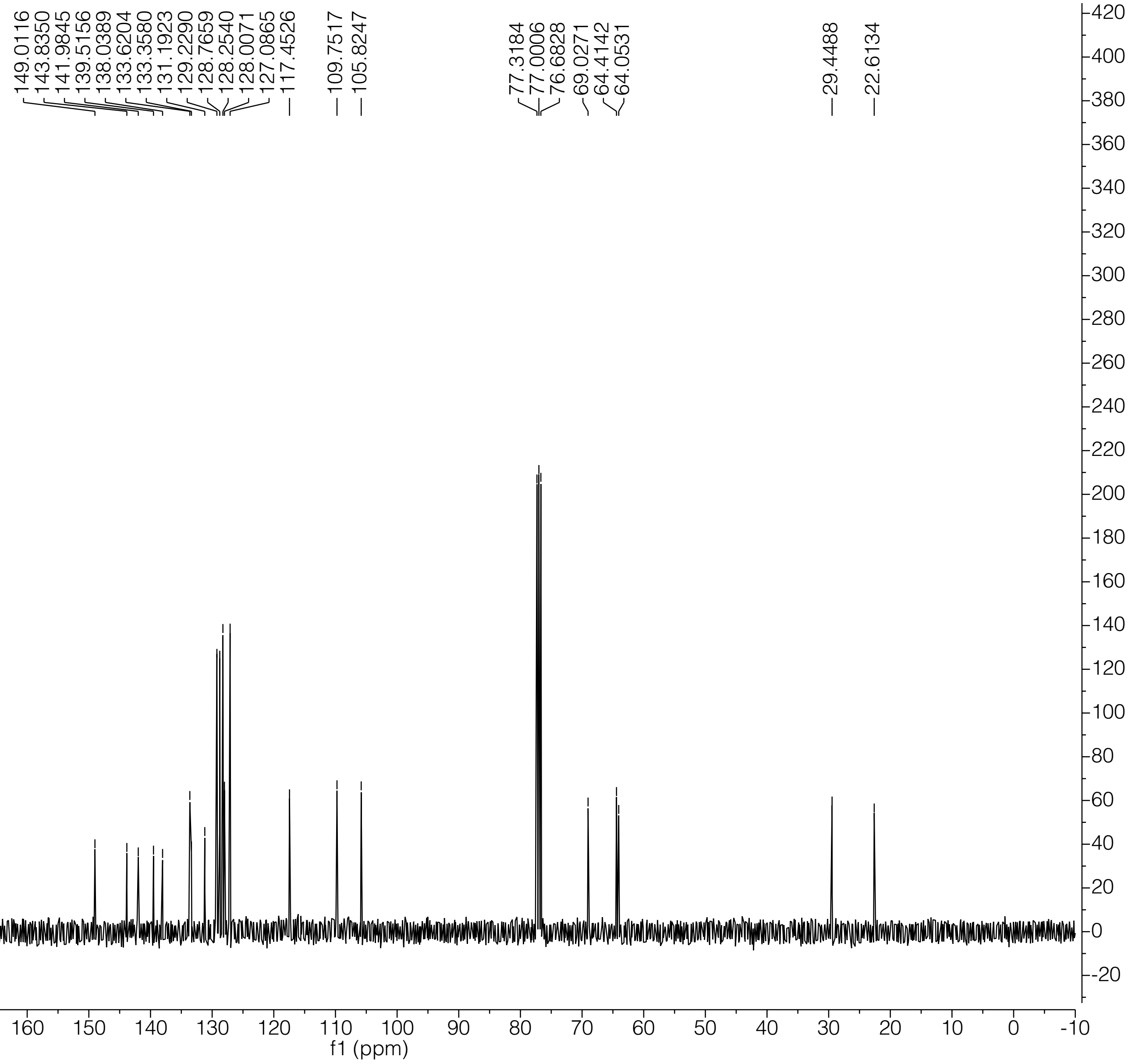

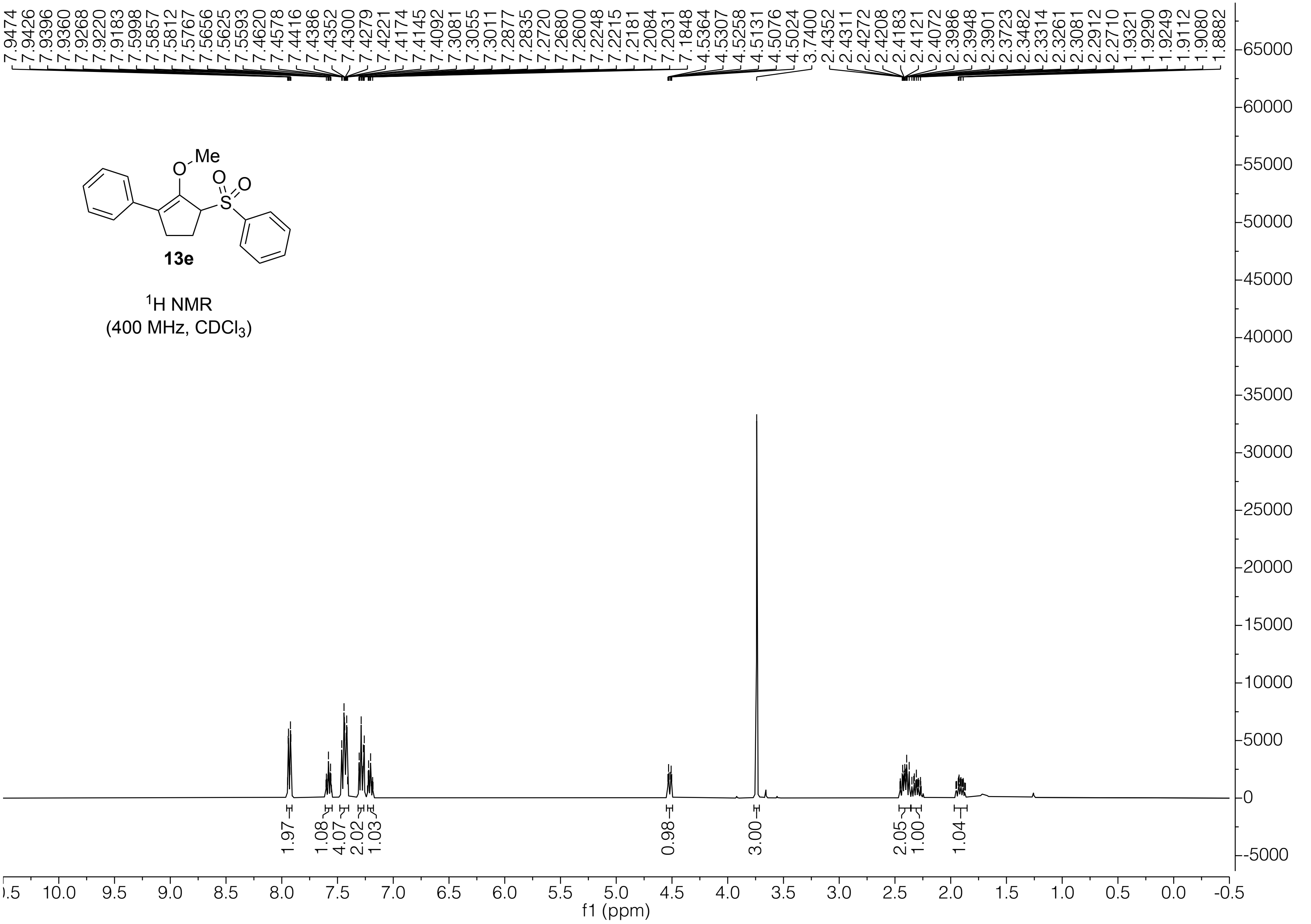
7.9474
7.9426
7.9396
7.9360
7.9268
7.9220
7.9183
7.5998
7.5857
7.5812
7.5767
7.5656
7.5625
7.5593
7.4620
7.4578
7.4416
7.4386
7.4352
7.4300
7.4279
7.4221
7.4174
7.4145
7.4092
7.3081
7.3055
7.3011
7.2877
7.2835
7.2720
7.2680
7.2600
7.2248
7.2215
7.2181
7.2084
7.2031
7.1848
4.5364
4.5307
4.5258
4.5131
4.5076
4.5024
3.7400
2.4352
2.4311
2.4272
2.4208
2.4183
2.4121
2.4072
2.3986
2.3948
2.3901
2.3723
2.3482
2.3314
2.3261
2.3081
2.2912
2.2710
1.9321
1.9290
1.9249
1.9112
1.9080
1.8882
Me
O
O
S
O
13e
1H NMR
(400 MHz, CDCl3)
1.97
1.08
4.07
2.02
1.03
0.98
3.00
2.05
1.00
1.04
f1 (ppm)

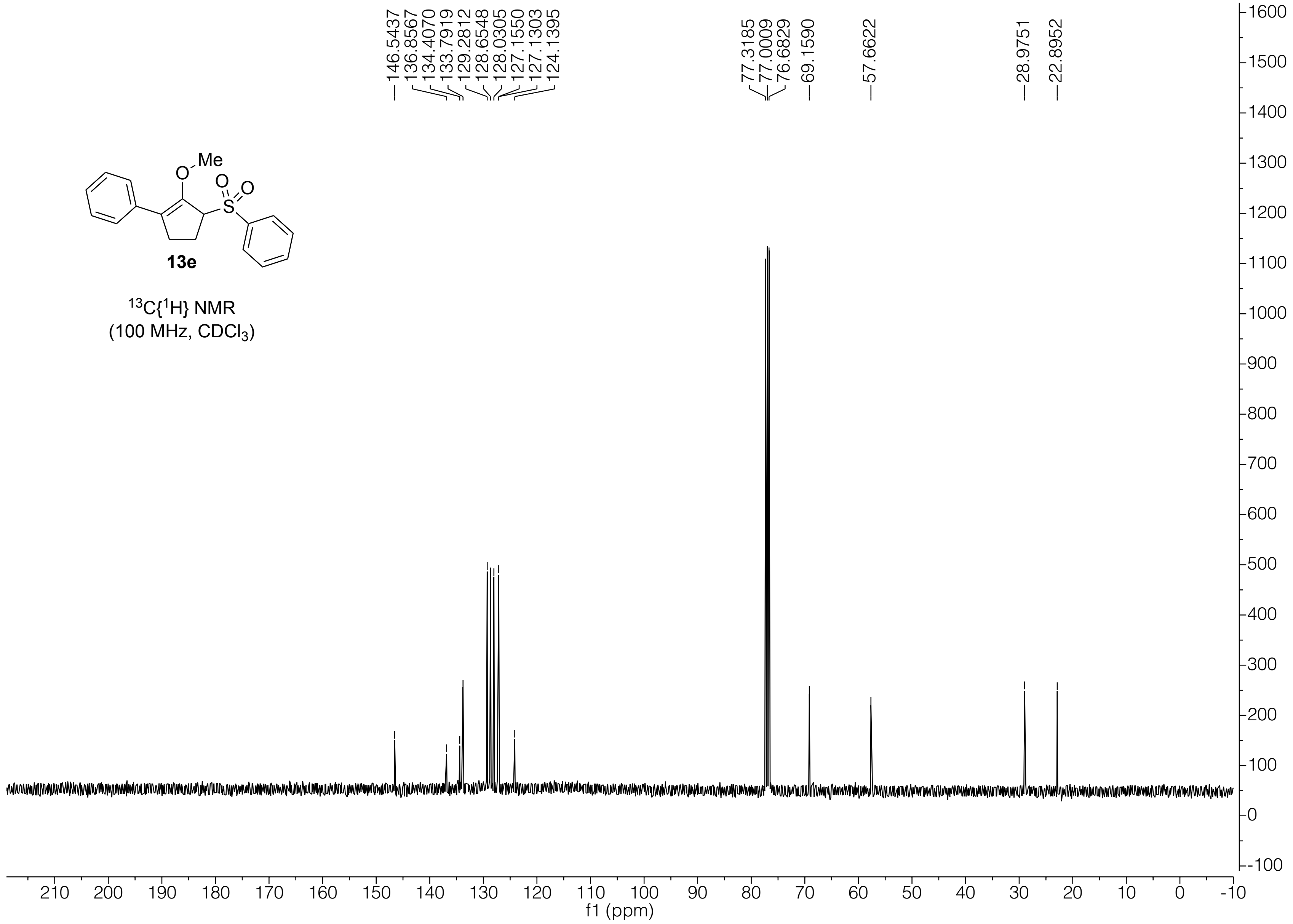

13e
$^{13}C\{^1H\}$ NMR
(100 MHz, $CDCl_3$)
146.5437
136.8567
134.4070
133.7919
129.2812
128.6548
128.0305
127.1550
127.1303
124.1395
77.3185
77.0009
76.6829
69.1590
57.6622
28.9751
22.8952
f1 (ppm)

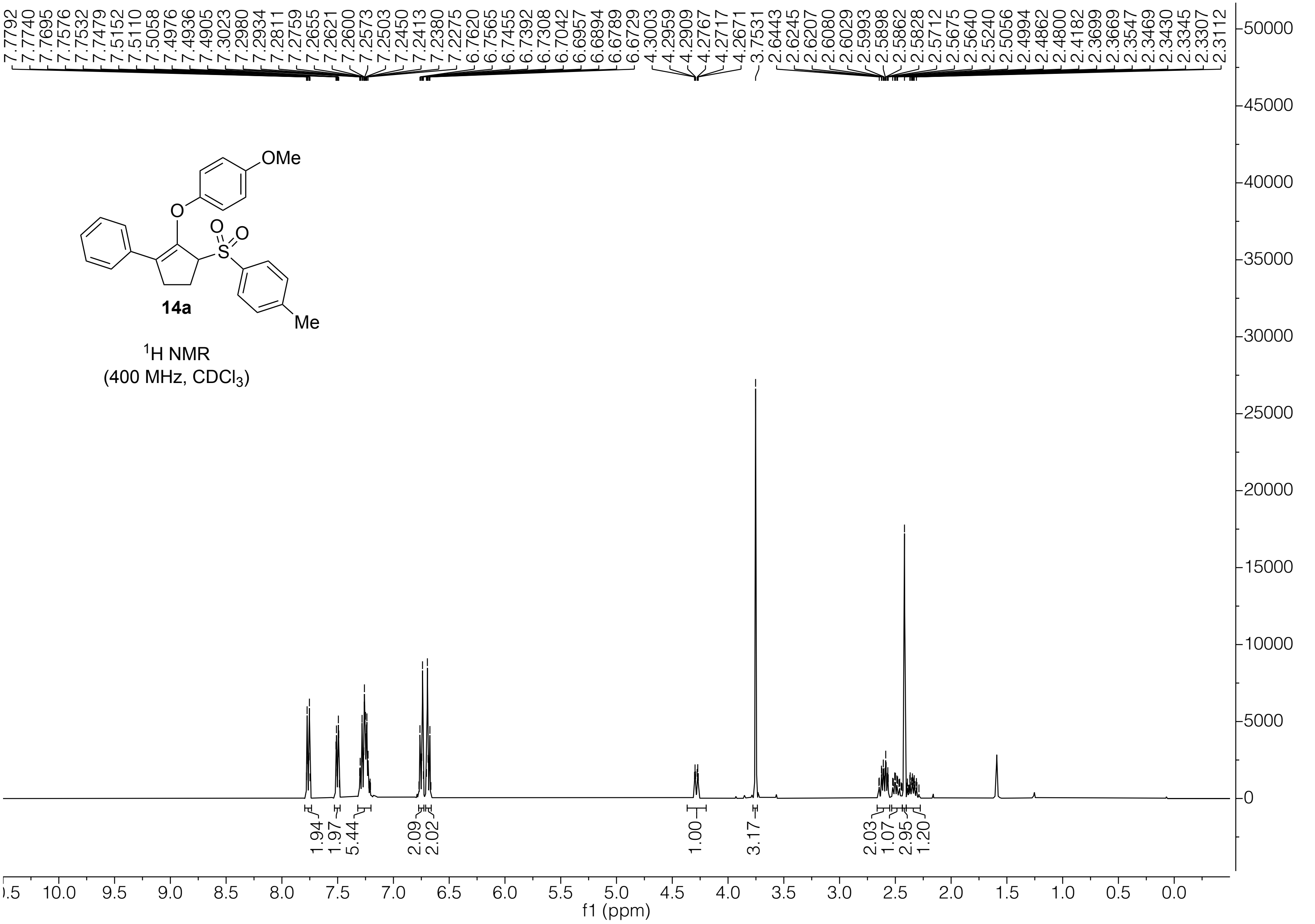
7.7792
7.7740
7.7695
7.7576
7.7532
7.7479
7.5152
7.5110
7.5058
7.4976
7.4936
7.4905
7.3023
7.2980
7.2934
7.2811
7.2759
7.2655
7.2621
7.2600
7.2573
7.2503
7.2450
7.2413
7.2380
7.2275
6.7620
6.7565
6.7455
6.7392
6.7308
6.7042
6.6957
6.6894
6.6789
6.6729
4.3003
4.2959
4.2909
4.2767
4.2717
4.2671
3.7531
2.6443
2.6245
2.6207
2.6080
2.6029
2.5993
2.5898
2.5862
2.5828
2.5712
2.5675
2.5640
2.5240
2.5056
2.4994
2.4862
2.4800
2.4182
2.3699
2.3669
2.3547
2.3469
2.3430
2.3345
2.3307
2.3112
OMe
O
O
S
O
Me
14a
1H NMR
(400 MHz, CDCl3)
1.94
1.97
5.44
2.09
2.02
1.00
3.17
2.03
1.07
2.95
1.20
f1 (ppm)

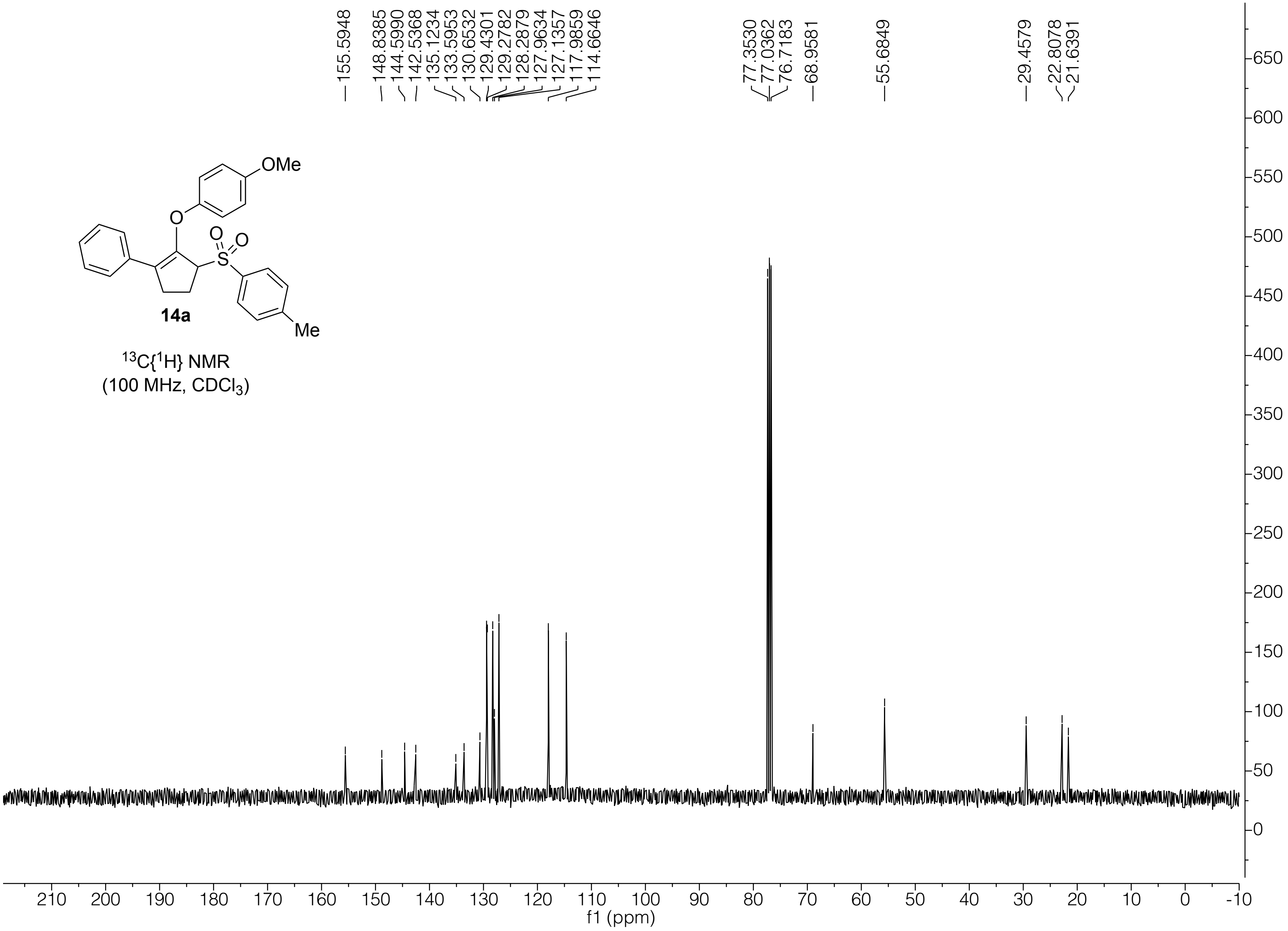

14a

$^{13}C\{^1H\}$ NMR
(100 MHz, $CDCl_3$)

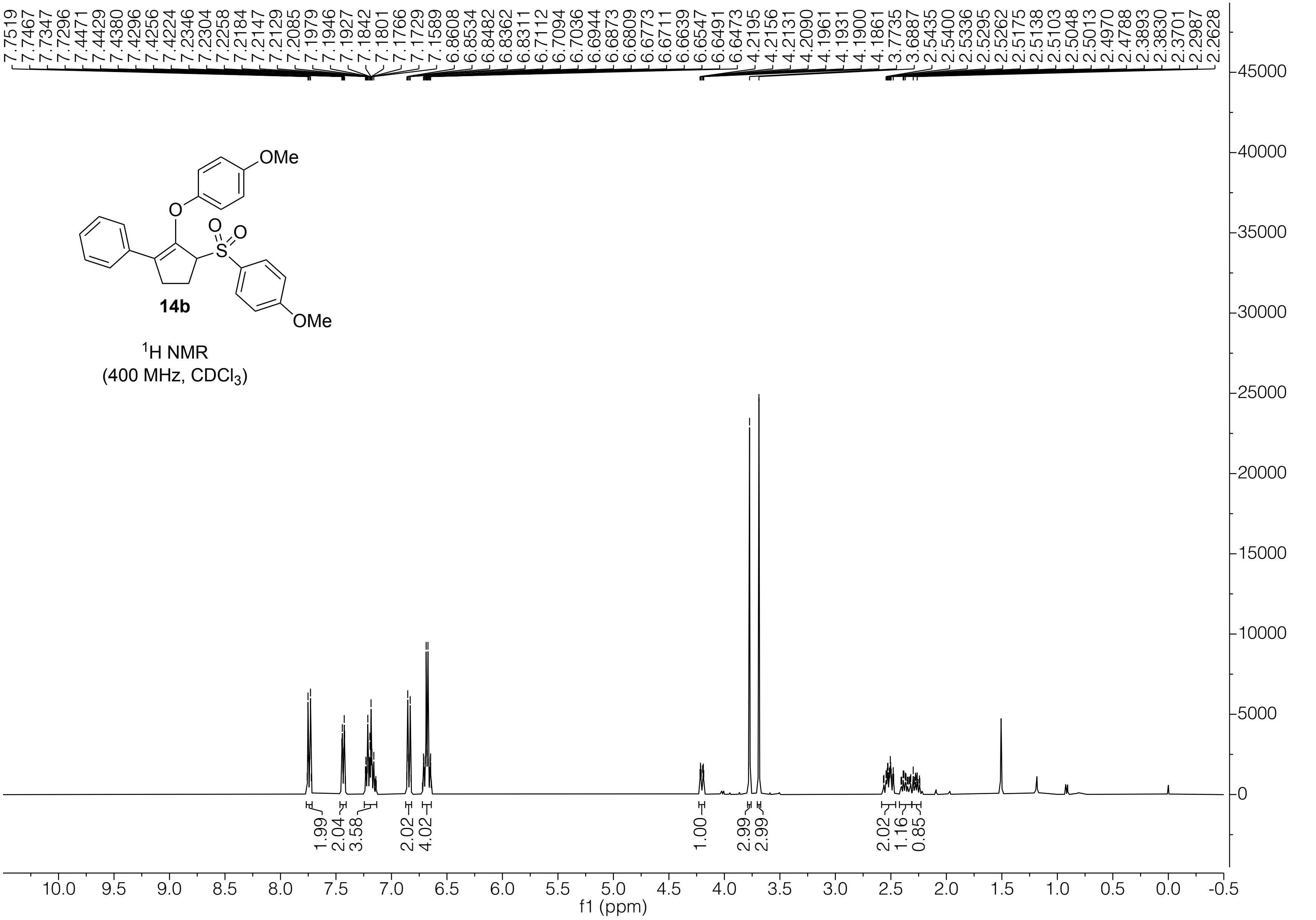
7.7519 7.7467 7.7347 7.7296 7.4471 7.4429 7.4380 7.4296 7.4256 7.4224 7.2346 7.2304 7.2258 7.2184 7.2147 7.2129 7.2085 7.1979 7.1946 7.1927 7.1842 7.1801 7.1766 7.1729 7.1589 6.8608 6.8534 6.8482 6.8362 6.8311 6.7112 6.7094 6.7036 6.6944 6.6873 6.6809 6.6773 6.6711 6.6639 6.6547 6.6491 6.6473 4.2195 4.2156 4.2131 4.2090 4.1961 4.1931 4.1900 4.1861 3.7735 3.6887 2.5435 2.5400 2.5336 2.5295 2.5262 2.5175 2.5138 2.5103 2.5048 2.5013 2.4970 2.4788 2.3893 2.3830 2.3701 2.2987 2.2628
OMe
O
O
S
O
OMe
14b
1H NMR
(400 MHz, CDCl3)
1.99 2.04 3.58 2.02 4.02 1.00 2.99 2.99 2.02 1.16 0.85
f1 (ppm)

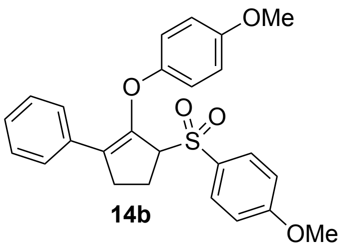

14b

$^{13}C\{^{1}H\}$ NMR
(125 MHz, $CDCl_3$)

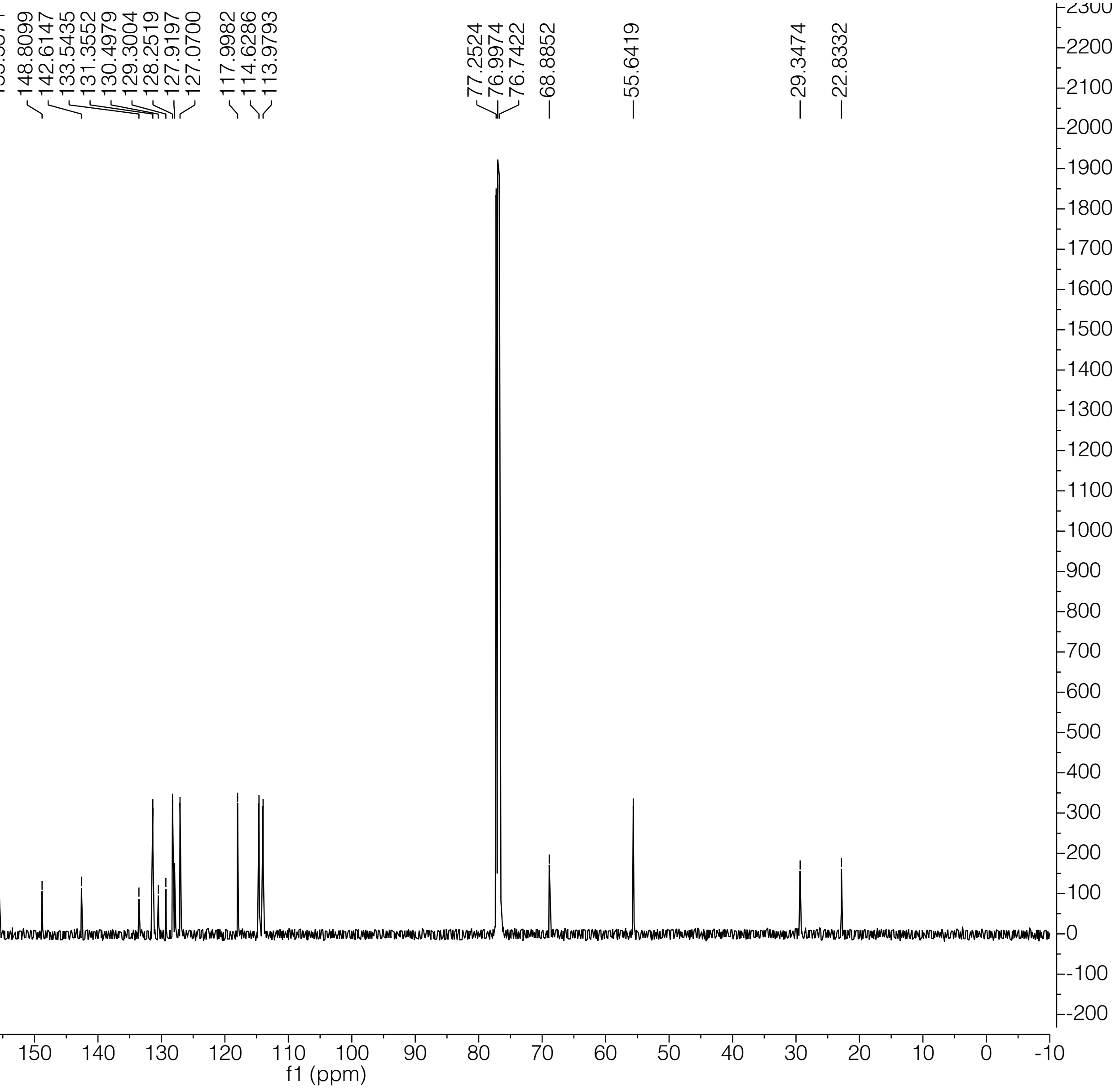

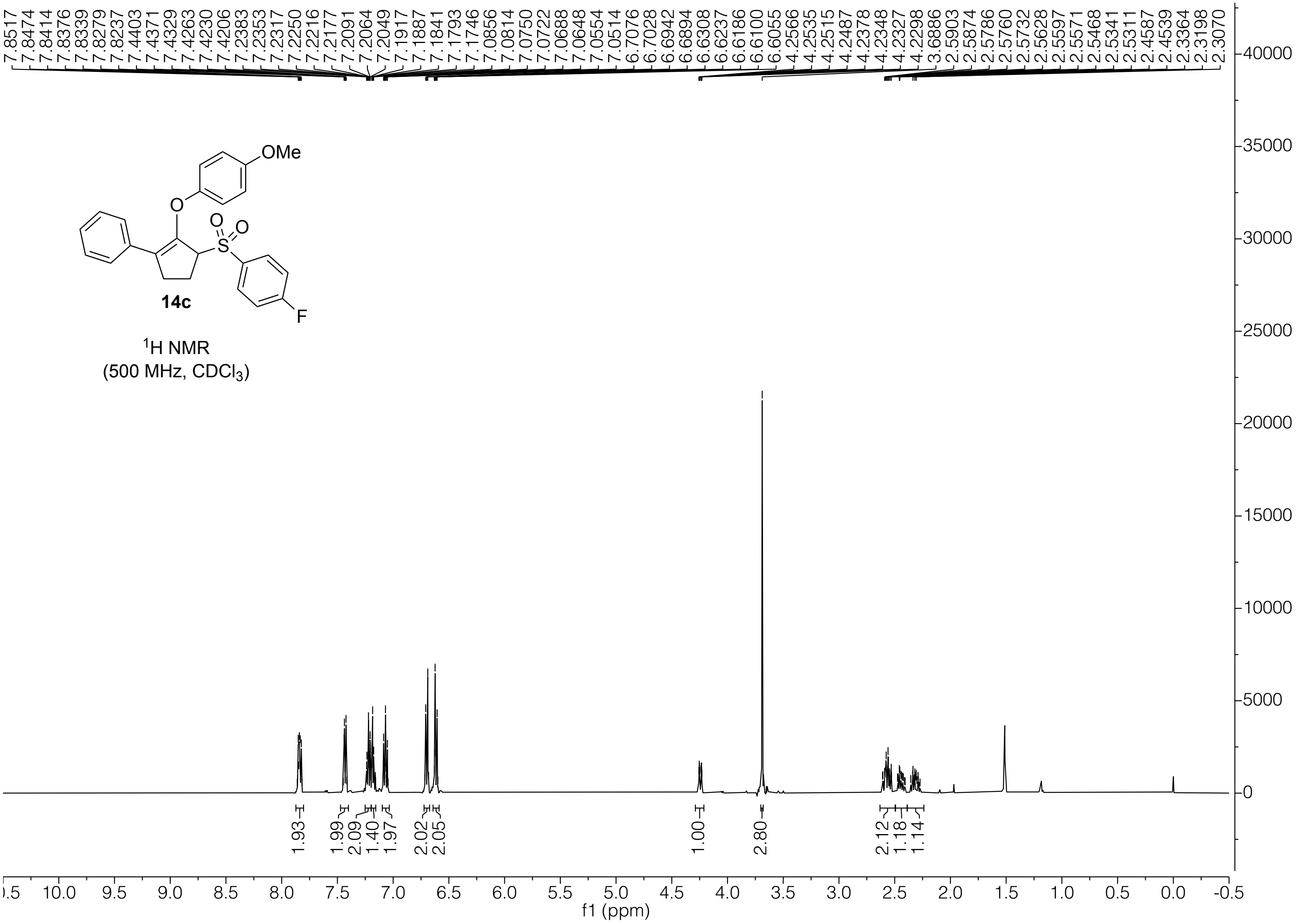
7.8517
7.8474
7.8414
7.8376
7.8339
7.8279
7.8237
7.4403
7.4371
7.4329
7.4263
7.4230
7.4206
7.2383
7.2353
7.2317
7.2250
7.2216
7.2177
7.2091
7.2064
7.2049
7.1917
7.1887
7.1841
7.1793
7.1746
7.0856
7.0814
7.0750
7.0722
7.0688
7.0648
7.0554
7.0514
6.7076
6.7028
6.6942
6.6894
6.6308
6.6237
6.6186
6.6100
6.6055
4.2566
4.2535
4.2515
4.2487
4.2378
4.2348
4.2327
4.2298
3.6886
2.5903
2.5874
2.5786
2.5760
2.5732
2.5628
2.5597
2.5571
2.5468
2.5341
2.5311
2.4587
2.4539
2.3364
2.3198
2.3070
OMe
O
O
S
O
F
14c
1H NMR
(500 MHz, CDCl3)
1.93
1.99
2.09
1.40
1.97
2.02
2.05
1.00
2.80
2.12
1.18
1.14
10.0 9.5 9.0 8.5 8.0 7.5 7.0 6.5 6.0 5.5 5.0 4.5 4.0 3.5 3.0 2.5 2.0 1.5 1.0 0.5 0.0 -0.5
f1 (ppm)
40000
35000
30000
25000
20000
15000
10000
5000
0

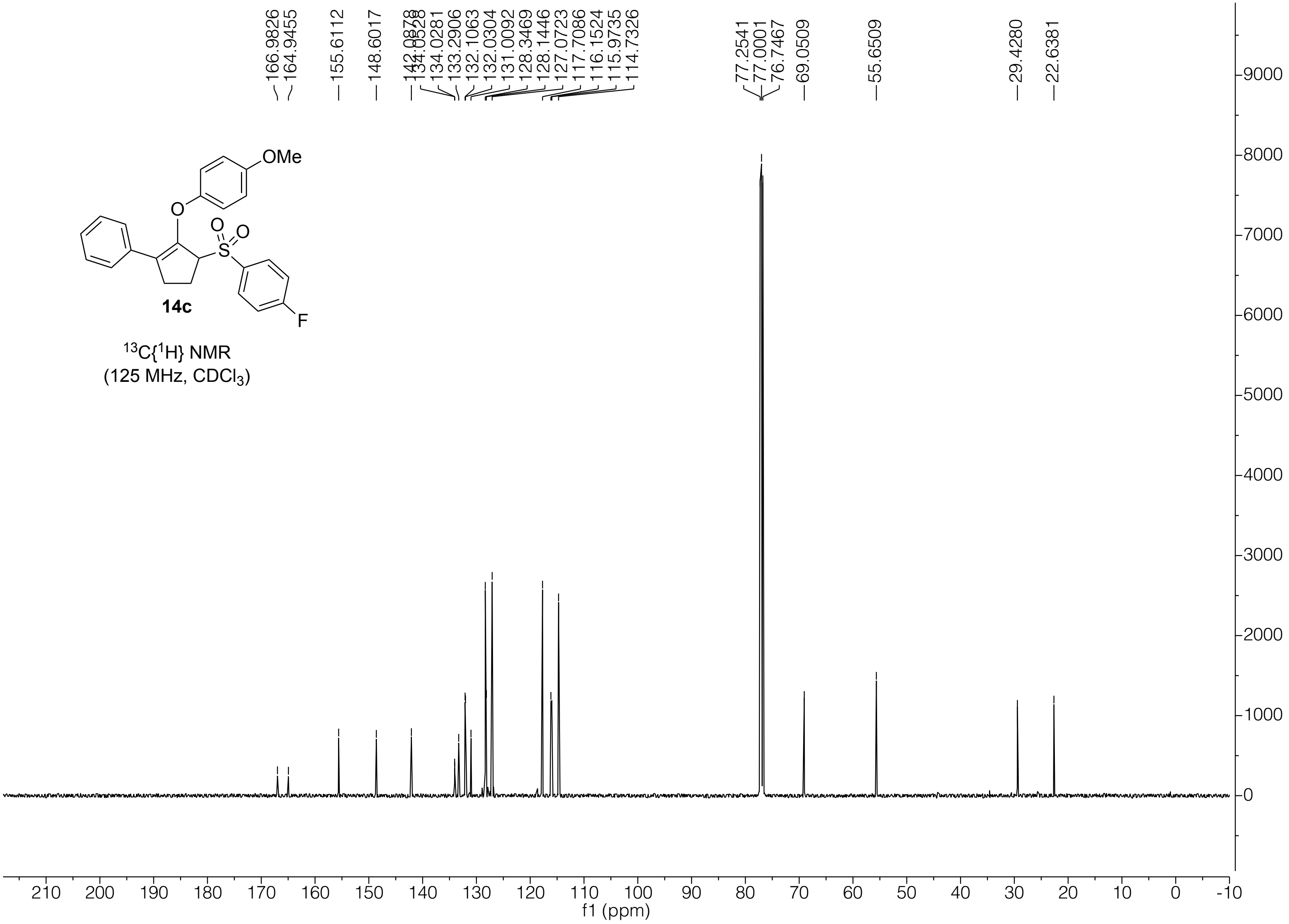

166.9826
164.9455
155.6112
148.6017
142.0878
134.0528
134.0281
133.2906
132.1063
132.0304
131.0092
128.3469
128.1446
127.0723
117.7086
116.1524
115.9735
114.7326
77.2541
77.0001
76.7467
69.0509
55.6509
29.4280
22.6381
OMe
O
O
S
O
F
14c
$^{13}C\{^1H\}$ NMR
(125 MHz, $CDCl_3$)
9000
8000
7000
6000
5000
4000
3000
2000
1000
0
210 200 190 180 170 160 150 140 130 120 110 100 90 80 70 60 50 40 30 20 10 0 -10
f1 (ppm)

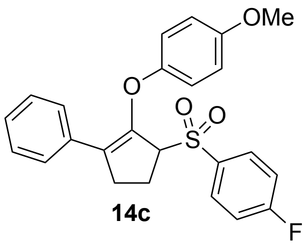

14c

^{19}F NMR
(470 MHz, $CDCl_3$)

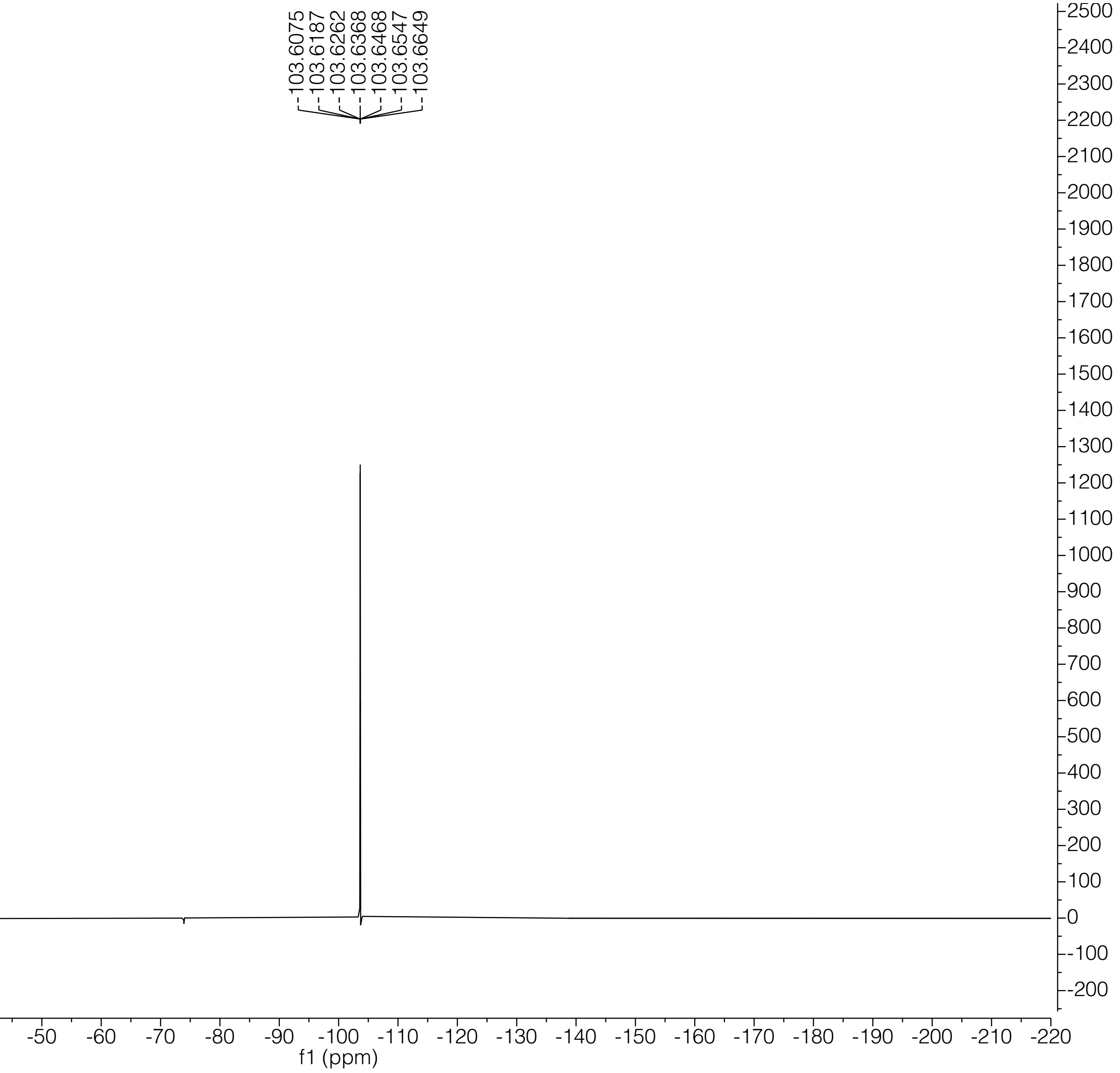

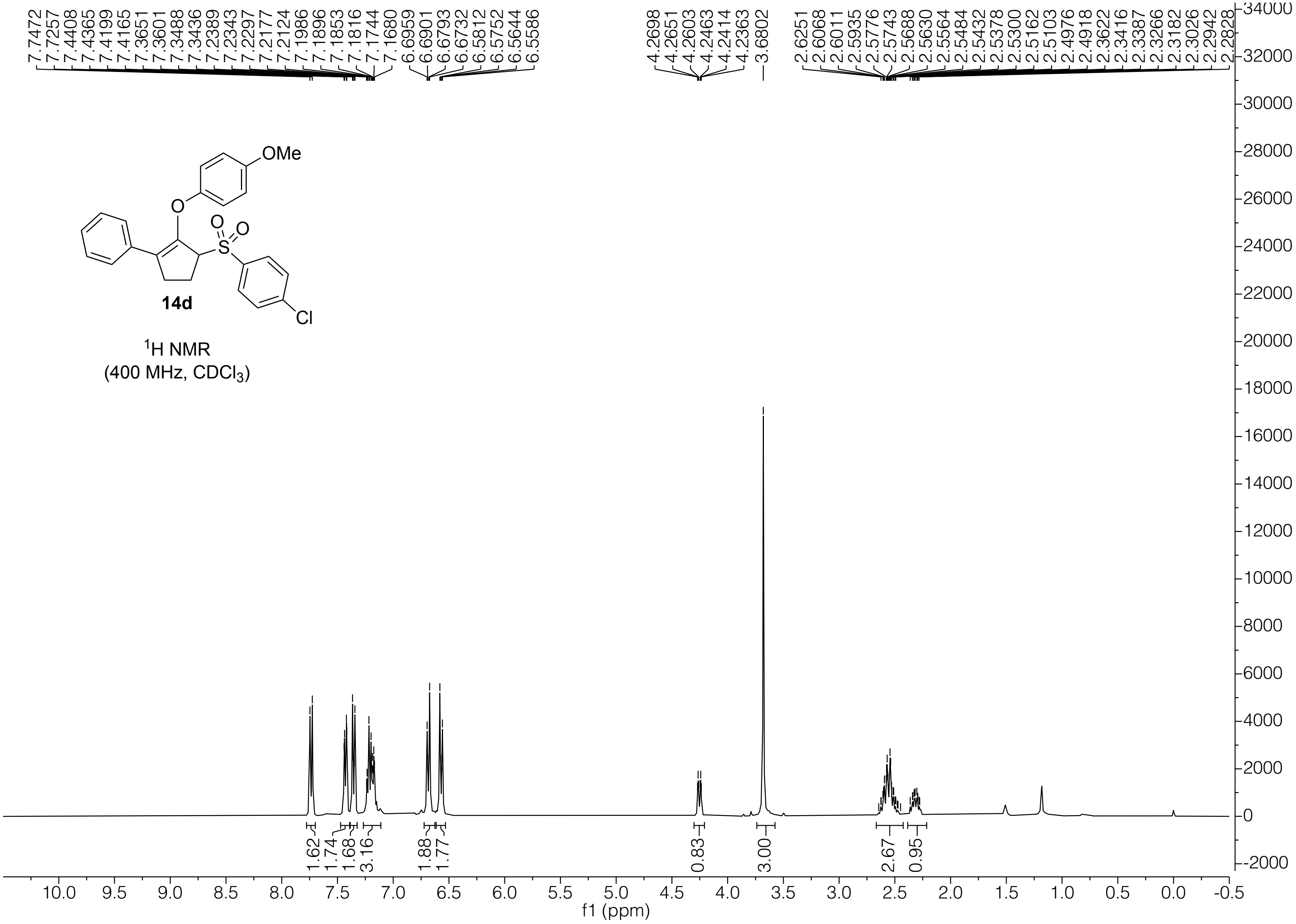
OMe
O
O
S
O
Cl
14d
^{1}H NMR
(400 MHz, $CDCl_3$)
f1 (ppm)

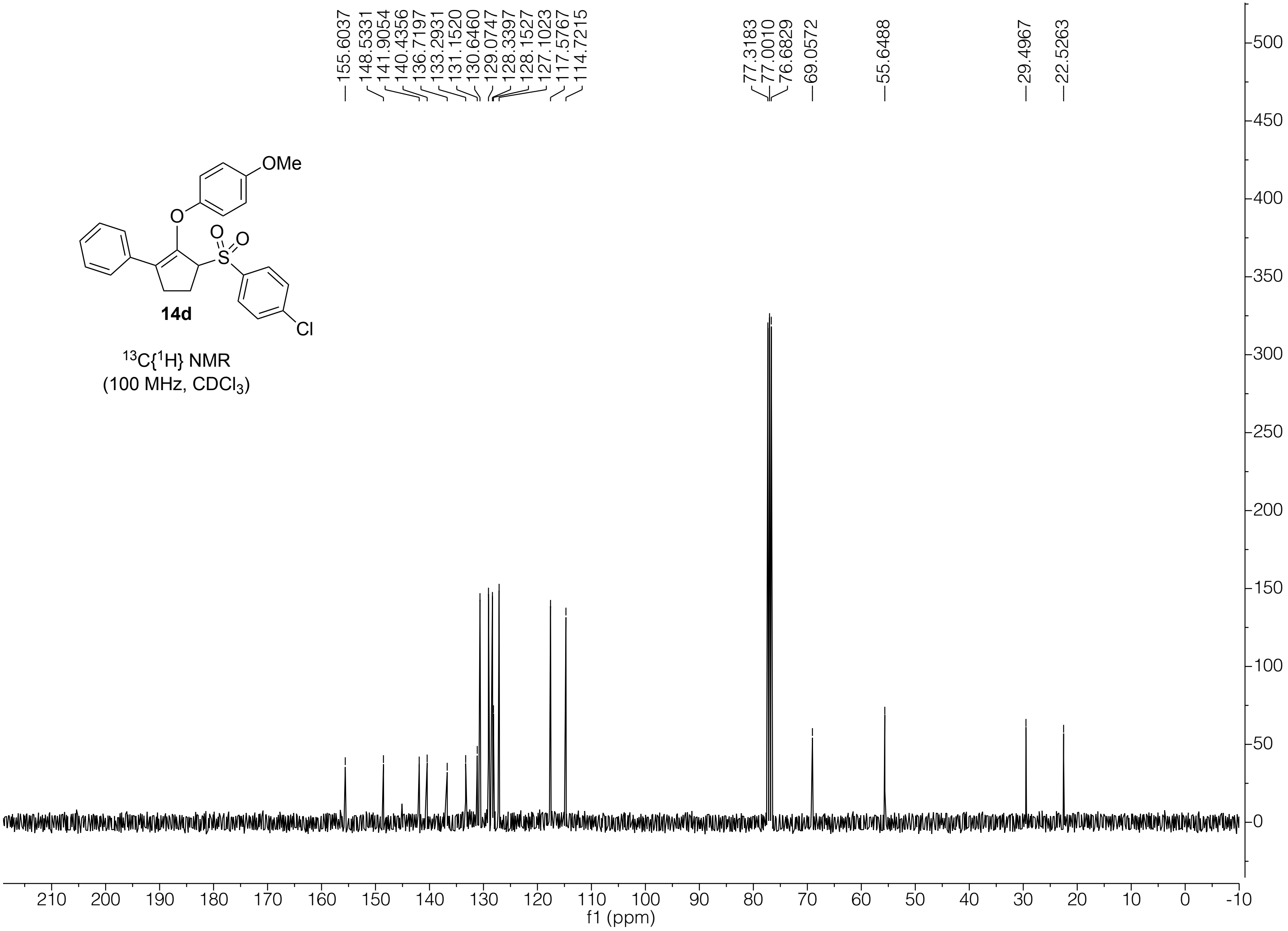
155.6037
148.5331
141.9054
140.4356
136.7197
133.2931
131.1520
130.6460
129.0747
128.3397
128.1527
127.1023
117.5767
114.7215
77.3183
77.0010
76.6829
69.0572
55.6488
29.4967
22.5263
OMe
O
O
S
O
Cl
14d
$^{13}C\{^{1}H\}$ NMR
(100 MHz, $CDCl_3$)
f1 (ppm)

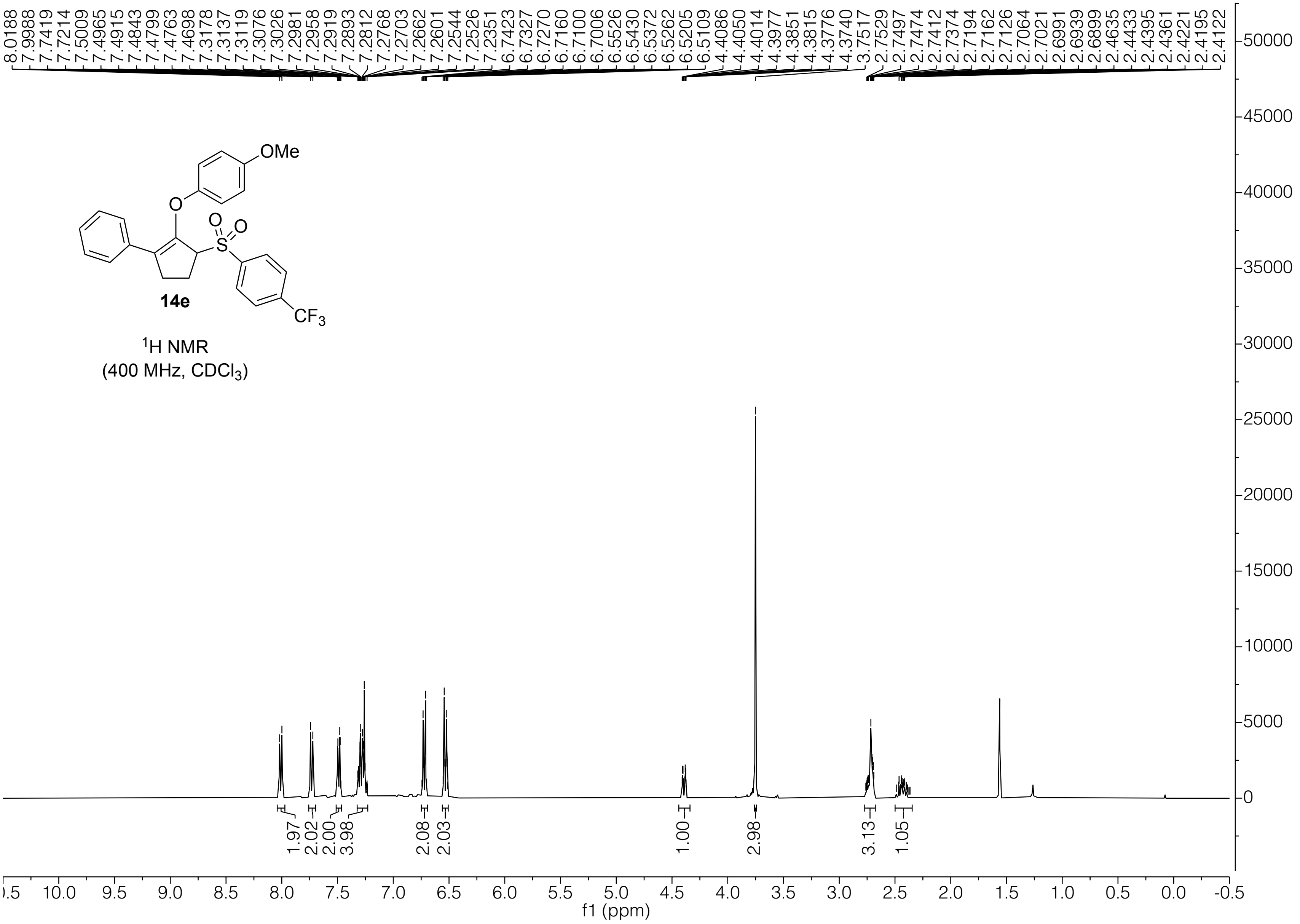

OMe
O
O
O
S
CF3
14e
1H NMR
(400 MHz, CDCl3)
8.0188
7.9988
7.7419
7.7214
7.5009
7.4965
7.4915
7.4843
7.4799
7.4763
7.4698
7.3178
7.3137
7.3119
7.3076
7.3026
7.2981
7.2958
7.2919
7.2893
7.2812
7.2768
7.2703
7.2662
7.2601
7.2544
7.2526
7.2351
6.7423
6.7327
6.7270
6.7160
6.7100
6.7006
6.5526
6.5430
6.5372
6.5262
6.5205
6.5109
4.4086
4.4050
4.4014
4.3977
4.3851
4.3815
4.3776
4.3740
3.7517
2.7529
2.7497
2.7474
2.7412
2.7374
2.7194
2.7162
2.7126
2.7064
2.7021
2.6991
2.6939
2.6899
2.4635
2.4433
2.4395
2.4361
2.4221
2.4195
2.4122
1.97
2.02
2.00
3.98
2.08
2.03
1.00
2.98
3.13
1.05
50000
45000
40000
35000
30000
25000
20000
15000
10000
5000
0
10.0
9.5
9.0
8.5
8.0
7.5
7.0
6.5
6.0
5.5
5.0
4.5
4.0
3.5
3.0
2.5
2.0
1.5
1.0
0.5
0.0
-0.5
f1 (ppm)

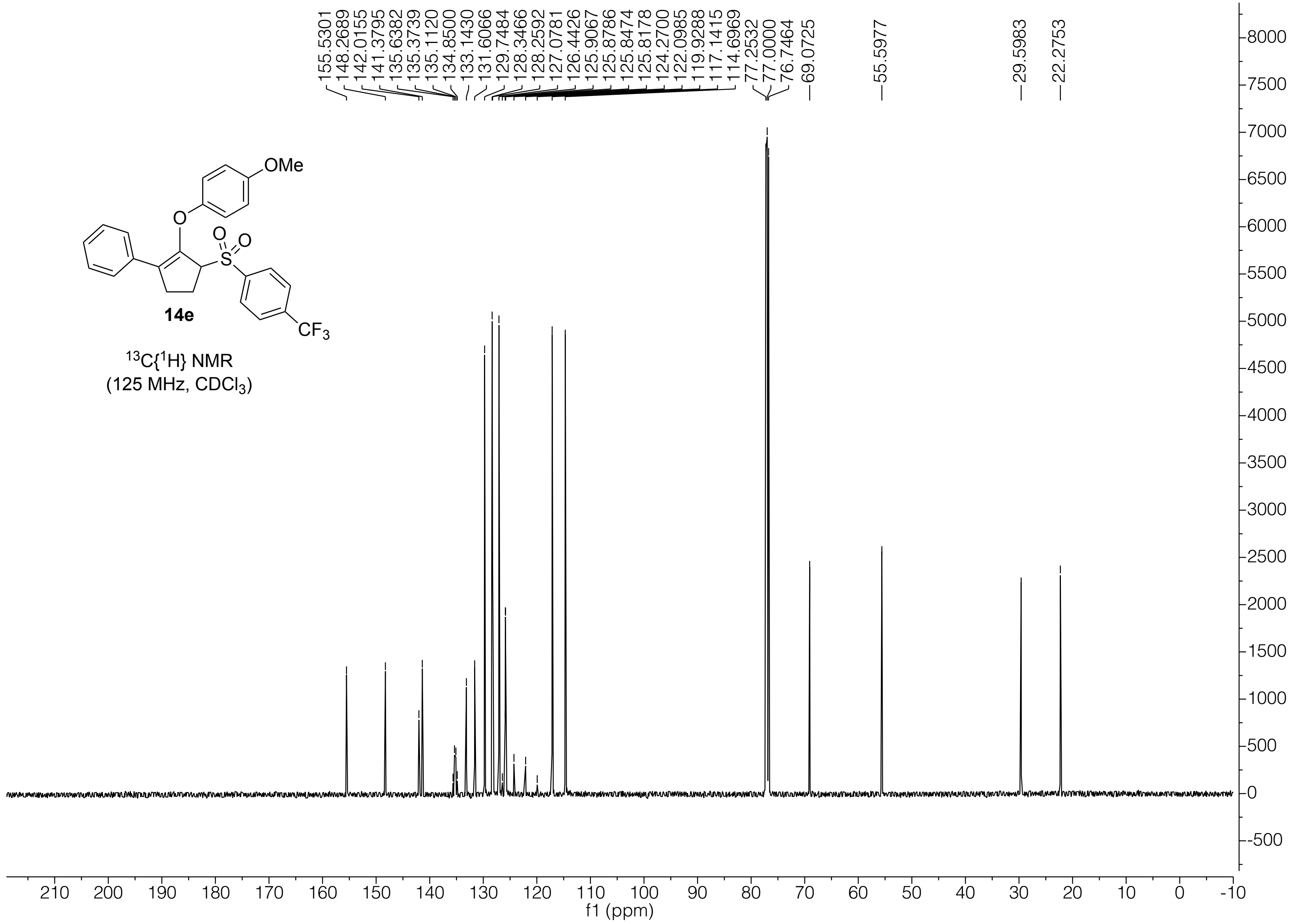
155.5301
148.2689
142.0155
141.3795
135.6382
135.3739
135.1120
134.8500
133.1430
131.6066
129.7484
128.3466
128.2592
127.0781
126.4426
125.9067
125.8786
125.8474
125.8178
124.2700
122.0985
119.9288
117.1415
114.6969
77.2532
77.0000
76.7464
69.0725
55.5977
29.5983
22.2753
OMe
O
O
S
O
14e
CF3
13C{1H} NMR
(125 MHz, CDCl3)
f1 (ppm)

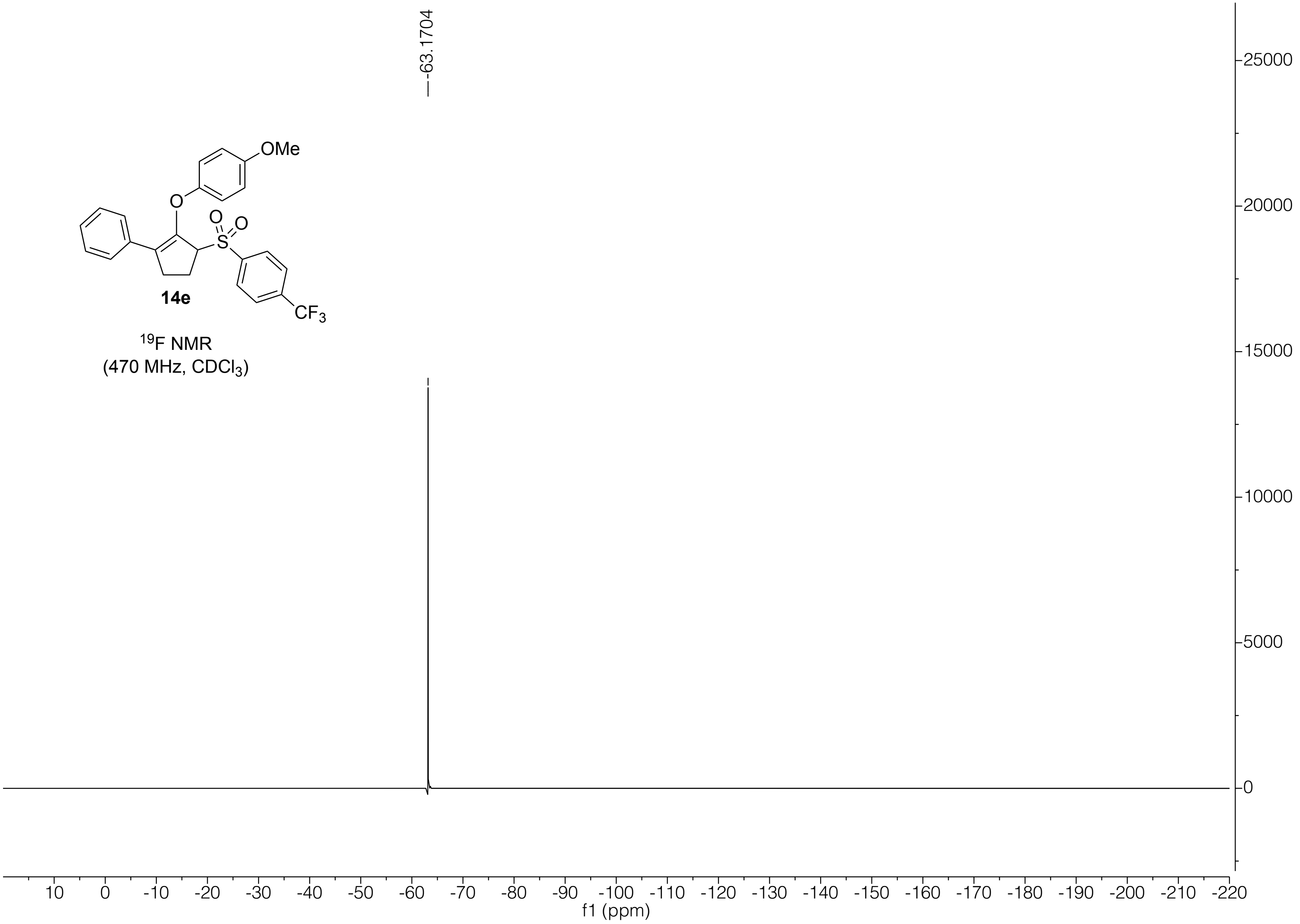
-63.1704
OMe
O
O
S
O
14e
CF3
19F NMR
(470 MHz, CDCl3)
25000
20000
15000
10000
5000
0
10 0 -10 -20 -30 -40 -50 -60 -70 -80 -90 -100 -110 -120 -130 -140 -150 -160 -170 -180 -190 -200 -210 -220
f1 (ppm)

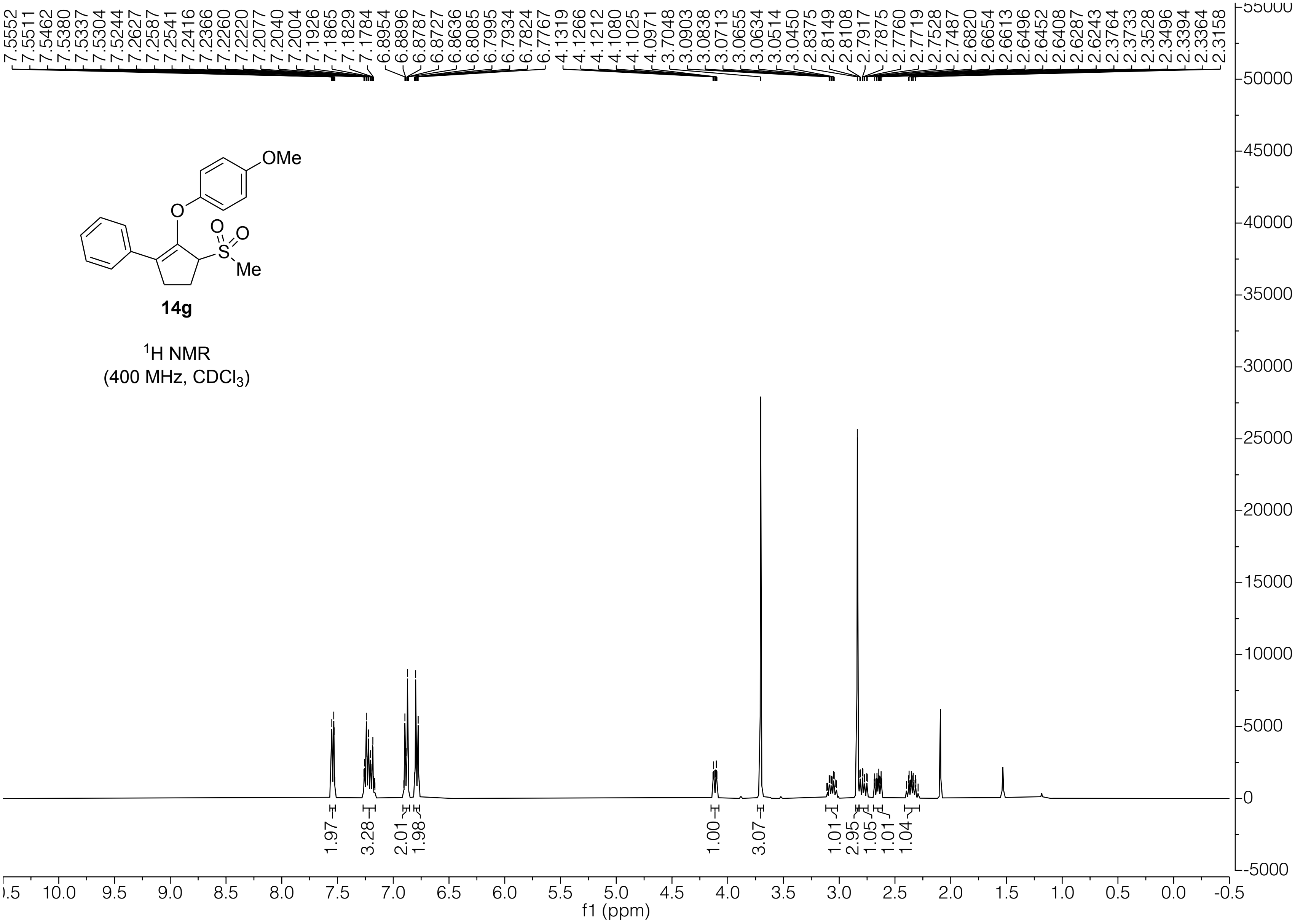
7.5552
7.5511
7.5462
7.5380
7.5337
7.5304
7.5244
7.2627
7.2587
7.2541
7.2416
7.2366
7.2260
7.2220
7.2077
7.2040
7.2004
7.1926
7.1865
7.1829
7.1784
6.8954
6.8896
6.8787
6.8727
6.8636
6.8085
6.7995
6.7934
6.7824
6.7767
4.1319
4.1266
4.1212
4.1080
4.1025
4.0971
3.7048
3.0903
3.0838
3.0713
3.0655
3.0634
3.0514
3.0450
2.8375
2.8149
2.8108
2.7917
2.7875
2.7760
2.7719
2.7528
2.7487
2.6820
2.6654
2.6613
2.6496
2.6452
2.6408
2.6287
2.6243
2.3764
2.3733
2.3528
2.3496
2.3394
2.3364
2.3158
OMe
O
O
S
O
Me
14g
1H NMR
(400 MHz, CDCl3)
1.97
3.28
2.01
1.98
1.00
3.07
1.01
2.95
1.05
1.01
1.04
f1 (ppm)

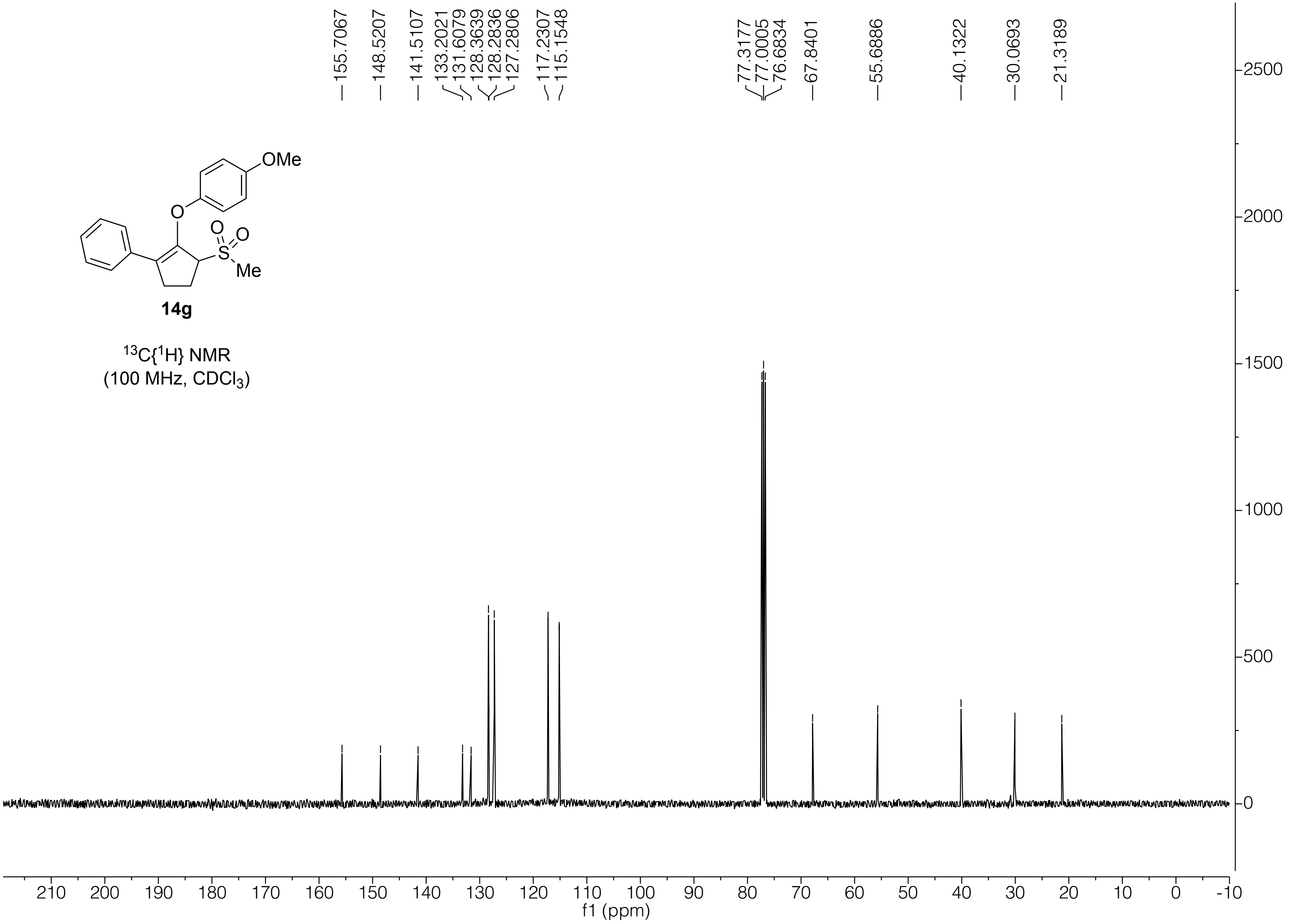
155.7067
148.5207
141.5107
133.2021
131.6079
128.3639
128.2836
127.2806
117.2307
115.1548
77.3177
77.0005
76.6834
67.8401
55.6886
40.1322
30.0693
21.3189
OMe
O
O
S
O
Me
14g
$^{13}C\{^1H\}$ NMR
(100 MHz, $CDCl_3$)
2500
2000
1500
1000
500
0
210 200 190 180 170 160 150 140 130 120 110 100 90 80 70 60 50 40 30 20 10 0 -10
f1 (ppm)

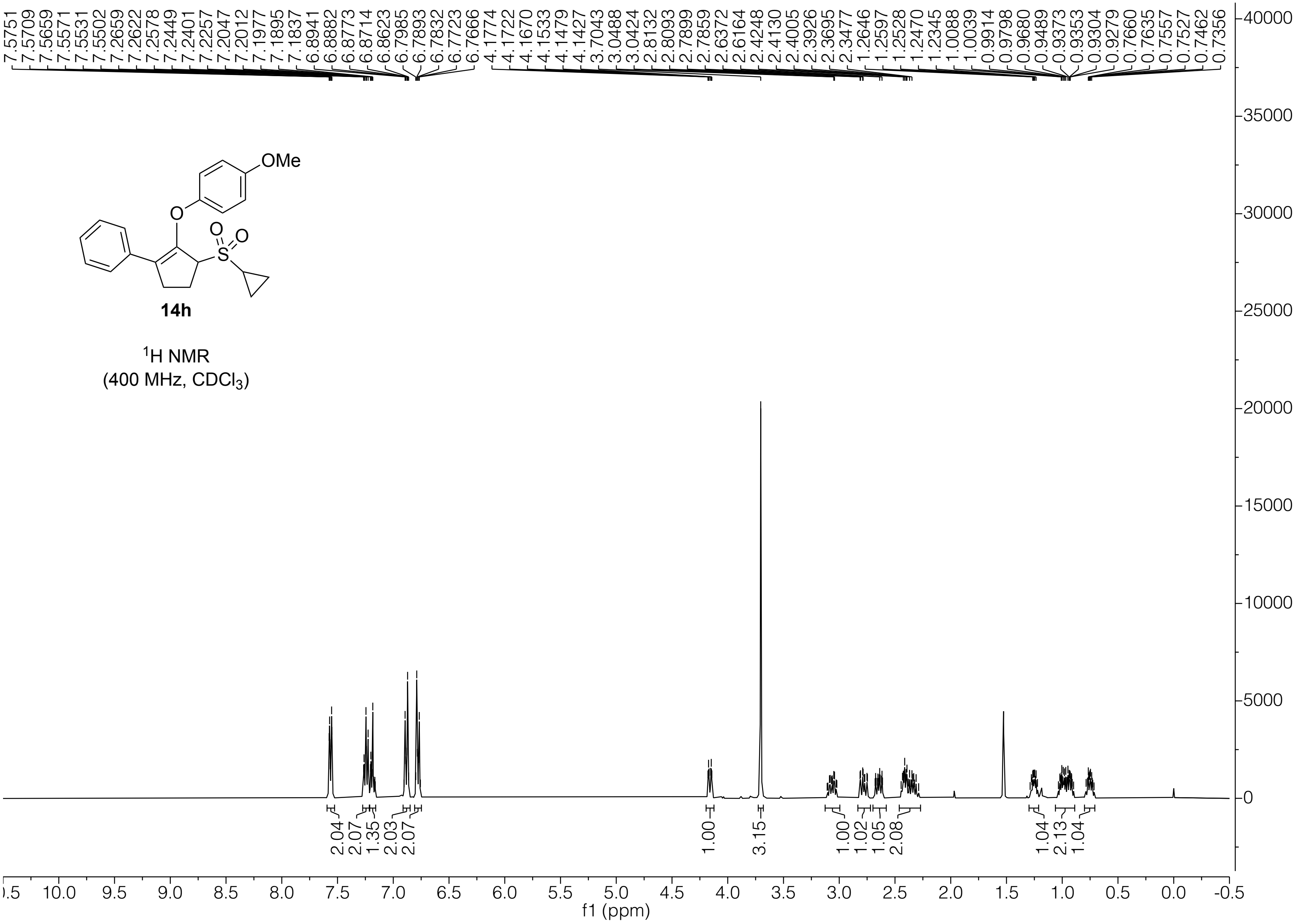
OMe
O
O
S
O
14h
^{1}H NMR
(400 MHz, CDCl$_3$)
7.5751 7.5709 7.5659 7.5571 7.5531 7.5502 7.2659 7.2622 7.2578 7.2449 7.2401 7.2257 7.2047 7.2012 7.1977 7.1895 7.1837 6.8941 6.8882 6.8773 6.8714 6.8623 6.7985 6.7893 6.7832 6.7723 6.7666 4.1774 4.1722 4.1670 4.1533 4.1479 4.1427 3.7043 3.0488 3.0424 2.8132 2.8093 2.7899 2.7859 2.6372 2.6164 2.4248 2.4130 2.4005 2.3926 2.3695 2.3477 1.2646 1.2597 1.2528 1.2470 1.2345 1.0088 1.0039 0.9914 0.9798 0.9680 0.9489 0.9373 0.9353 0.9304 0.9279 0.7660 0.7635 0.7557 0.7527 0.7462 0.7356
2.04 2.07 1.35 2.03 2.07 1.00 3.15 1.00 1.02 1.05 2.08 1.04 2.13 1.04
f1 (ppm)

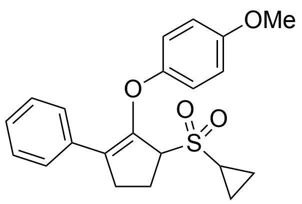

14h

$^{13}C\{^{1}H\}$ NMR
(100 MHz, $CDCl_3$)

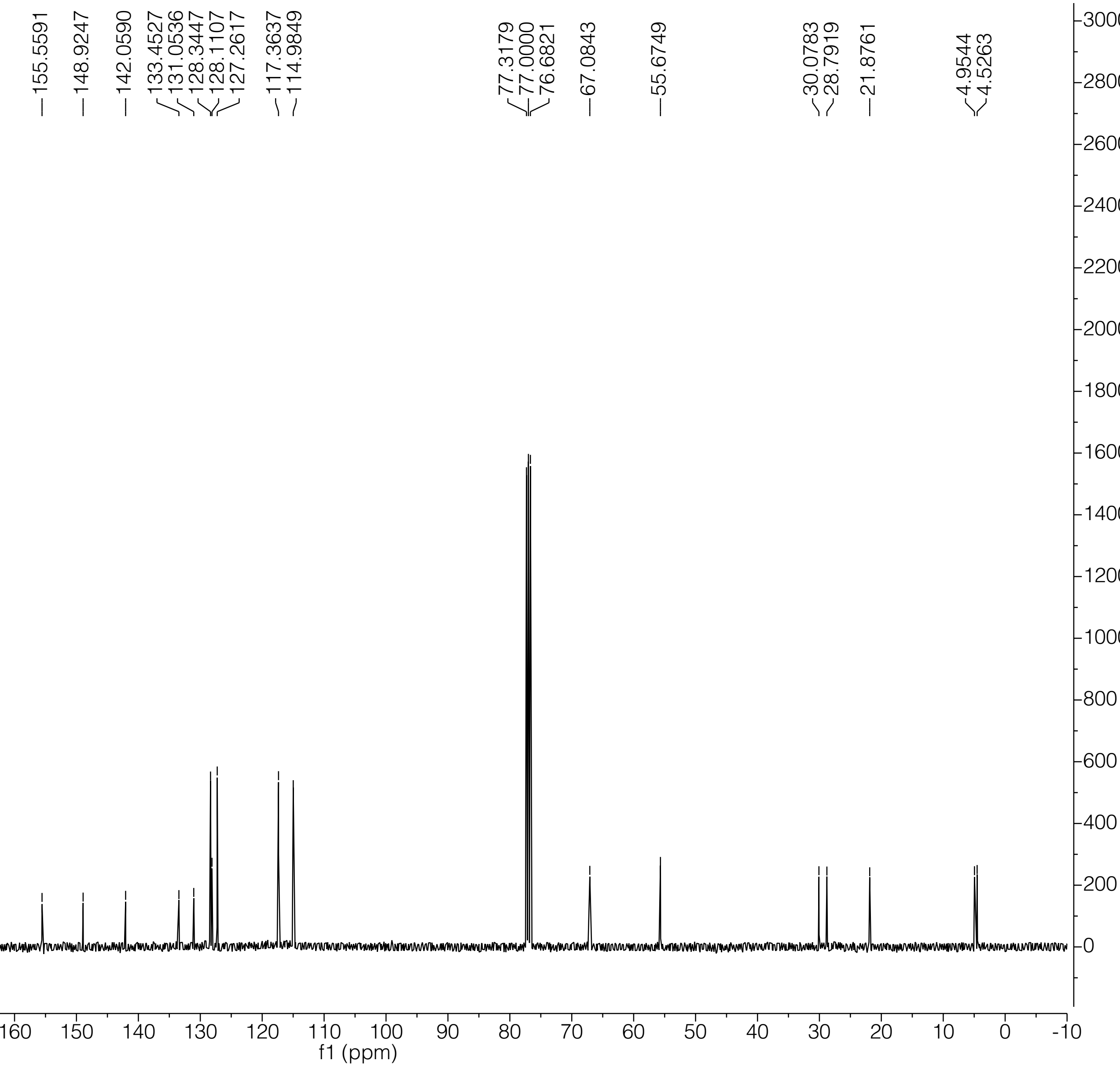

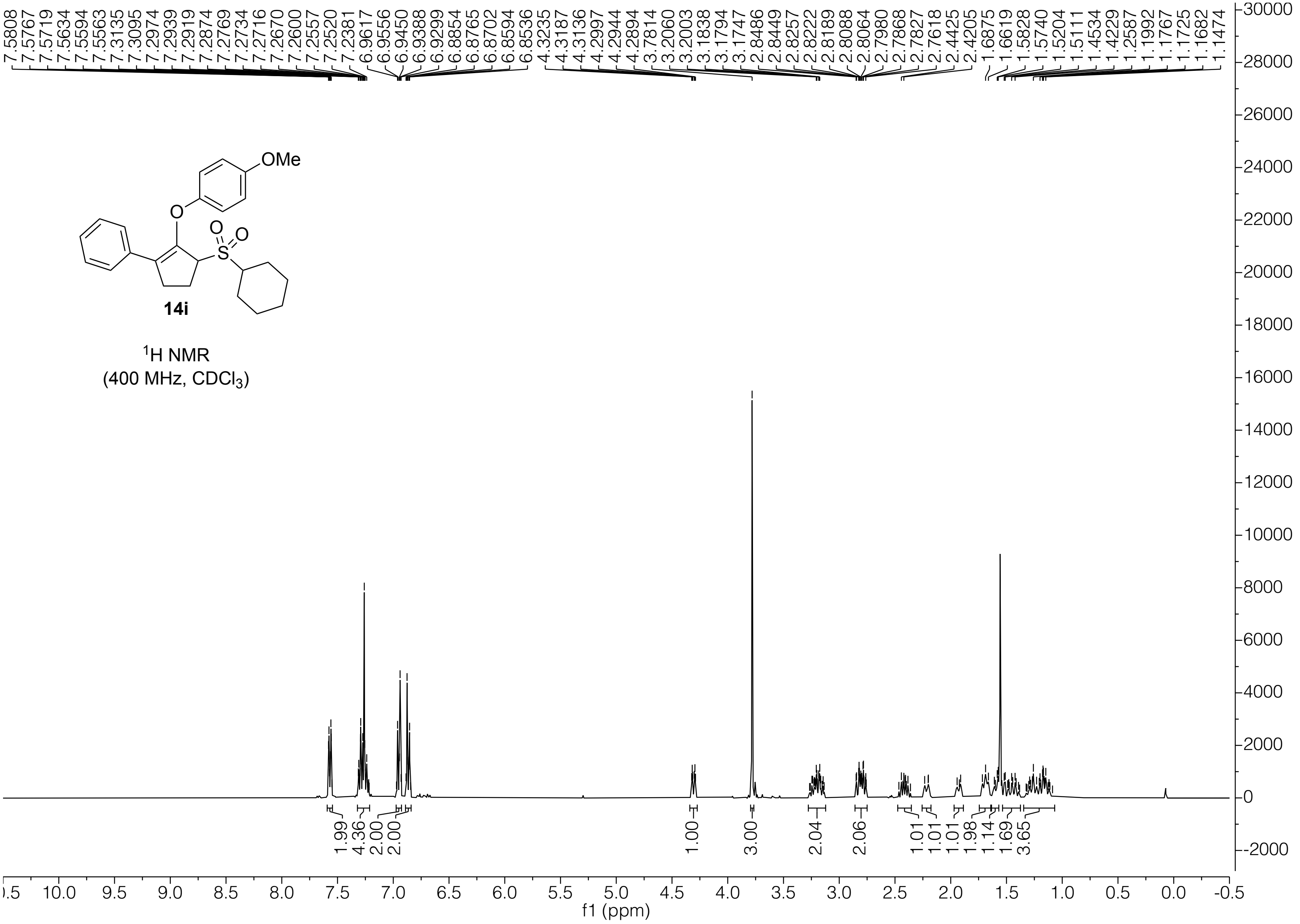

7.5808
7.5767
7.5719
7.5634
7.5594
7.5563
7.3135
7.3095
7.2974
7.2939
7.2919
7.2874
7.2769
7.2734
7.2716
7.2670
7.2600
7.2557
7.2520
7.2381
6.9617
6.9556
6.9450
6.9388
6.9299
6.8854
6.8765
6.8702
6.8594
6.8536
4.3235
4.3187
4.3136
4.2997
4.2944
4.2894
3.7814
3.2060
3.2003
3.1838
3.1794
3.1747
2.8486
2.8449
2.8257
2.8222
2.8189
2.8088
2.8064
2.7980
2.7868
2.7827
2.7618
2.4425
2.4205
1.6875
1.6619
1.5828
1.5740
1.5204
1.5111
1.4534
1.4229
1.2587
1.1992
1.1767
1.1725
1.1682
1.1474
OMe
O
O
O
S
14i
^{1}H NMR
(400 MHz, CDCl$_3$)
1.99
4.36
2.00
2.00
1.00
3.00
2.04
2.06
1.01
1.01
1.01
1.98
1.14
1.69
3.65
f1 (ppm)

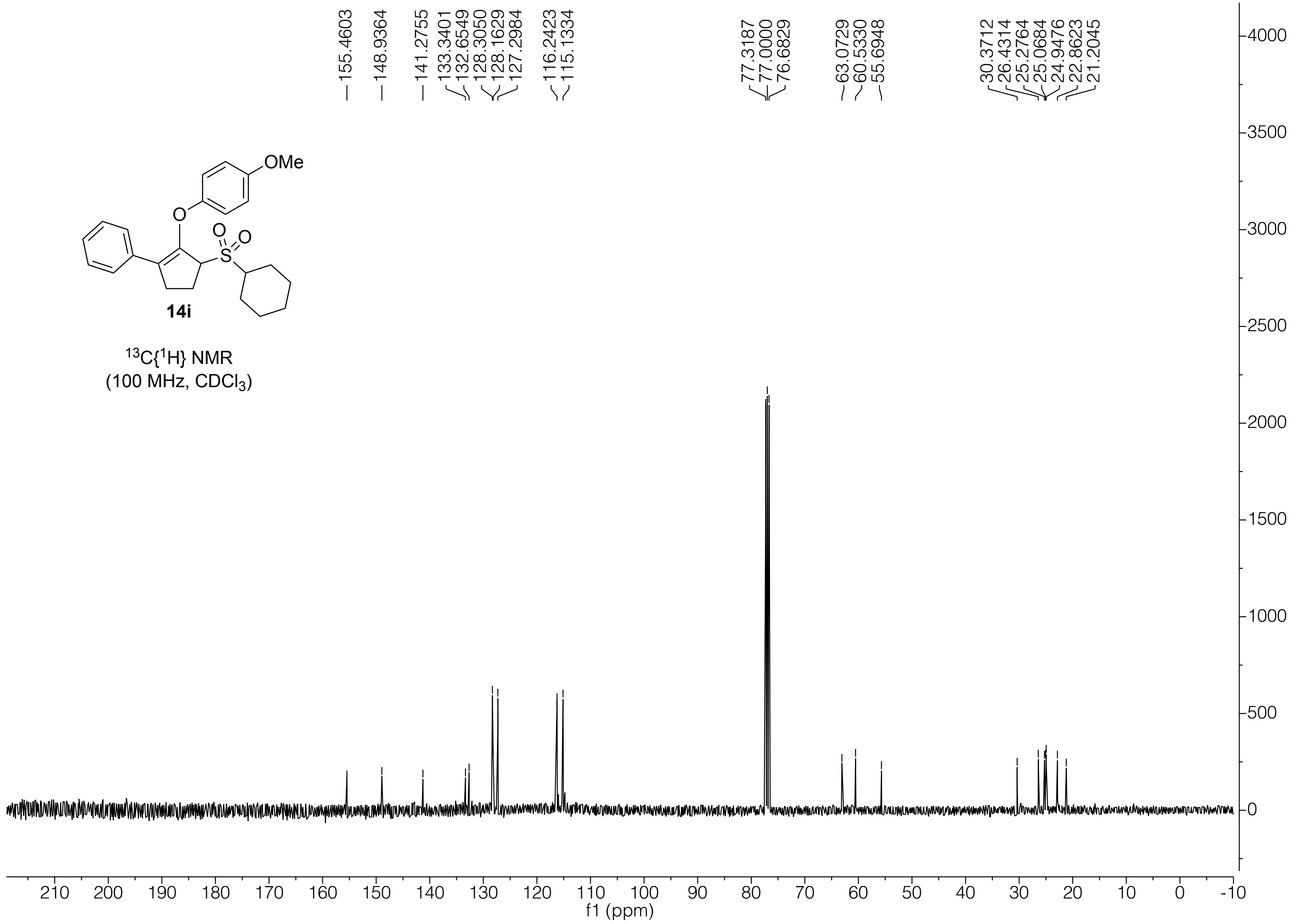
155.4603
148.9364
141.2755
133.3401
132.6549
128.3050
128.1629
127.2984
116.2423
115.1334
77.3187
77.0000
76.6829
63.0729
60.5330
55.6948
30.3712
26.4314
25.2764
25.0684
24.9476
22.8623
21.2045
OMe
O
14i
$^{13}C\{^1H\}$ NMR
(100 MHz, $CDCl_3$)
f1 (ppm)

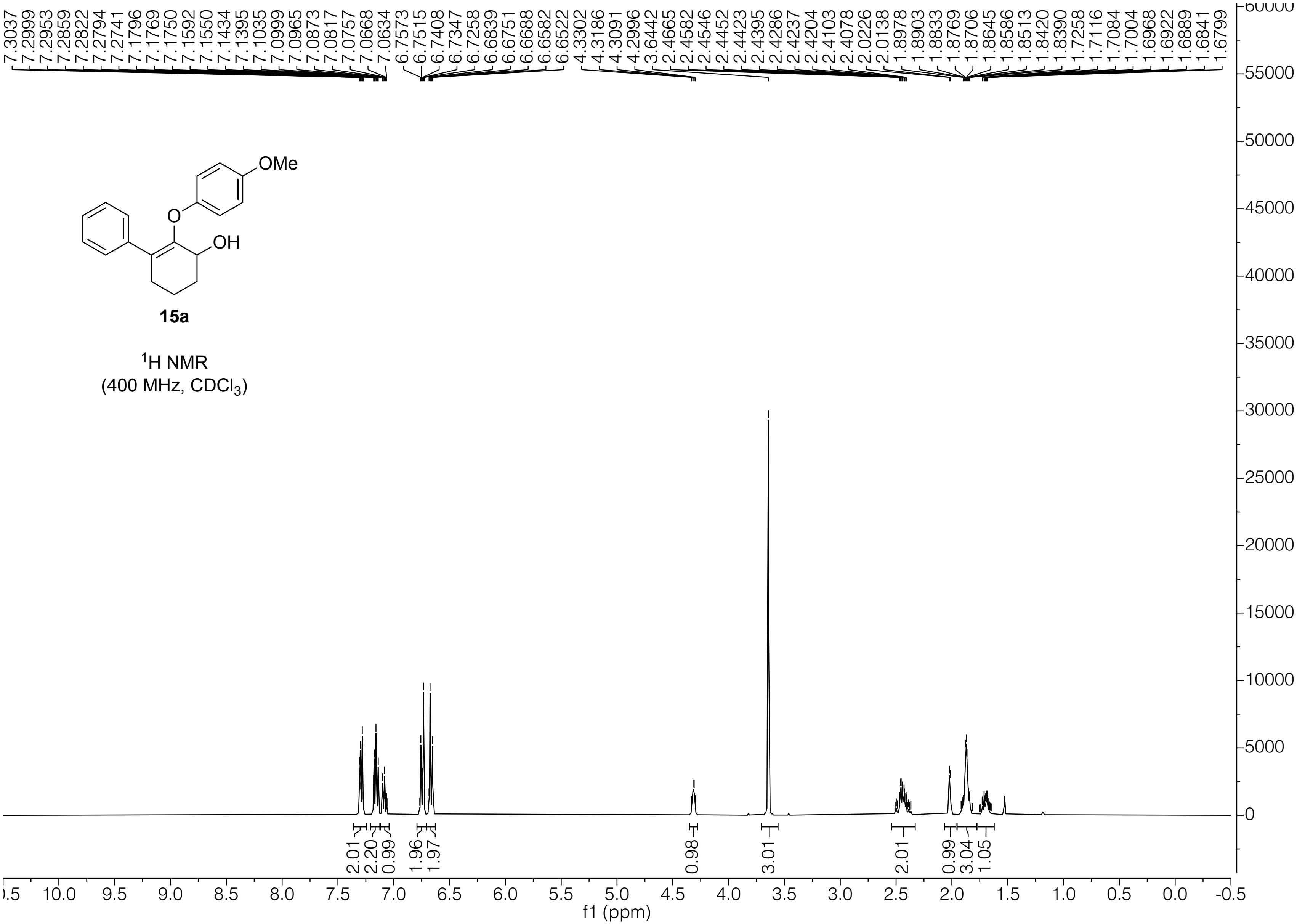

7.3037
7.2999
7.2953
7.2859
7.2822
7.2794
7.2741
7.1796
7.1769
7.1750
7.1592
7.1550
7.1434
7.1395
7.1035
7.0999
7.0965
7.0873
7.0817
7.0757
7.0668
7.0634
6.7573
6.7515
6.7408
6.7347
6.7258
6.6839
6.6751
6.6688
6.6582
6.6522
4.3302
4.3186
4.3091
4.2996
3.6442
2.4665
2.4582
2.4546
2.4452
2.4423
2.4395
2.4286
2.4237
2.4204
2.4103
2.4078
2.0226
2.0138
1.8978
1.8903
1.8833
1.8769
1.8706
1.8645
1.8586
1.8513
1.8420
1.8390
1.7258
1.7116
1.7084
1.7004
1.6968
1.6922
1.6889
1.6841
1.6799
OMe
O
OH
15a
1H NMR
(400 MHz, CDCl3)
2.01
2.20
0.99
1.96
1.97
0.98
3.01
2.01
0.99
3.04
1.05
f1 (ppm)

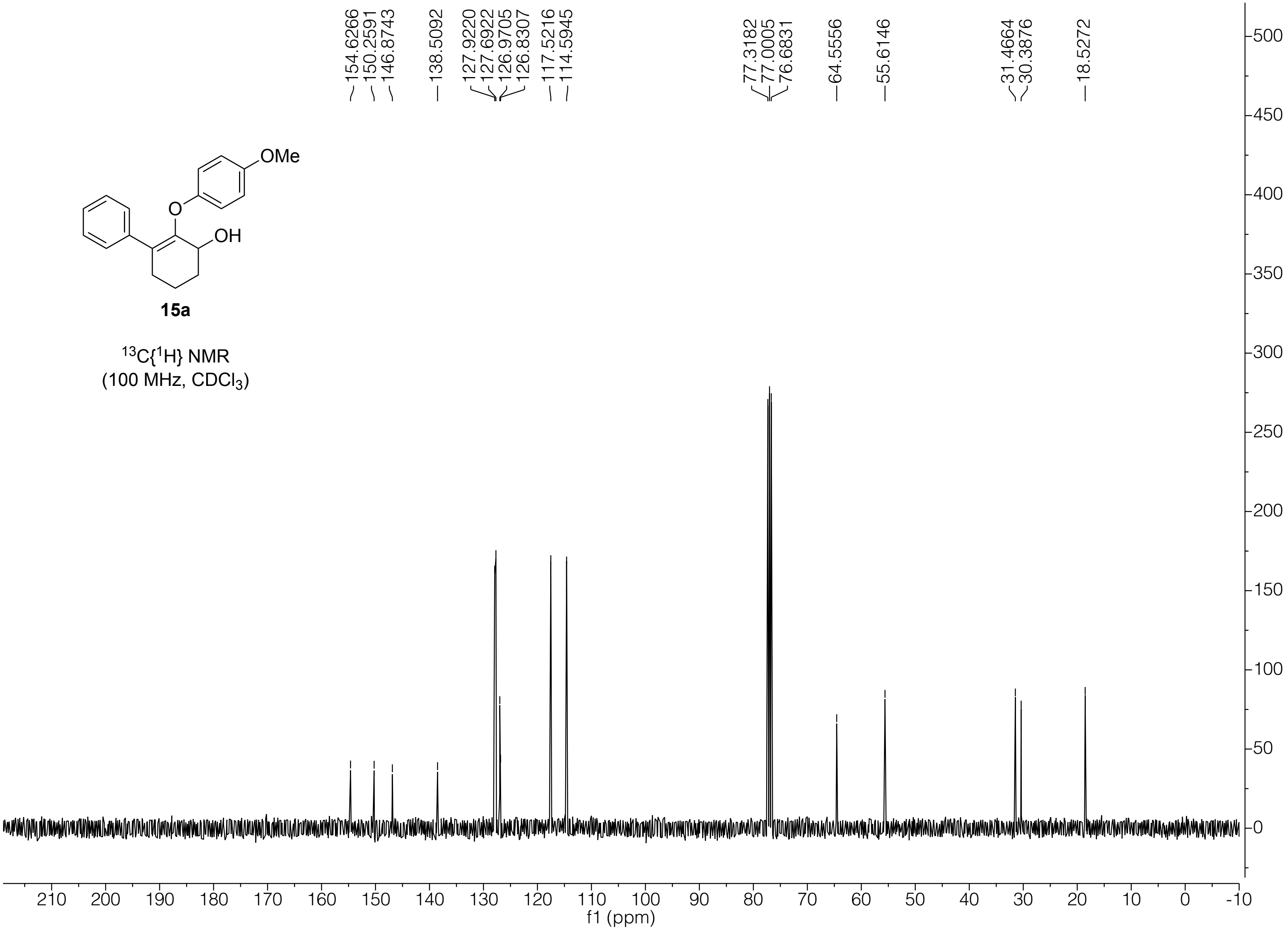
154.6266
150.2591
146.8743
138.5092
127.9220
127.6922
126.9705
126.8307
117.5216
114.5945
77.3182
77.0005
76.6831
64.5556
55.6146
31.4664
30.3876
18.5272
OMe
O
OH
15a
$^{13}C\{^{1}H\}$ NMR
(100 MHz, $CDCl_3$)
f1 (ppm)

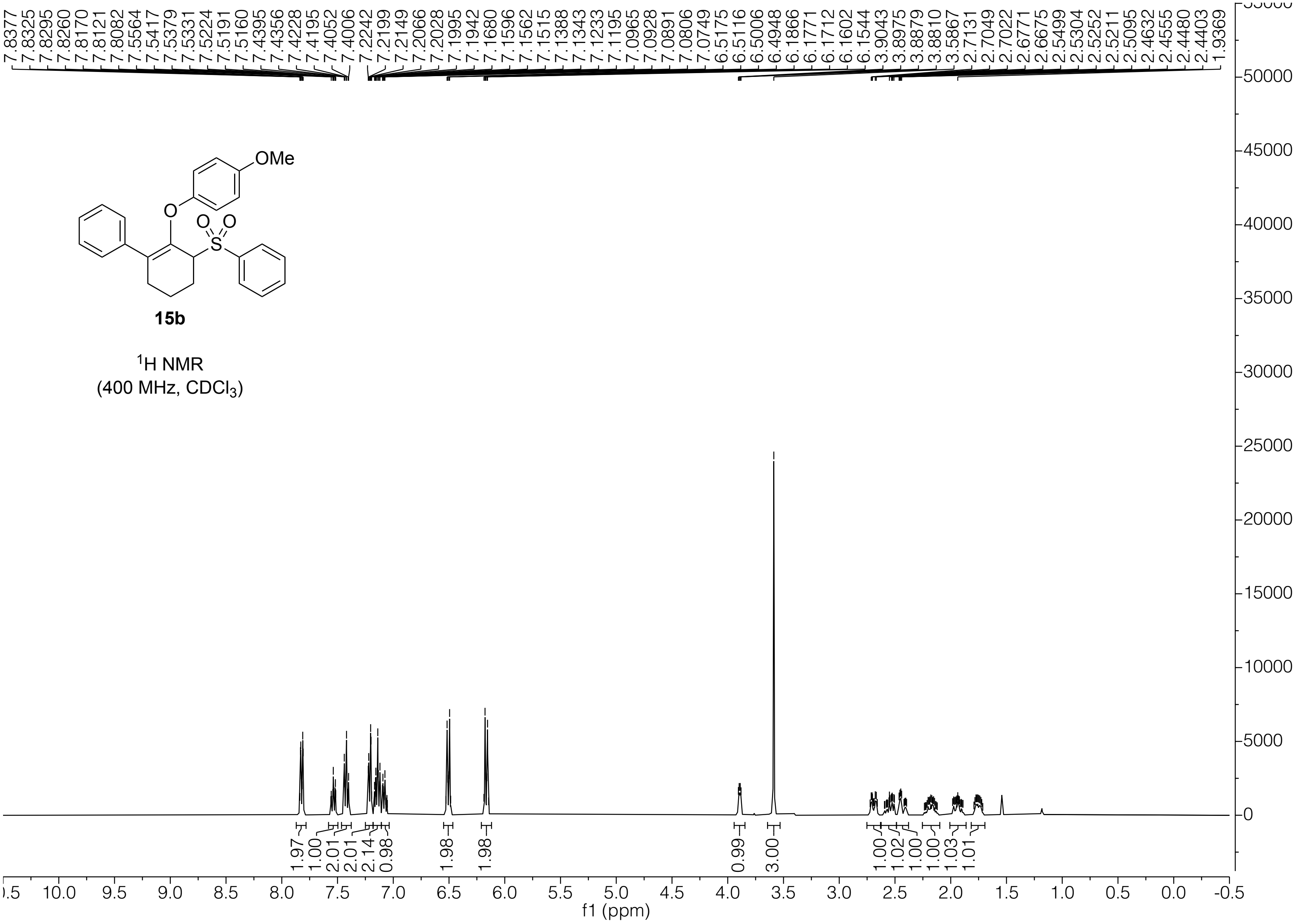
7.8377
7.8325
7.8295
7.8260
7.8170
7.8121
7.8082
7.5564
7.5417
7.5379
7.5331
7.5224
7.5191
7.5160
7.4395
7.4356
7.4228
7.4195
7.4052
7.4006
7.2242
7.2199
7.2149
7.2066
7.2028
7.1995
7.1942
7.1680
7.1596
7.1562
7.1515
7.1388
7.1343
7.1233
7.1195
7.0965
7.0928
7.0891
7.0806
7.0749
6.5175
6.5116
6.5006
6.4948
6.1866
6.1771
6.1712
6.1602
6.1544
3.9043
3.8975
3.8879
3.8810
3.5867
2.7131
2.7049
2.7022
2.6771
2.6675
2.5499
2.5304
2.5252
2.5211
2.5095
2.4632
2.4555
2.4480
2.4403
1.9369
OMe
O
O
S
O
15b
1H NMR
(400 MHz, CDCl3)
1.97
1.00
2.01
2.01
2.14
0.98
1.98
1.98
0.99
3.00
1.00
1.02
1.00
1.00
1.03
1.01
f1 (ppm)

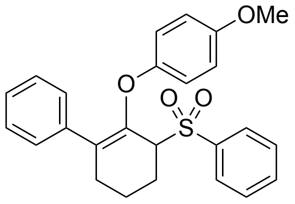

15b

$^{13}C\{^{1}H\}$ NMR
(100 MHz, $CDCl_3$)

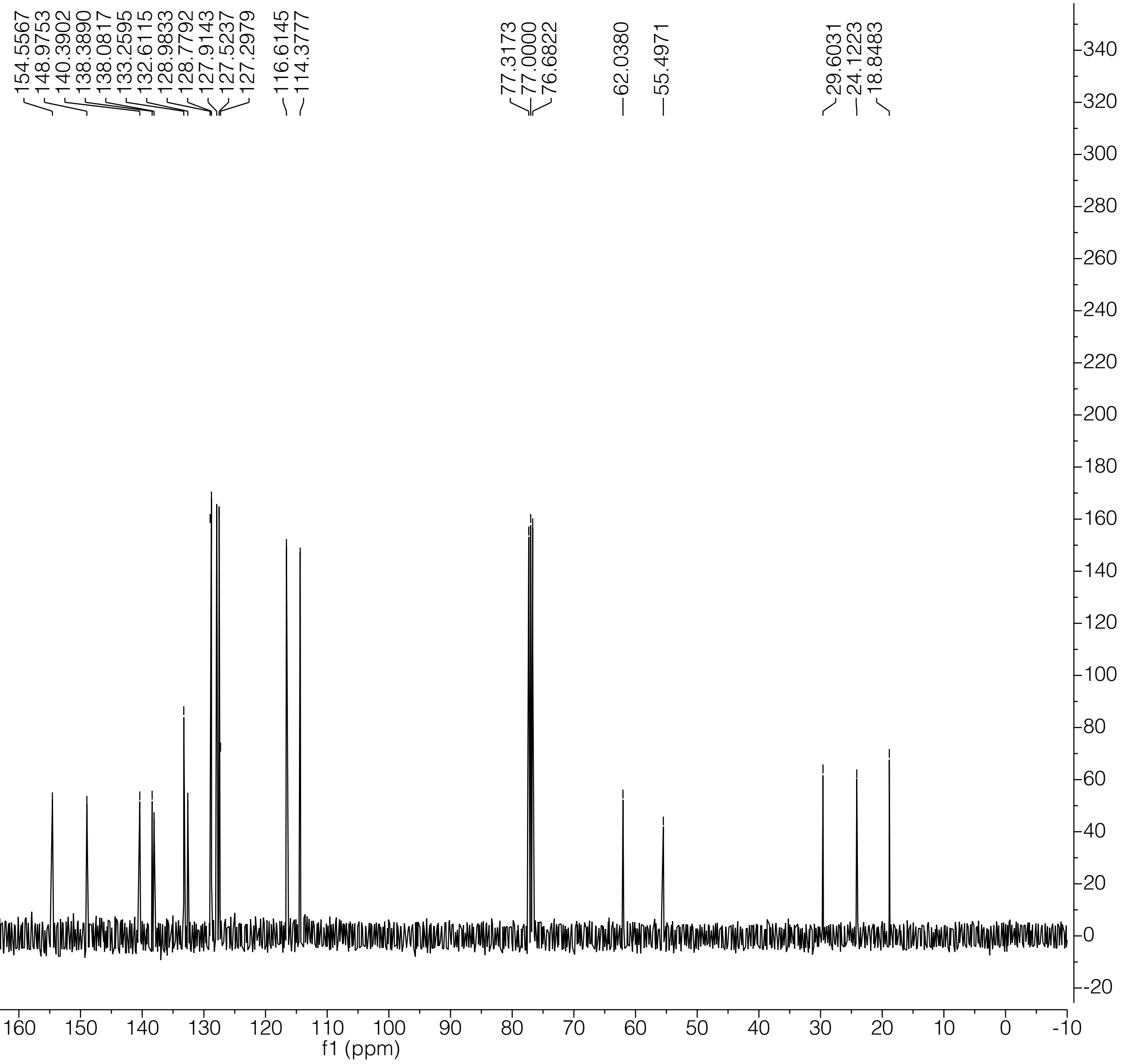

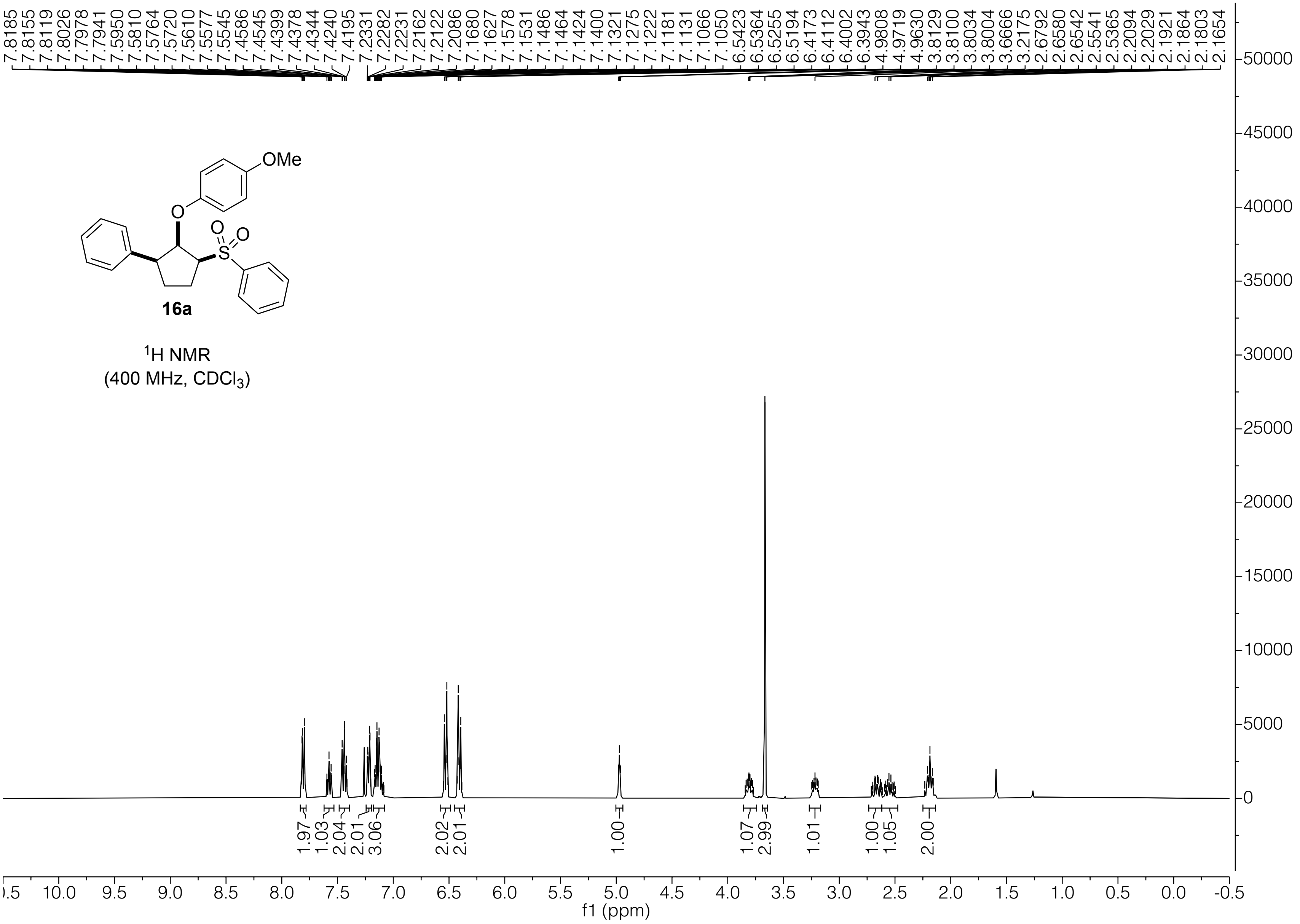
7.8185 7.8155 7.8119 7.8026 7.7978 7.7941 7.5950 7.5810 7.5764 7.5720 7.5610 7.5577 7.5545 7.4586 7.4545 7.4399 7.4378 7.4344 7.4240 7.4195 7.2331 7.2282 7.2231 7.2162 7.2122 7.2086 7.1680 7.1627 7.1578 7.1531 7.1486 7.1464 7.1424 7.1400 7.1321 7.1275 7.1222 7.1181 7.1131 7.1066 7.1050 6.5423 6.5364 6.5255 6.5194 6.4173 6.4112 6.4002 6.3943 4.9808 4.9719 4.9630 3.8129 3.8100 3.8034 3.8004 3.6666 3.2175 2.6792 2.6580 2.6542 2.5541 2.5365 2.2094 2.2029 2.1921 2.1864 2.1803 2.1654
OMe
O
S
O
O
16a
^{1}H NMR
(400 MHz, CDCl$_3$)
1.97 1.03 2.04 2.01 3.06 2.02 2.01 1.00 1.07 2.99 1.01 1.00 1.05 2.00
10.0 9.5 9.0 8.5 8.0 7.5 7.0 6.5 6.0 5.5 5.0 4.5 4.0 3.5 3.0 2.5 2.0 1.5 1.0 0.5 0.0 -0.5
f1 (ppm)
50000 45000 40000 35000 30000 25000 20000 15000 10000 5000 0

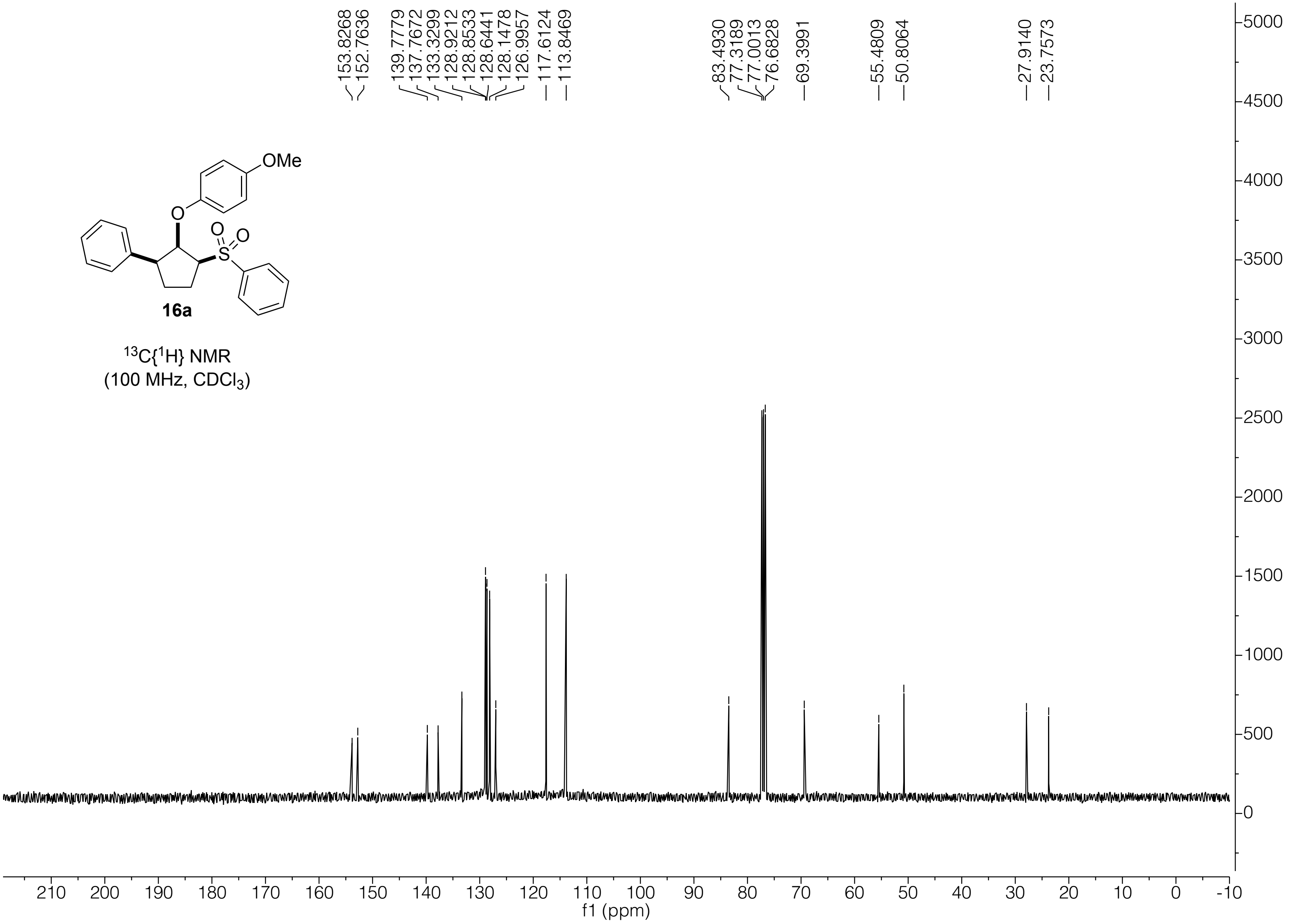

153.8268
152.7636
139.7779
137.7672
133.3299
128.9212
128.8533
128.6441
128.1478
126.9957
117.6124
113.8469
83.4930
77.3189
77.0013
76.6828
69.3991
55.4809
50.8064
27.9140
23.7573
OMe
O
S
O
O
16a
$^{13}C\{^1H\}$ NMR
(100 MHz, $CDCl_3$)
f1 (ppm)

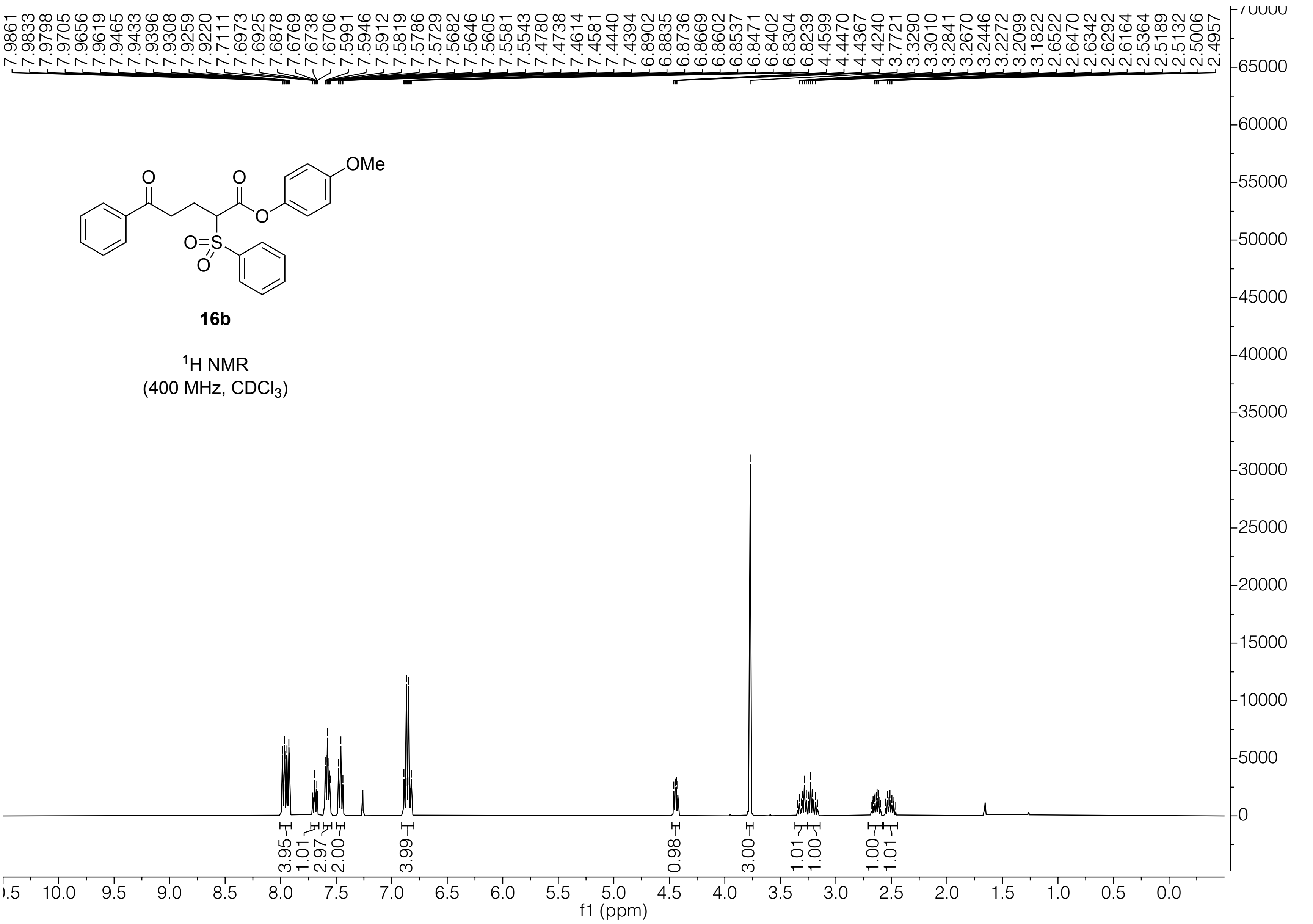
7.9861
7.9833
7.9798
7.9705
7.9656
7.9619
7.9465
7.9433
7.9396
7.9308
7.9259
7.9220
7.7111
7.6973
7.6925
7.6878
7.6769
7.6738
7.6706
7.5991
7.5946
7.5912
7.5819
7.5786
7.5729
7.5682
7.5646
7.5605
7.5581
7.5543
7.4780
7.4738
7.4614
7.4581
7.4440
7.4394
6.8902
6.8835
6.8736
6.8669
6.8602
6.8537
6.8471
6.8402
6.8304
6.8239
4.4599
4.4470
4.4367
4.4240
3.7721
3.3290
3.3010
3.2841
3.2670
3.2446
3.2272
3.2099
3.1822
2.6522
2.6470
2.6342
2.6292
2.6164
2.5364
2.5189
2.5132
2.5006
2.4957
OMe
O
O
O
O=S
O
16b
1H NMR
(400 MHz, CDCl3)
3.95
1.01
2.97
2.00
3.99
0.98
3.00
1.01
1.00
1.00
1.01
f1 (ppm)

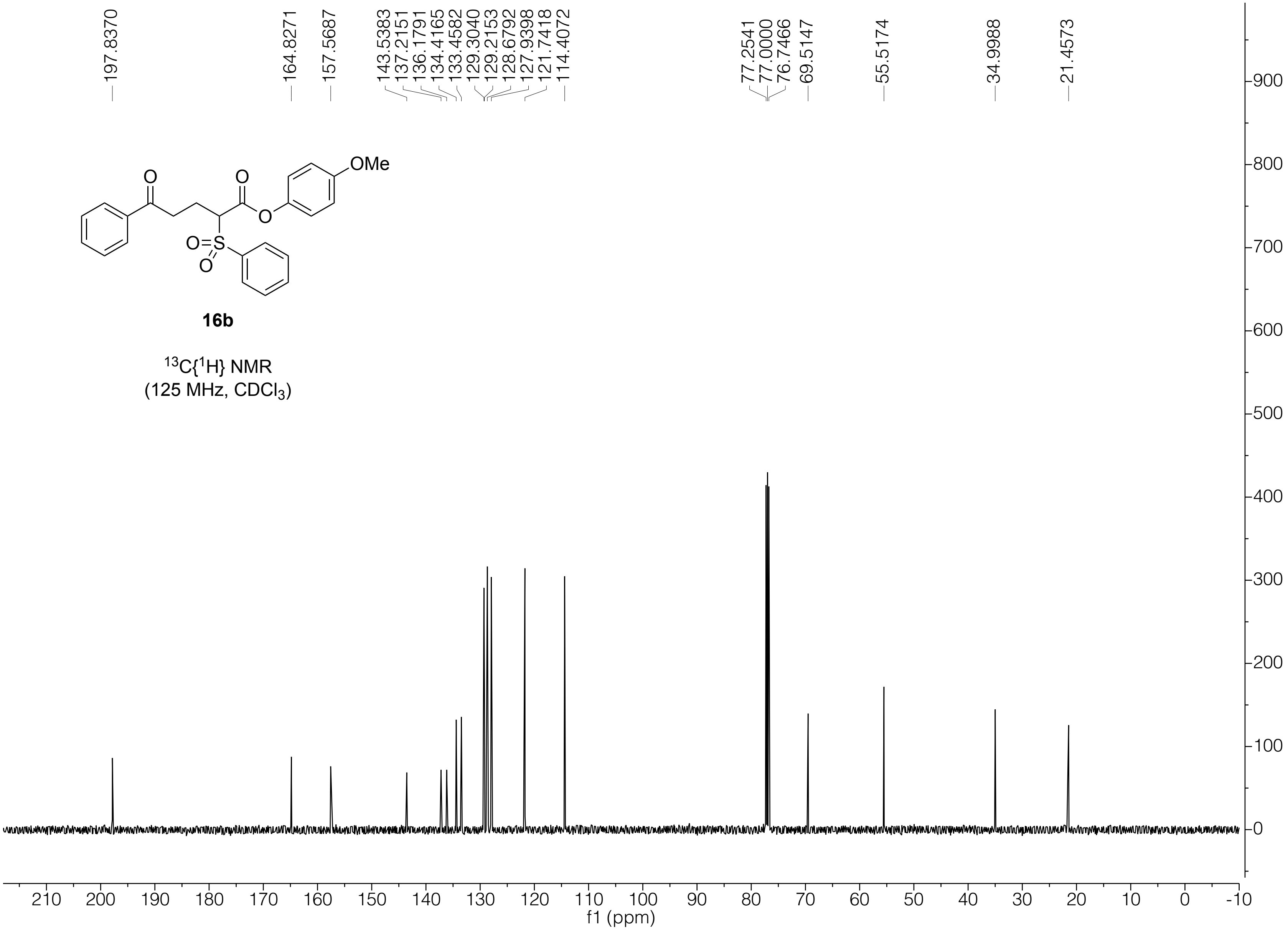
197.8370
164.8271
157.5687
143.5383
137.2151
136.1791
134.4165
133.4582
129.3040
129.2153
128.6792
127.9398
121.7418
114.4072
77.2541
77.0000
76.7466
69.5147
55.5174
34.9988
21.4573
OMe
O
O
O
O=S
O
16b
13C{1H} NMR
(125 MHz, CDCl3)
f1 (ppm)

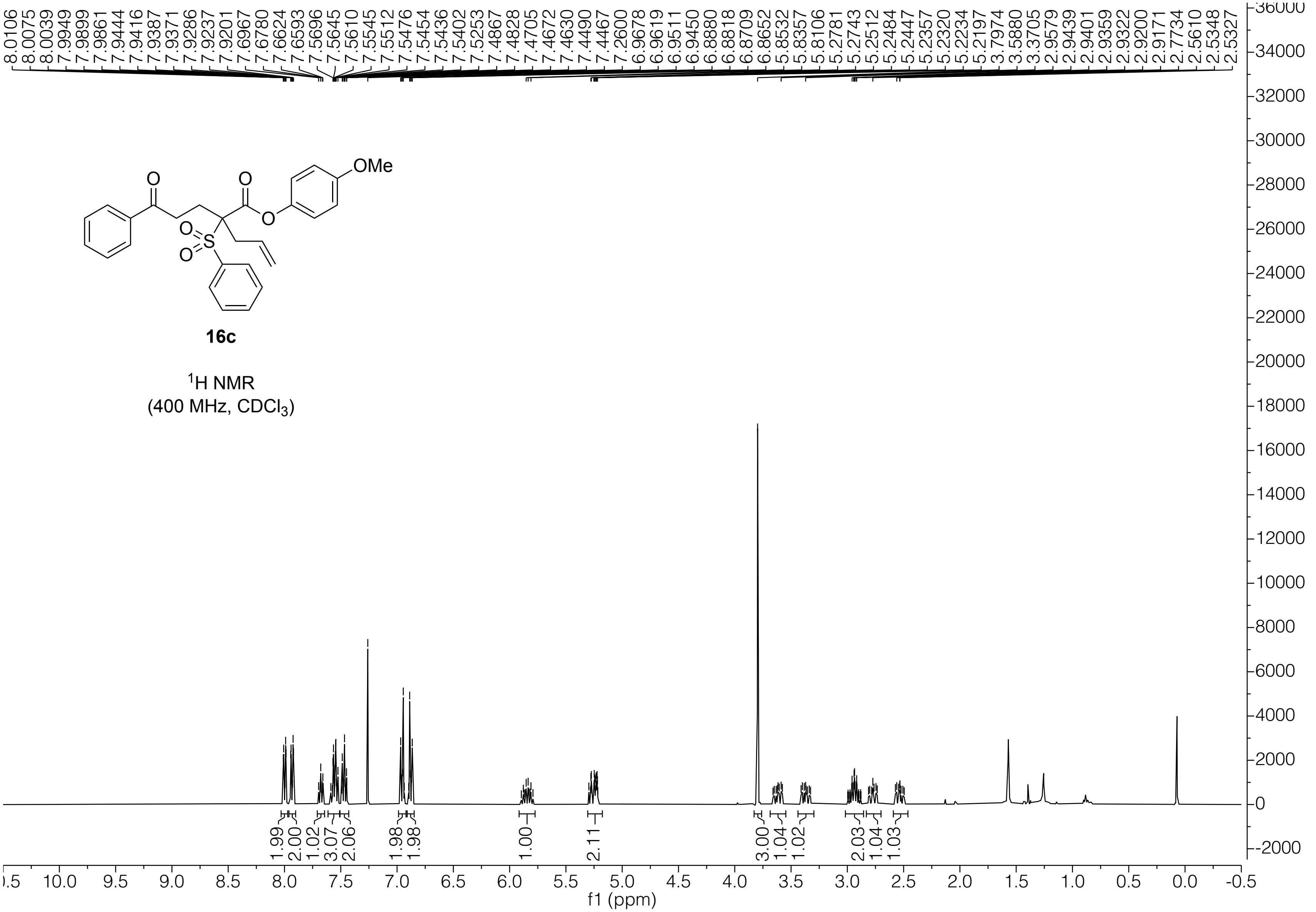

JAB1228_400.4.fid —

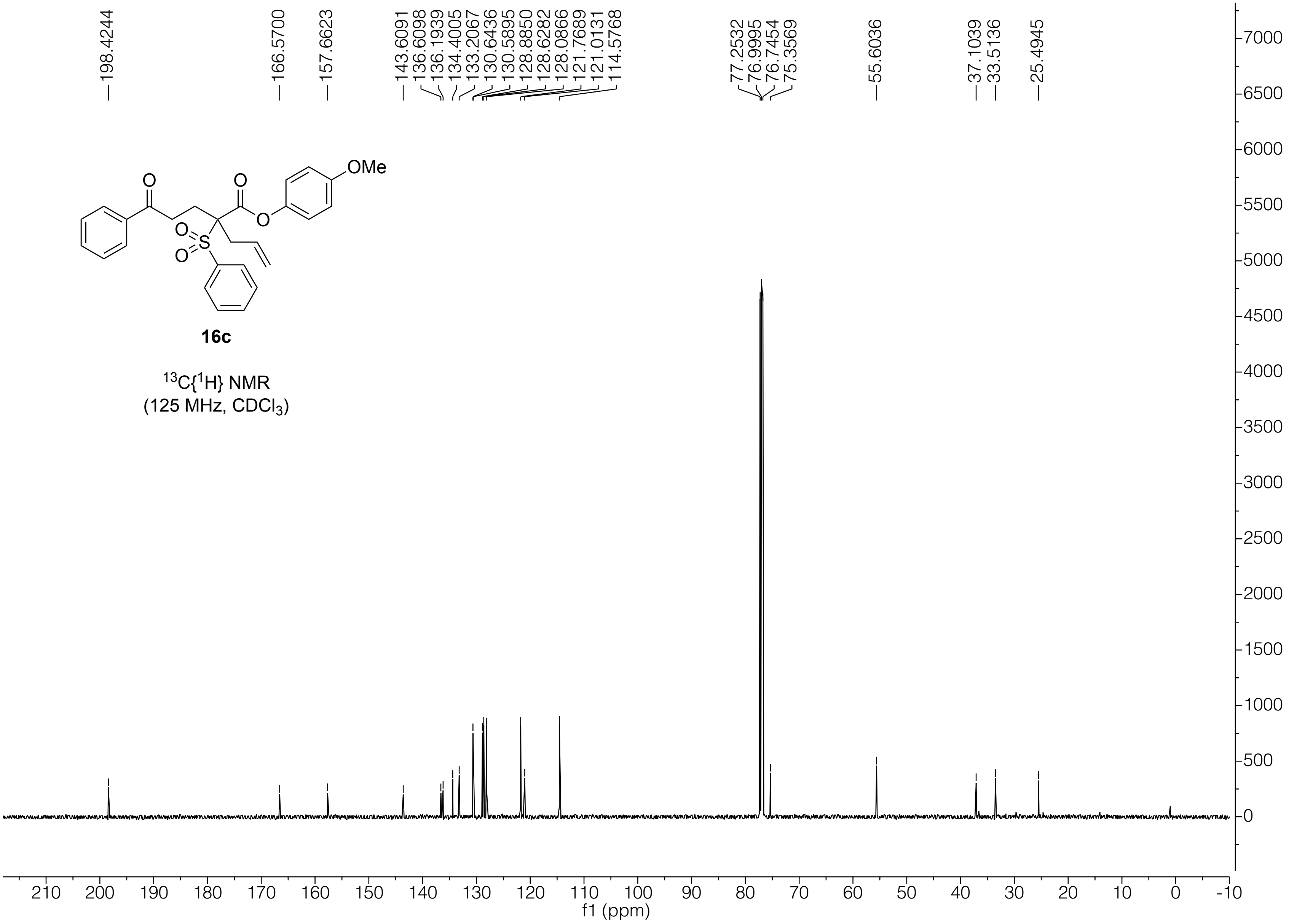
198.4244
166.5700
157.6623
143.6091
136.6098
136.1939
134.4005
133.2067
130.6436
130.5895
128.8850
128.6282
128.0866
121.7689
121.0131
114.5768
77.2532
76.9995
76.7454
75.3569
55.6036
37.1039
33.5136
25.4945
OMe
16c
$^{13}C\{^{1}H\}$ NMR
(125 MHz, $CDCl_3$)
f1 (ppm)

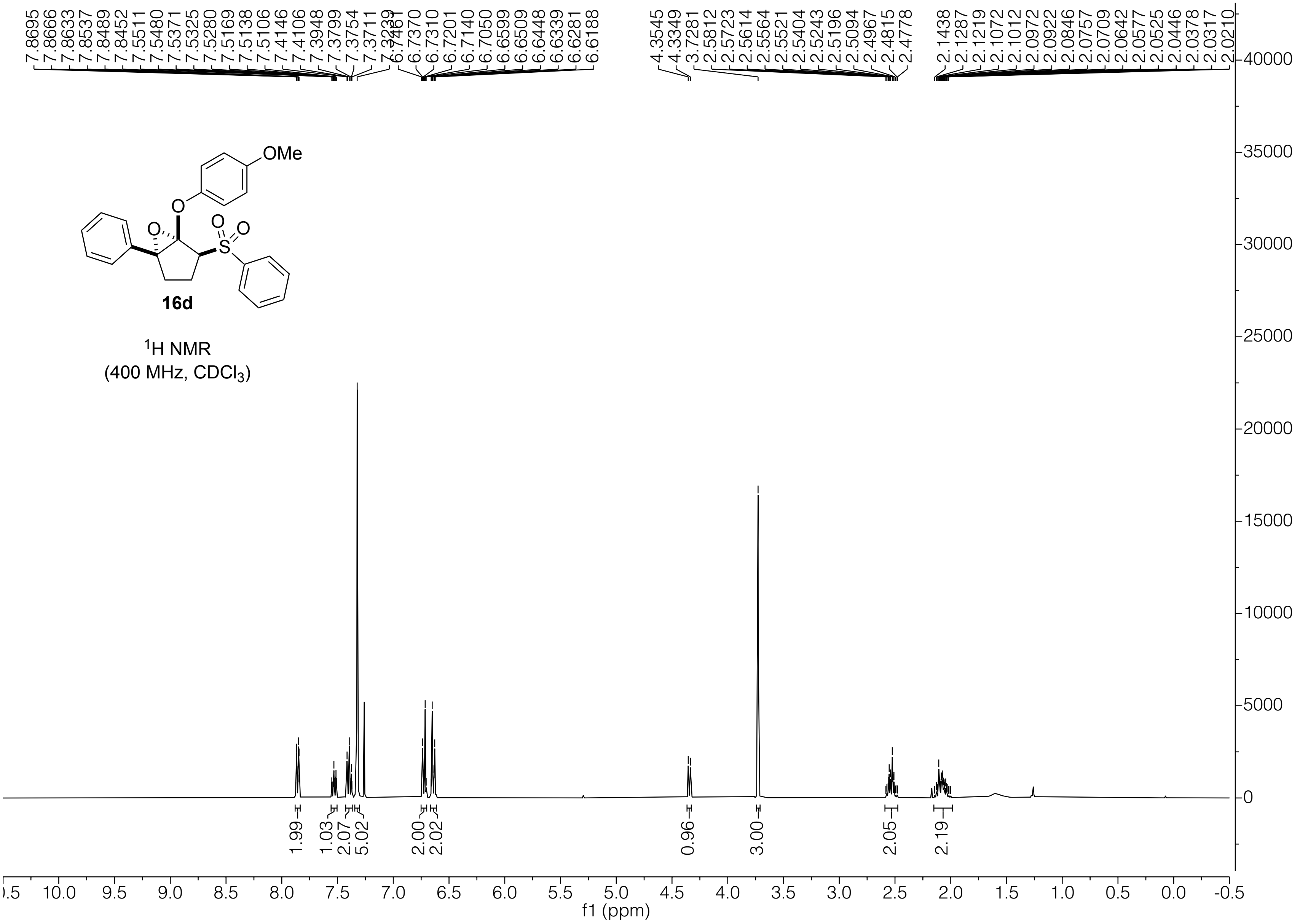
OMe
O
O
S
O
O
16d
^{1}H NMR
(400 MHz, $CDCl_3$)
f1 (ppm)

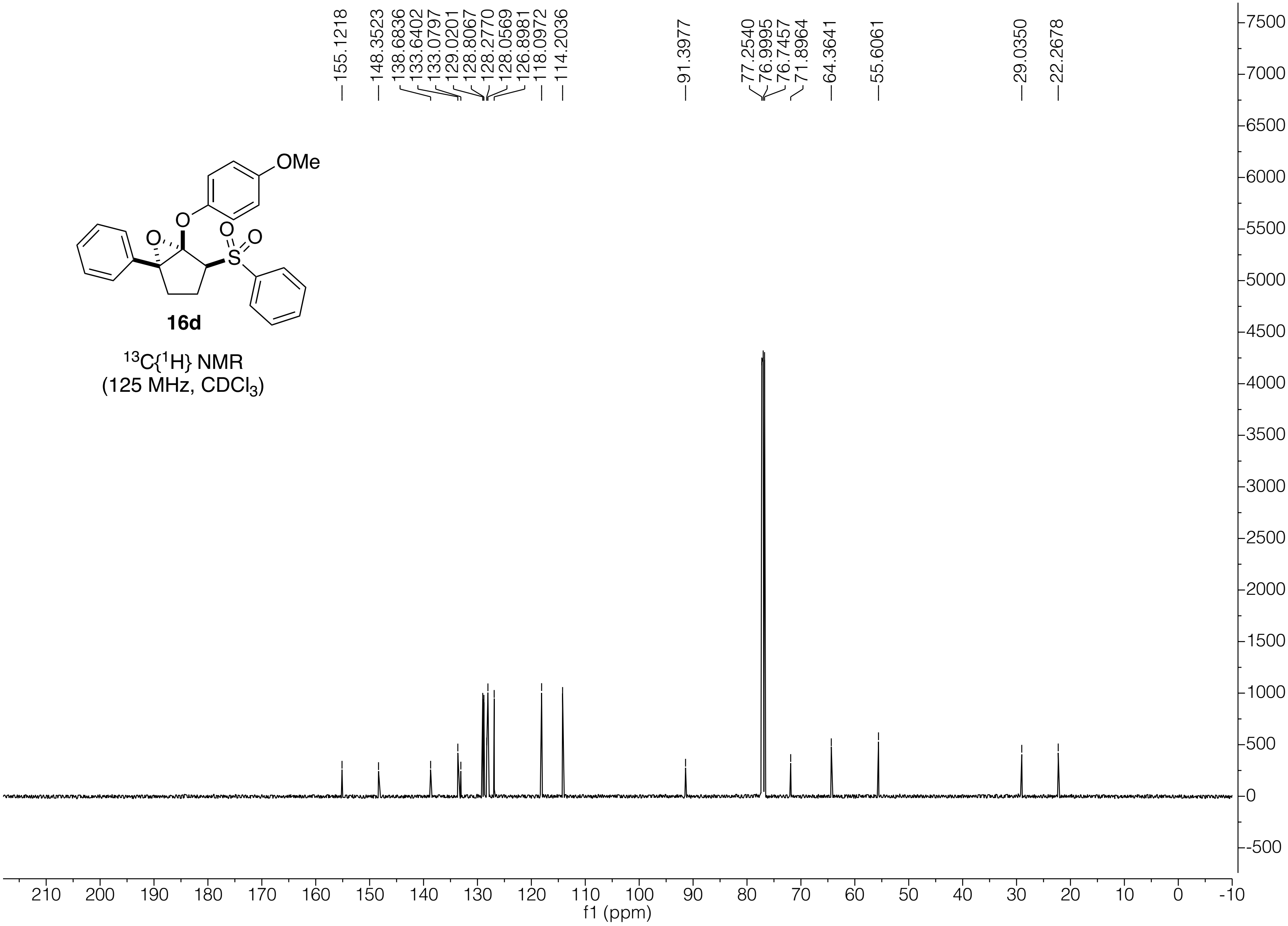

16d

$^{13}C\{^{1}H\}$ NMR
(125 MHz, $CDCl_3$)

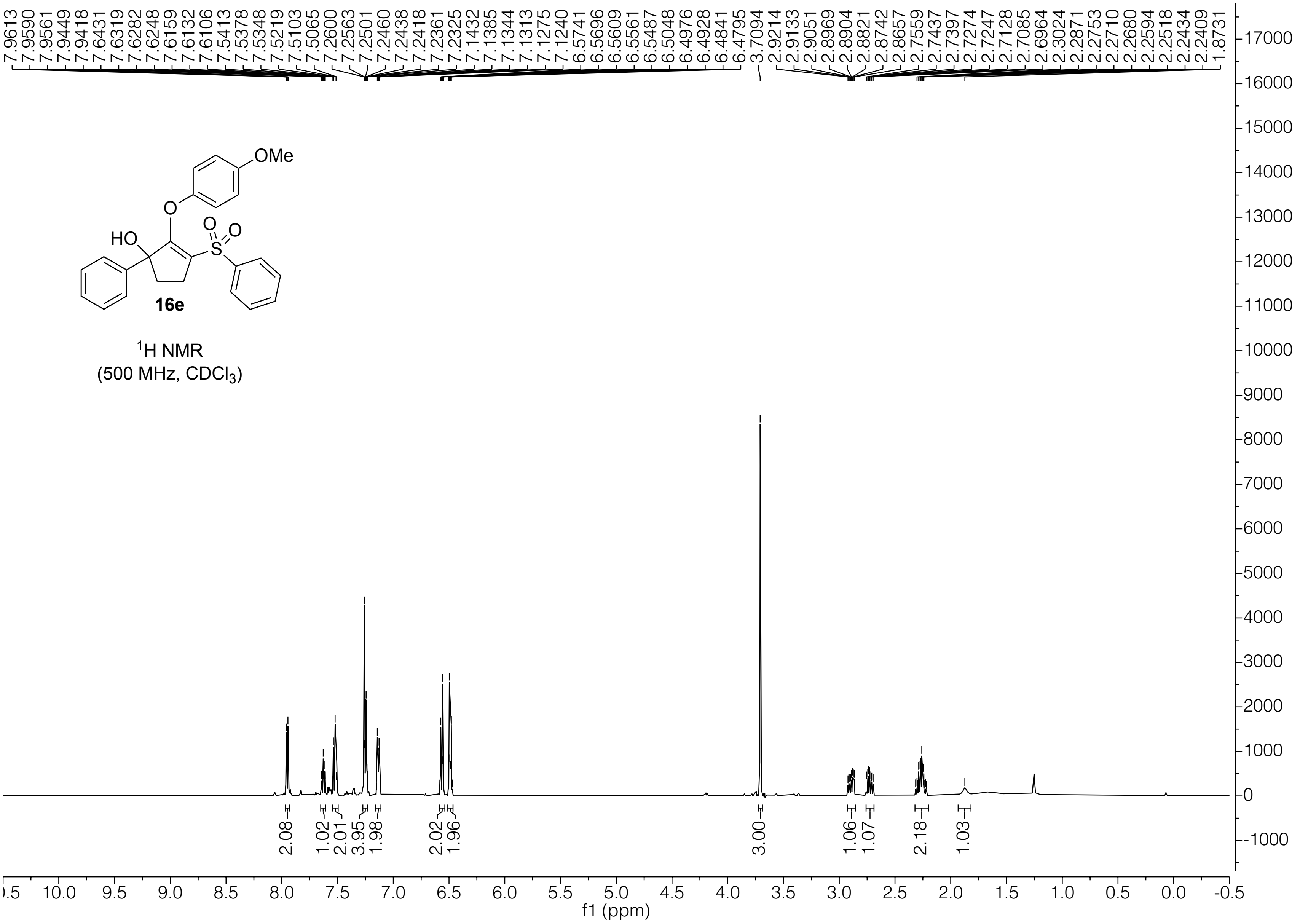
OMe
HO
16e
^{1}H NMR
(500 MHz, $CDCl_3$)
f1 (ppm)

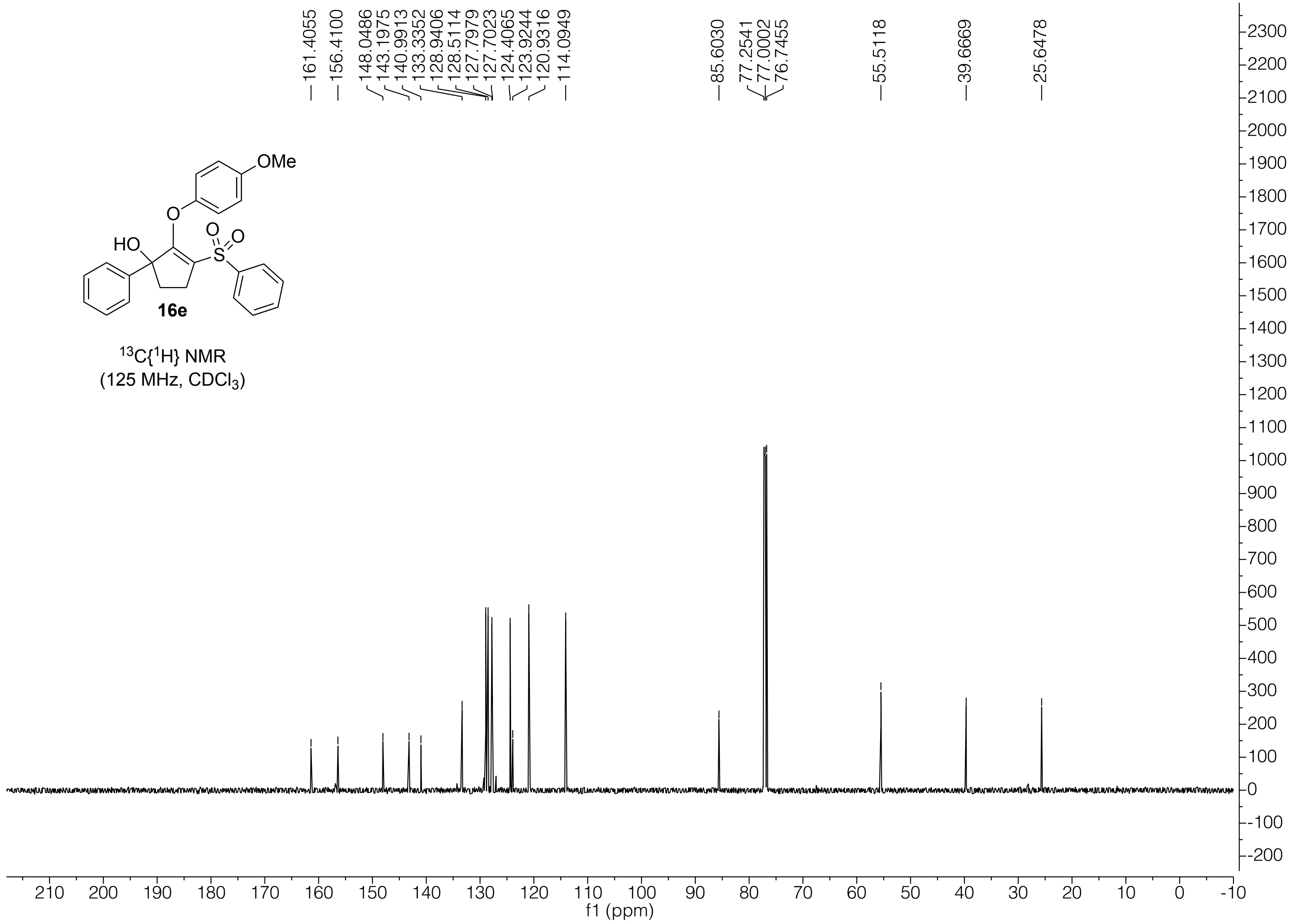
161.4055
156.4100
148.0486
143.1975
140.9913
133.3352
128.9406
128.5114
127.7979
127.7023
124.4065
123.9244
120.9316
114.0949
85.6030
77.2541
77.0002
76.7455
55.5118
39.6669
25.6478
OMe
O
HO
O
S
O
16e
$^{13}C\{^1H\}$ NMR
(125 MHz, $CDCl_3$)
f1 (ppm)

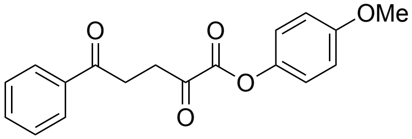

16f

^{1}H NMR
(400 MHz, $CDCl_3$)

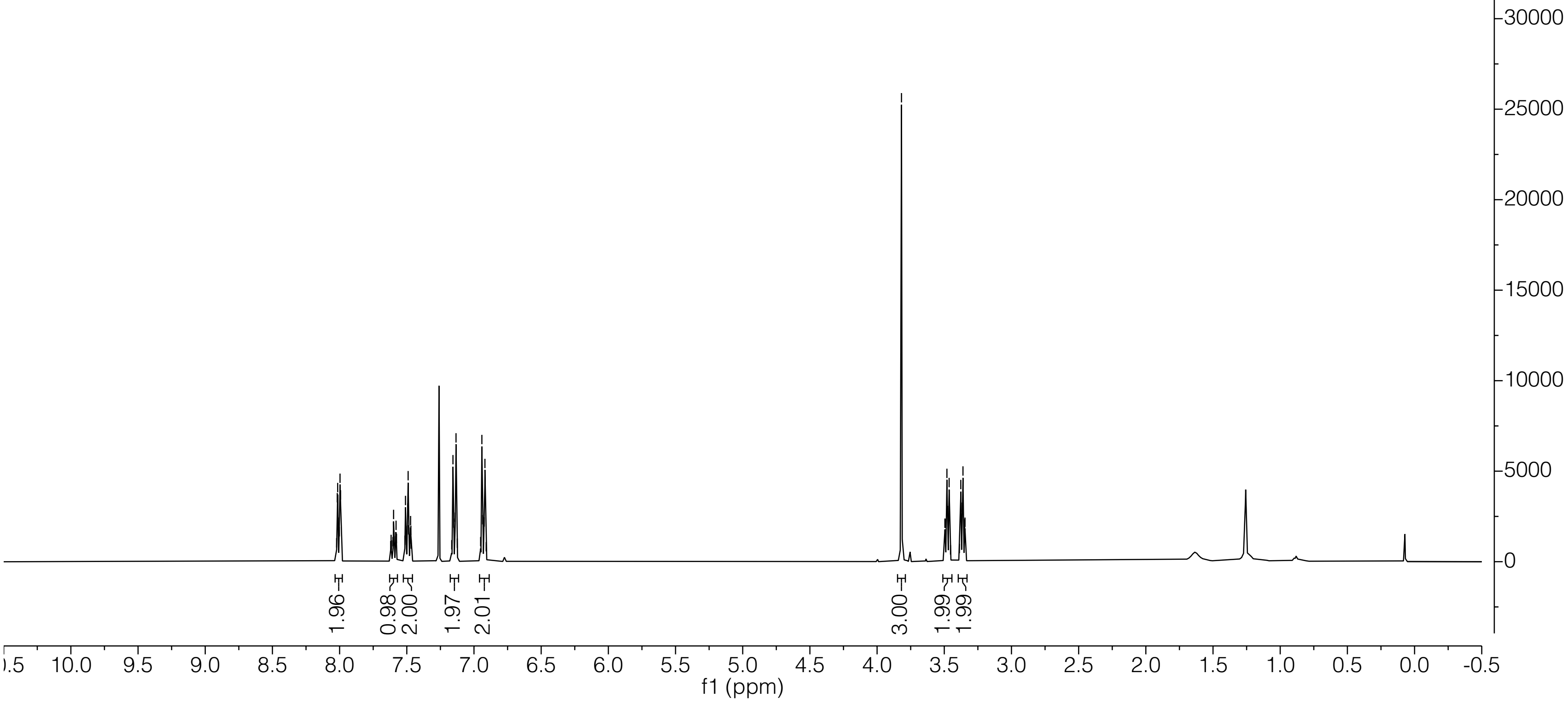

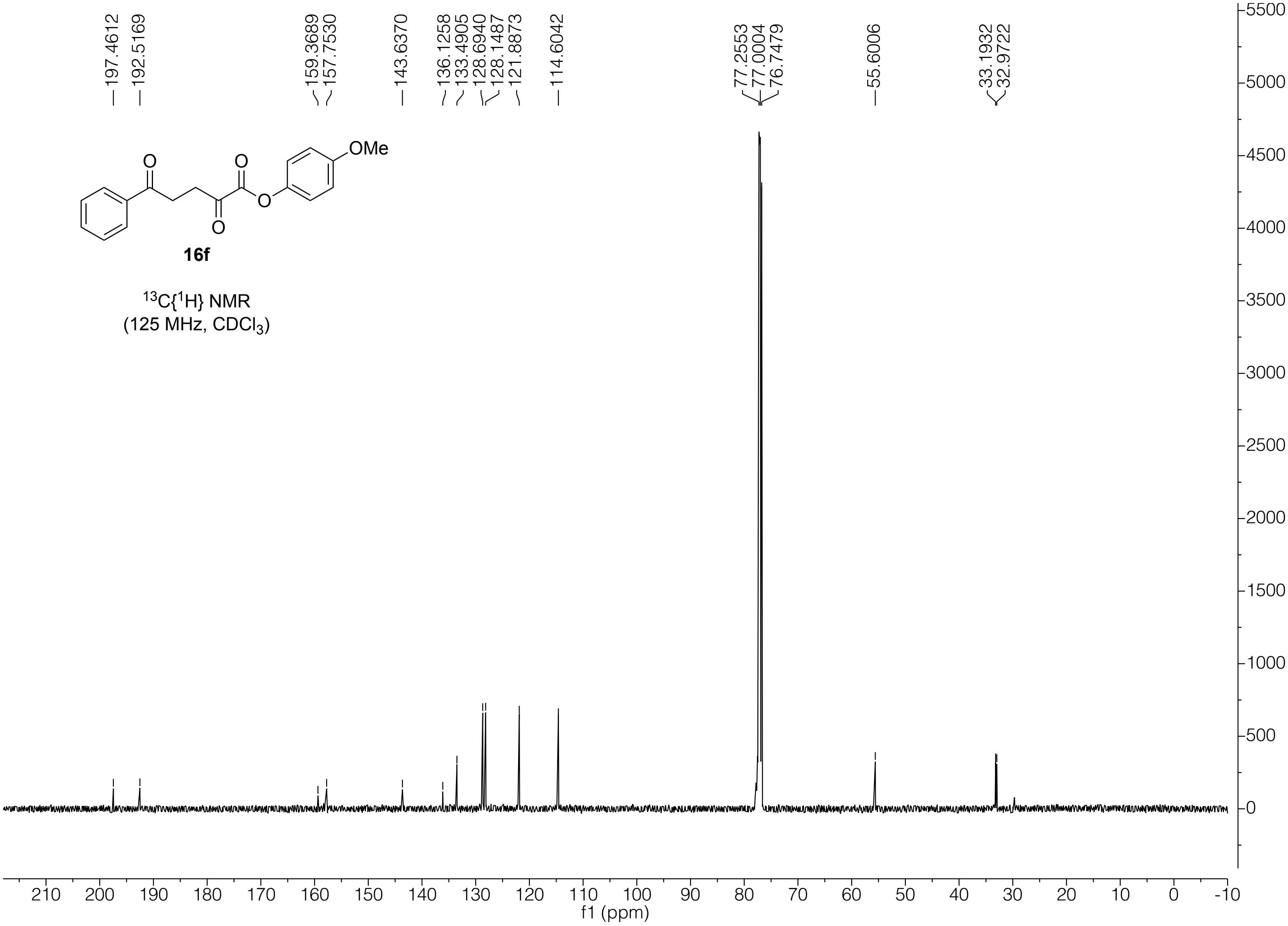

197.4612
192.5169
159.3689
157.7530
143.6370
136.1258
133.4905
128.6940
128.1487
121.8873
114.6042
77.2553
77.0004
76.7479
55.6006
33.1932
32.9722
OMe
16f
$^{13}C\{^{1}H\}$ NMR
(125 MHz, $CDCl_3$)
f1 (ppm)
